# A Lewis Base Catalysis Approach for the Photoredox Activation of Boronic Acids and Esters

**DOI:** 10.1002/anie.201709690

**Published:** 2017-10-24

**Authors:** Fabio Lima, Upendra K. Sharma, Lars Grunenberg, Debasmita Saha, Sandra Johannsen, Joerg Sedelmeier, Erik V. Van der Eycken, Steven V. Ley

**Affiliations:** ^1^ Department of Chemistry University of Cambridge Lensfield Road Cambridge CB2 1EW UK; ^2^ Laboratory for Organic and Microwave-Assisted Chemistry (LOMAC) Department of Chemistry University of Leuven (KU Leuven) Celestijnenlaan 200F 3001 Leuven Belgium; ^3^ Novartis Pharma AG Novartis Campus 4002 Basel Switzerland; ^4^ Peoples Friendship University of Russia (RUDN University) Miklukho-Maklaya street 6 117198 Moscow Russia

**Keywords:** boronic acids, cross-coupling, Lewis base catalysis, photoredox catalysis, synthetic methods

## Abstract

We report herein the use of a dual catalytic system comprising a Lewis base catalyst such as quinuclidin‐3‐ol or 4‐dimethylaminopyridine and a photoredox catalyst to generate carbon radicals from either boronic acids or esters. This system enabled a wide range of alkyl boronic esters and aryl or alkyl boronic acids to react with electron‐deficient olefins via radical addition to efficiently form C−C coupled products in a redox‐neutral fashion. The Lewis base catalyst was shown to form a redox‐active complex with either the boronic esters or the trimeric form of the boronic acids (boroxines) in solution.

Carbon‐centered radicals are a synthetically powerful class of reactive intermediates.^1^ They are particularly attractive in the context of C−C bond‐forming reactions,^2^ overcoming problems often associated with two‐electron processes.^3^ By enabling visible‐light‐promoted single electron transfer, photoredox catalysis has become a method of choice for the single‐electron reduction or oxidation of organic substrates and allows to generate open‐shell intermediates in a mild and selective fashion.^4^ A range of reductive or oxidative carbon radical precursors are now available to generate carbon radicals in the context of a photocatalytic cycle.^5^ Oxidative carbon radical precursors are often anionic species suffering from poor solubility in common organic solvents. For example, extensively studied organoborates^6^ possess an electron‐rich B(sp^3^) moiety that can be subjected to single‐electron oxidation, leading to a neutral carbon radical after C−B bond cleavage (Scheme 1 A).

**Scheme 1:**
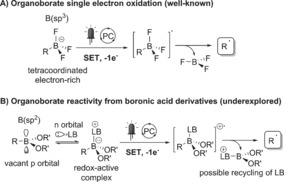
Photoredox activation of organoboron reagents. LB=Lewis base.

Despite their ubiquity as reagents in organic synthesis^7^ and in biologically active molecules,^8^ the use of boronic acid derivatives to generate carbon‐centered radicals remains underexplored.^9^ Owing to their high oxidation potentials, they have received much less attention in this regard, with few reports making use of strong stoichiometric oxidants or anodic oxidation.^10^ We recently demonstrated that benzyl boronic esters can undergo single‐electron oxidation under photoredox conditions when their vacant p orbital is engaged in a dative bond with the n orbital of a stoichiometric Lewis base (LB) additive (Scheme 1 B).^11^ Lewis base catalysis was introduced as a concept by Denmark to enhance the reactivity of electrophilic n*, π*, and σ* orbitals.^12^ Based on this knowledge, we hypothesized that the use of a catalytic amount of an organic Lewis base would be a viable option for the photoredox activation of boronic acids and esters.^13^

Herein, we describe a dual catalytic method to effectively form alkyl and aryl radicals from a wide array of boronic esters and acids by direct photoredox single‐electron oxidation under mild and safe conditions, without the requirement for stoichiometric activators or oxidants. These reactive species were further engaged in intermolecular C−C bond‐forming processes to deliver desirable C(sp^3^)−C(sp^3^) and C(sp^2^)−C(sp^3^) bonds in a redox‐neutral fashion.

The addition of electron‐rich carbon‐centered radicals onto electron‐deficient olefins, also known as Giese‐type addition,^14^ is an interesting method to form C−C bonds in a redox‐neutral fashion and can also be used to assess the presence of the postulated radical intermediates.^15^ We initially subjected model boronic ester **1 a** to an excess of methyl acrylate (**2 a**) in the presence of 1.5 equiv of 4‐dimethylaminopyridine (DMAP) as an additive and the photoredox catalyst PC(1), which we have already shown to be quenched by DMAP‐activated **1 a**.^11^ Irradiation of this mixture with blue LEDs for 24 h readily led to the coupling product **3 aa** in 86 % yield. Reducing the DMAP catalyst loading to 20 mol % still provided **3 aa** in 75 % yield (Scheme 2), with the remaining mass balance resulting from oligomerization due to multiple acrylate additions. Pleased by this level of catalytic activity, we decided to investigate other Lewis bases.

**Scheme 2:**
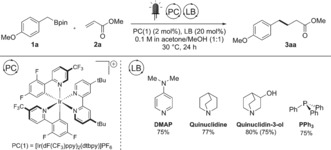
Optimized reaction conditions for the Giese‐type addition of boronic ester **1 a** to acrylate **2 a**, highlighting the results obtained with selected Lewis base catalysts. The optimization was conducted with 0.1 mmol of **1 a** and 0.4 mmol of **2 a**. Yields of **3 aa** determined by ^1^H NMR analysis of the crude reaction mixture with CH_2_Br_2_ as an internal standard. Yield of isolated product given in parentheses.

According to Denmark's theory, n–n* interactions are the most productive type of activation for a Lewis base catalyst to be active,^12^ so a range of commercial neutral Lewis bases with an available non‐bonding n orbital were screened at 20 mol % loading. Strongly nucleophilic^16^ quinuclidine‐derived bases such as quinuclidin‐3‐ol and quinuclidine were identified as productive catalysts, leading to the formation of **3 aa** in 80 % (75 % upon isolation) and 77 % yield, respectively. Phosphine‐derived Lewis bases were also investigated, with triphenylphosphine (PPh_3_) showing good activity. Control experiments revealed the necessity of blue light irradiation, photocatalyst, Lewis base, and methanol for the successful conversion of boronic esters (see the Supporting Information for the full optimization and control experiments).

With optimized reaction conditions in hand, we assessed the scope with electron‐deficient alkenes **2 a**–**2 r** (Scheme 3). Aside from methyl acrylate, *tert*‐butyl and benzyl acrylate are also suitable coupling partners (**3 aa**–**3 ac**). Methyl vinyl ketone was identified as the best coupling partner, with the conjugate addition product being isolated in 82 % yield (**3 ad**). Pleasingly, acrolein and acrylonitrile coupling products (**3 ae** and **3 af**) were also obtained in high yields, thereby expanding the range of functional groups tolerated with this method. *gem*‐Disubstituted olefins also reacted in a radical conjugate addition, and methyl methacrylate (**3 ah**), a conjugated lactam (**3 ai**), and two cyclic enones (**3 aj** and **3 ak**) were selectively coupled in 58–68 % yield. Interestingly, 2‐ and 4‐vinylpyridines were successfully alkylated at the β‐carbon atom (**3 am** and **3 an**), providing examples of reactions with challenging N‐heteroaromatic compounds.^17^ These results could be extended to a 2‐pyridyl‐containing 1,1‐disubstituted olefin (**3 ao**), showcasing the possibility to generate pheniramine analogues and the potential application of the method for antihistaminic drug discovery.^18^ Finally, flavone natural products can also be alkylated, albeit in lower yield (**3 aq** and **3 ar**).

**Scheme 3:**
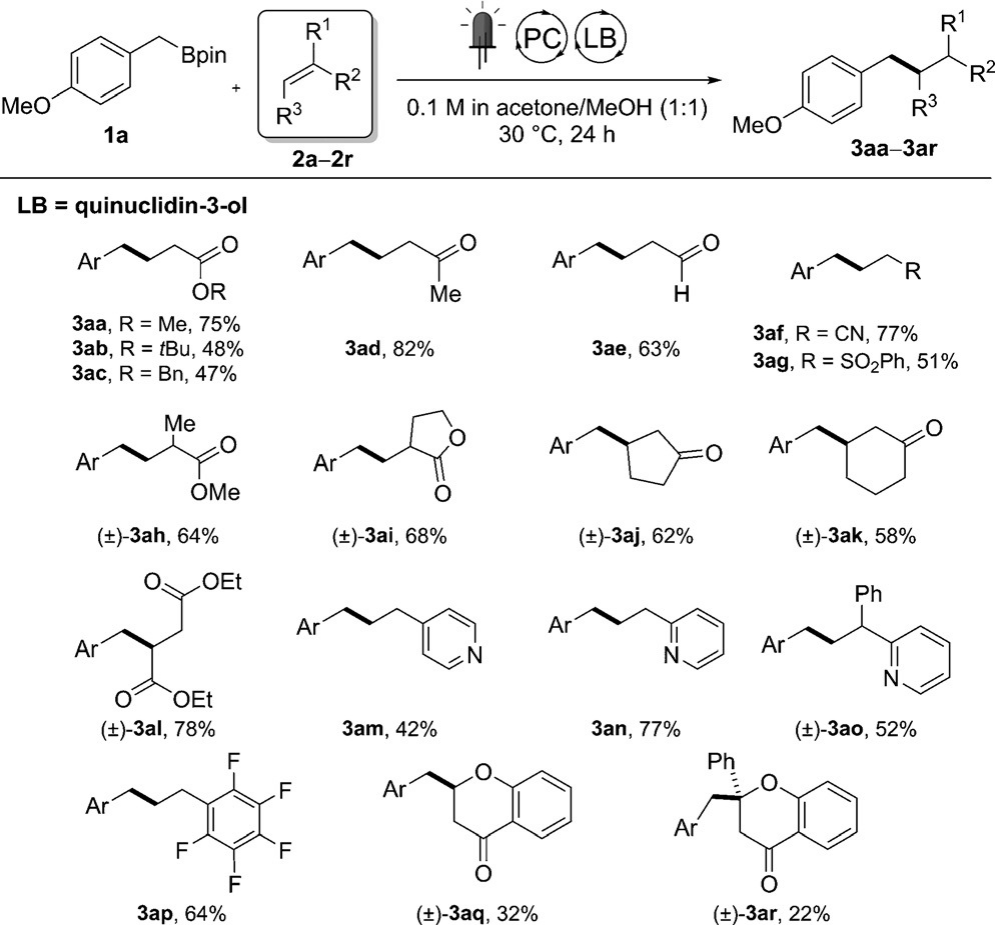
Scope with electron‐deficient alkenes. Yields of isolated products are given. Ar=4‐MeOC_6_H_4_. Reaction conditions: **1 a** (0.2 mmol), **2 a**–**2 r** (0.4–0.8 mmol), PC(1) (2 mol %), quinuclidin‐3‐ol (20 mol %), irradiation supplied by a commercial blue LED strip (14.4 W at 450 nm).

We next turned our attention to establishing the scope with respect to boronic ester coupling partners with methyl vinyl ketone **2 d** (Scheme 4). Primary benzylic pinacol esters were selectively coupled (**3 ad**–**3 ed**) in the presence of quinuclidin‐3‐ol as the Lewis base catalyst. Interestingly, α‐heteroatom‐substituted primary alkyl boronic esters were also coupled in high yields (**3 fd**–**3 hd**, 86–91 %), with triphenylphosphine proving to be the most efficient catalyst for the α‐amino products **3 gd** and **3 hd**. More sterically demanding secondary benzylic esters required the use of DMAP as the Lewis base catalyst, highlighting the effect of the steric hindrance on the required initial complexation between boronic ester and Lewis base. Whereas methyl (**3 id** and **3 jd**) and benzyl (**3 kd**) substituents were well tolerated, the presence of larger isopropyl (**3 ld**) or phenyl (**3 md**) groups led to less efficient coupling. Lastly, tertiary boronic esters were explored (**3 nd**–**3 pd**). Despite their well‐known difficulty to be efficiently engaged in metal‐catalyzed cross‐couplings,14a, 15, 19 DMAP allowed for clean activation to form quaternary carbon centers in respectable yields even from commercial and less activated *t*BuBpin (**3 pd**).

**Scheme 4:**
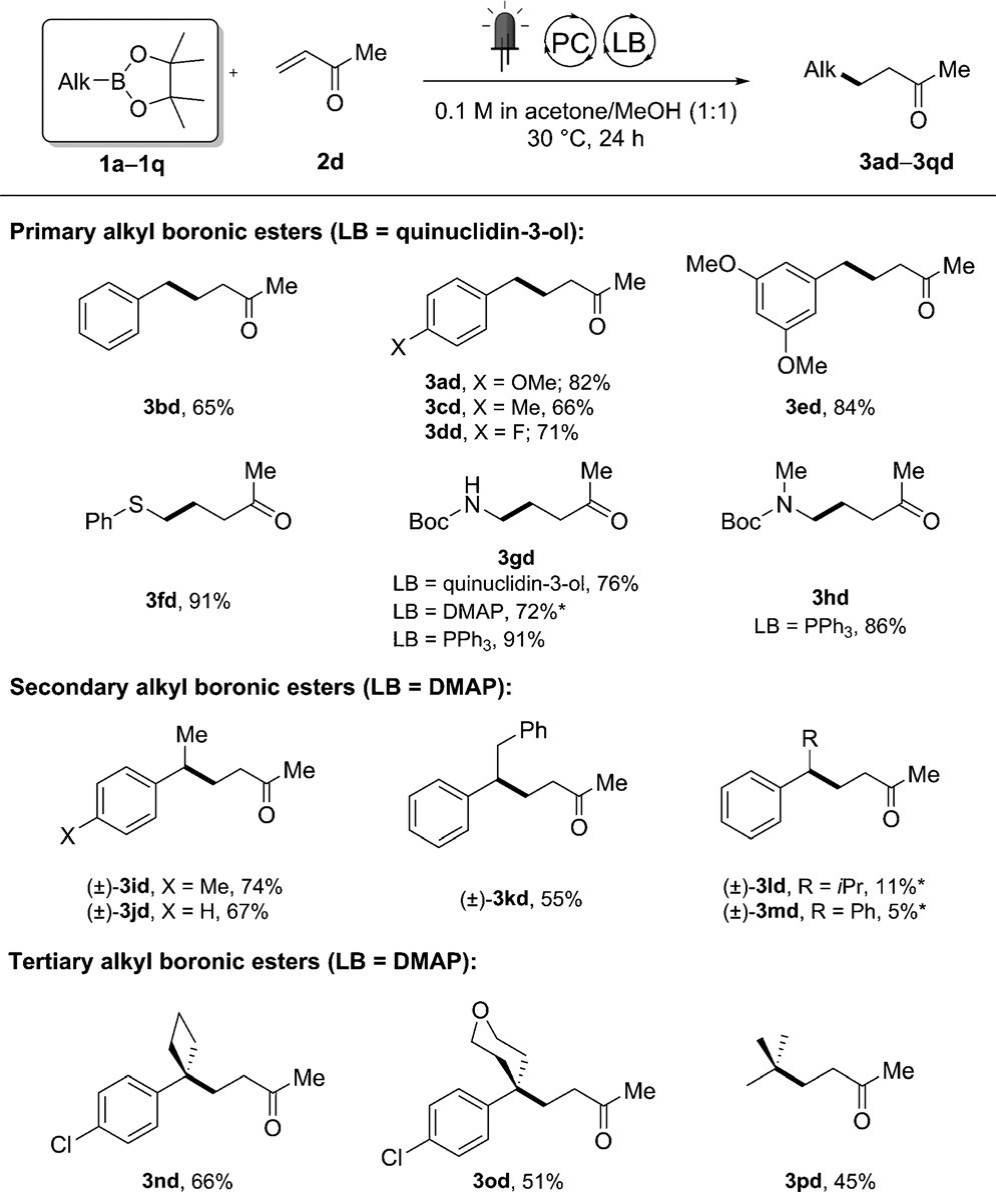
Scope with boronic pinacol esters. Yields of isolated products are given. Reaction conditions: **1 a–1 q** (0.2 mmol), **2 d** (0.8 mmol), PC(1) (2 mol %), LB (20 mol %), irradiation supplied by a commercial blue LED strip (14.4 W at 450 nm). * Yield determined by ^1^H NMR analysis of the crude reaction mixture with CH_2_Br_2_ as an internal standard.

Aryl boronic esters, on the other hand, were found to be substantially less reactive than their activated alkyl counterparts. We initially observed only low reactivity after 24 h of irradiation, and therefore surveyed different aryl‐substituted B(sp^2^) species to find that aryl boronic acids were more reactive than the corresponding pinacol, glycol, neopentyl, and catechol esters (see the Supporting Information). Our experience with the Lewis acidity of aryl boronic acids led us to propose that the reactive species in solution was more likely to be the trimeric boroxine than the monomeric species.^20^ This was confirmed by NMR experiments showing the complexation of quinuclidin‐3‐ol with boroxine instead of the corresponding free boronic acid (see the Supporting Information). This finding led us to screen a series of commercially available boronic acids in this reaction (Scheme 5).

**Scheme 5:**
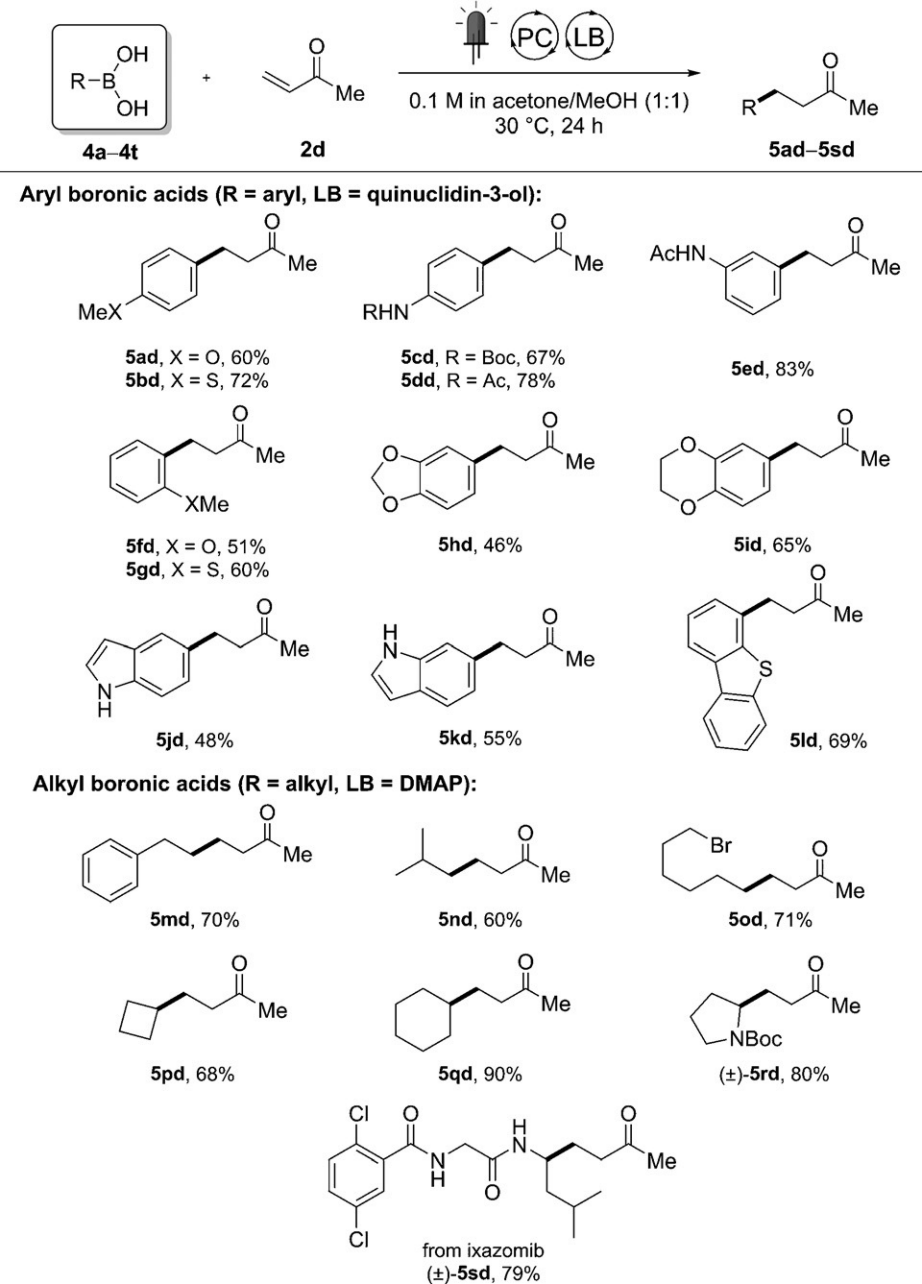
Scope with boronic acids. Yields of isolated products are given. Reaction conditions: **4 a**–**4 s** (0.2 mmol), **2 d** (0.8 mmol), PC(1) (2 mol %), LB (20 mol %), irradiation supplied by a commercial blue LED strip (14.4 W at 450 nm).

Despite the usually harsh reaction conditions employed to oxidize aryl boronic acids,10b we found that a large number of electron‐rich aryl boronic acids could be successfully coupled to **2 d** under extremely mild and redox‐efficient conditions. The couplings of aryl boronic acids with nitrogen (**5 cd**–**5 ed**), oxygen (**5 ad** and **5 fd**), and sulfur (**5 bd** and **5 gd**) substituents on the ring all proceeded in good to excellent yields. Oxygen‐containing heterocycles derived from catechol could be incorporated into the substrates (**5 hd** and **5 id**), and unprotected 5‐ and 6‐indoyl boronic acids (**5 jd** and **5 kd**) were also successfully functionalized in the presence of nucleophilic NH and C3 centers. The enhanced reactivity observed with boroxines relative to boronic esters encouraged us to attempt using unactivated alkyl boronic acids as starting materials. Primary alkyl boronic acids were successfully coupled (**5 md**–**5 od**) along with secondary alkyl derivatives (**5 pd** and **5 qd**), showcasing the usefulness of this method to generate functional unstabilized alkyl radicals.5a Secondary α‐amino boronic acids derived from amino acids7d were also well tolerated, with proline‐derived **5 rd** as well as the peptide drug ixazomib transformed in high yield (**5 sd**), illustrating the potential application to late‐stage functionalization.

According to NMR studies, a fast, dynamic equilibrium is established between the boroxine **6 a**′ derived from boronic acid **6 a** or boronic ester **6 b** and the Lewis base catalyst (LB) in the reaction solvent mixture (see the Supporting Information). Cyclic voltammetry measurements informed us that complex **7** can be single‐electron‐oxidized (*E*_1/2_ (**1 a**‐DMAP)=+0.81 V vs. SCE) within the reductive quenching cycle of PC(1) (*E*_1/2_ (Ir^III*^)=+1.2 V vs. SCE).^21^ The carbon radical thus generated (**8**) undergoes a radical addition with **10** to form the intermediate radical **11**, which can then be reduced and quenched by a proton from methanol to provide coupling product **13** (Scheme 6).6c The resulting methanolate can then be used to regenerate the LB from **9**.

**Scheme 6:**
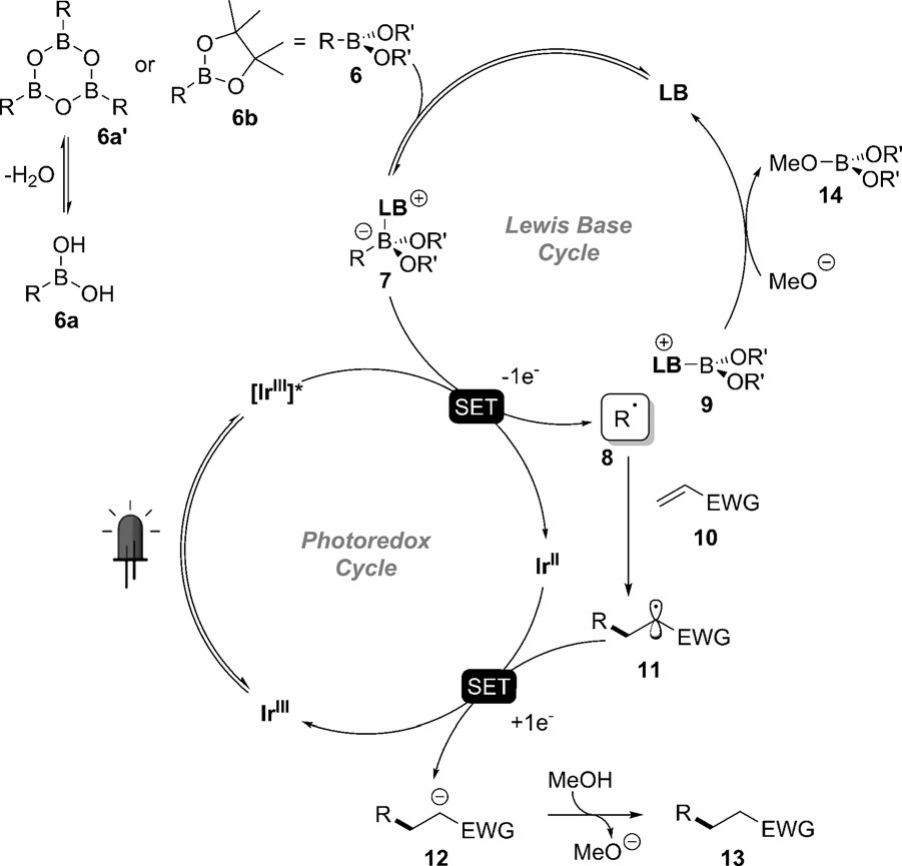
Possible mechanism for the Lewis base and photoredox catalyzed Giese‐type addition of boronic acid derivatives.

In conclusion, we have developed a new set of photoredox reaction conditions taking advantage of the Lewis acidity of boronic esters and boroxines (from boronic acids) to generate primary, secondary, and tertiary alkyl or aryl radicals. These intermediates were engaged in redox‐neutral C−C couplings with electron‐deficient olefins, forming a range of new C(sp^3^)−C(sp^3^) and C(sp^2^)−C(sp^3^) cross‐coupled products. Over 50 structurally and functionally diverse products were successfully synthesized. This new activation method should enable the use of boronic acids and esters in a wide range of other radical‐based reactions.

## Conflict of interest

The authors declare no conflict of interest.

## Supporting information

As a service to our authors and readers, this journal provides supporting information supplied by the authors. Such materials are peer reviewed and may be re‐organized for online delivery, but are not copy‐edited or typeset. Technical support issues arising from supporting information (other than missing files) should be addressed to the authors.

Supplementary
