# Supplementary material for: A Lewis Base Catalysis Approach for the Photoredox Activation of Boronic Acids and Esters
Source: Angew Chem Int Ed Engl. 2017 Oct 24;56(47):15136–40. doi: 10.1002/anie.201709690 (PMC5708277; doi:10.1002/anie.201709690)

Angewandte Chemie
Eine Zeitschrift der Gesellschaft Deutscher Chemiker

Supporting Information

A Lewis Base Catalysis Approach for the Photoredox Activation of Boronic Acids and Esters

Fabio Lima, Upendra K. Sharma, Lars Grunenberg, Debasmita Saha, Sandra Johannsen, Joerg Sedelmeier, Erik V. Van der Eycken, and Steven V. Ley**

anie_201709690_sm_miscellaneous_information.pdf

Contents

1. General information 3
2. Selected optimisation experiments 5
   - 2.1 Initial Optimisation 5
   - 2.2 Lewis Base Catalyst Screening 6
   - 2.3 Final optimisation for 1a 8
   - 2.4 Aryl boronic species comparison 9
3. Mechanistic experiments 10
   - 3.1 Complexation experiments 10
     - 3.1.1 Boronic ester complexation 10
     - 3.1.2 Boroxine vs. boronic acid complexation 11
   - 3.2 Electrochemical measurements 14
4. Synthesis and characterisation of starting materials 16
   - 4.1 Synthesis of alkene starting materials 16
   - 4.2 Synthesis of boronate starting materials 17
5. Synthesis and characterisation of coupling products 19
   - 5.1 Scope of electron-deficient olefins 19
     - 5.1.1 General procedure I 19
     - 5.1.2 Characterization of coupling products 19
   - 5.2 Alkyl boronic esters scope 27
     - 5.2.1 General Procedure II 27
     - 5.2.2 Characterization of coupling products 27
   - 5.3 Aryl and alkyl boronic acid scope 34
     - 5.3.1 General Procedure III 34
     - 5.3.2 Characterization of coupling products 34
6. References 42
7. NMR spectra 44
   - 7.1 Starting Materials 44
     - 7.1.1 Alkene Starting Materials 44
     - 7.1.2 Boronate Starting Materials 46
   - 7.2 Coupling Products 51
     - 7.2.1 Scope of electron-deficient alkenes 51
     - 7.2.2 Scope of alkyl boronic esters 69
     - 7.2.3 Scope of aryl and alkyl boronic acids 83

1 General information

General methods: All reactions, unless otherwise noted, were performed magnetically stirred under Ar atmosphere using standard Schlenk techniques. Reaction temperatures were electronically monitored as external heating block temperatures. Reagents were purchased from different commercial sources and used without further purification. The removal of solvent under reduced pressure was carried out on a standard rotary evaporator.

Solvents: Et_2O and THF were distilled with sodium and benzophenone under inert gas prior to use. Degassed solvents were degassed by purging with Ar for at least 20 min. Solvents for flash column chromatography and crystallisations were distilled under reduced pressure. *Iso*-hexane mentioned as petrol ether (PE) consist of the boiling fractions between 40 and 50°C.

Chromatography: Analytical thin-layer chromatography was carried out on pre-coated glass plates (silica gel 60 F_{254}) from Merck. Compound spots were visualized under ultraviolet (UV) light (254 nm or 365 nm for fluorescent compounds), ceric ammoniummolybdate (CAM), ninhydrin or $KMnO_4$ stain solutions. For flash column chromatography silica gel 60 from Merck, with a particle size between 40 and 63 µm, was used. Crudes were often loaded onto columns using dry loading technique with Isolute HM-N (Biotage PN: 9800-5000).

NMR spectroscopy: ^{1}H-NMR spectra were recorded on a Bruker Avance DPX-400 or DRX-600 spectrometer at 400 and 600 MHz respectively and are reported as follows: chemical shift δ in ppm (multiplicity, coupling constants *J* in Hz, number of protons, assignment). The multiplicity and shape of the ^{1}H signals are designated by the following abbreviations: s = singlet, d = doublet, t = triplet, q = quartet, m = multiplet, br. = broad, or combinations of thereof. All chemical shifts δ are reported to the nearest 0.01 ppm with the residual solvent peak as the internal reference (chloroform-d = 7.26 ppm, methanol-d^4 = 3.31 ppm, acetone-d^6 = 2.05 ppm, water-d^2 = 4.790 ppm). ^{13}C-NMR spectra were recorded on the same spectrometers at 100 and 150 MHz with ^{1}H decoupling. All ^{13}C resonances are reported to the nearest 0.1 ppm with the central resonance of the solvent peak as the internal reference (chloroform-d = 77.16 ppm, methanol-d^4 = 49.00 ppm, acetone-d^6 = 29.84 ppm). The ^{13}C signal of the carbon bonded to boron was not observed in some cases due to quadrupolar relaxation. ^{19}F-NMR spectra were recorded on a Bruker DPX-400 spectrometer at 376 MHz with ^{1}H decoupling. All chemical shifts δ are reported to the nearest 0.1 ppm with $CFCl_3$ as the external standard ($CFCl_3$ = 0.0 ppm). ^{11}B-NMR NMR spectra were recorded on a Bruker DPX-400 or DRX-600 spectrometer at 128 MHz and 193 MHz respectively with ^{1}H decoupling. All chemical shifts δ are reported to the nearest 0.1 ppm with $BF_3{\cdot}OEt_2$ as the external standard ($BF_3{\cdot}OEt_2$ = 0.0 ppm). Spectra are assigned using ^{1}H-COSY, ^{13}C-DEPT-135 and HMQC where appropriate to facilitate structural determination. The numbering of the proton and carbon atoms does not match the IUPAC nomenclature. Diastereotopic protons in the ^{1}H-NMR spectra are referenced with a and b, nomenclature is arbitrarily and does not correspond to the spin system.

Infrared spectroscopy: Infrared spectra were recorded neat on a PerkinElmer Spectrum One FT-IR spectrometer using Universal ATR sampling accessories. measuring unit. IR data are reported in wavenumbers (cm^{-1}) of absorption.

High-resolution mass spectrometry (HRMS) was performed using a Waters Micromass LCT Premier™ spectrometer using time of flight mass detection and positive ESI ionization method. All reported values are within 5 ppm of the calculated value.

Melting points (m.p.) were recorded on a Stanford Research Systems OptiMelt Automated Melting Point System calibrated against vanillin (m.p. 83 °C), phenacetin (m.p. 136°C) and caffeine (m.p. 237°C).

Photochemical experiments were performed magnetically stirred in 5 mL glass test-tubes, sealed with a rubber septum. The tubes were irradiated with blue light (450 nm) using a coiled commercial LED strip (1 m, from LEDXON, PN: 9009083) with a total power output of 14.4 W. To maintain a constant reaction temperature of 30°C, the setup was cooled by a constant air flow from a clip fan (**Figure S1**).

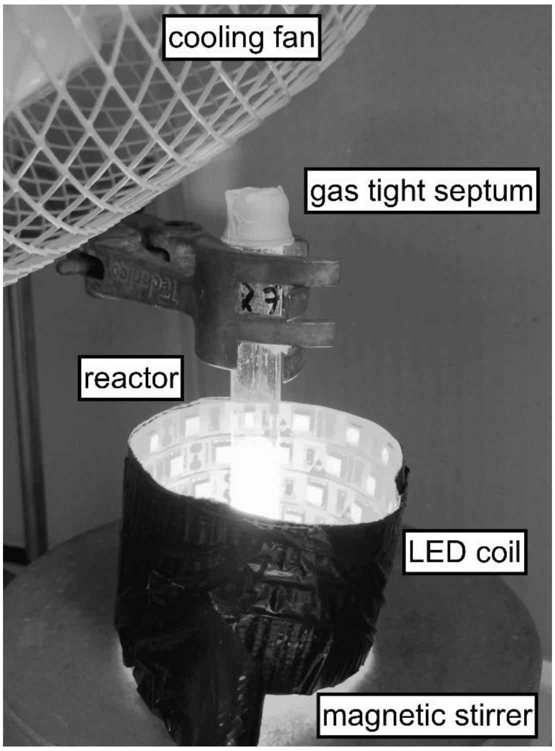

Figure S1 – Photoreactor setup

2 Selected optimisation experiments

2.1 Initial Optimisation

Initial optimization protocol: A 5 mL glass vial equipped with a magnetic stir bar was charged with 2-(4-methoxybenzyl)-4,4,5,5-tetramethyl-1,3,2-dioxaborolane (**1a**, 25 mg, 0.10 mmol), the photoredox catalyst (2 mol%) and DMAP (5 mg, 0.04 mmol, 150 mol%). The vial was then sealed with a rubber septum and evacuated/backfilled with argon three times. Methyl acrylate (**2a**, 36 µL, 0.4 mmol, 4.0 equiv.) was then added followed by 1.0 mL of degassed solvent to lead a clear yellow transparent 0.1 M solution. This solution was then stirred while irradiated with a commercial blue LED strip (14.4 W, 450 nm) for 24 hours, the temperature was maintained at 30°C using a clip fan. After 24 h of irradiation the mixture was concentrated and dried *in vacuo*. Dibromomethane (10 µL, exact mass measured on balance) was added to the dry crude mixture as internal standard to determine the NMR yield in **3aa**.

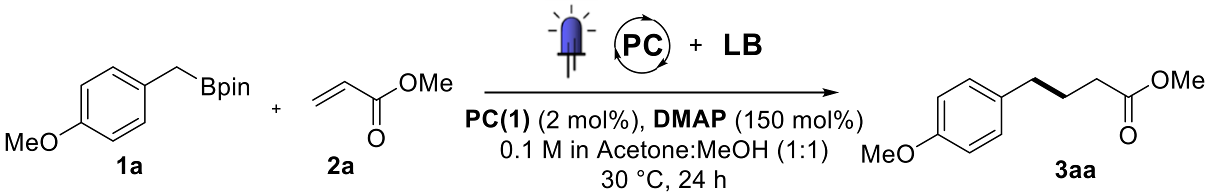

Entry	Photocatalyst	Solvent	Yield (%)[b]
1[c]	[Ir{dF(CF_3)ppy}$_2$(dtbpy)]PF_6	Acetone	50%(49%)
2	[Ir{dF(CF_3)ppy}$_2$(dtbpy)]PF_6	Acetone	54%
3	[Ir{dF(CF_3)ppy}$_2$(dtbpy)]PF_6	Acetone:MeOH (95:5)	66%
4	[Ir{dF(CF_3)ppy}$_2$(dtbpy)]PF_6	Acetone:MeOH (1:1)	**77%(74%)**
5	[Ir{dF(CF_3)ppy}$_2$(dtbpy)]PF_6	MeOH	74%(69%)
6	[Ir{dF(CF_3)ppy}$_2$(dtbpy)]PF_6	MeCN	62%
7	[Ir(dtbbpy)(ppy)$_2$][PF_6]	Acetone:MeOH (1:1)	71%
8	*fac*-Ir(ppy)$_3$	Acetone:MeOH (1:1)	0%
9	[Acr^+–Mes]ClO_4^-	Acetone:MeOH (1:1)	5%
10	[Acr^+–Mes]BF_4^-	Acetone:MeOH (1:1)	23%
11[d]	[Ir{dF(CF_3)ppy}$_2$(dtbpy)]PF_6	Acetone:MeOH (1:1)	75%

Entry	Change from best conditions (entry 3)	Yield (%)
12	no light	0%
13	no photocatalyst	0%
14	no degassing	0%

Optimization reaction conducted with 0.1 mmol of **1a**, 0.4 mmol of **2a**. [b]Yields in **3aa** determined by ^1H-NMR analysis of crude reaction mixture with CH_2Br_2 as an internal standard. Isolated yield in parentheses. Irradiation supplied by a commercial blue LED stripe (14.4 W at 450 nm).[c] 0.2 mmol of **2a**. [d]Reaction run for 36 hours.

2.2 Lewis Base Catalyst Screening

Lewis base catalyst screening protocol: A 5 mL microwave vial equipped with a magnetic stir bar was charged with 2-(4-methoxybenzyl)-4,4,5,5-tetramethyl-1,3,2-dioxaborolane (**1a,** 25 mg, 0.10 mmol), the photoredox catalyst Ir(dF(CF_3)ppy_2)(dtbpy)]PF_6 (2.2 mg, 2.0 mol%) and the Lewis base catalyst (50–20 mol%). The vial was then capped, evacuated and backfilled with argon three times. Methyl acrylate (**2a**, 36 µL, 0.40 mmol, 4.0 equiv.) was then added followed by 1.0 mL of a degassed acetone/methanol (1:1) mixture to lead a clear yellow transparent 0.1 M solution. This solution was then stirred while irradiated with a commercial blue LED strip (14.4 W, 450 nm) for 24 hours, the temperature was maintained at 30°C using a clip fan. After 24 h of irradiation the mixture was concentrated and dried *in vacuo*. Dibromomethane (10 µL, exact mass measured on balance) was added to the dry crude mixture as internal standard to determine the NMR yield in **3aa**.

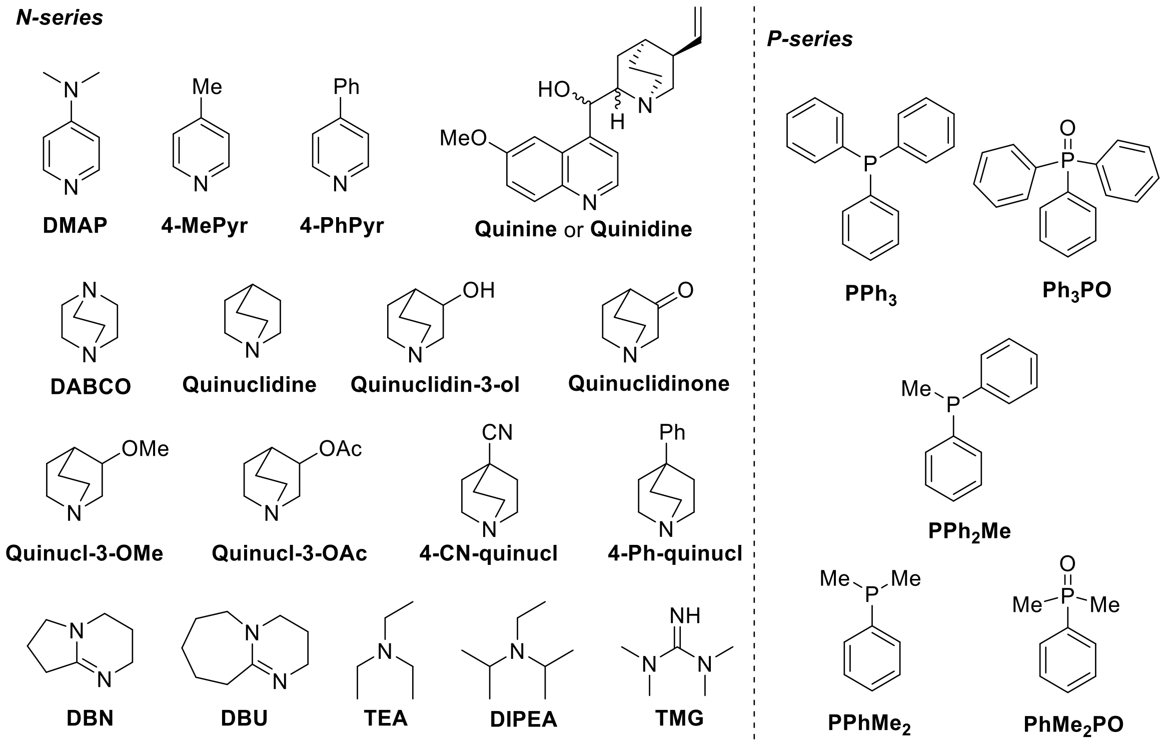

Figure S2 – Lewis base catalysts (**LB**) investigated

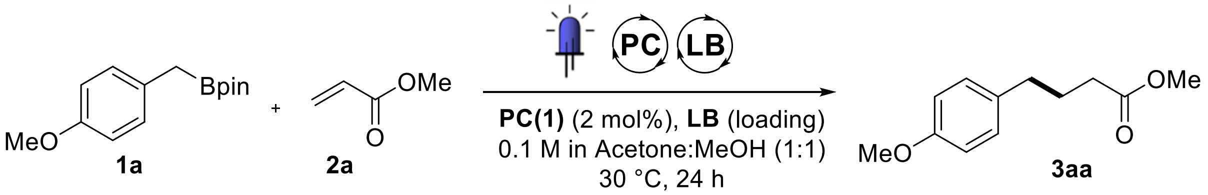

Entry	Lewis Base (LB)	Loading	Yield (%)[b]
1	DMAP	150 mol%	86%
**2**	**DMAP**	**20 mol%**	**75%**
3	4-MePyr	20 mol%	7%
4	4-PhPyr	20 mol%	12%
5	Quinine	20 mol%	65%
6	Quinidine	20 mol%	53%
7	DABCO	50 mol%	80%
8	DABCO	30 mol%	72%
9	DABCO	20 mol%	65%
**10**	**Quinuclidine**	**20 mol%**	**77%**
**11**	**Quinuclidin-3-ol**	**20 mol%**	**80% (75%)**
12	Quinuclidinone	20 mol%	43%
13	Quinucl-3-OMe	20 mol%	7%
14	Quinucl-3-OAc	20 mol%	65%
15	4-CN-quinucl	20 mol%	42%
16	4-Ph-quinucl	20 mol%	73%
17	DBN	50 mol%	54%
18	DBU	50 mol%	16%
19	TEA	50 mol%	44%
20	DIPEA	50 mol%	22%
21	TMG	50 mol%	0%
22	PPh_3	50 mol%	87%
23	PPh_3	40 mol%	83%
24	PPh_3	30 mol%	77%
25	**PPh_3**	**20 mol%**	**75%**
26	Ph_3PO	20 mol%	0%
27	PPh_2Me	20 mol%	53%
28	$PPhMe_2$	20 mol%	80%
29	$PhMe_2PO$	20 mol%	0%

Optimization reaction conducted with 0.1 mmol of **1a**, 0.4 mmol of **2a**. [b]Yields in **3aa** determined by ^1H-NMR analysis of crude reaction mixture with CH_2Br_2 as an internal standard. Isolated yield in parentheses. Irradiation supplied by a commercial blue LED stripe (14.4 W at 450 nm).

2.3 *Final optimisation for 1a*

Standard conditions protocol: A 5 mL microwave vial equipped with a magnetic stir bar was charged with 2-(4-methoxybenzyl)-4,4,5,5-tetramethyl-1,3,2-dioxaborolane (**1a,** 25 mg, 0.10 mmol), the photoredox catalyst Ir(dF(CF$_3$)ppy$_2$)(dtbpy)]PF$_6$ (2.2 mg, 2.0 mol%) and the quinuclidin-3-ol (2.5 mg, 0.02 mmol, 20 mol%). The vial was then capped, evacuated and backfilled with argon three times. Methyl acrylate (**2a**, 36 µL, 0.40 mmol, 4.0 equiv.) was then added followed by 1.0 mL of a degassed acetone/methanol (1:1) mixture to lead a clear yellow transparent 0.1 M solution. This solution was then stirred while irradiated with a commercial blue LED strip (14.4 W, 450 nm) for 24 hours, the temperature was maintained at 30°C using a clip fan. After 24 h of irradiation the mixture was concentrated and dried *in vacuo*. Dibromomethane (10 µL, exact mass measured on balance) was added to the dry crude mixture as internal standard to determine the NMR yield in **3aa**.

Standard Conditions (SC):

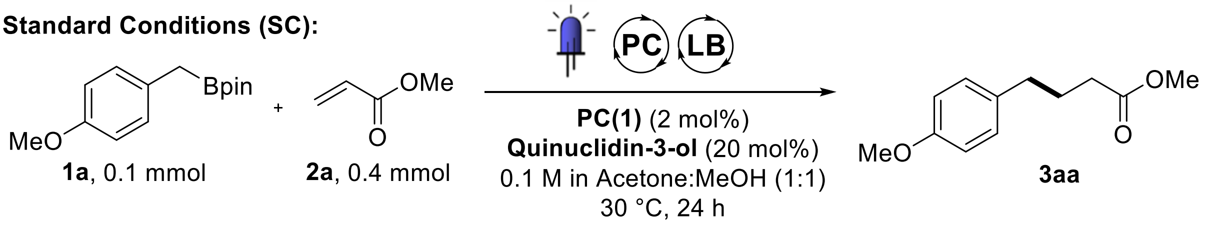

Entry	Variation from Standard Conditions (SC)	Yield (%)[b]
**1**	**none**	**80% (75%)**
2	3 mol% of **PC(1)** = [Ir{dF(CF$_3$)ppy}$_2$(dtbpy)]PF$_6$	83%
3	PC = **PC(2)** = [Ir{dF(CF$_3$)ppy}$_2$(bpy)]PF$_6$	79%
4	Acetone as solvent	8% (20% conv.)
5	Acetone:MeOH (9:1) as solvent	42% (54% conv.)
6	non-degased	40%
7	0.2 mmol of **2a**	65%
8	reaction time = 6 h	73% (92% conv.)
9	no photocatalyst	0%
10	no light	0%
11	no Lewis base	0%

Optimization reaction conducted with 0.1 mmol of **1a**, 0.4 mmol of **2a**. [b]Yields in **3aa** determined by ^{1}H-NMR analysis of crude reaction mixture with CH_2Br_2 as an internal standard. Isolated yield in parentheses. Irradiation supplied by a commercial blue LED stripe (14.4 W at 450 nm).

2.4 Aryl boronic species comparison

Standard conditions protocol: A 5 mL microwave vial equipped with a magnetic stir bar was charged with the desired aryl boronic acid derivative (0.10 mmol), the photoredox catalyst Ir(dF(CF$_3$)ppy$_2$)(dtbpy)]PF$_6$ (2.2 mg, 2.0 mol%) and the quinuclidin-3-ol (2.5 mg, 0.02 mmol, 20 mol%). The vial was then capped, evacuated and backfilled with argon three times. Methyl acrylate (**2a**, 36 µL, 0.40 mmol, 4.0 equiv.) was then added followed by 1.0 mL of a degassed acetone/methanol (1:1) mixture to lead a clear yellow transparent 0.1 M solution. This solution was then stirred while irradiated with a commercial blue LED strip (14.4 W, 450 nm) for 24 hours, the temperature was maintained at 30°C using a clip fan. After 17 h of irradiation the mixture was concentrated and dried *in vacuo*. Dibromomethane (10 µL, exact mass measured on balance) was added to the dry crude mixture as internal standard to determine the NMR yield in **5aa**.

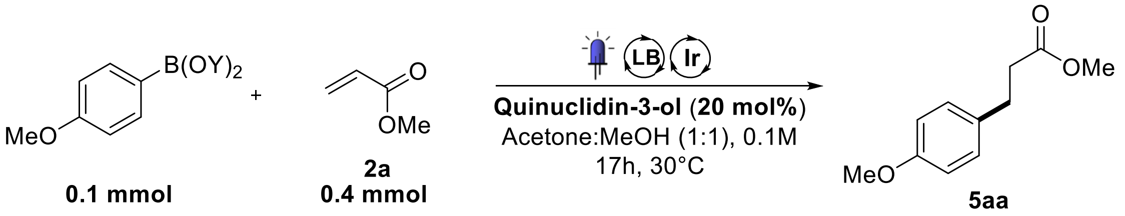

Aryl boronic acid derivative used

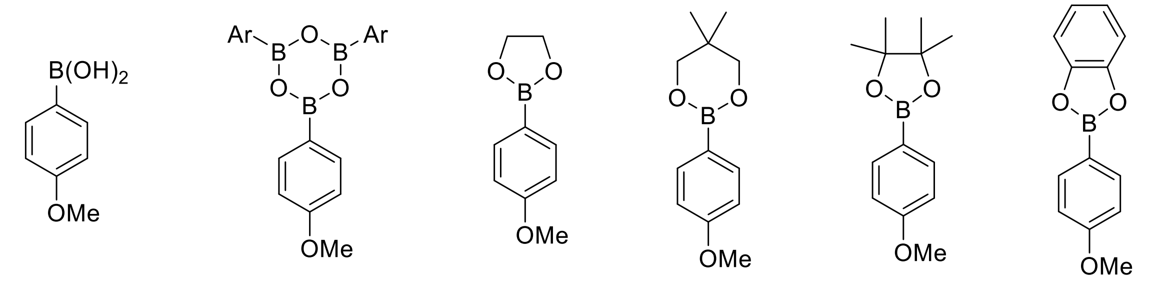

**conv** = 92%	**conv** = 92%	**conv** = 63%	**conv** = 44%	**conv** = 31%	**conv** = 32%
**yield** = 47%	**yield** = 47%	**yield** = 29%	**yield** = 24%	**yield** = 18%	**yield** = 0%

From this study, we rationalized that boronic acids and boroxines were identical in reactivity and superior to boronic esters. As we will prove in the next section (3.1.2), starting from the boronic acid or boroxine is not affecting the outcome of the reaction since there will be a fast-dynamic equilibrium between boronic acid and boroxine happening in reaction solvent mixture once the Lewis base added. It can be noted that aryl boronic esters could be used as well, albeit giving a slower reaction. Tt was therefore decided to employ more reactive and commercially available aryl boronic acids as starting materials.

3 Mechanistic experiments

3.1 Complexation experiments

3.1.1 Boronic ester complexation

When studying the ^{1}H-NMR spectrum of the mixture **2a** and DMAP in acetone-d^{6} we could observe that gradually increasing the total concentration of DMAP ($[DMAP]_t$) in the NMR tube had a shielding effect on the benzylic CH_2 signal (see protocol[1] and **Graph S1**). This is a proof of a fast dynamic complex formation where weighted average signals of the complex **1a-DMAP** and independent **1a** and DMAP signals are observed.[1] We also tried to separate signals at a lower temperature (at 228 K and 213 K) but signals were still fully averaged at this temperature meaning that the equilibrium is faster than NMR measurement timescale even at these low temperatures.

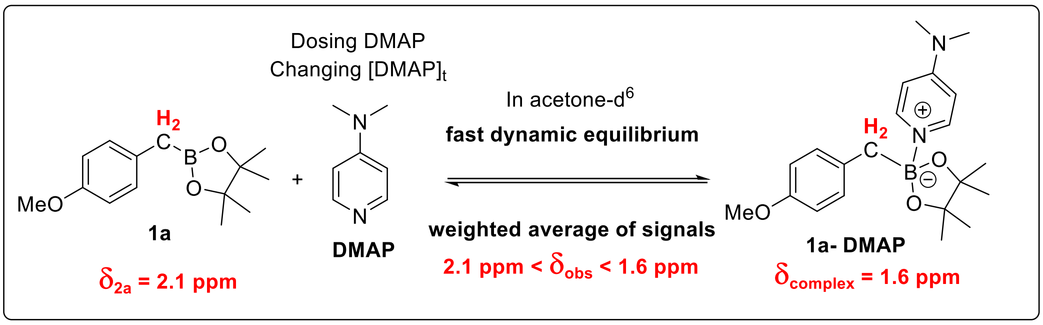

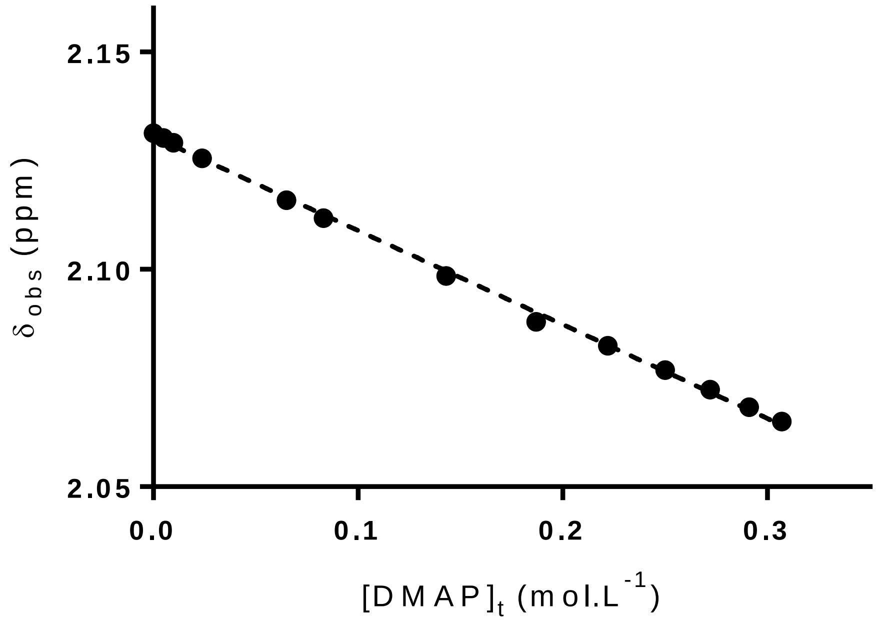

Graph 1 – Chemical shift of benzylic CH_2 of **2a** at different DMAP concentrations

Following the protocol described by Espenson[1] we were able to extrapolate the equilibrium constant ($K_{eq} = 0.8$) and the chemical shift of the complex ($\delta_{complex} = 1.6$ ppm).

3.1.2 *Boroxine vs. boronic acid complexation*

Complexation experiments on aryl boronic acids showed a much more dramatic effect than the boronic ester **1a**. Initial ^{1}H-NMR measurement (**Figure S3**) of commercial 4-methoxyphenyl boronic acid (**4a**) in reaction solvent mixture revealed the presence of two species in solution, **4a** and the corresponding trimeric boroxine **4a′**. The two distinct set of signals were observed for **4a** and **4a′** are a proof of a slow equilibrium (relative to NMR measurement time-scale) between the two species in acetone:methanol (1:1).

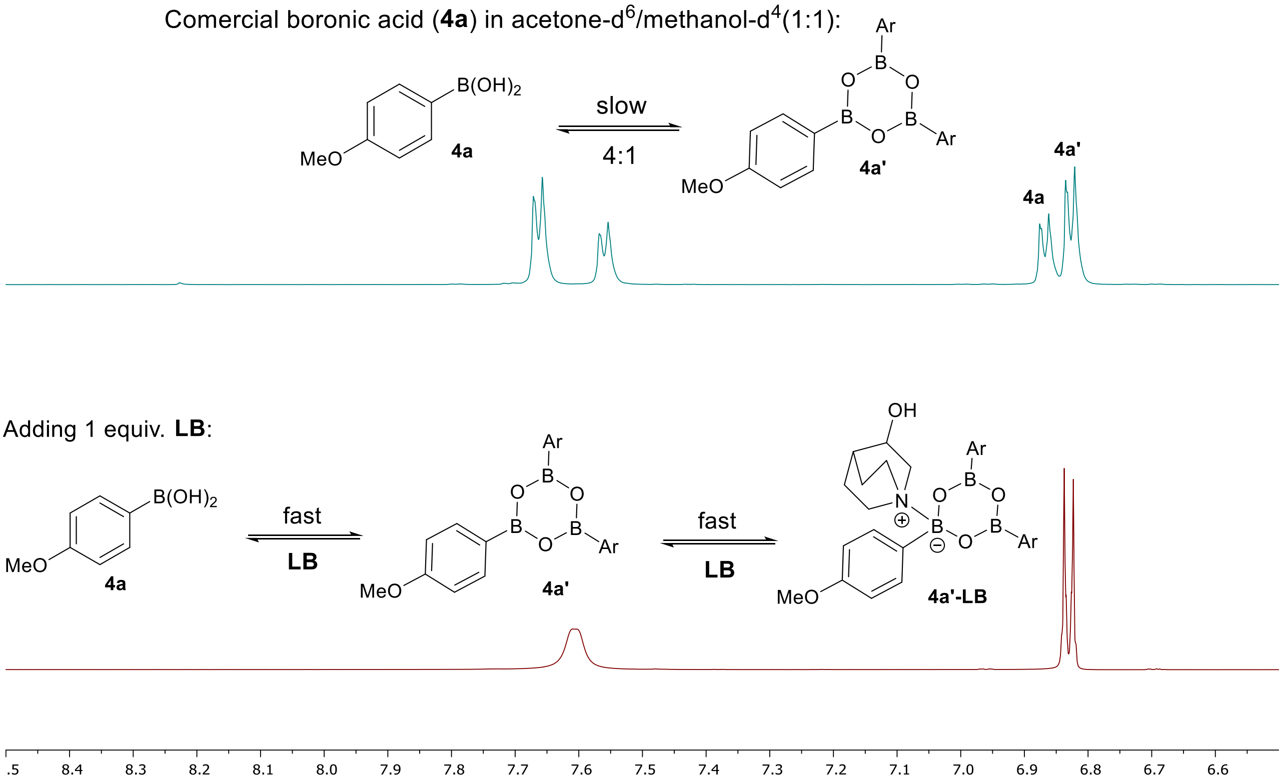

Figure S3 – 4a in Acetone-d^{6}/Methanol-d^{4} (1:1) with and without quinuclidin-3-ol = **LB**

When adding 1 equiv. of quinuclidin-3-ol (**LB**), all peaks merged into a single set of signals, meaning a fast equilibrium between **4a**, **4a′** and **4a′-LB** and potentially the **4a-LB** complex (not shown). From the literature it seemed that the boroxine[2,3] complex was more favoured than the complex with monomeric boronic acid **4a**. To check this hypothesis, we developed the following experiments where we could observe equilibrium between the species and the **LB** but no fast equilibrium between **4a** and **4a′**.

To deconvolute the equilibrium between **4a** and **4a′** from the ones involving the Lewis base we ran the same experiment in acetone-d^{6} as sole solvent. The absence of protic solvent was found to suppress the fast equilibrium between **4a** and **4a′** in the presence of **LB**. In this initial measurement (**Figure S4,** blue line), the slow equilibrium between **4a** and **4a′** is still observed (albeit being more shifted on the monomeric boronic acid side).

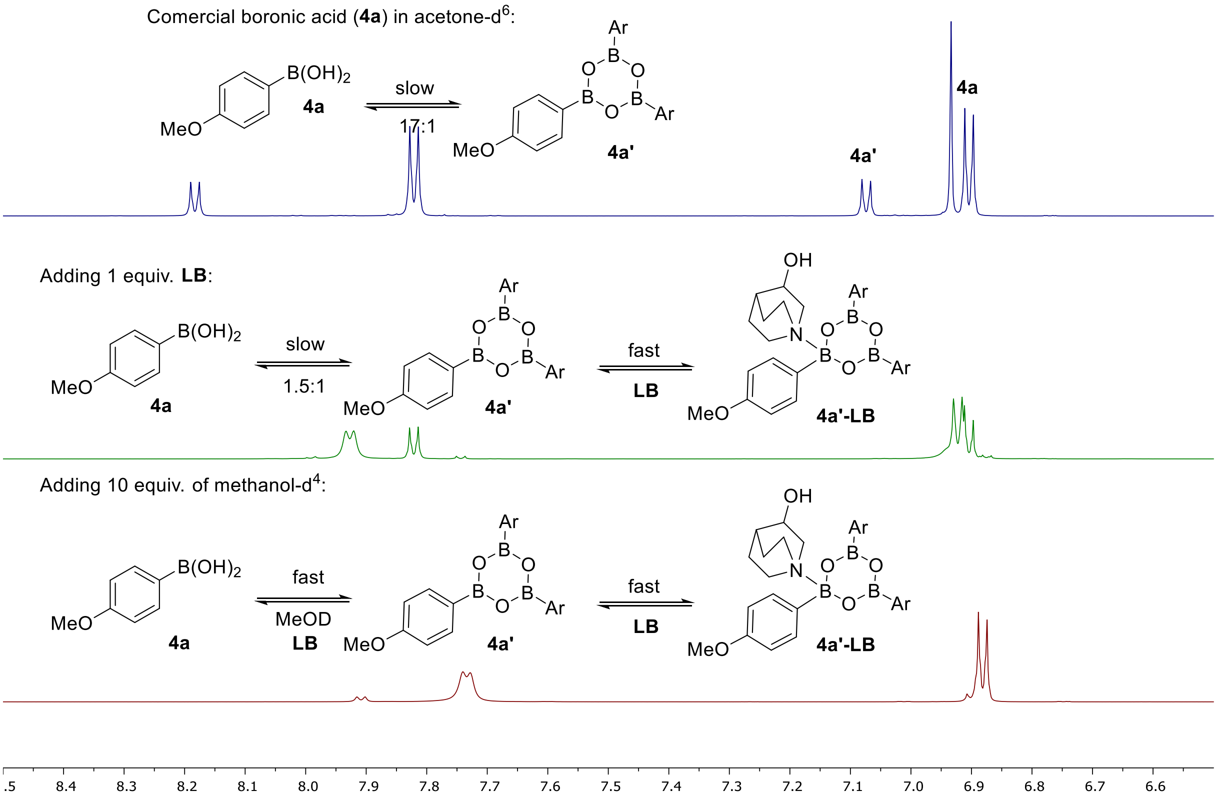

Figure S4 – from top to bottom, **4a** in acetone-d^{6}, addition of quinuclidin-3-ol = **LB** then methanol-d^{4}.

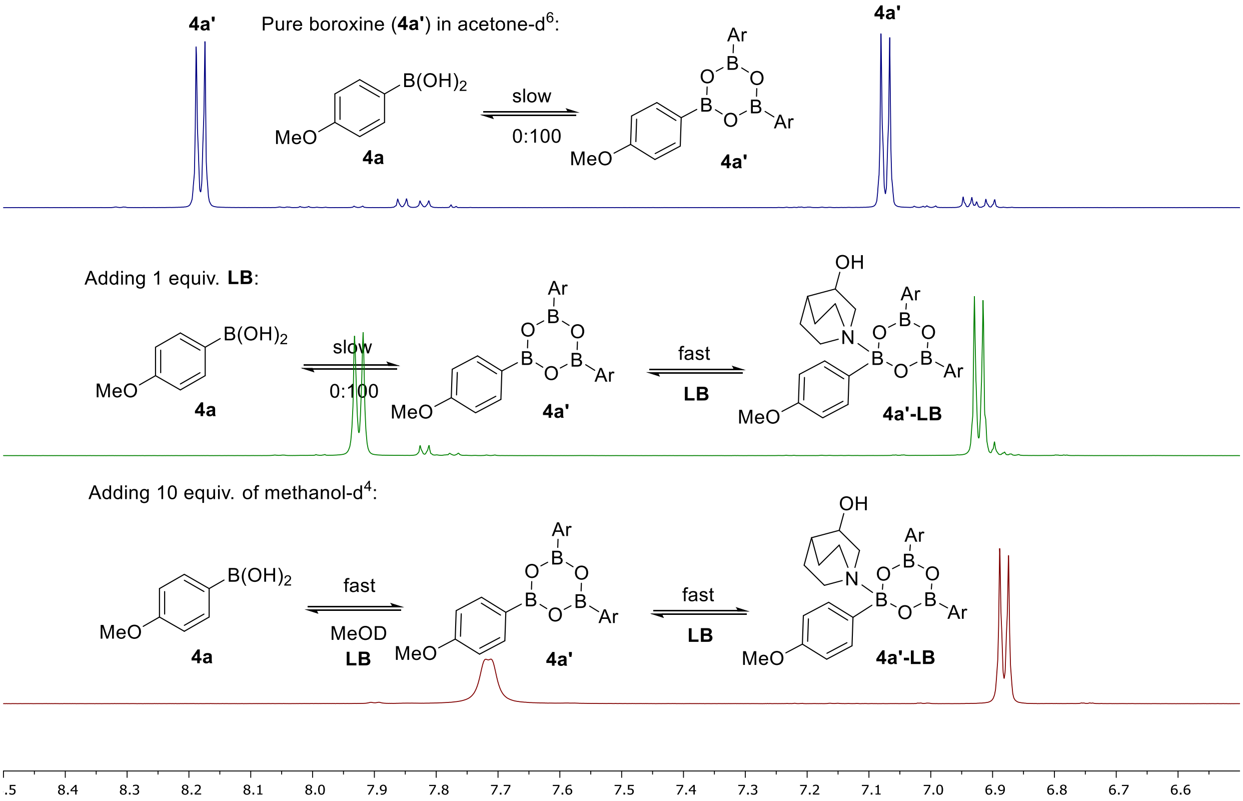

Figure S5 - from top to bottom, **4a′** in acetone-d^{6}, addition of quinuclidin-3-ol = **LB** then methanol-d^{4}.

Addition of 1 equiv. of **LB** (**Figure S4**, green line) only affected the peaks of **4a′** (in fast equilibrium with the **4a′-LB** complex) and left the **4a** peaks unshifted (despite a change in the proportions of the equilibrium). This confirmed us that no complex between LB and the boronic acid (**4a-LB)** was not forming. Addition of 10 equiv. of methanol-d^4 resulted in the same state than red line in **Figure S3**, where all the species are in fast equilibrium.

As a control, we performed the same experiment using the pure boroxine (**4a′**) prepared by azeotropic removal of water from **4a** (**Figure S5**). In acetone-d^6 only boroxine peaks were observed (blue line, **Figure S5**). Addition of the **LB** led again to a change in the shift with the formation of **4a′-LB** (green line). Addition of 10 equiv. of methanol-d^4 resulted in the same state than red line in **Figure S3** and **Figure S4**, where all the species are in fast equilibrium.

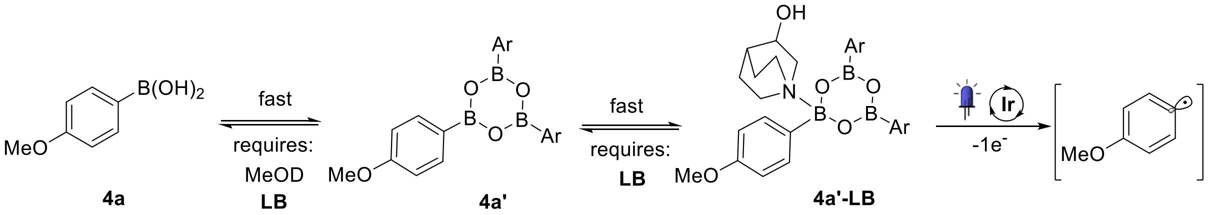

Scheme S1 – Summary of postulated boronic acid reactivity in reaction solvent

These experiments thought us that in the case of boronic acid starting materials (**4a**), complex formation with **LB** is preferred from the most Lewis acidic trimeric boroxine species (**4a′**). We also observed that methanol acts as a proton source to enable a fast, dynamic equilibrium between **4a** and **4a′** when **LB** is present (in addition as being required for the recycling of the **LB** in catalytic experiments). These finding explains why starting from a "pure" boroxine starting materials gives the same results as the boronic acid (see p. S9) in our reaction conditions where both methanol and **LB** are present. We therefore postulate that as in the case of the boronic esters this **4a′-LB** complex will be the one single electron oxidized by the excited photoredox catalyst (e.g. the one with the lowest $E_{1/2}$ value).

3.2 *Electrochemical measurements*

Cyclic voltammetry measurements were performed to study the effect of the Lewis base complexation on the oxidation potential of the boronic pinacol ester. Based on previous observations from NMR complexation experiments (see p.S10) and DFT calculations previously performed,[4] we assumed that the oxidation potential of the boronic ester **1a** is significantly reduced by the coordination of DMAP.

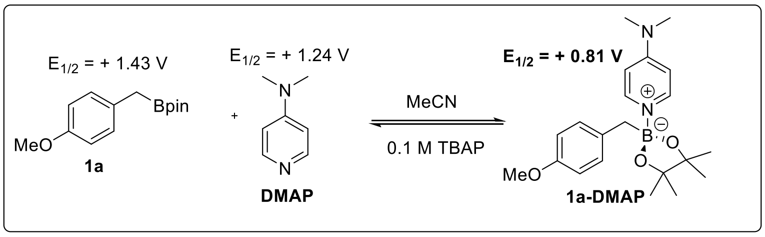

Protocol: Experiments were conducted on a Palmstat EmStat 3 potentiostat with a glassy carbon working, a platinum mesh counter and a Ag/AgCl reference electrode, referenced to SCE using ferrocene (Fc) as an internal standard (0.42 V vs. SCE).[5] In the standard procedure 0.02 mmol substrate were dissolved in 10 mL of a 0.1 M $[N(Bu)_4]PF_6$ electrolyte solution in degassed MeCN. The reactor was sealed with a rubber septum and degassed by purging with nitrogen for 10 min. Each measurement was conducted with a new glassy carbon working electrode and a scan rate of 100 mV/s at room temperature under nitrogen atmosphere without stirring. The half-peak oxidation potentials ($E_{p/2}$) were determined as the voltage at half the current of the local maximum current (C_{max}) using QTI-Plot (see Eq. 1).[5]

$$f\left(E_{\frac{p}{2}}\right) = \frac{C_{\max}}{2} \quad (1)$$

Due to the generation of radicals during oxidation of the analytes, which undergo further reactions, all cyclic voltammograms showed an asymmetric shape describing the irreversibility of the oxidation. Deviations of the oxidation waves from the baseline (DMAP and **1a** measurements) were justified as an effect of the electrolyte solution.

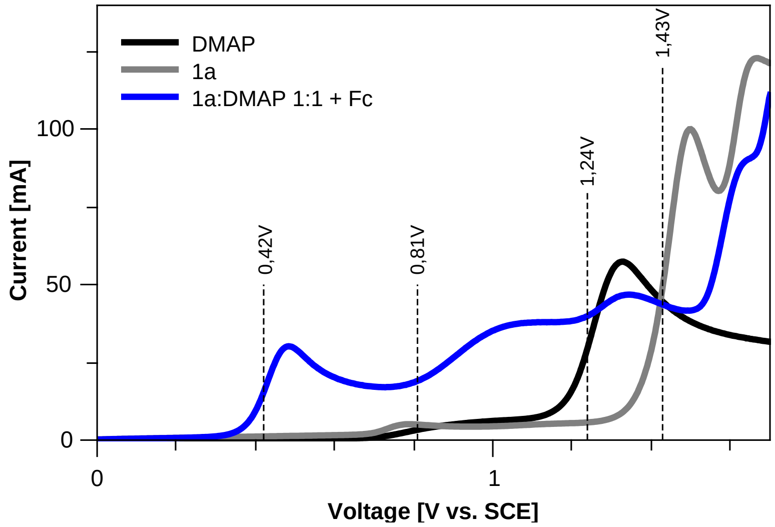

Figure S6 Oxidation waves of the cyclovoltagrams with boronic ester **1a** and DMAP.

At first, the redox potentials of DMAP ($E_{p/2}$ = 1.24 V vs. SCE) and the model boronic ester **1a** ($E_{p/2}$ = 1.43 V vs. SCE) were quantified. Whereas the first maximum in the measurement with the ester **1a** corresponds to the formation of the benzylic radical, the second maximum is assumed to refer to its oxidation to the benzylic cation.[6]

Then a 1:1 mixture of DMAP and the boronic ester **1a** was engaged under addition of a small amount of ferrocene (Fc) as an internal reference (0.42 V vs. SCE). Besides the oxidations of ferrocene and residual DMAP, there is a new local maximum visible at around 1 V, which indicates the oxidation of the **1a-DMAP** zwitterionic complex in the mixture. The measured potential ($E_{p/2}$) for this maximum is 0.81 V vs. SCE and corresponds the previously calculated value for a similar complex in acetone ($E_{1/2}$ = 0.73 V vs. SCE)[4]. The fact, that the maximum referring to the oxidation of the boronic ester **1a** is significantly downscaled, and the appearance of the new maximum match our observation from the NMR complexation experiments. The obtained oxidation potentials prove the boronic ester **1a** to be activated by Lewis base complexation. The excited state of the photocatalyst ($E_{1/2}$ = 1.21 V vs. SCE)[7] is therefore preferably quenched by the activated DMAP complex of 1a ($E_{p/2}$ = 0.81 V vs. SCE).

4 Synthesis and characterisation of starting materials

4.1 Synthesis of alkene starting materials

2-(1-phenylvinyl) pyridine (2o)

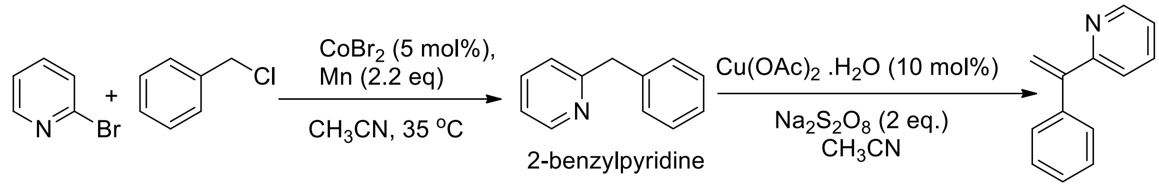

2o

2-benzylpyridine was prepared according to a reported method.[8] Thus obtained 2-benzylpyridine (0.56 mmol, 95 mg) was taken in a 20 mL two-necked flask equipped with a reflux condenser along with $Cu(OAc)_2{\cdot}H_2O$ (0.056 mmol, 11 mg), $Na_2S_2O_8$ (1.12 mmol, 266 mg) and DMA (2.5 mL). The resulting mixture was then stirred under argon at 120 °C for 4 h. The reaction mixture was then cooled down to room temperature and extracted with ethyl acetate. The organic layer was washed with water (3 x 100 mL), dried over Na_2SO_4 and solvents removed *in vacuo*. Column chromatography on silica gel (15% EtOAc in hexane) afforded product **2o** (65 g, 0.35mmol) as a colourless oil in 64.3% yield.[9]

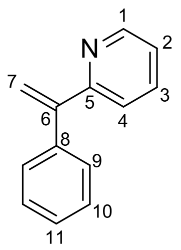

^{1}H-NMR (400 MHz, $CDCl_3$) δ 8.64 (d, J = 4.8 Hz, 1H, H_1), 7.63 (t, J = 7.7 Hz, 1H, H_3), 7.35 (s, 5H, H_8, H_9, H_{10} and H_{11}), 7.27 (d, J = 8.1 Hz, 1H, H_4), 7.20 (t, J = 6.3 Hz, 1H, H_2), 5.99 (s, 1H, H_{7a}), 5.61 (s, 1H, H_{7b}).**^{13}C-NMR (151 MHz, $CDCl_3$)** 158.5, 149.4, 149.2, 140.4, 136.3, 128.4, 128.3, 127.8, 122.8, 122.4, 117.7. **HRMS for $[C_{13}H_{12}N]^+$** calc. 182.0970 found 182.0966. ***R*f** (1:4 EtOAc/PE) = 0.58. Spectroscopic data were consistent with literature values.[9]

4.2 *Synthesis of boronate starting materials*

***tert*-butyl dimethylcarbamate**

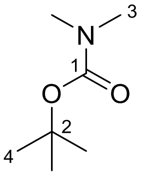

A solution of triethylamine (3.0 mL, 22 mmol, 1.0 equiv.) and dimethylamine (40% in H_2O, 2.7 mL, 22 mmol, 1.0 equiv.) in THF (10 mL) was cooled in an ice bath. Boc_2O (5.0 mL, 22 mmol, 1.0 equiv.) was added slowly and the reaction mixture was stirred at 0°C for 10 min. The ice bath was removed and the mixture stirred for additional 18 h at room temperature. The solvent was carefully removed under reduced pressure and the residue was suspended in water (15 mL). The suspension was extracted with EtOAc (3 x 20 mL), the combined organic layers were dried ($MgSO_4$) and concentrated *in vacuo*. Fractional distillation under reduced pressure afforded *tert*-butyl dimethylcarbamate (3.1 g, 22 mmol) as a colourless oil in 99% yield.

^{1}H-NMR (600 MHz, $CDCl_3$) δ 2.85 (s, 6H, H_3), 1.45 (s, 9H, H_4). **^{13}C-NMR (151 MHz, $CDCl_3$)** δ 156.2 (C_1), 79.3 (C_2), 36.3 (C_3), 28.6 (C_4). **HRMS for $[C_7H_{15}O_2NNa]^+$** calc.168.0995 found 168.0991. **B.p** (12 mTorr) = 40°C. Spectroscopic data were consistent with literature values.[10]

***tert*-butyl methyl((4,4,5,5-tetramethyl-1,3,2-dioxaborolan-2-yl)methyl)carbamate (1h)**

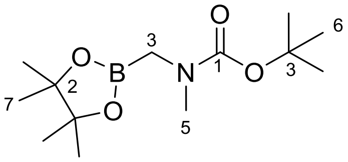

A solution of *tert*-butyl dimethylcarbamate (0.20 g, 1.4 mmol, 1.0 equiv.) and TMEDA (0.40 mL, 2.8 mmol, 2.0 equiv.) in Et_2O (10 mL) was cooled to -78°C. *s*-BuLi (1.4 M in hexane, 1.2 mL, 1.7 mmol, 1.2 equiv.) was added dropwise and the mixture was stirred at -78°C for 3 h. Then a solution of 2-isopropoxy-4,4,5,5-tetramethyl-1,3,2-dioxaborolane (0.34 mL, 1.7 mmol, 1.2 equiv.) in Et_2O (2 mL) was added. After stirring at -78°C for 1.5 h acetyl chloride (0.12 mL, 1.7 mmol, 1.2 equiv.) was added and the reaction mixture was allowed to warm to room temperature. The reaction mixture was concentrated under reduced pressure and the residue was suspended in 0.1 M PBS (pH 7, 30 mL) and extracted with EtOAc (3 x 40 mL). The combined organic layers were dried ($MgSO_4$) and the solvent was removed *in vacuo.* Column chromatography on oven-dried silica (20% to 25% EtOAc in hexane) afforded the product (0.34 g, 1.2 mmol) as a yellowish oil in 90% yield.

^{1}H-NMR (600 MHz, $CDCl_3$) δ 2.77 (s, 3H, H_5), 2.34 (s, 2H, H_4), 1.43 (s, 9H, H_6), 1.14 (s, 12H, H_7) ppm. **^{13}C-NMR (151 MHz, $CDCl_3$)** δ 160.7 (C_1), 84.7 (C_2), 80.7 (C_3), 40.7 (C_4), 34.2 (C_5), 28.4 (C_6), 25.1 (C_7). **^{11}B-NMR (193 MHz, $CDCl_3$)** δ 17.2. **IR (ATR – neat)** $\tilde{v}$ (cm^{-1}) =

2976, 1689, 1367, 1335, 1215, 1160, 1139, 968, 879, 846, 771. **HRMS for [$C_{13}H_{27}O_4NB$]$^+$** calc. 272.2028 found 272.2026. **R*f*** (1:1 EtOAc/PE) = 0.35.

***tert*-butyl ((4,4,5,5-tetramethyl-1,3,2-dioxaborolan-2-yl)methyl)carbamate (1g)**

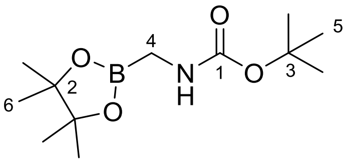

A solution of NaHMDS (1 M in THF, 5.6 mL, 5.6 mmol, 1.0 equiv.) in THF (30 mL) was cooled to -78°C. 2-(chloromethyl)-4,4,5,5-tetramethyl-1,3,2-dioxaborolane (0.98 g, 5.6 mmol, 1.0 equiv.) in THF (6 mL) was added dropwise and the reaction mixture was stirred at -78°C for 20 min, before it was allowed to warm to room temperature and stirred for additional 2h. The reaction mixture was cooled in an ice bath, MeOH (0.44 mL, 11 mmol, 2.0 equiv.) was added and the mixture was stirred at 0°C for 1h. The ice bath was removed, Boc_2O (1.5 mL, 6.7 mmol, 1.2 equiv.) was added and the mixture was stirred at room temperature for 72 h. The solvent was evaporated and the crude mixture was loaded on oven dried silica. Column chromatography on oven-dried silica (20% Et_2O in PE) afforded the product (1.1 g, 4.3 mmol) as a colourless oil in 77% yield.

^{1}H-NMR (600 MHz, $CDCl_3$) δ 4.63 (s, 1H, *NH*), 2.77 (d, *J* = 4.4 Hz, 2H, H_4), 1.44 (s, 9H, H_5), 1.27 (s, 12H, H_6). **^{13}C-NMR (151 MHz, $CDCl_3$)** δ 157.1 (C_1), 84.2 (C_2), 79.2 (C_3), 28.6 (C_5), 24.9 (C_6). **^{11}B-NMR (193 MHz, $CDCl_3$)** δ 32.6. **IR (ATR – neat)** $\tilde{\nu}$ (cm^{-1}) = 3376, 2979, 1697, 1506, 1382, 1367, 1335, 1167, 1141, 968, 845, 780. **HRMS for [$C_{12}H_{24}O_4NBNa$]$^+$** calc. 280.1691 found 280.1686. **R*f*** (1:1 EtOAc/PE) = 0.28.

5 Synthesis and characterisation of coupling products

5.1 Scope of electron-deficient olefins

5.1.1 General procedure I

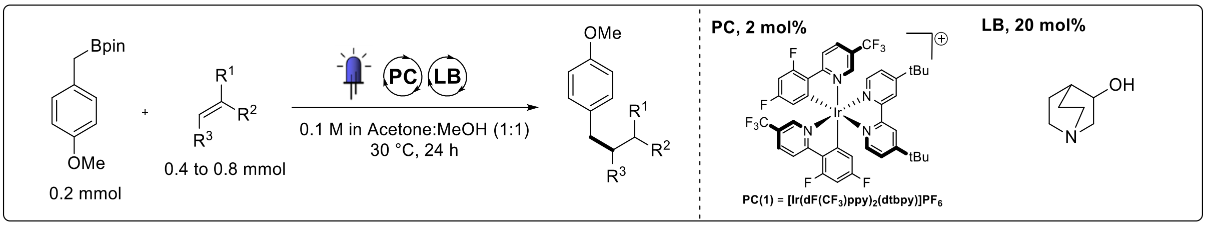

General Procedure I - GP (I): A 5 mL glass vial equipped with a magnetic stir bar was charged with 2-(4-methoxybenzyl)-4,4,5,5-tetramethyl-1,3,2-dioxaborolane (**1a**, 50 mg, 0.20 mmol), the photoredox catalyst Ir(dF(CF_3)ppy_2)(dtbpy)]PF_6 (4.4 mg, 2 mol%) and quinuclidin-3-ol (5 mg, 0.04 mmol, 20 mol%). The vial was then sealed with a rubber septum and evacuated/backfilled with argon three times. The volatile olefin (0.4–0.8 mmol, 2.0–4.0 equiv.) was then added followed by 2.0 mL of a degassed acetone/methanol (1:1) mixture to lead a clear yellow transparent 0.1 M solution. This solution was then stirred while irradiated with a commercial blue LED strip (14.4 W, 450 nm) for 24 hours, the temperature was maintained at 30°C using a desktop fan. The content of the vial was then concentrated and immobilized on Isolute HM-N for easy dry loading on flash column chromatography to yield the pure product.

5.1.2 Characterization of coupling products

Methyl 4-(4-methoxyphenyl)butanoate (3aa)

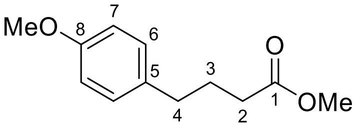

Obtained following **GP (I)** at 0.2 mmol scale using 0.8 mmol of methyl acrylate. Purification by column chromatography on silica gel (3% EtOAc in hexane) afforded product **3aa** (31 mg, 0.15 mmol) as colourless oil in 75% yield.**^{1}H-NMR (600 MHz, $CDCl_3$)** δ 7.09 (d, J = 8.6 Hz, 2H, H_6), 6.83 (d, J = 8.6 Hz, 2H, H_7), 3.79 (s, 3H, ArO*Me*), 3.66 (s, 3H, COO*Me*), 2.61 – 2.58 (m, 2H, H_2), 2.32 (t, J = 7.5 Hz, 2H, H_4), 1.95 – 1.89 (m, 2H, H_3). **^{13}C-NMR (151 MHz, $CDCl_3$)** δ 174.0 (C_1), 157.9 (C_8), 133.4 (C_5), 129.4 (C_6), 113.8 (C_7), 55.2 (ArO*Me*), 51.5 (COO*Me*), 34.2 (C_4), 33.3 (C_2), 26.7 (C_3). **HRMS for [$C_{12}H_{17}O_3$]$^+$** calc. 209.1172, found 209.1163. ***R*f** (1:4 EtOAc/PE) = 0.38. Spectroscopic data were consistent with literature values.[11]

***tert*-butyl 4-(4-methoxyphenyl)butanoate (3ab)**

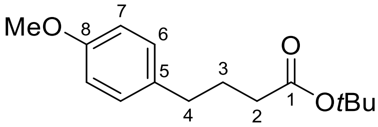

Obtained following **GP (I)** at 0.2 mmol scale using 0.8 mmol of *tert*-butyl acrylate. Purification by column chromatography on silica gel (7% EtOAc in hexane) afforded product **3ab** (24 mg, 0.096 mmol) as colourless oil in 48% yield. **^{1}H-NMR (300 MHz, $CDCl_3$)** δ 7.10 (d, *J* = 8.6 Hz, 2H, H_6), 6.83 (d, *J* = 8.6 Hz, 2H, H_7), 3.79 (s, 3H, O*Me*) 2.58 (t, *J* = 7.4 Hz, 2H, H_2), 2.22 (t, *J* = 7.5 Hz, 2H, H_4), 1.88 (q, *J* = 7.6 Hz, 2H, H_3), 1.44 (s, 9H, O*tBu*). **^{13}C-NMR (101 MHz, $CDCl_3$)** δ 173.0 (C_1), 157.8 (C_8), 133.7 (C_5), 129.4 (C_6), 113.8 (C_7), 80.1 (O*C*(Me)$_3$), 55.3 (O*Me*), 34.9 (C_4), 34.2 (C_2), 28.1 (OC(*Me*)$_3$), 27.0 (C_3). **HRMS for $[C_{15}H_{23}O_3Na]^+$** calc. 273.1464, found 273.1461. ***R*f** (1:4 EtOAc/PE) = 0.60. Spectroscopic data were consistent with literature values.[12]

Benzyl 4-(4-methoxyphenyl)butanoate (3ac)

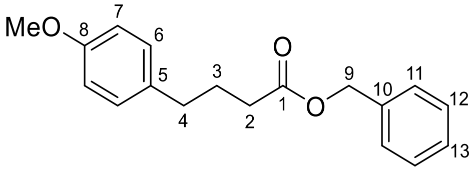

Obtained following **GP (I)** at 0.2 mmol scale using 0.8 mmol of benzyl acrylate. Purification by column chromatography on silica gel (7% EtOAc in hexane) afforded product **3ac** (27 mg, 0.10 mmol) as colourless oil in 47% yield. **^{1}H-NMR (400 MHz, $CDCl_3$)** δ 7.41 – 7.28 (m, 5H, H_{11}, $H_{12,}$ and H_{13}), 7.07 (d, *J* = 8.2 Hz, 2H, H_6), 6.81 (d, *J* = 8.2 Hz, 2H, H_7), 5.11 (s, 2H, H_9), 3.78 (s, 3H, O*Me*), 2.58 (t, *J* = 7.5 Hz, 2H, H_4), 2.36 (t, *J* = 7.5 Hz, 2H, H_2), 1.94 (p, *J* = 7.5 Hz, 2H, H_3). **^{13}C-NMR (101 MHz, $CDCl_3$)** δ 173.4 (C_1), 157.9 (C_8), 136.0 (C_{10}), 133.4 (C_5), 129.4 (C_6), 128.6 (C_{12}), 128.2 (C_{13}), 127.7 (C_{11}), 113.8 (C_7), 66.2 (C_9), 55.3 (O*Me*), 34.2 (C_4), 33.6 (C_2), 26.7 (C_3). **HRMS for $[C_{18}H_{21}O_3Na]^+$** calc. 308.1385, found 308.1389. ***R*f** (1:4 EtOAc/PE) = 0.82. Spectroscopic data were consistent with literature values.[13]

5-(4-methoxyphenyl)pentan-2-one (3ad)

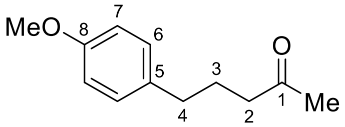

Obtained following **GP (I)** at 0.2 mmol scale using 0.8 mmol of methyl vinyl ketone. Purification by column chromatography on silica gel (10% EtOAc in hexane) afforded product **3ad** (32 mg, 0.16 mmol) as colourless oil in 82% yield. **^{1}H-NMR (300 MHz, $CDCl_3$)** δ 7.08 (d, *J* = 8.7 Hz, 2H, H_6), 6.83 (d, *J* = 8.7 Hz, 2H, H_7), 3.79 (s, 3H, O*Me*), 2.56 (t, *J* = 7.5 Hz, 2H, H_4), 2.42 (t, *J* = 7.5 Hz, 2H, H_2), 2.11 (s, 3H, CO*Me*), 1.88 (q, *J* = 7.5 Hz, 2H, H_3). **^{13}C-NMR (151 MHz, $CDCl_3$)** δ 208.9 (C_1), 157.9 (C_8), 133.6 (C_5), 129.4 (C_6), 113.8 (C_7), 55.3 (O*Me*), 42.8 (C_2), 34.1 (C_4), 30.0 (CO*Me*), 25.5 (C_3). **HRMS for $[C_{12}H_{17}O_2]^+$** calc. 193.1223, found 193.1218. ***R*f** (1:4 EtOAc/PE) = 0.46. Spectroscopic data were consistent with literature values.[11]

4-(4-methoxyphenyl)butanal (3ae)

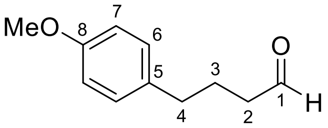

Obtained following **GP (I)** at 0.2 mmol scale using 0.4 mmol of acrolein. Purification by column chromatography on silica gel (10% EtOAc in hexane) afforded product **3ae** (22 mg, 0.16 mmol) as colourless oil in 63% yield. **^{1}H-NMR (400 MHz, $CDCl_3$)** δ 9.75 (s, 1H, C*H*O), 7.09 (d, *J* = 8.2 Hz, 2H, H_6), 6.83 (d, *J* = 8.2 Hz, 2H, H_7), 3.79 (s, 3H, O*Me*), 2.60 (t, *J* = 7.5 Hz, 2H, H_2), 2.44 (t, *J* = 7.5 Hz, 1H, H_4), 1.93 (p, *J* = 7.5 Hz, 2H, H_3). **^{13}C-NMR (101 MHz, $CDCl_3$)** δ 202.5 (C_1), 158.0 (C_8), 133.3 (C_5), 129.4 (C_6), 113.9 (C_7), 55.3 (O*Me*), 43.1 (C_2), 34.1 (C_4), 23.9 (C_3). **HRMS for $[C_{11}H_{15}O_2]^+$** calc. 179.1067, found 179.1060. ***R*f** (1:4 EtOAc/PE) = 0.30. Spectroscopic data were consistent with literature values.[14]

4-(4-methoxyphenyl)butanenitrile (3af)

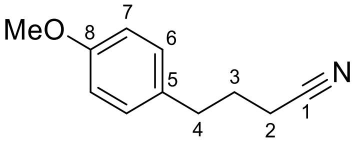

Obtained following **GP (I)** at 0.2 mmol scale using 0.8 mmol of acrylonitrile. Purification by column chromatography on silica gel (10% EtOAc in hexane) afforded product **3af** (27 mg, 0.15 mmol) as colourless oil in 77% yield. **^{1}H-NMR (400 MHz, $CDCl_3$)** δ 7.10 (d, *J* = 8.2 Hz, 2H, H_6), 6.85 (d, *J* = 8.2 Hz, 2H, H_7), 3.79 (s, 3H, O*Me*), 2.72 (t, *J* = 7.4 Hz, 2H, H_4), 2.30 (t, *J* = 7.1 Hz, 2H, H_2), 1.94 (p, *J* = 7.3 Hz, 2H, H_3). **^{13}C-NMR (101 MHz, $CDCl_3$)** δ 158.3 (C_8), 131.7 (C_5), 129.4 (C_6), 119.6 (C_1), 114.1 (C_7), 55.3 (O*Me*), 33.5 (C_4), 27.1 (C_3), 16.3 (C_2). **HRMS for $[C_{11}H_{14}NO]^+$** calc. 176.1070, found 176.1077. ***R*f** (1:4 EtOAc/PE) = 0.50. Spectroscopic data were consistent with literature values.[11]

1-methoxy-4-(3-(phenylsulfonyl)propyl)benzene (3ag)

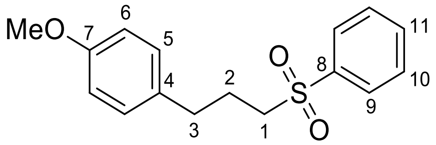

Obtained following **GP (I)** at 0.2 mmol scale using 0.4 mmol of acrylonitrile. Purification by column chromatography on silica gel (12% EtOAc in hexane) afforded product **3ag** (30 mg, 0.10 mmol) as white solid in 51% yield. **^{1}H-NMR (400 MHz, $CDCl_3$)** δ 7.88 (d, *J* = 7.6 Hz, 2H, H_9), 7.65 (t, *J* = 7.5 Hz, 1H, H_{11}), 7.56 (t, *J* = 7.6 Hz, 2H, H_{10}), 7.01 (d, *J* = 8.1 Hz, 2H, H_5), 6.80 (d, *J* = 8.1 Hz, 2H, H_6), 3.78 (s, 3H, O*Me*), δ 3.11 – 3.01 (m, 1H, H_1), 2.64 (t, *J* = 7.5 Hz, 2H, H_3), 2.01 (p, *J* = 7.6 Hz, 2H, H_2). **^{13}C-NMR (101 MHz, $CDCl_3$)** δ 158.2 (C_7), 139.1 (C_8), 133.6 (C_4), 131.8 (C_{11}), 129.3 (C_5), 129.3 (C_6), 128.0 (C_{19}), 114.0 (C_6), 55.4 (C_1), 55.3 (O*Me*), 33.2 (C_3), 24.4 (C_2). **HRMS for $[C_{16}H_{19}O_3S]^+$** calc. 291.1049, found 291.1044. ***R*f** (1:4 EtOAc/PE) = 0.25. **M p.** 60–62°C. Spectroscopic data were consistent with literature values.[9]

methyl 4-(4-methoxyphenyl)-2-methylbutanoate (3ah)

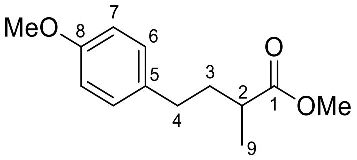

Obtained following **GP (I)** at 0.2 mmol scale using 0.8 mmol of methyl methacrylate. Purification by column chromatography on silica gel (10% EtOAc in hexanes) afforded product **3ah** (28 mg, 0.13 mmol) as colourless oil in 64% yield. **^{1}H-NMR (600 MHz, CDCl$_3$)** δ 7.10 (d, *J* = 8.5 Hz, 2H, H$_7$), 6.83 (d, *J* = 8.5 Hz, 2H, H$_6$), 3.79 (s, 3H, O*Me*), 3.68 (s, 3H, COO*Me*), 2.56 (t, *J* = 7.9 Hz, 2H, H$_4$), 2.48 (h, *J* = 7.0 Hz, 1H, H$_2$), 2.04 – 1.94 (m, 1H, H$_3$), 1.69 (dq, *J* = 14.2, 7.7 Hz, 1H, H$_3$), 1.19 (d, *J* = 7.0 Hz, 3H, H$_7$). **^{13}C-NMR (151 MHz, CDCl$_3$)** δ 177.0 (C$_1$), 157.8 (C$_8$), 133.7 (C$_5$), 129.3 (C$_6$), 113.8 (C$_7$), 55.2 (O*Me*), 51.5 (CO*Me*), 38.9 (C$_2$), 35.6 (C$_4$), 32.5 (C$_3$), 17.1 (C$_9$). **HRMS for [C$_{13}$H$_{19}$O$_3$]$^+$** calc. 223.1329, found 223.1328. ***R*f** (1:4 EtOAc/PE) = 0.31. Spectroscopic data were consistent with literature values.[11]

3-(4-methoxyphenethyl) dihydrofuran-2(3*H*)-one (3ai)

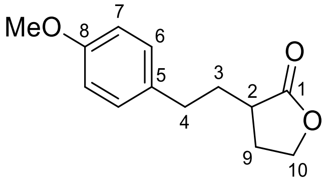

Obtained following **GP (I)** at 0.2 mmol scale using 0.8 mmol of 3-methylenedihydrofuran-2(3*H*)-one. Purification by column chromatography on silica gel (10% EtOAc in hexanes) afforded product **3ai** (28 mg, 0.13 mmol) as colourless oil in 68% yield. **^{1}H-NMR (600 MHz, CDCl$_3$)** δ 7.11 (d, *J* = 8.6 Hz, 2H, H$_7$), 6.83 (d, *J* = 8.6 Hz, 2H, H$_6$), 4.33 (td, *J* = 8.8, 2.8 Hz, 1H, H$_{10a}$), 4.15 (td, *J* = 9.4, 6.7 Hz, 1H, H$_{10b}$), 3.78 (s, 3H, O*Me*), 2.70-2.77 (m, 1H, H$_{4a}$), 2.69 – 2.61 (m, 1H, H$_{4b}$), 2.53 – 2.44 (m, 1H, H$_2$), 2.40 – 2.33 (m, 1H, H$_{9a}$), 2.16-2.23 (m, 1H, H$_{9b}$), 1.94 (ddd, *J* = 18.6, 12.5, 10.0 Hz, 1H, H$_{3a}$), 1.72 (dtd, *J* = 14.6, 8.9, 5.9 Hz, 1H, H$_{3b}$). **^{13}C-NMR (151 MHz, CDCl$_3$)** δ 179.5 (C$_1$), 158.1 (C$_8$), 132.8 (C$_5$), 129.4 (C$_6$), 114.0 (C$_7$), 66.5 (C$_{10}$), 55.3 (O*Me*), 38.4 (C$_2$), 32.5 (C$_4$), 32.2(C$_3$), 28.9 (C$_9$). **IR (ATR – neat)** $\tilde{\nu}$ (cm^{-1}) 2914, 1763, 1611, 1510, 1374, 1243, 1023, 824. **HRMS for [C$_{13}$H$_{17}$O$_3$]$^+$** calc. 221,1172, found 221.1156. ***R*f** (1:4 EtOAc/PE) = 0.32.

2-(4-methoxybenzyl)cyclopentanone (3aj)

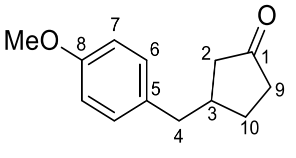

Obtained following **GP (I)** at 0.2 mmol scale using 0.8 mmol of cyclopent-2-en-1-one. Purification by column chromatography on silica gel (15% EtOAc in hexane) afforded product **3aj** (25 mg, 0.12 mmol) as yellowish oil in 62% yield. **^{1}H-NMR (400 MHz, CDCl$_3$)** δ 7.08 (d, *J* = 8.2 Hz, 2H, H$_6$), 6.84 (d, *J* = 8.2 Hz, 2H, H$_7$), 3.79 (s, 3H, O*Me*), 2.68 (d, *J* = 6.2 Hz, 2H, H$_4$), 2.44 (sept, *J* = 7.3 Hz, 1H, H$_3$), 2.31 (ddd, *J* = 16.6, 10.0, 6.7 Hz, 2H, H$_{2a,}$ H$_{9a}$), 2.21–2.00

(m, 2H, $H_{9b,}$ H_{10a}), 1.90 (dd, J = 18.0, 9.7 Hz, 1H, H_{2b}), 1.67 – 1.54 (m, 1H, H_{10b}).**^{13}C-NMR (101 MHz, $CDCl_3$)** δ 219.4 (C_1), 158.1 (C_8), 132.1 (C_5), 129.7 (C_6), 113.8 (C_7), 55.3 (O*Me*), 44.9 (C_4), 40.6 (C_2), 39.0 (C_9), 38.3 (C_3), 29.0 (C_{10}). **IR (ATR – neat)** $\tilde{\nu}$ (cm^{-1}) = 2920, 1691, 1603, 1460, 1116, 892, 765. **HRMS for $[C_{13}H_{17}O_2]^+$** calc. 205.1223, found 205.1220. ***R*f** (1:4 EtOAc/PE) = 0.57.

3-(4-methoxybenzyl)cyclohexanone (3ak)

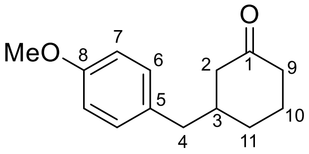

Obtained following **GP (I)** at 0.2 mmol scale using 0.8 mmol of cyclohex-2-en-1-one. Purification by column chromatography on silica gel (15% EtOAc in hexane) afforded product **3ak** (25 mg, 0.12 mmol) as yellowish oil in 58% yield. **^{1}H-NMR (400 MHz, $CDCl_3$)** δ 7.04 (d, J = 8.1 Hz, 2H, H_6), 6.83 (d, J = 8.1 Hz, 2H, H_7), 3.79 (s, 3H, O*Me*), 2.57 (d, J = 4.8 Hz, 2H, H_4), 2.39-2.21 (m, 3H, $H_{2a,}$ $H_{3,}$ H_{9a}), 2.10–1.96 (m, 3H, H_{2b}, $H_{9a,}$ H_{11a}), 1.92-1.81 (m, 1H, H_{10a}), 1.63–1.54 (m, 1H, H_{10b}), 1.40-1.30 (m,1H, H_{11b}). **^{13}C-NMR (101 MHz, $CDCl_3$)** δ 211.8 (C_1), 158.0 (C_8), 131.5 (C_5), 130.0 (C_6), 113.8 (C_7), 55.3 (O*Me*), 47.8 (C_2), 42.0 (C_9), 41.4 (C_4), 41.1 (C_3), 30.8 (C_{11}), 25.1 (C_{10}). **HRMS for $[C_{14}H_{19}O_2]^+$** calc. 219.1380, found 219.1375. ***R*f** (1:4 EtOAc/PE) = 0.58. Spectroscopic data were consistent with literature values.[11]

Diethyl 2-(4-methoxybenzyl)succinate (3al)

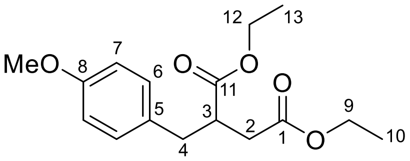

Obtained following **GP (I)** at 0.2 mmol scale using 0.8 mmol of diethyl fumarate. Purification by column chromatography on silica gel (10% EtOAc in hexane) afforded product **3al** (46 mg, 0.16 mmol) as yellowish oil in 78% yield. **^{1}H-NMR (400 MHz, $CDCl_3$)** δ 7.08 (d, J = 8.6 Hz, 2H, H_6), 6.82 (d, J = 8.6 Hz, 2H, H_7), 4.12 (q, J = 7.1 Hz, 2H, H_9 or H_{12}), 4.09 (q, J = 7.1 Hz, 2H, H_{12} or H_9), 3.78 (s, 3H, O*Me*), 3.06 (dddd, J = 9.2, 8.1, 6.5, 4.9 Hz, 1H, H_2), 2.98 (dd, J = 13.6, 6.5 Hz, 1H, H_{4a}), 2.75 – 2.59 (m, 2H, H_{4b} and H_{2a}), 2.38 (dd, J = 16.7, 5.0 Hz, 1H, H_{2b}), 1.22 (t, J = 7.1 Hz, 3H, H_{10} or H_{13}), 1.20 (t, J = 7.1 Hz, 3H, H_{13} or H_{10}). **^{13}C-NMR (101 MHz, $CDCl_3$)** δ 174.3 (C_{11}), 171.9 (C_1), 158.4 (C_8), 130.3 (C_5), 130.0 (C_6), 113.9 (C_7), 60.7 (C_{12} or C_9), 60.6 (C_9 or C_{12}), 55.3 (O*Me*), 43.3 (C_3), 37.0 (C_4), 35.2 (C_2), 14.2 (C_{10} or C_{13}), 14.1 (C_{13} or C_{10}). **HRMS for $[C_{16}H_{23}O_5]^+$** calc. 295.1540, found 295.1543. ***R*f** (1:4 EtOAc/PE) = 0.50. Spectroscopic data were consistent with literature values.[16]

4-(3-(4-methoxyphenyl)propyl)pyridine (3am)

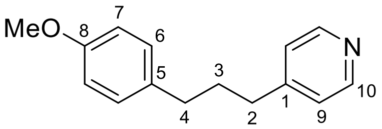

Obtained following **GP (I)** at 0.2 mmol scale using 0.8 mmol of 4-vinyl pyridine. Purification by column chromatography on silica gel (15% EtOAc in hexane) afforded product **3am** (20 mg, 0.09 mmol) as colourless oil in 45% yield. **^{1}H-NMR (600 MHz, $CDCl_3$)** δ 8.49 (d, *J* = 6.1 Hz, 2H, H_{10}), 7.13 – 7.07 (m, 4H, H_6 and H_9), 6.84 (d, *J* = 8.6 Hz, 2H, H_7), 3.79 (s, 3H, O*Me*), 2.62 (t, *J* = 7.7 Hz, 2H, H_4), 2.60 (t, *J* = 7.7 Hz, 2H, H_2), 1.94 (p, *J* = 7.7 Hz, 2H, H_3). **^{13}C-NMR (151 MHz, $CDCl_3$)** δ 157.9 (C_8), 151.3 (C_1), 149.6 (C_{10}), 133.6 (C_5), 129.3 (C_6), 123.9 (C_9), 113.8 (C_7), 55.2 (O*Me*), 34.5 (C_2), 34.3 (C_4), 32.0 (C_3). **HRMS for $[C_{15}H_{18}NO]^+$** calc. 228.1383, found 228.1380. ***R*$_f$** (1:4 EtOAc/PE) = 0.50. Spectroscopic data were consistent with literature values.[11]

2-(3-(4-methoxyphenyl)propyl)pyridine (3an)

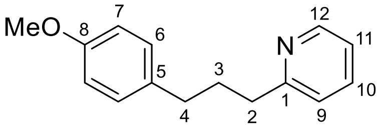

Obtained following **GP (I)** at 0.2 mmol scale using 0.4 mmol of 2-vinyl pyridine. Purification by column chromatography on silica gel (20% EtOAc in hexane) afforded product **3an** (35 mg, 0.15 mmol) as yellowish oil in 77% yield. **^{1}H-NMR (300 MHz, $CDCl_3$)** δ 8.53 (ddd, *J* = 4.9, 1.7, 0.8 Hz, 1H, H_{12}), 7.58 (td, *J* = 7.6, 1.9 Hz, 1H, H_{10}), 7.16 – 7.06 (m, 4H, H_6, H_9 and H_{11}), 6.82 (d, *J* = 8.6 Hz, 2H, H_7), 3.78 (s, 3H, O*Me*), 2.81 (t, *J* = 7.8 Hz, 2H, H_4), 2.63 (t, *J* = 7.5 Hz, 2H, H_2), 2.03 (p, *J* = 7.8 Hz, 2H, H_3). **^{13}C-NMR (101 MHz, $CDCl_3$)** δ 162.0 (C_1), 157.7 (C_8), 149.2 (C_{12}), 136.3 (C_{10}), 134.2 (C_5), 129.3 (C_6), 122.8 (C_9), 121.0 (C_{11}), 113.7 (C_7), 55.3 (O*Me*), 37.8 (C_2), 34.6 (C_4), 31.7 (C_3). **IR (ATR – neat)** $\tilde{\nu}$ (cm^{-1}) = 2926, 2854, 1510, 1434, 1242, 1176, 1035, 828, 748. **HRMS for $[C_{15}H_{18}NO]^+$** calc. 228.1383, found 228.1382. ***R*$_f$** (1:4 EtOAc/PE) = 0.30.

2-(3-(4-methoxyphenyl)-1-phenylpropyl)pyridine (3ao)

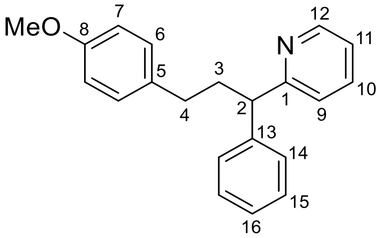

Obtained following **GP (I)** at 0.2 mmol scale using 0.4 mmol of 2-(1-phenylvinyl) pyridine (**2o**). Purification by column chromatography on silica gel (7% EtOAc in hexane) afforded product **3ao** (32 mg, 0.10 mmol) as colourless oil in 52% yield. **^{1}H-NMR (400 MHz, $CDCl_3$)** δ 8.57 (d, *J* = 4.8 Hz, 1H, H_{12}), 7.55 (t, *J* = 7.7 Hz, 1H, H_{10}), 7.37 – 7.26 (m, 4H, H_9, H_{11} and H_{15}), 7.19 (t, *J* = 7.0 Hz, 1H, H_{16}), 7.13 (d, *J* = 7.9 Hz, 1H, H_{14a}), 7.10 – 7.04 (m, 3H, H_6 and H_{14b}), 6.81 (d, *J* = 8.1 Hz, 2H, H_7), 4.06 (t, *J* = 7.4 Hz, 1H, H_2), 3.78 (s, 3H, O*Me*), 2.59 – 2.49

(m, 3H, H_4, H_{3a}), 2.38 (dd, J = 11.7, 7.5 Hz, 2H, H_{3b}). **^{13}C-NMR (101 MHz, $CDCl_3$)** δ 163.7 (C_1), 157.7 (C_8), 149.3 (C_{12}), 143.6 (C_{13}), 136.4 (C_{10}), 134.1 (C_5), 129.4 (C_6), 128.5 (C_{11}), 128.1 (C_{15}), 126.4 (C_{14}), 122.8 (C_{16}), 121.3 (C_9), 113.7 (C_7), 55.3 (O*Me*), 52.9 (C_2), 36.8 (C_4), 33.0 (C_3). **IR (ATR – neat)** $\tilde{\nu}$ (cm^{-1}) = 3003, 2929, 2833, 1610, 1568, 1431, 1242, 1176, 1033, 827, 746, 699, 536. **HRMS for $[C_{21}H_{22}NO]^+$** calc. 304.1696, found 304.1694. ***R*f** (1:4 EtOAc/PE) = 0.80.

1,2,3,4,5-pentafluoro-6-(3-(4-methoxyphenyl)propyl)benzene (3ap)

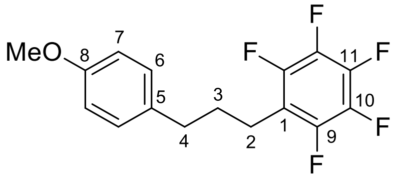

Obtained following **GP (I)** at 0.2 mmol scale using 0.4 mmol of 1,2,3,4,5-pentafluoro-6-vinylbenzene. Purification by column chromatography on silica gel (5% EtOAc in hexane) afforded product **3ap** (40 mg, 0.13 mmol) as colourless oil in 64% yield. **^{1}H-NMR (400 MHz, $CDCl_3$)** δ 7.09 (d, J = 8.6 Hz, 2H, H_6), 6.83 (d, J = 8.6 Hz, 2H, H_7), 3.79 (s, 3H, O*Me*), 2.71 (t, J = 7.7 Hz, 2H, H_2), 2.61 (t, J = 7.8 Hz, 2H, H_4), 1.88 (p, J = 7.7 Hz, 2H, H_3). **^{13}C-NMR (101 MHz, $CDCl_3$)** δ 157.9 (C_8), 146.2 (q, J = 10 Hz, C_{10a}), 143.8 (q, J = 10 Hz, C_{10b}), 140.7 (q, J = 5 Hz, C_{11}), 138.7 (t, J = 5 Hz, C_{9a}), 136.2 (t, J = 5 Hz, C_{9b}), 133.2 (C_5), 129.2 (C_6), 115.2 (t, J = 19 Hz, C_1), 113.8 (C_7), 55.2 (O*Me*), 34.5 (C_4), 30.9 (C_3), 22.0 (C_2). **HRMS for $[C_{16}H_{14}F_5O]^+$** calc. 317.0959, found 317.0962. ***R*f** (1:4 EtOAc/PE) = 0.20. Spectroscopic data were consistent with literature values.[17]

3-(4-methoxybenzyl) chroman-4-one (3aq)

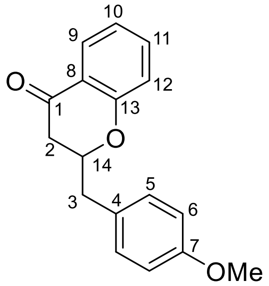

Obtained following **GP (I)** at 0.2 mmol scale using 0.4 mmol of 4*H*-chromen-4-one. Purification by column chromatography on silica gel (10% EtOAc in hexane) afforded product **3aq** (17 mg, 0.064 mmol) as yellowish oil in 32% yield. **^{1}H-NMR (400 MHz, $CDCl_3$)** δ 7.86 (dd, J = 7.8, 1.8 Hz, 1H, H_9), 7.47 (ddd, J = 8.6, 7.2, 1.8 Hz, 1H, H_{11}), 7.17 (d, J = 8.6 Hz, 2H, H_5), 7.03 – 6.96 (m, 2H, H_{10} and H_{12}), 6.87 (d, J = 8.6 Hz, 2H, H_6), 4.63 (dq, J = 8.6, 6.5 Hz, 1H, H_{10}), 3.80 (s, 3H, O*Me*), 3.15 (dd, J = 14.1, 6.2 Hz, 1H, H_{2a}), 2.99 (dd, J = 14.1, 6.5 Hz, 1H, H_{3a}), 2.70 – 2.62 (m, 2H, H_{2a}, H_{3a}). **^{13}C-NMR (101 MHz, $CDCl_3$)** δ 192.4 (C_1), 161.5 (C_{13}), 158.6 (C_7), 136.0 (C_{11}), 130.6 (C_4), 128.1 (C_9), 126.9 (C_8), 121.3 (C_{10}), 118.0 (C_{12}), 114.0 (C_6), 55.3 (O*Me*), 78.5 (C_{14}), 42.1 (C_2), 40.3 (C_3), **IR (ATR – neat)** $\tilde{\nu}$ (cm^{-1}) = 2909, 1736, 1651,

1507, 1132, 1100, 981, 699. **HRMS for [$C_{17}H_{17}O_3$]$^+$** calc. 269.1172, found 269.1175. ***R*f** (1:4 EtOAc/PE) = 0.50.

3-(4-methoxybenzyl)-2-phenylchroman-4-one (3ar)

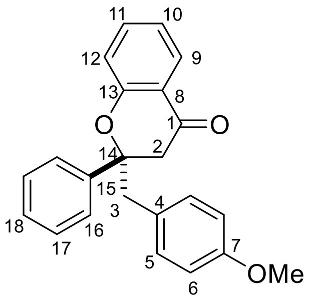

Obtained following **GP (I)** at 0.2 mmol scale using 0.8 mmol of 3-phenyl-4*H*-chromen-4-one. Purification by column chromatography on silica gel (10% EtOAc in hexanes) afforded product **3ar** (17 mg, 0.064 mmol) as yellowish oil in 22% yield. **^{1}H-NMR (400 MHz, $CDCl_3$)** δ 7.69 (d, *J* = 7.8 Hz, 1H, H_9), 7.45 (t, *J* = 7.8 Hz, 1H, H_{11}), 7.31-7.26 (m, 2H, H_{16}), 7.26 – 7.15 (m, 3H, H_{17}, H_{18}), 7.09 (d, *J* = 8.3 Hz, 1H, H_{10}), 6.90 (d, *J* = 8.2 Hz, 3H, H_5, H_{12}), 6.75 (d, *J* = 8.2 Hz, 2H, H_6), 3.77 (s, 3H, O*Me*), 3.31 (d, *J* = 13.9 Hz, 1H, H_{2a}), 3.23 (d, *J* = 16.5 Hz, 1H, H_{2b}), 3.10 (t, *J* = 15.3 Hz, 2H, H_3). **^{13}C-NMR (101 MHz, $CDCl_3$)** δ 191.8 (C_1), 159.9 (C_{13}), 158.6 (C_7), 141.1 (C_{15}), 136.1 (C_{11}), 131.8 (C_5), 128.4 (C_4), 127.7 (C_{17}), 127.2 (C_{16}), 126.5 (C_9), 126.2 (C_{18}), 121.4 (C_8), 121.0 (C_{10}), 118.3 (C_{12}), 113.4 (C_6), 85.0 (C_{14}), 55.2 (C_2), 48.7 (C*OMe*), 45.2 (C_3). **IR (ATR – neat)** $\tilde{\nu}$ (cm^{-1}) = 2914, 1687, 1605, 1459, 1302, 1176, 1030, 734, 696. **HRMS for [$C_{23}H_{21}O_3$]$^+$** calc. 345.1485, found 345.1480. ***R*f** (1:4 EtOAc/PE) = 0.54.

5.2 Alkyl boronic esters scope

5.2.1 General Procedure II

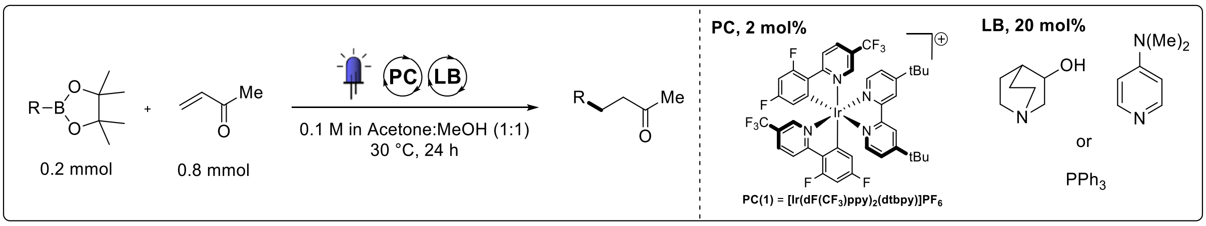

General Procedure II - GP (II): A 5 mL glass vial equipped with a magnetic stir bar was charged with the desired boronic ester (0.20 mmol), the photoredox catalyst Ir(dF(CF_3)ppy_2)(dtbpy)]PF_6 (4.4 mg, 2 mol%) and the Lewis base catalyst (0.04 mmol, 20 mol%). The vial was then sealed with a rubber septum and evacuated/backfilled with argon three times. Methyl vinyl ketone (**2d**, 66 µL, 0.8 mmol, 4.0 equiv.) was then added followed by 2.0 mL of a degassed acetone/methanol (1:1) mixture to lead a clear yellow transparent 0.1 M solution. This solution was then stirred while irradiated with a commercial blue LED strip (14.4 W, 450 nm) for 24 hours, the temperature was maintained at 30°C using a desktop fan. The content of the vial was then concentrated and immobilized on Isolute HM-N for easy dry loading on flash column chromatography to yield the pure product.

5.2.2 Characterization of coupling products

5-phenylpentan-2-one (3bd)

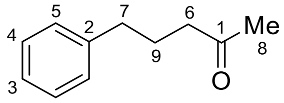

Obtained following **GP (II)** using 2-benzyl-4,4,5,5-tetramethyl-1,3,2-dioxaborolane (44 mg, 0.20 mmol) and quinuclidin-3-ol (5.0 mg, 40 µmol, 20 mol%) as Lewis base catalyst. Purification by column chromatography on silica gel (0% to 5% EtOAc in hexane) afforded product **3bd** (21 mg, 0.13 mmol) as a colourless oil in 65% yield. **^{1}H-NMR (600 MHz, $CDCl_3$)** δ 7.29 (t, J = 7.6 Hz, 2H, H_3), 7.22 – 7.15 (m, 3H, H_4, H_5), 2.62 (t, J = 7.5 Hz, 2H, H_7), 2.44 (t, J = 7.5 Hz, 2H, H_6), 2.12 (s, 3H, H_8), 1.91 (p, J = 7.5 Hz, 2H, H_9). **^{13}C-NMR (151 MHz, $CDCl_3$)** δ 208.9 (C_1), 141.7 (C_2), 128.6 (C_3), 128.5 (C_4), 126.1 (C_5), 43.0 (C_6), 35.2 (C_7), 30.1 (C_8), 25.3 (C_9). **IR (ATR – neat)** $\tilde{\nu}$ (cm^{-1}) = 1712, 1454, 1356, 1159, 1031, 745, 699. **HRMS for [$C_{11}H_{15}O$]$^+$** calc. 163.1117 found 163.1121. ***R*f** (1:4 EtOAc/PE) = 0.54. Spectroscopic data were consistent with literature values.[18]

5-(4-methoxyphenyl)pentan-2-one (3ad)

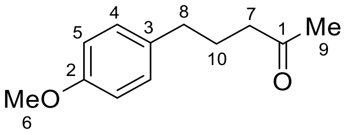

Obtained following **GP (II)** using 2-(4-methoxybenzyl)-4,4,5,5-tetramethyl-1,3,2-dioxaborolane (50 mg, 0.20 mmol) and quinuclidin-3-ol (5.0 mg, 40 µmol, 20 mol%) as Lewis base catalyst. Purification by column chromatography on silica gel (0% to 5% EtOAc in hexane) afforded product **3ad** (32 mg, 0.16 mmol) as a colourless oil in 82% yield. **^{1}H-NMR (600 MHz, $CDCl_3$)** δ 7.08 (d, *J* = 8.6 Hz, 2H, H_4), 6.83 (d, *J* = 8.6 Hz, 2H, H_5), 3.79 (s, 3H, H_6), 2.56 (t, *J* = 7.5 Hz, 2H, H_8), 2.42 (t, *J* = 7.5 Hz, 2H, H_7), 2.11 (s, 3H, H_9), 1.87 (p, *J* = 7.5 Hz, 2H, H_{10}). **^{13}C-NMR (151 MHz, $CDCl_3$)** δ 209.0 (C_1), 158.0 (C_2), 133.7 (C_3), 129.5 (C_4), 113.9 (C_5), 55.4 (C_6), 42.9 (C_7), 34.2 (C_8), 30.1 (C_9), 25.6 (C_{10}). **IR (ATR – neat)** $\tilde{\nu}\ (cm^{-1})$ = 1712, 1611, 1511, 1243, 1177, 1033, 827, 811. **HRMS for [$C_{12}H_{17}O_2$]$^+$** calc. 193.1223 found 193.1228. ***R*f** (1:4 EtOAc/PE) = 0.38. Spectroscopic data were consistent with literature values.[19]

5-(*p*-tolyl)pentan-2-one (3cd)

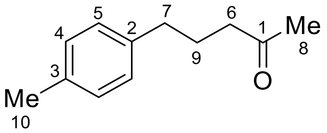

Obtained following **GP (II)** using 4,4,5,5-tetramethyl-2-(4-methylbenzyl)-1,3,2-dioxaborolane (46 mg, 0.20 mmol) and quinuclidin-3-ol (5.0 mg, 40 µmol, 20 mol%) as Lewis base catalyst. Purification by column chromatography on silica gel (0% to 5% EtOAc in hexane) afforded product **3cd** (23 mg, 0.13 mmol) as a colourless oil in 66% yield. **^{1}H-NMR (600 MHz, $CDCl_3$)** δ 7.10 (d, *J* = 7.9 Hz, 2H, H_4), 7.06 (d, *J* = 7.9 Hz, 2H, H_5), 2.59 (t, *J* = 7.5 Hz, 2H, H_7), 2.43 (t, *J* = 7.5 Hz, 2H, H_6), 2.32 (s, 3H, H_{10}), 2.12 (s, 3H, H_8), 1.89 (p, *J* = 7.5 Hz, 2H, H_9). **^{13}C-NMR (151 MHz, $CDCl_3$)** δ 209.0 (C_1), 138.6 (C_2), 135.5 (C_3), 129.2 (C_4), 128.5 (C_5), 43.0 (C_6), 34.7 (C_7), 30.1 (C_8), 25.4 (C_9), 21.1 (C_{10}). **IR (ATR – neat)** $\tilde{\nu}\ (cm^{-1})$ = 1714, 1515, 1356, 1160, 808. **HRMS for [$C_{12}H_{17}O$]$^+$** calc. 177.1274 found 177.1278. ***R*f** (1:4 EtOAc/PE) = 0.56. Spectroscopic data were consistent with literature values.[20]

5-(4-fluorophenyl)pentan-2-one (3dd)

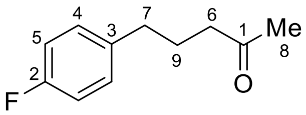

Obtained following **GP (II)** using 2-(4-fluorobenzyl)-4,4,5,5-tetramethyl-1,3,2-dioxaborolane (47 mg, 0.20 mmol) and quinuclidin-3-ol (5.0 mg, 40 µmol, 20 mol%) as Lewis base catalyst. Purification by column chromatography on silica gel (0% to 5% EtOAc in hexane) afforded product **3dd** (28 mg, 0.15 mmol) as a colourless oil in 71% yield. **^{1}H-NMR (600 MHz, $CDCl_3$)** δ 7.12 (m, 2H, H_4), 6.96 (m, 2H, H_5), 2.59 (t, *J* = 7.5 Hz, 2H, H_7), 2.42 (t, *J* = 7.5 Hz, 2H, H_6), 2.12 (s, 3H, H_8), 1.88 (p, *J* = 7.5 Hz, 2H, H_9). **^{13}C-NMR (151 MHz, $CDCl_3$)** δ 208.7 (C_1), 161.5

(d, J = 243.5 Hz, C_2), 137.3 (d, J = 3.3 Hz, C_3), 129.9 (d, J = 7.8 Hz, C_4), 115.3 (d, J = 21.1 Hz, C_5), 42.8 (C_6), 34.3 (C_7), 30.1 (C_8), 25.4 (C_9). **^{19}F NMR (376 MHz, $CDCl_3$)** δ -117.6. **IR (ATR – neat)** $\tilde{\nu}$ (cm^{-1}) = 1713, 1508, 1357, 1218, 1157, 830, 752. **HRMS for $[C_{11}H_{14}OF]^+$** calc. 181.1023 found 181.1022. ***R*f** (1:4 EtOAc/PE) = 0.41. Spectroscopic data were consistent with literature values.[19]

5-(3,5-dimethoxyphenyl)pentan-2-one (3ed)

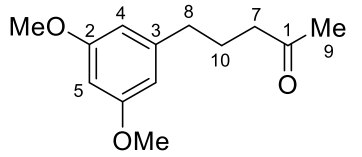

Obtained following **GP (II)** using 2-(3,5-dimethoxybenzyl)-4,4,5,5-tetramethyl-1,3,2-dioxaborolane (56 mg, 0.20 mmol) and quinuclidin-3-ol (5.0 mg, 40 µmol, 20 mol%) as Lewis base catalyst. Purification by column chromatography on silica gel (5% to 10% EtOAc in hexane) afforded product **3ed** (36 mg, 0.13 mmol) as a colourless oil in 63% yield. **^{1}H-NMR (600 MHz, $CDCl_3$)** δ 6.32 (d, J = 2.3 Hz, 2H, H_4), 6.30 (t, J = 2.3 Hz, 1H, H_5), 3.77 (s, 6H, H_6), 2.56 (t, J = 7.4 Hz, 2H, H_8), 2.43 (t, J = 7.4 Hz, 2H, H_7), 2.11 (s, 3H, H_9), 1.89 (p, J = 7.4 Hz, 2H, H_{10}). **^{13}C-NMR (151 MHz, $CDCl_3$)** δ 208.9 (C_1), 160.9 (C_2), 144.1 (C_3), 106.6 (C_4), 98.0 (C_5), 55.4 (C_6), 42.9 (C_7), 35.4 (C_8), 30.1 (C_9), 25.0 (C_{10}). **IR (ATR – neat)** $\tilde{\nu}$ (cm^{-1}) = 1713, 1594, 1460, 1428, 1354, 1203, 1147, 1056, 830, 695. **HRMS for $[C_{13}H_{19}O_3]^+$** calc. 223.1329 found 223.1328. ***R*f** (1:4 EtOAc/PE) = 0.28.

5-(phenylthio)pentan-2-one (3fd)

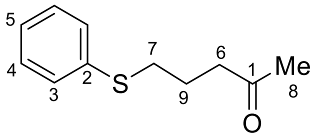

Obtained following **GP (II)** using 4,4,5,5-tetramethyl-2-((phenylthio)methyl)-1,3,2-dioxaborolane (50 mg, 0.20 mmol) and quinuclidin-3-ol (5.0 mg, 40 µmol, 20 mol%) as Lewis base catalyst. Purification by column chromatography on silica gel (5% to 7% EtOAc in hexane) afforded product **3fd** (35 mg, 0.18 mmol) as a colourless oil in 91% yield. **^{1}H-NMR (600 MHz, $CDCl_3$):** δ 7.35 – 7.31 (m, 2H, H_3), 7.30 – 7.26 (m, 2H, H_4), 7.20 – 7.14 (m, 1H, H_5), 2.94 (t, J = 7.0 Hz, 2H, H_7), 2.60 (t, J = 7.0 Hz, 2H, H_6), 2.12 (s, 3H, H_8), 1.90 (p, J = 7.0 Hz, 2H, H_9). **^{13}C-NMR (151 MHz, $CDCl_3$)** δ 208.1 (C_1), 136.2 (C_2), 129.3 (C_3), 129.0 (C_4), 126.1 (C_5), 42.0 (C_6), 33.1 (C_7), 30.1 (C_8), 23.1 (C_9). **IR (ATR – neat)** $\tilde{\nu}$ (cm^{-1}) = 1711, 1583, 1481, 1438, 1365, 1175, 1089, 1025, 738, 690. **HRMS for $[C_{11}H_{15}OS]^+$** calc. 195.0844 found 195.0838. ***R*f** (1:4 EtOAc/PE) = 0.43. Spectroscopic data were consistent with literature values.[21]

***tert*-butyl (4-oxopentyl)carbamate (3gd)**

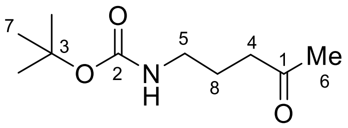

Obtained following **GP (II)** using *tert*-butyl ((4,4,5,5-tetramethyl-1,3,2-dioxaborolan-2-yl)methyl)carbamate (51 mg, 0.20 mmol, 1.0 equiv.) and PPh_3 (10 mg, 40 µmol, 20 mol%) as Lewis base catalyst. Purification by column chromatography on silica gel (10% to 60% EtOAc and 1% Et_3N in hexane) afforded product **3gd** (37 mg, 0.19 mmol) as a colourless oil in 91% yield. **^{1}H-NMR (600 MHz, $CDCl_3$)** δ 4.60 (s, 1H, N*H*), 3.13 (m, 2H, H_5), 2.49 (t, *J* = 7.1 Hz, 2H, H_4), 2.16 (s, 3H, H_6), 1.76 (p, *J* = 7.1 Hz, 2H, H_8), 1.44 (s, 9H, H_7). **^{13}C-NMR (151 MHz, $CDCl_3$)** δ 208.6 (C_1), 156.2 (C_2), 79.4 (C_3), 40.9 (C_4), 40.1 (C_5), 30.1 (C_6), 28.6 (C_7), 24.2 (C_8). **IR (ATR – neat)** $\tilde{\nu}$ (cm^{-1}) = 3363, 1691, 1517, 1366, 1269, 1249, 1163, 863, 782. **HRMS for $[C_{10}H_{19}O3NNa]^+$** calc. 224.1257 found 224.1258. ***R*f** (1:1 EtOAc/PE) = 0.50. Spectroscopic data were consistent with literature values.[22]

***tert*-butyl methyl(4-oxopentyl)carbamate (3hd)**

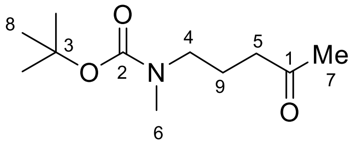

Obtained following **GP (II)** using *tert*-butyl methyl((4,4,5,5-tetramethyl-1,3,2-dioxaborolan-2-yl)methyl)carbamate (54 mg, 0.20 mmol) and PPh_3 (10 mg, 40 µmol, 20 mol%) as Lewis base catalyst. Purification by column chromatography on silica gel (10% EtOAc and 1% Et_3N in hexane) afforded product **3hd** (35 mg, 0.16 mmol) as a colourless oil in 86% yield. **^{1}H-NMR (600 MHz, $CDCl_3$)** δ 3.19 (t, *J* = 7.1 Hz, 2H, H_4), 2.80 (s, 3H, H_6), 2.41 (t, *J* = 7.1 Hz, 2H, H_5), 2.12 (s, 3H, H_7), 1.75 (p, *J* = 7.1 Hz, 2H, H_9), 1.43 (s, 9H, H_8). **^{13}C-NMR (151 MHz, $CDCl_3$)** δ 208.2 (C_1), 156.0 (C_2), 79.4 (C_3) 47.9 (d, C_4), 40.5 (C_5), 34.1 (C_6), 30.1 (C_7), 28.6 (C_8), 21.8 (C_9). **IR (ATR – neat)** $\tilde{\nu}$ (cm^{-1}) = 1716, 1687, 1393, 1364, 1160, 1134, 881, 772. **HRMS for $[C_{11}H_{21}O_3N]^+$** calc. 215.1521 found 215.1519. ***R*f** (1:4 EtOAc/PE) = 0.32. Spectroscopic data were consistent with literature values.[23]

5-(*p*-tolyl)hexan-2-one (3id)

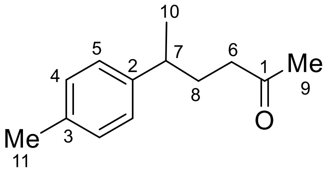

Obtained following **GP (II)** using 4,4,5,5-tetramethyl-2-(1-(*p*-tolyl)ethyl)-1,3,2-dioxaborolane (50 mg, 0.20 mmol) and DMAP (4.8 mg, 40 µmol, 20 mol%) as Lewis base catalyst. Purification by column chromatography on silica gel (0% to 5% EtOAc in hexane) afforded product **3id** (28 mg, 0.15 mmol) as a colourless oil in 74% yield. **^{1}H-NMR (600 MHz, $CDCl_3$)** δ 7.11 (d, *J* = 8.0 Hz, 2H, H_4), 7.06 (d, *J* = 8.0 Hz, 2H, H_5), 2.69 – 2.61 (m, 1H, H_7), 2.36 – 2.23

(m, 5H, H_6, H_{11}), 2.06 (s, 3H, H_9), 1.89 (m, 1H, H_{8a}), 1.84 – 1.76 (m, 1H, H_{8b}), 1.25 (d, J = 6.9 Hz, 3H, H_{10}). **^{13}C-NMR (151 MHz, $CDCl_3$)** δ 209.1 (C_1), 143.5 (C_2), 135.7 (C_3), 129.3 (C_4), 127.0 (C_5), 42.0 (C_6), 39.1 (C_7), 32.0 (C_8), 30.1 (C_9), 22.7 (C_{10}), 21.1 (C_{11}). **IR (ATR – neat)** $\tilde{\nu}\ (cm^{-1})$ = 1715, 1515, 1456, 1355, 1161, 817, 723. **HRMS for [$C_{13}H_{19}O$]$^+$** calc. 191.1430 found 191.1429. ***R*f** (1:4 EtOAc/PE) = 0.49. Spectroscopic data were consistent with literature values.[24]

5-phenylhexan-2-one (3jd)

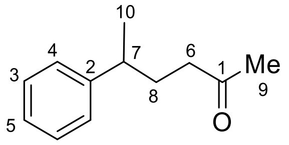

Obtained following **GP (II)** using 4,4,5,5-tetramethyl-2-(1-phenylethyl)-1,3,2-dioxaborolane (46 mg, 0.20 mmol) and DMAP (4.8 mg, 40 µmol, 20 mol%) as Lewis base catalyst. Purification by column chromatography on silica gel (0% to 5% EtOAc in hexane) afforded product **3jd** (24 mg, 0.13 mmol) as a colourless oil in 67% yield. **^{1}H-NMR (600 MHz, $CDCl_3$)** δ 7.30 (t, J = 7.6 Hz, 2H, H_3), 7.23 – 7.18 (m, 1H, H_5), 7.18 – 7.14 (m, 2H, H_4), 2.74 – 2.64 (m, 1H, H_7), 2.40 – 2.23 (m, 2H, H_6), 2.05 (s, 3H, H_9), 1.91 (ddd, J = 13.8, 9.3, 6.0 Hz, 1H, H_{6a}), 1.86 – 1.78 (m, 1H, H_{6b}), 1.26 (d, J = 6.9 Hz, 3H, H_{10}). **^{13}C-NMR (151 MHz, $CDCl_3$)** δ 209.1 (C_1), 146.6 (C_2), 128.6 (C_3), 127.1 (C_4), 126.3 (C_5), 42.0 (C_6), 39.5 (C_7), 32.0 (C_8), 30.1 (C_9), 22.6 (C_{10}). **IR (ATR – neat)** $\tilde{\nu}\ (cm^{-1})$ = 1714, 1494, 1452, 1357, 1162, 763, 700. **HRMS for [$C_{12}H_{17}O$]$^+$** calc. 177.1274 found 177.1276. ***R*f** (1:4 EtOAc/PE) = 0.64. Spectroscopic data were consistent with literature values.[25]

5,6-diphenylhexan-2-one (3kd)

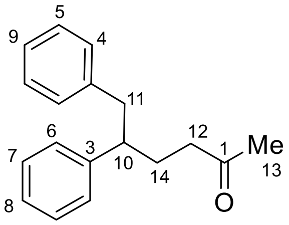

Obtained following **GP (II)** using 2-(1,2-diphenylethyl)-4,4,5,5-tetramethyl-1,3,2-dioxaborolane (62 mg, 0.20 mmol) and DMAP (4.8 mg, 40 µmol, 20 mol%) as Lewis base catalyst. Purification by column chromatography on silica gel (0% to 5% EtOAc in hexane) afforded product **3kd** (27 mg, 0.11 mmol) as a colourless oil in 55% yield. **^{1}H-NMR (600 MHz, $CDCl_3$)** δ 7.30 – 7.24 (m, 2H, H_5), 7.22 – 7.17 (m, 3H, H_6, H_8), 7.16 – 7.12 (m, 1H, H_9), 7.11 – 7.08 (m, 2H, H_7), 7.05 – 7.02 (m, 2H, H_4), 2.95 – 2.84 (m, 2H, H_{11}), 2.79 (m, 1H, H_{10}), 2.30 – 2.16 (m, 2H, H_{12}), 2.06 – 1.97 (m, 4H, H-13, H_{14a}), 1.85 (dddd, J = 14.2, 10.7, 8.9, 5.8 Hz, 1H, H_{14b}). **^{13}C-NMR (151 MHz, $CDCl_3$)** δ 208.9 (C_1), 144.2 (C_2), 140.4 (C_3), 129.2 (C_4), 128.6 (C_5), 128.2 (C_6), 127.9 (C_7), 126.5 (C_8), 126.0 (C_9), 47.4 (C_{10}), 44.1 (C_{11}), 41.9 (C_{12}), 30.0 (C_{13}), 29.3 (C_{14}). **IR (ATR – neat)** $\tilde{\nu}\ (cm^{-1})$ = 1713, 1495, 1452, 1358, 1158, 758, 698. **HRMS for [$C_{18}H_{21}O$]$^+$** calc. 253.1587 found 253.1586. ***R*f** (1:4 EtOAc/PE) = 0.58.

4-(1-(4-chlorophenyl)cyclobutyl)butan-2-one (3nd)

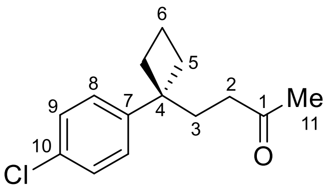

Obtained following **GP (II)** at 0.2 mmol scale using DMAP (4.8 mg, 40 µmol, 20 mol%) as Lewis base catalyst. Purification by column chromatography on silica gel (2% EtOAc in hexane) afforded product **3nd** (32 mg, 0.13 mmol) as a colourless oil in 66% yield. **^{1}H-NMR (600 MHz, $CDCl_3$)** δ 7.26 (d, J = 7.9 Hz, 2H, H_9), 7.03 (d, J = 8.1 Hz, 2H, H_8), 2.36 – 2.26 (m, 2H, H_{5a}), 2.18 – 2.03 (m, 7H, $H_{5b,}$ $H_{6b,}$ H_3 and H_2), 2.03 (s, 3H, H_{11}), 1.89 – 1.79 (m, 1H, H_{6a}). **^{13}C-NMR (151 MHz, $CDCl_3$)** δ 208.8 (C_1), 147.8 (C_7), 131.2 (C_{10}), 128.2 (C_9), 127.1 (C_8), 45.3 (C_4), 39.2 (C_2), 35.7 (C_3), 32.5 (C_5), 29.9 (C_{11}), 15.8 (C_6). **IR (ATR – neat)** $\tilde{\nu}$ (cm^{-1}) = 2976, 2952, 2853, 1715, 1491, 1364, 1245, 1161, 1092, 1013, 828, 721. **HRMS for $[C_{14}H_{18}OCl]^+$** calc. 237.1046 found 237.1037. ***R*f** (1:4 EtOAc/PE) = 0.47.

4-(4-(4-chlorophenyl)tetrahydro-2H-pyran-4-yl)butan-2-one (3od)

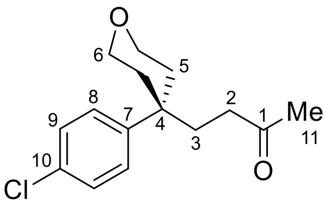

Obtained following **GP (II)** at 0.2 mmol scale using DMAP (4.8 mg, 40 µmol, 20 mol%) as Lewis base catalyst. Purification by column chromatography on silica gel (10% EtOAc in hexane) afforded product **3od** (27 mg, 0.10 mmol) as a colourless oil in 51% yield. **^{1}H-NMR (600 MHz, $CDCl_3$)** δ 7.33 (d, *J* = 8.6 Hz, 2H, H_9), 7.19 (d, *J* = 8.6 Hz, 2H, H_8), 3.80 – 3.76 (m, 2H, H_{6a}), 3.55 – 3.51 (m, 2H, H_{6b}), 2.10 – 2.03 (m, 4H, H_2 and H_{5a}), 2.00 (s, 3H, H_{11}), 1.91 – 1.87 (m, 2H, H_3), 1.83 – 1.78 (m, 2H, H_{5b}). **^{13}C-NMR (151 MHz, $CDCl_3$)** δ 208.2 (C_1), 143.2 (C_7), 132.0 (C_{10}), 128.7 (C_9), 128.2 (C_8), 64.1 (C_6), 38.4 (C_2), 37.8 (C_3), 36.2 (C_4), 36.1 (C_5), 29.9 (C_{11}). **IR (ATR – neat)** $\tilde{\nu}$ (cm^{-1}) = 2948, 2857, 1714, 1494, 1360, 1241, 1114, 1094, 1012, 824. **HRMS for $[C_{15}H_{20}O_2Cl]^+$** calc. 267.1152 found 267.1149. ***R*f** (1:4 EtOAc/PE) = 0.14.

5,5-dimethylhexan-2-one (3pd)

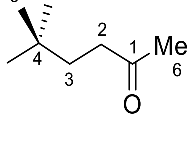

Obtained following **GP (II)** at 0.2 mmol scale using DMAP (4.8 mg, 40 µmol, 20 mol%) as Lewis base catalyst. Purification by column chromatography on silica gel (5% Et_2O in pentane) afforded product **3pd** (11 mg, 0.09 mmol) as a colourless oil in 45% yield. **^{1}H-NMR (600 MHz, $CDCl_3$)** δ 2.42 – 2.36 (m, 2H, H_3), 2.15 (s, 3H, H_6), 1.50 – 1.45 (m, 2H, H_2), 0.88 (s, 9H, H_5). **NMR (151 MHz, $CDCl_3$)** δ 209.8 (C_1), 39.7 (C_2), 37.5 (C_3), 30.1 (C_4), 30.0 (C_6), 29.3 (C_5). **HRMS for**

[$C_8H_{17}O$]$^+$ calc. 129.1274 found 129.1269. ***R*f** (1:4 EtOAc/PE) = 0.66. Spectroscopic data were consistent with literature values.[26]

5.3 Aryl and alkyl boronic acid scope

5.3.1 General Procedure III

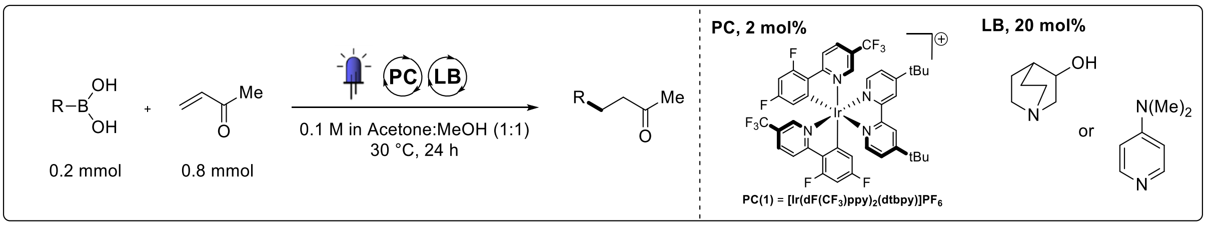

General Procedure III - GP (III): A 5 mL glass vial equipped with a magnetic stir bar was charged with the desired boronic acid (0.20 mmol), the photoredox catalyst Ir(dF(CF_3)ppy_2)(dtbpy)]PF_6 (2.2 mg, 2.0 mol%) and the Lewis base catalyst (0.04 mmol, 20 mol%). The vial was then sealed with a rubber septum and evacuated/backfilled with argon three times. Methyl vinyl ketone (66 µL, 0.8 mmol, 4.0 equiv.) was then added followed by 2.0 mL of a degassed acetone/methanol (1:1) mixture to lead a clear yellow transparent 0.1 M solution. This solution was then stirred while irradiated with a commercial blue LED strip (14.4 W, 450 nm) for 24 hours, the temperature was maintained at 30°C using a desktop fan. The content of the vial was then concentrated and immobilized on Isolute HM-N for easy dry loading on flash column chromatography to yield the pure product.

5.3.2 Characterization of coupling products

4-(4-methoxyphenyl)butan-2-one (5ad)

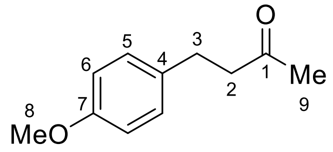

Obtained following **GP (III)** at 0.2 mmol scale using quinuclidin-3-ol (5.0 mg, 40 µmol, 20 mol%) as Lewis base catalyst. Purification by column chromatography on silica gel (3% EtOAc in hexane) afforded product **5ad** (21 mg, 0.12 mmol) as a colourless oil in 60% yield. **^{1}H-NMR (600 MHz, $CDCl_3$)** δ 7.10 (d, J = 8.6 Hz, 2H, H_5), 6.82 (d, J = 8.6 Hz, 2H, H_6), 3.78 (s, 3H, H_8), 2.83 (t, J = 7.6 Hz, 2H, H_3), 2.72 (t, J = 7.6 Hz, 2H, H_2), 2.13 (s, 3H, H_9). **^{13}C-NMR (151 MHz, $CDCl_3$)** δ 208.2 (C_9), 158.1 (C_7), 133.1 (C_4), 129.3 (C_5), 114.0 (C_6), 55.4 (C_8), 45.6 (C_2), 30.2 (C_3), 29.0 (C_9). **IR (ATR – neat)** $\tilde{\nu}$ (cm^{-1}) = 3004, 2921, 2833, 1713, 1611, 1510, 1362, 1300, 1241, 1179, 1157, 1033, 820. **HRMS for [$C_{11}H_{14}O_2$]$^+$** calc. 178.0994 found 178.0987. ***R*f** (1:4 EtOAc/PE) = 0.25.

4-(4-(methylthio)phenyl)butan-2-one (5bd)

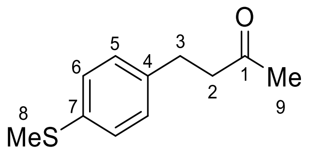

Obtained following **GP (III)** at 0.2 mmol scale using quinuclidin-3-ol (5.0 mg, 40 µmol, 20 mol%) as Lewis base catalyst. Purification by column chromatography on silica gel (3% EtOAc in hexane) afforded product **5bd** (28 mg, 0.144 mmol) as a pinkish oil in 72% yield. **^{1}H-NMR (600 MHz, $CDCl_3$)** δ 7.16 (d, *J* = 8.3 Hz, 2H, H_6), 7.08 (d, *J* = 8.2 Hz, 2H, H_5), 2.82 (d, *J* = 7.6 Hz, 2H, H_3), 2.71 (t, *J* = 7.6 Hz, 2H, H_2), 2.43 (s, 3H, H_8), 2.11 (s, 3H, H_9). **^{13}C-NMR (151 MHz, $CDCl_3$)** δ 207.7 (C_1), 138.0 (C_4), 135.7 (C_7), 128.8 (C_5), 127.1 (C_6), 45.0 (C_2), 30.0 (C_3), 29.1 (C_9), 16.1 (C_8). **IR (ATR – neat)** $\tilde{\nu}$ (cm^{-1}) = 2988, 2921, 1715, 1495, 1439, 1407, 1360, 1161, 1096, 1017, 967, 807. **HRMS for $[C_{11}H_{14}O_2]^+$** calc. 194.0765 found 194.0761. ***R*f** (1:4 EtOAc/PE) = 0.34.

***tert*-butyl (4-(3-oxobutyl)phenyl)carbamate (5cd)**

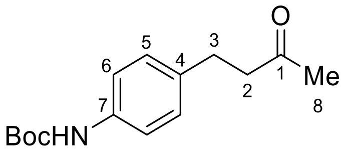

Obtained following **GP (III)** at 0.2 mmol scale using quinuclidin-3-ol (5.0 mg, 40 µmol, 20 mol%) as Lewis base catalyst. Purification by column chromatography on silica gel (15% EtOAc in hexane) afforded product **5cd** (35 mg, 0.13 mmol) as white needles in 67% yield. **^{1}H-NMR (600 MHz, $CDCl_3$)** δ 7.26 (d, *J* = 8.2 Hz, 2H, H_6), 7.08 (d, *J* = 8.2 Hz, 2H, H_5), 6.55 (bs, 1H, N*H*Boc), 2.82 (t, *J* = 7.6 Hz, 2H, H_3), 2.71 (t, *J* = 7.6 Hz, 2H, H_2), 2.11 (s, 3H, H_8), 1.50 (s, 9H, NHCOO*tBu*).**^{13}C-NMR (151 MHz, $CDCl_3$)** δ 208.2 (C_1), 153.0 (NH*C*OOtBu), 136.6 (C_7), 135.7 (C_4), 128.9 (C_5), 118.9 (C_6), 80.5 (NHCOO*C*(CH_3)$_3$), 45.3 (C_2), 30.2 (C_3), 29.2 (C_8), 28.4 (NHCOOC(*C*H_3)$_3$). **IR (ATR – neat)** $\tilde{\nu}$ (cm^{-1}) = 3675, 3333, 2972, 2925, 1709, 1598, 1526, 1413, 1366, 1314, 1235, 1157, 1056, 819, 773. **HRMS for $[C_{15}H_{21}NO_3]^+$** calc. 263.1521 found 263.1511. ***R*f** (2:3 EtOAc/PE) = 0.48. **M.p.** = 96–98 °C

N-(4-(3-oxobutyl)phenyl)acetamide (5dd)

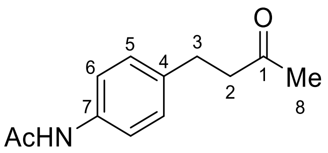

Obtained following **GP (III)** at 0.2 mmol scale using quinuclidin-3-ol (5.0 mg, 40 µmol, 20 mol%) as Lewis base catalyst. Purification by column chromatography on silica gel (60% EtOAc in hexane) afforded product **5dd** (32 mg, 0.16 mmol) as white needles in 78% yield. **^{1}H-NMR (600 MHz, $CDCl_3$)** δ 7.63 (bs, 1H, N*H*Ac), 7.39 (d, *J* = 8.5 Hz, 2H, H_6), 7.10 (d, *J* = 8.4 Hz, 2H, H_5), 2.84 (t, *J* = 7.5 Hz, 2H, H_2), 2.72 (t, *J* = 7.5 Hz, 2H, H_3), 2.13 (s, 3H, H_8), 2.12 (s, 3H, NHCO*Me*). **^{13}C-NMR (151 MHz, $CDCl_3$)** 208.3 (C_1), 168.6 (NH*C*OMe), 137.0 (C_7), 136.2 (C_4), 128.9 (C_5), 120.3 (C_6), 45.2 (C_2), 30.2 (C_3), 29.2 (C_8), 24.6 (NHCO*Me*). **IR (ATR – neat)** $\tilde{\nu}$ (cm^{-1}) = 3294, 3123, 3060, 2937, 1711, 1665, 1606, 1534, 1514, 1411, 1370, 1316, 1263, 1161, 819. **HRMS for $[C_{12}H_{15}NO_2]^+$** calc. 205.1103 found 205.1099. ***R*f** (EtOAc) = 0.33. **M.p.** = 104–106 °C.

N-(3-(3-oxobutyl)phenyl)acetamide (5ed)

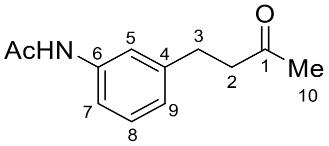

Obtained following **GP (III)** at 0.2 mmol scale using quinuclidin-3-ol (5.0 mg, 40 µmol, 20 mol%) as Lewis base catalyst. Purification by column chromatography on silica gel (40% EtOAc in hexane) afforded product **5ed** (34 mg, 0.17 mmol) as a colourless oil in 83% yield. **^{1}H-NMR (600 MHz, CDCl$_3$)** δ 7.38 (bs, 1H, N*H*Ac), 7.36 – 7.27 (m, 2H, H_5 and H_7), 7.21 (t, *J* = 7.8 Hz, 1H, H_8), 6.92 (d, *J* = 7.6 Hz, 1H, H_9), 2.87 (t, *J* = 7.5 Hz, 2H, H_3), 2.75 (t, *J* = 7.5 Hz, 2H, H_2), 2.16 (s, 3H, H_{11}), 2.13 (s, 3H, H_{10}). **^{13}C-NMR (151 MHz, CDCl$_3$)** δ 208.3 (C_1), 168.6 (NH*C*OMe), 142.1 (C_4), 138.1 (C_6), 129.2 (C_8), 124.4 (C_9), 119.8 (C_5), 117.7 (C_7), 45.1 (C_2), 30.3 (C_3), 29.7 (C_{10}), 24.8 (NHCO*Me*). **IR (ATR – neat)** $\tilde{\nu}$ (cm^{-1}) = 3298, 2964, 2929, 1713, 1669, 1611, 1594, 1552, 1491, 1437, 1421, 1372, 1314, 1262, 1161, 789, 698. **HRMS for [$C_{12}H_{15}NO_2$]$^+$** calc. 205.1103 found 205.1097. ***R*f** (Et_2O) = 0.40.

4-(2-methoxyphenyl)butan-2-one (5fd)

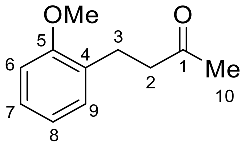

Obtained following **GP (III)** at 0.2 mmol scale using quinuclidin-3-ol (5.0 mg, 40 µmol, 20 mol%) as Lewis base catalyst. Purification by column chromatography on silica gel (5% EtOAc in hexane) afforded product **5fd** (18 mg, 0.10 mmol) as a colourless oil in 51% yield. **^{1}H-NMR (600 MHz, CDCl$_3$)** δ 7.19 (t, *J* = 7.8 Hz, 1H, H_7), 7.13 (d, *J* = 7.4 Hz, 1H, H_9), 6.87 (t, *J* = 7.4 Hz, 1H, H_8), 6.84 (d, *J* = 7.8 Hz, 1H, H_6), 3.82 (s, 3H, O*Me*), 2.88 (t, *J* = 7.7 Hz, 2H, H_3), 2.72 (t, *J* = 7.7 Hz, 2H, H_2), 2.14 (s, 3H, H_{10}).**^{13}C-NMR (151 MHz, CDCl$_3$)** δ 208.8 (C_1), 157.5 (C_5), 130.0 (C_9), 129.4 (C_4), 127.6 (C_7), 120.6 (C_8), 110.3 (C_6), 55.3 (O*Me*), 43.8 (C_2), 30.0 (C_{10}), 25.1 (C_3). **IR (ATR – neat)** $\tilde{\nu}$ (cm^{-1}) = 3004, 2956, 2933, 2837, 1713, 1602, 1588, 1494, 1465, 1441, 1358, 1241, 1161, 1116, 1035, 753. **HRMS for [$C_{11}H_{14}O_2$]$^+$** calc. 178.0994 found 178.0988. ***R*f** (1:4 EtOAc/PE) = 0.36.

4-(2-(methylthio)phenyl)butan-2-one (5gd)

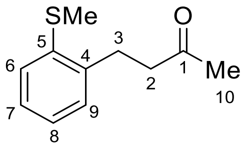

Obtained following **GP (III)** at 0.2 mmol scale using quinuclidin-3-ol (5.0 mg, 40 µmol, 20 mol%) as Lewis base catalyst. Purification by column chromatography on silica gel (3% EtOAc in hexane) afforded product **5gd** (23 mg, 0.12 mmol) as a colourless oil in 60% yield. **^{1}H-NMR (600 MHz, CDCl$_3$)** δ 7.24 – 7.18 (m, 2H, H_6 and H_7), 7.15 (d, *J* = 8.0 Hz, 1H, H_9), 7.09 (ddd, *J* = 8.0, 6.0, 2.6 Hz, 1H; H_8), 2.99 (dd, *J* = 8.6, 6.9 Hz, 2H, H_3), 2.78 (dd, *J* = 8.5, 7.0 Hz, 2H, H_2), 2.47 (s, 3H, S*Me*), 2.16 (s, 3H, H_{10}). **^{13}C-NMR (151 MHz, CDCl$_3$)** δ 208.0

(C_1), 138.7 (C_5), 137.1 (C_4), 129.2 (C_9), 127.0 (C_6), 125.6 (C_7), 125.1 (C_8), 43.5 (C_2), 29.9 (C_3), 27.9 (C_{10}), 15.7 (S*Me*). **IR (ATR – neat)** $\tilde{\nu}\ (cm^{-1})$ = 3060, 2921, 1715, 1590, 1471, 1439, 1358, 1286, 1159, 1068, 967, 957, 747. **HRMS for** $[C_{11}H_{15}OS]^+$ calc. 195.0844 found 195.0838. $\boldsymbol{R_f}$ (1:4 EtOAc/PE) = 0.40.

4-(benzo[d][1,3]dioxol-5-yl)butan-2-one (5hd)

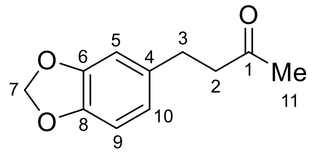

Obtained following **GP (III)** at 0.2 mmol scale using quinuclidin-3-ol (5.0 mg, 40 µmol, 20 mol%) as Lewis base catalyst. Purification by column chromatography on silica gel (5% EtOAc in hexane) afforded product **5hd** (17 mg, 0.088 mmol) as an amorphous solid in 44% yield. ^{1}**H-NMR (600 MHz, CDCl$_3$)** δ 6.71 (d, *J* = 7.9 Hz, 1H, H_9), 6.66 (d, *J* = 1.7 Hz, 1H, H_5), 6.61 (dd, *J* = 8.0, 1.7 Hz, 1H, H_{10}), 5.90 (s, 2H, H_7), 2.80 (t, *J* = 7.6 Hz, 2H, H_3), 2.70 (t, *J* = 7.6 Hz, 2H, H_2), 2.12 (s, 3H, H_{11}). ^{13}**C-NMR (151 MHz, CDCl$_3$)** δ 208.0 (C_1), 147.7 (C_6), 145.9 (C_8), 134.9 (C_4), 121.1 (C_{10}), 108.9 (C_5), 108.3 (C_9), 100.9 (C_7), 45.5 (C_2), 30.2 (C_3), 29.6 (C_{11}). **IR (ATR – neat)** $\tilde{\nu}\ (cm^{-1})$ = 2921, 2897, 2782, 1713, 1504, 1489, 1443, 1360, 1245, 1187, 1159, 1096, 1035, 922, 803, 773.**HRMS for** $[C_{11}H_{12}O_3]^+$ calc. 192.0786 found 192.0782. $\boldsymbol{R_f}$ (1:4 EtOAc/PE) = 0.27.

4-(2,3-dihydrobenzo[b][1,4]dioxin-6-yl)butan-2-one (5id)

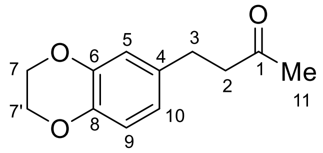

Obtained following **GP (III)** at 0.2 mmol scale using quinuclidin-3-ol (5.0 mg, 40 µmol, 20 mol%) as Lewis base catalyst. Purification by column chromatography on silica gel (5% EtOAc in hexane) afforded product **5id** (27 mg, 0.13 mmol) as an amorphous solid in 65% yield. ^{1}**H-NMR (600 MHz, CDCl$_3$)** δ 6.76 (d, *J* = 8.0 Hz, 1H, H_9), 6.68 (d, *J* = 2.1 Hz, 1H, H_5), 6.64 (dd, *J* = 8.2, 2.2 Hz, 1H, H_{10}), 4.26 – 4.17 (m, 4H, H_7 and $H_{7'}$), 2.78 (t, *J* = 7.5 Hz, 2H, H_3), 2.70 (t, *J* = 7.5 Hz, 2H, H_2), 2.13 (s, 3H, H_{11}). ^{13}**C-NMR (151 MHz, CDCl$_3$)** δ 208.1 (C_1), 143.4 (C_6), 141.9 (C_8), 134.3 (C_4), 121.3 (C_{10}), 117.3 (C_9), 117.0 (C_5), 64.5 (C_7 or $C_{7'}$), 64.4 (C_7 or $C_{7'}$), 45.4 (C_2), 30.2 (C_3), 29.1 (C_{11}). **IR (ATR – neat)** $\tilde{\nu}\ (cm^{-1})$ = 2976, 2925, 2873, 1715, 1590, 1508, 1431, 1364, 1308, 1282, 1259, 1205, 1161, 1126, 1066, 1050, 918, 888, 807. **HRMS for** $[C_{12}H_{14}O_3]^+$ calc. 206.0942 found 206.0934. $\boldsymbol{R_f}$ (1:4 EtOAc/PE) = 0.24.

4-(1H-indol-5-yl)butan-2-one (5jd)

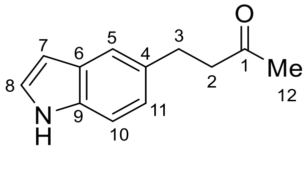

Obtained following **GP (III)** at 0.2 mmol scale using quinuclidin-3-ol (5.0 mg, 40 µmol, 20 mol%) as Lewis base catalyst. Purification by column chromatography on silica gel (15% EtOAc in hexane) afforded product **5jd** (18 mg, 0.096 mmol) as a colourless oil in 48% yield. **^{1}H-NMR (600 MHz, $CDCl_3$)** δ 8.18 (bs, 1H, N*H*), 7.45 (s, 1H, H_5), 7.31 (d, *J* = 8.3 Hz, 1H, H_{10}), 7.18 (t, *J* = 2.8 Hz, 1H, H_8), 7.03 (dd, *J* = 8.3, 1.7 Hz, 1H; H_{11}), 6.50 (ddd, *J* = 3.1, 2.0, 0.9 Hz, 1H, H_7), 3.01 (t, *J* = 7.7 Hz, 2H, H_3), 2.81 (t, *J* = 7.7 Hz, 2H, H_2), 2.15 (s, 3H, H_{12}). **^{13}C-NMR (151 MHz, $CDCl_3$)** δ 208.9 (C_1), 134.6 (C_9), 132.4 (C_4), 128.2 (C_6), 124.6 (C_8), 122.8 (C_{11}), 119.9 (C_5), 111.1 (C_{10}), 102.3 (C_7), 46.3 (C_2), 30.3 (C_{12}), 30.1 (C_3). **IR (ATR – neat)** $\tilde{\nu}$ (cm^{-1}) = 3675, 3400, 2996, 2972, 1707, 1479, 1413, 1362, 1221, 1161, 1066, 729. **HRMS for $[C_{12}H_{13}NO]^+$** calc. 187.0997 found 187.0992. ***R*f** (2:3 EtOAc/PE) = 0.52.

4-(1H-indol-6-yl)butan-2-one (5kd)

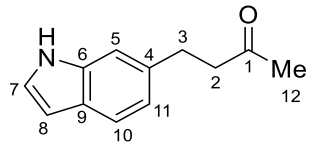

Obtained following **GP (III)** at 0.2 mmol scale using quinuclidin-3-ol (5.0 mg, 40 µmol, 20 mol%) as Lewis base catalyst. Purification by column chromatography on silica gel (15% EtOAc in hexane) afforded product **5kd** (20 mg, 0.11 mmol) as a colourless oil in 55% yield. **^{1}H-NMR (600 MHz, $CDCl_3$)** δ 8.23 (bs, 1H, N*H*), 7.57 (d, *J* = 8.1 Hz, 1H, H_{10}), 7.20 (dt, *J* = 1.6, 0.8 Hz, 1H, H_5), 7.14 (dd, *J* = 3.2, 2.4 Hz, 1H, H_7), 6.97 (dd, *J* = 8.1, 1.5 Hz, 1H, H_{11}), 6.52 (ddd, *J* = 3.1, 2.0, 1.0 Hz, 1H, H_8), 3.02 (t, *J* = 7.7 Hz, 2H, H_3), 2.82 (t, *J* = 7.7 Hz, 2H, H_2), 2.14 (s, 3H, H_{12}). **^{13}C-NMR (151 MHz, $CDCl_3$)** δ 208.8 (C_1), 136.2 (C_6), 134.9 (C_4), 126.3 (C_9), 124.1 (C_7), 120.7 (C_{11}), 120.6 (C_{10}), 110.7 (C_5), 102.4 (C_8), 46.0 (C_3), 30.3 (C_{12}), 30.3 (C_3). **IR (ATR – neat)** $\tilde{\nu}$ (cm^{-1}) = 3400, 2960, 2921, 2853, 1703, 1625, 1510, 1455, 1403, 1346, 1274, 1249, 1161, 1092, 896, 866, 807, 769, 725. **HRMS for $[C_{12}H_{13}NO]^+$** calc. 187.0997 found 187.0992. ***R*f** (2:3 EtOAc/PE) = 0.49.

4-(dibenzo[b,d]thiophen-4-yl)butan-2-one (5ld)

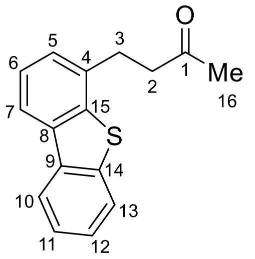

Obtained following **GP (III)** at 0.2 mmol scale using quinuclidin-3-ol (5.0 mg, 40 µmol, 20 mol%) as Lewis base catalyst. Purification by column chromatography on silica gel (10% EtOAc in hexane) afforded product **5ld** (35 mg, 0.14 mmol) as a yellowish solid in 69% yield. **^{1}H-NMR (600 MHz, $CDCl_3$)** δ 8.16 – 8.08 (m, 1H, H_{10}), 8.01 (d, *J* = 7.7 Hz, 1H, H_7), 7.90 – 7.82 (m, 1H, H_{13}), 7.48 – 7.43 (m, 2H, H_{11} and H_{12}), 7.41 (t, *J* = 7.6 Hz, 1H, H_6), 7.28 (d, *J* = 7.2 Hz, 1H, H_5), 3.18 (t, *J* = 7.7 Hz, 2H, H_3), 2.93 (t, *J* = 7.7 Hz, 2H, H_2), 2.17 (s, 3H, H_{16}). **^{13}C-NMR (151 MHz, $CDCl_3$)** δ 207.6 (C_1), 139.0 (C_{14} or C_{15}), 138.9 (C_{15} or C_{14}), 136.2 (C_8 or

C_9), 135.9 (C_9 or C_8), 135.2 (C_4), 126.8 (C_{12}), 126.3 (C_5), 125.0 (C_6), 124.5 (C_{11}), 122.9 (C_{10}), 121.8 (C_{13}), 119.7 (C_7), 42.7 (C_2), 30.1 (C_3), 29.0 (C_{16}). **IR (ATR – neat)** $\tilde{v}$ (cm^{-1}) = 3063, 2905, 1713, 1582, 1443, 1403, 1356, 1161, 1054, 1021, 791, 706. **HRMS for [$C_{16}H_{15}O^{32}S$]$^+$** calc. 255.0838 found 255.0834. ***R*f** (2:3 EtOAc/PE) = 0.60. **M.p.** = 85–87 °C

6-phenylhexan-2-one (5md)

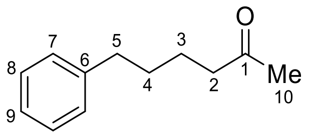

Obtained following **GP (III)** at 0.2 mmol scale using DMAP (4.8 mg, 40 µmol, 20 mol%) as Lewis base catalyst. Purification by column chromatography on silica gel (3% EtOAc in hexane) afforded product **5md** (25 mg, 0.14 mmol) as colourless oil in 70% yield. **^{1}H-NMR (600 MHz, $CDCl_3$)** δ 7.31 – 7.26 (m, 2H, H_8), 7.21 – 7.16 (m, 3H, H_7 and H_9), 2.67 – 2.60 (m, 2H, H_5), 2.48 – 2.42 (m, 2H, H_2), 2.13 (s, 3H, H_{10}), 1.67 – 1.60 (m, 4H, H_2 and H_3). **^{13}C-NMR (151 MHz, $CDCl_3$)** δ 208.9 (C_1), 142.2 (C_6), 128.4 (C_7), 128.3 (C_8), 125.7 (C_9), 43.6 (C_2), 35.7 (C_5), 30.9 (C_{10}), 29.9 (C_4), 23.5 (C_3). **HRMS for [$C_{12}H_{17}NO$]$^+$** calc. 177.1279 found 177.1279. ***R*f** (1:4 EtOAc/PE) = 0.43.

6-methylheptan-2-one (5nd)

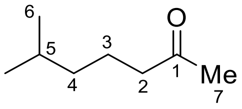

Obtained following **GP (III)** at 0.2 mmol scale using DMAP (4.8 mg, 40 µmol, 20 mol%) as Lewis base catalyst. Purification by column chromatography on silica gel (10% Et_2O in pentane) afforded product **5nd** (15 mg, 0.12 mmol) as colourless oil in 60% yield. **^{1}H-NMR (600 MHz, $CDCl_3$)** δ 2.40 (t, *J* = 7.5 Hz, 2H, H_2), 2.13 (s, 3H, H_7), 1.60 – 1.54 (m, 3H, H_3 and H_5), 1.19 – 1.08 (m, 2H, H_4), 0.87 (d, *J* = 6.6 Hz, 6H, H_6). **^{13}C-NMR (151 MHz, $CDCl_3$)** δ 209.4 (C_1), 44.0 (C_2), 38.4 (C_4), 29.8 (C_5), 27.8 (C_7), 22.5 (C_6), 21.7 (C_3). **HRMS for [$C_8H_{17}O$]$^+$** calc. 129.1274 found 129.1268. ***R*f** (1:4 EtOAc/PE) = 0.43. Spectroscopic data were consistent with literature values.[27]

10-bromodecan-2-one (5od)

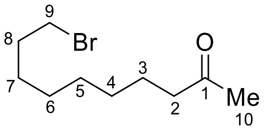

Obtained following **GP (III)** at 0.2 mmol scale using DMAP (4.8 mg, 40 µmol, 20 mol%) as Lewis base catalyst. Filtration on silica gel (5% EtOAc in hexane) afforded product **5od** (33 mg, 0.14 mmol) as colourless oil in 71% yield. **^{1}H-NMR (600 MHz, $CDCl_3$)** δ 3.39 (t, *J* = 6.8 Hz, 2H, H_9), 2.41 (t, *J* = 7.4 Hz, 2H, H_2), 2.12 (s, 3H, H_{10}), 1.84 (p, *J* = 7.0 Hz, 2H, H_8), 1.60 – 1.49 (m, 2H, H_3), 1.44 – 1.38 (m, 2H, H_7), 1.34 – 1.26 (m, 6H, H_4, H_5 and H_6). **^{13}C-NMR (151 MHz, $CDCl_3$)** δ 209.2 (C_1), 43.7 (C_2), 34.0 (C_9), 32.7 (C_8), 29.9 (C_{10}), 29.2 (C_5), 29.0 (C_6), 28.5 (C_4),

28.1 (C_7), 23.7 (C_3). **HRMS for [$C_{10}H_{20}OBr$]$^+$** calc. 235.0698 found 235.0693. ***R*f** (1:4 EtOAc/PE) = 0.44. Spectroscopic data were consistent with literature values.[28]

4-cyclobutylbutan-2-one (5pd)

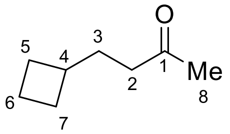

Obtained following **GP (III)** at 0.2 mmol scale using DMAP (4.8 mg, 40 µmol, 20 mol%) as Lewis base catalyst. Purification by column chromatography on silica gel (5% Et_2O in pentane) afforded product **5pd** (17 mg, 0.14 mmol) as colourless oil in 68% yield. **^{1}H-NMR (600 MHz, $CDCl_3$)** δ 2.31 (t, *J* = 7.6 Hz, 2H), 2.25 – 2.16 (m, 1H), 2.11 (s, 3H), 2.05 – 1.97 (m, 2H), 1.80 (dddd, *J* = 23.8, 15.5, 10.0, 5.1 Hz, 2H), 1.64 (q, *J* = 7.6 Hz, 2H), 1.56 (pd, *J* = 8.8, 2.6 Hz, 2H). **^{13}C-NMR (151 MHz, $CDCl_3$)** δ 209.3 (C_1), 41.4 (C_2), 35.4 (C_3), 30.9 (C_8), 29.8 (C_4), 27.9 (C_5 and C_7), 18.2 (C_6). **IR (ATR – neat)** $\tilde{\nu}$ (cm^{-1}) = 2956, 2925, 2857, 1719, 1441, 1411, 1358, 1241, 1169. **HRMS for [$C_8H_{15}O$]$^+$** calc. 127.1117 found 127.1112. ***R*f** (1:4 EtOAc/PE) = 0.45.

4-cyclohexylbutan-2-one (5qd)

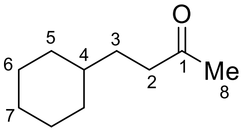

Obtained following **GP (III)** at 0.2 mmol scale using DMAP (4.8 mg, 40 µmol, 20 mol%) as Lewis base catalyst. Filtration on silica gel (10% Et_2O in pentane) afforded product **5qd** (28 mg, 0.18 mmol) as colourless oil in 90% yield. **^{1}H-NMR (600 MHz, $CDCl_3$)** δ 2.43 (t, *J* = 7.8 Hz, 2H, H_2), 2.14 (s, 3H, H_8), 1.72 – 1.63 (m, 5H, H_{5a}, H_{6a} and H_{7a}), 1.50 – 1.43 (m, 2H, H_3), 1.26 – 1.08 (m, 4H, H_4, H_{6b} and H_{7b}), 0.93 – 0.84 (m, 2H, 5_b). **^{13}C-NMR (151 MHz, $CDCl_3$)** δ 209.6 (C_1), 41.4 (C_8), 37.2 (C_4), 33.1 (C_5), 31.2 (C_3), 29.8 (C_8), 26.5 (C_7), 26.2 (C_6). **HRMS for [$C_{10}H_{19}O$]$^+$** calc. 155.1430 found 155.1428. ***R*f** (1:4 EtOAc/PE) = 0.51. Spectroscopic data were consistent with literature values.[29]

***tert*-butyl 2-(3-oxobutyl)pyrrolidine-1-carboxylate (5rd)**

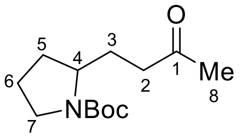

Obtained following **GP (III)** at 0.2 mmol scale using DMAP (4.8 mg, 40 µmol, 20 mol%) as Lewis base catalyst. Purification by column chromatography on silica gel (10% EtOAc in hexane) afforded product **5rd** (38 mg, 0.16 mmol) as colourless oil in 80% yield. **^{1}H-NMR (600 MHz, $CDCl_3$)** δ 3.93 – 3.59 (m, 1H, H_4), 3.46 – 3.22 (m, 2H, H_7), 2.51 – 2.32 (m, 2H, H_2), 2.11 (s, 3H, H_8), 1.92 – 1.71 (m, 4H, H_{3a}, H_{5a} and H_6), 1.64 – 1.52 (m, 2H, H_{3b} and H_{5b}), 1.42 (s, 9H, N*Boc*). **^{13}C-NMR (151 MHz, $CDCl_3$)** δ 208.5 (C_1), 154.8 (*C*OOC($CH_3)_3$), 79.1 (COO*C*($CH_3)_3$), 56.5 (C_4), 46.2 (C_7), 40.6 (C_2), 30.5 (C_5), 29.8 (C_8), 28.6 (C_3), 28.5

(COOC(*C*H_3)$_3$), 23.3 (C_6).**IR (ATR – neat)** $\tilde{\nu}$ (cm^{-1}) = 2968, 2933, 2873, 1715, 1685, 1391, 1364, 1253, 1167, 1102, 1124, 939, 864, 775. **HRMS for [$C_{13}H_{24}NO_3$]$^+$** calc. 242.1756 found 242.1749. ***R*f** (1:4 EtOAc/PE) = 0.20.

2,5-dichloro-N-(2-((2-methyl-7-oxooctan-4-yl)amino)-2-oxoethyl)benzamide (5sd)

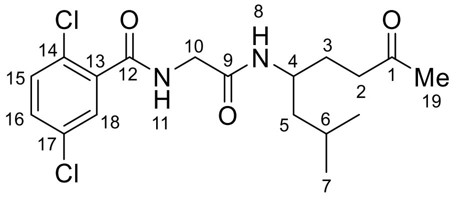

Obtained following **GP (III)** at 0.2 mmol scale using DMAP (4.8 mg, 40 µmol, 20 mol%) as Lewis base catalyst. Purification by column chromatography on silica gel (50% EtOAc in hexane) afforded product **5sd** (61 mg, 0.158 mmol) as yellowish oil in 79% yield. **^{1}H-NMR (600 MHz, CDCl$_3$)** δ 7.61 (bt, *J* = 1.6 Hz, 1H, H_{18}), 7.36 – 7.34 (m, 3H, H_{11}, H_{15} and H_{16}), 6.43 (d, *J* = 9.2 Hz, 1H, H_8), 4.17 – 4.07 (m, 2H, H_{10}), 3.98 (qt, *J* = 9.2, 4.6 Hz, 1H, H_4), 2.57 – 2.44 (m, 2H, H_2), 2.11 (s, 3H, H_{19}), 1.85 – 1.74 (m, 1H, H_{3a}), 1.65 – 1.54 (m, 2H, H_{3b} and H_6), 1.35 (ddd, *J* = 14.5, 9.1, 5.6 Hz, 1H, H_{5a}), 1.29 – 1.23 (m, 1H, H_{5b}), 0.89 (d, *J* = 6.6 Hz, 3H, H_{7a}), 0.88 (d, *J* = 6.6 Hz, 3H, H_{7b}). **^{13}C-NMR (151 MHz, CDCl$_3$)** δ 208.8 (C_4), 167.8 (C_9), 165.4 (C_{12}), 135.5 (C_{13}), 133.2 (C_{14}), 131.6 (C_{15} or C_{16}), 131.5 (C_{16} or C_{15}), 129.9 (C_{18}), 129.2 (C_{17}), 47.6 (C_4), 44.7 (C_5), 43.8 (C_{10}), 40.1 (C_2), 30.1 (C_{19}), 29.3 (C_3), 24.9 (C_6), 23.0 (C_{7a}), 22.1 (C_{7b}).**IR (ATR – neat)** $\tilde{\nu}$ (cm^{-1}) = 3298, 3075, 2952, 2929, 2869, 1711, 1643, 1530, 1463, 1368, 1292, 1257, 1167, 1098, 1046, 912, 819, 729. **HRMS for [$C_{18}H_{25}N_2O_3Cl_2$]$^+$** calc. 387.1242 found 387.1237. ***R*f** (1:1 EtOAc/PE) = 0.19.

6 References

[1] W. D. Wang, J. H. Espenson, *J. Am. Chem. Soc.* **1998**, *120*, 11335–11341.

[2] A. L. Korich, P. M. Iovine, *Dalt. Trans.* **2010**, *39*, 1423–1431.

[3] C. Bomio, M. A. Kabeshov, A. R. Lit, S.-H. Lau, J. Ehlert, C. Battilocchio, S. V Ley, *Chem. Sci.* **2017**, *8*, 6071–6075.

[4] F. Lima, M. A. Kabeshov, D. N. Tran, C. Battilocchio, J. Sedelmeier, G. Sedelmeier, B. Schenkel, S. V. Ley, *Angew. Chem. Int. Ed.* **2016**, *55*, 14085–14089.

[5] H. G. Roth, N. A. Romero, D. A. Nicewicz, *Synlett* **2016**, *27*, 714–723.

[6] B. A. Sim, D. Griller, D. D. M. Wayner, *J. Am. Chem. Soc.* **1989**, *111*, 754–755.

[7] K. Teegardin, J. I. Day, J. Chan, J. Weaver, *Org. Process Res. Dev.* **2016**, *20*, 1156–1163.

[8] S. Pal, S. Chowdhury, E. Rozwadowski, A. Auffrant, C. Gosmini, *Adv. Synth. Catal.* **2016**, *358*, 2431–2435.

[9] M. Itoh, K. Hirano, T. Satoh, M. Miura, *Org. Lett.* **2014**, *16*, 2050–2053.

[10] R. K. Dieter, S. Li, *J. Org. Chem.* **1997**, *62*, 7726–7735.

[11] N. El Achi, M. Penhoat, Y. Bakkour, C. Rolando, L. Chausset-Boissarie, *European J. Org. Chem.* **2016**, *2016*, 4284–4288.

[12] X. J. Dai, H. Wang, C. J. Li, *Angew. Chemie - Int. Ed.* **2017**, *56*, 6302–6306.

[13] E. E. Finney, K. A. Ogawa, A. J. Boydston, *J. Am. Chem. Soc.* **2012**, *134*, 12374–12377.

[14] M. Shimogaki, M. Fujita, T. Sugimura, *Angew. Chemie - Int. Ed.* **2016**, *55*, 15797–15801.

[15] G. C. Tsui, M. Lautens, *Angew. Chemie - Int. Ed.* **2010**, *49*, 8938–8941.

[16] M. H. Wang, D. T. Cohen, C. B. Schwamb, R. K. Mishra, K. A. Scheidt, *J. Am. Chem. Soc.* **2015**, *137*, 5891–5894.

[17] H. Jiang, W. Yang, H. Chen, J. Li, W. Wu, *Chem. Commun.* **2014**, *50*, 7202–4.

[18] K. M. M. Huihui, J. A. Caputo, Z. Melchor, A. M. Olivares, A. M. Spiewak, K. A. Johnson, T. A. Dibenedetto, S. Kim, L. K. G. Ackerman, D. J. Weix, *J. Am. Chem. Soc.* **2016**, *138*, 5016–5019.

[19] F. Berthiol, H. Doucet, M. Santelli, *Synthesis (Stuttg).* **2005**, 3589–3602.

[20] N. Kise, T. Mano, T. Sakurai, *J. Org. Chem.* **1994**, *62*, 1407–1413.

[21] F. Chen, B. Mudryk, T. Cohen, *Tetrahedron* **1999**, *55*, 3291–3304.

[22] K. Miyazawa, T. Koike, M. Akita, *Adv. Synth. Catal.* **2014**, *356*, 2749–2755.

[23] R. K. Dieter, C. W. Alexander, L. E. Nice, *Tetrahedron* **2000**, *56*, 2767–2778.

[24] K. Adachi, *Nippon Kagaku Kaishi* **1972**, *1972*, 985–987.

[25] P. Wipf, W. Xu, J. H. Smitrovich, R. Lehmann, L. M. Venanzi, *Tetrahedron* **1994**, *50*, 1935–1954.

[26] W. . Kerr, R. . Mudd, J. . Brown, *Chem. - A Eur. J.* **2016**, *22*, 4738.

[27] M. B. Reardon, M. Xu, Q. Tan, P. G. Baumgartel, D. J. Augur, S. Huo, C. E. Jakobsche, *J. Org. Chem.* **2016**, *81*, 10964–10974.

[28] D. A. Chaudhari, R. A. Fernandes, *J. Org. Chem.* **2016**, *81*, 2113–2121.

[29] V. Corcé, L. M. Chamoreau, E. Derat, J. P. Goddard, C. Ollivier, L. Fensterbank, *Angew. Chemie Int. Ed.* **2015**, *54*, 11414–11418.

7 NMR spectra

7.1 Starting Materials

7.1.1 Alkene Starting Materials

2-(1-phenylvinyl) pyridine (2o)

^{1}H-NMR (400 MHz, $CDCl_3$)

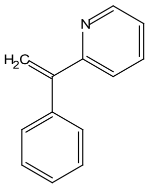

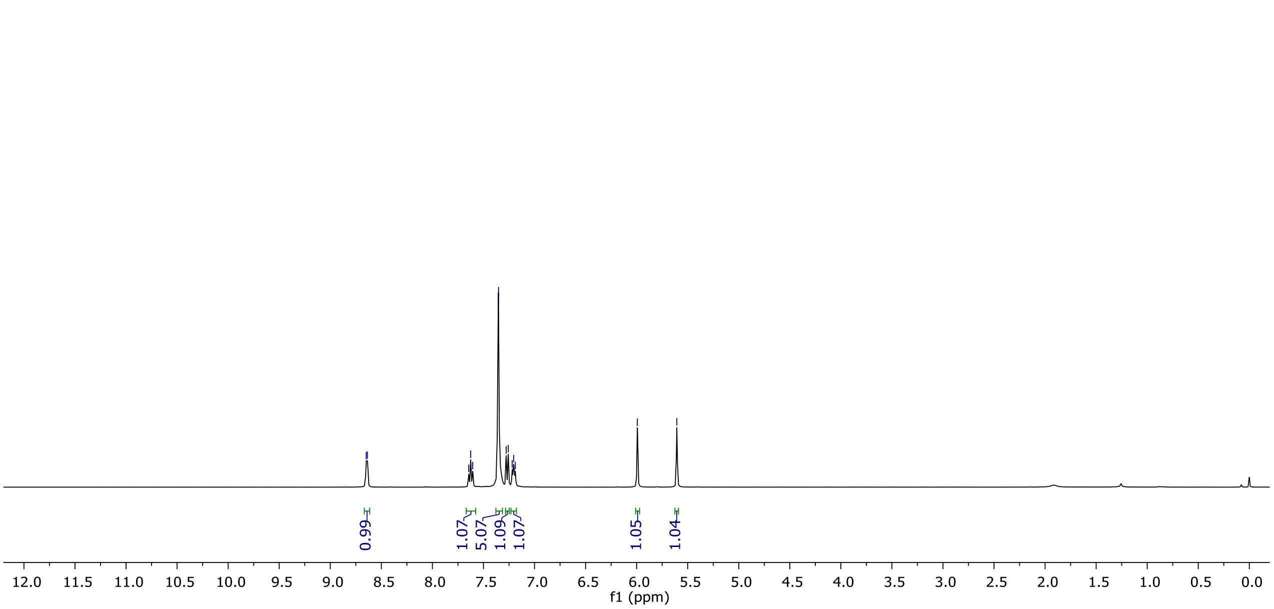

^{13}C-NMR (151 MHz, $CDCl_3$)

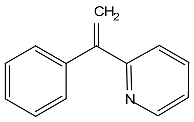

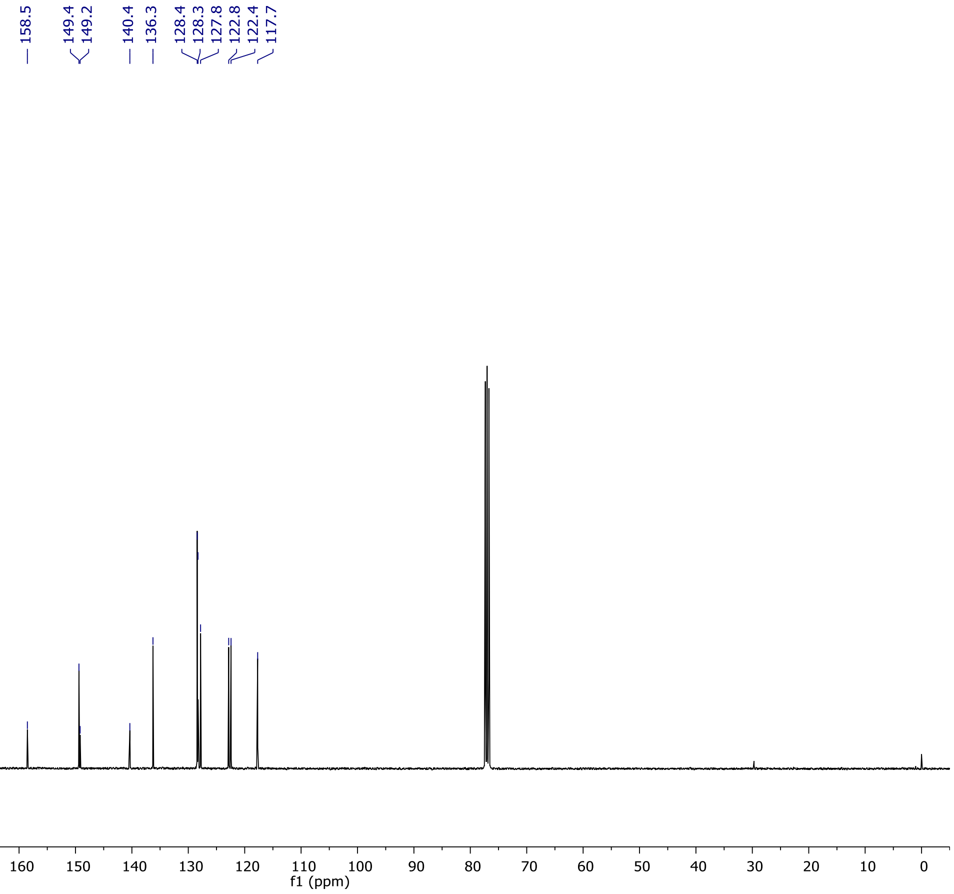

7.1.2 Boronate Starting Materials

***tert*-butyl dimethylcarbamate**

^{1}H-NMR (600 MHz, $CDCl_3$)

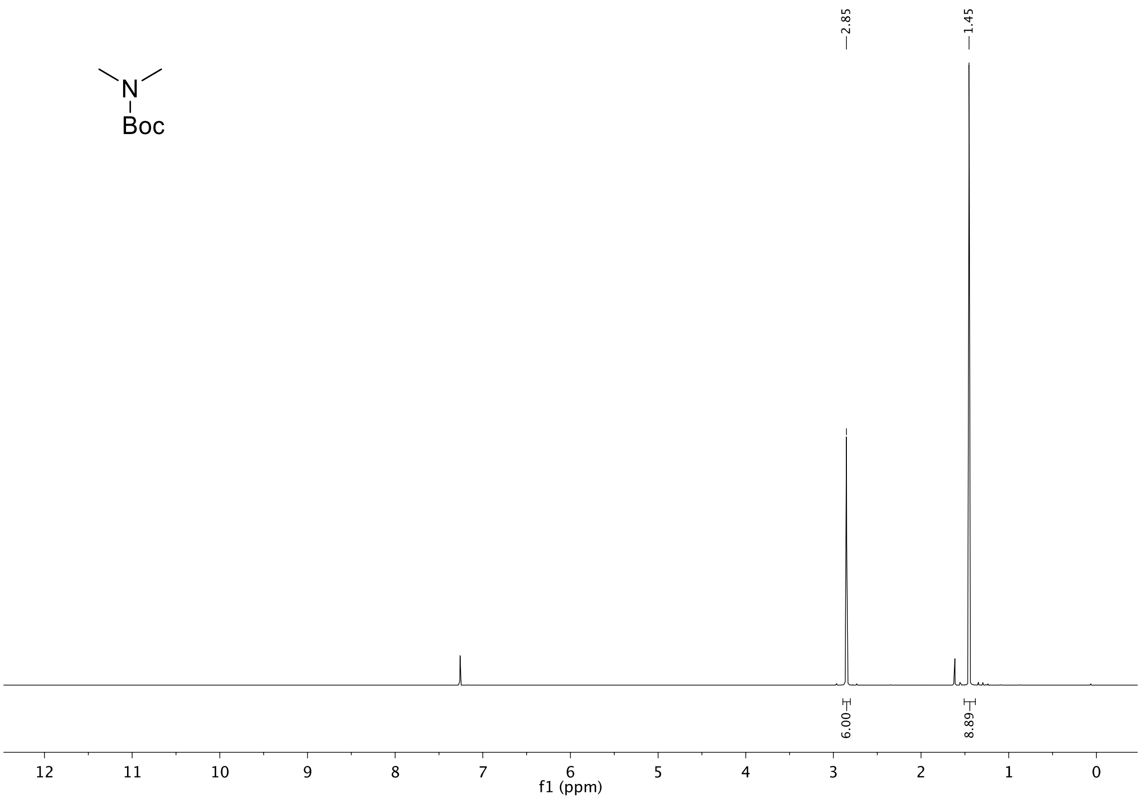

^{13}C-NMR (151 MHz, $CDCl_3$)

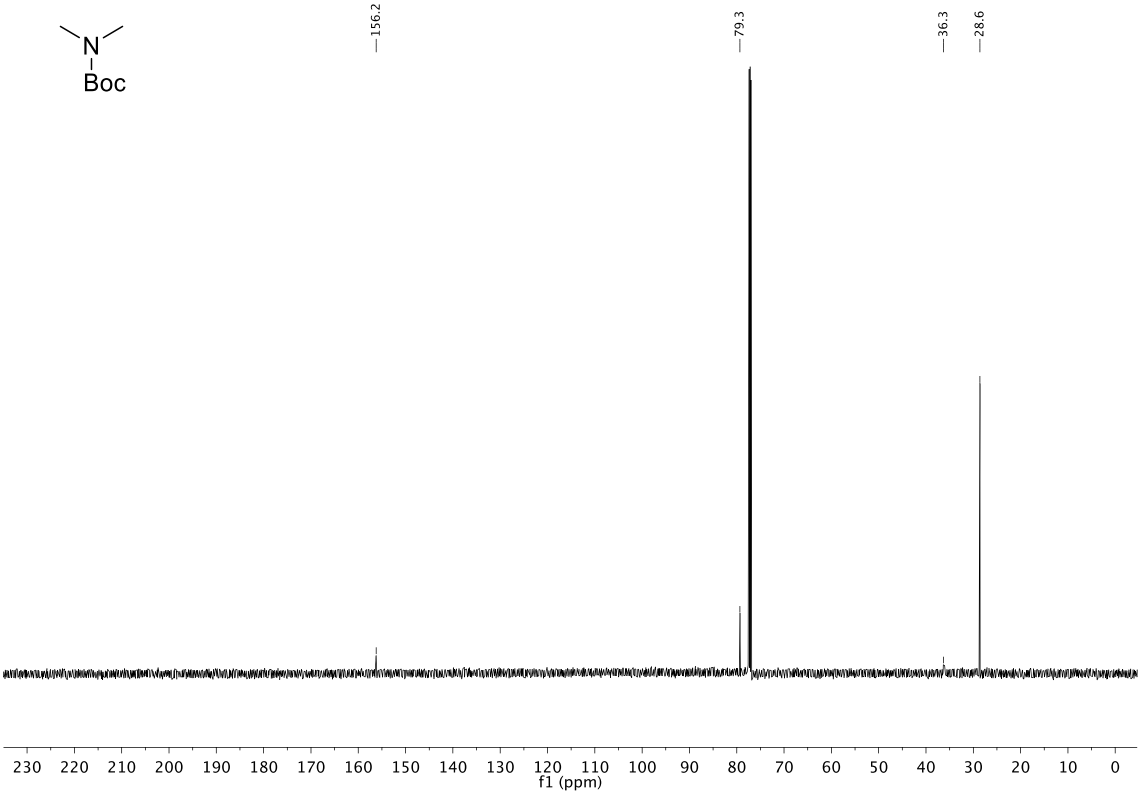

tert-butyl methyl((4,4,5,5-tetramethyl-1,3,2-dioxaborolan-2-yl)methyl)carbamate (1h)

^{1}H-NMR (600 MHz, $CDCl_3$)

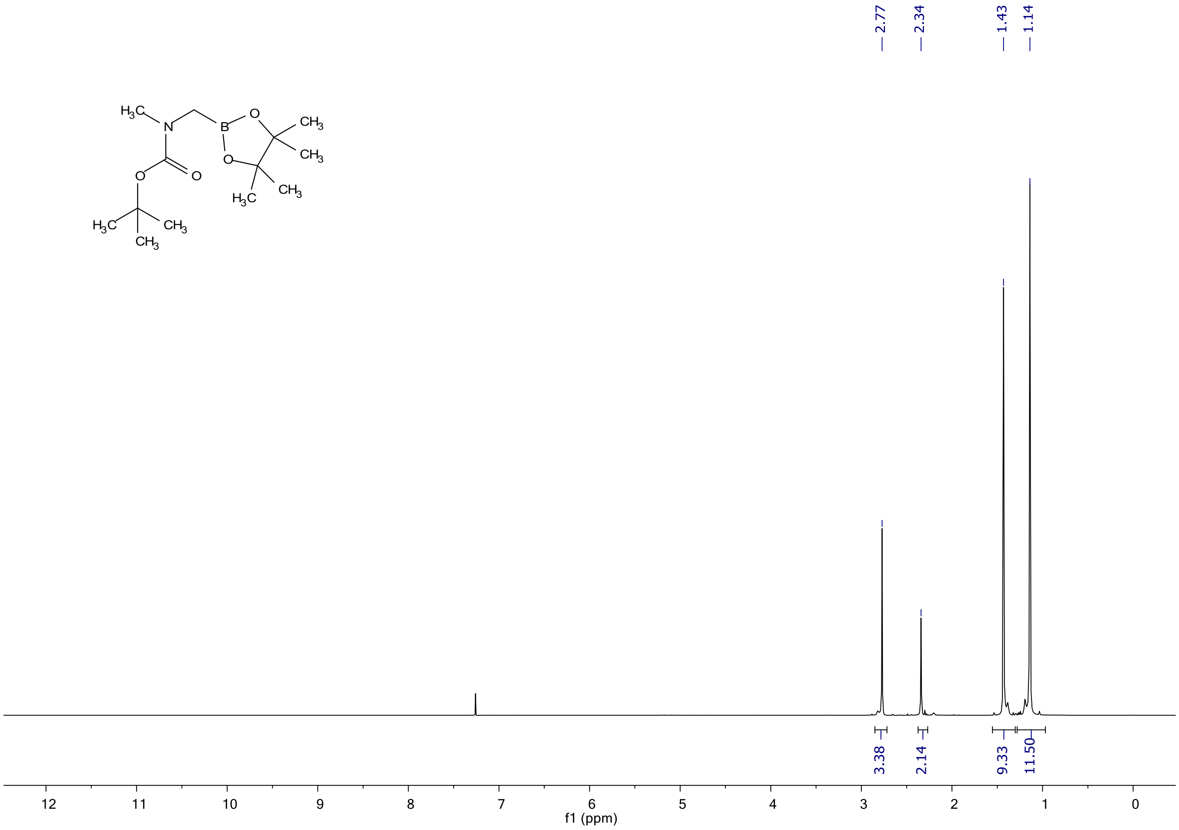

^{13}C-NMR (151 MHz, $CDCl_3$)

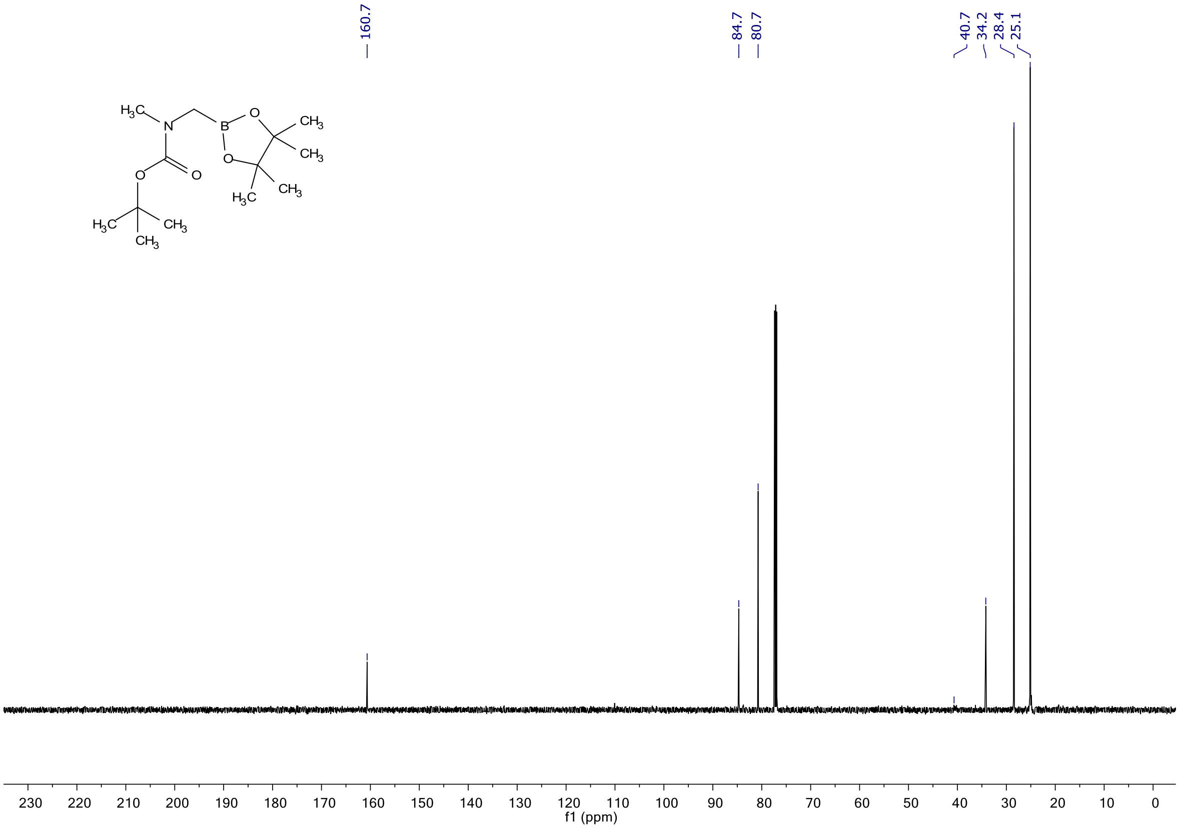

^{11}B-NMR (193 MHz, $CDCl_3$)

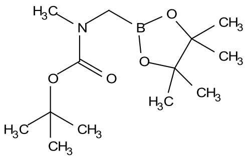

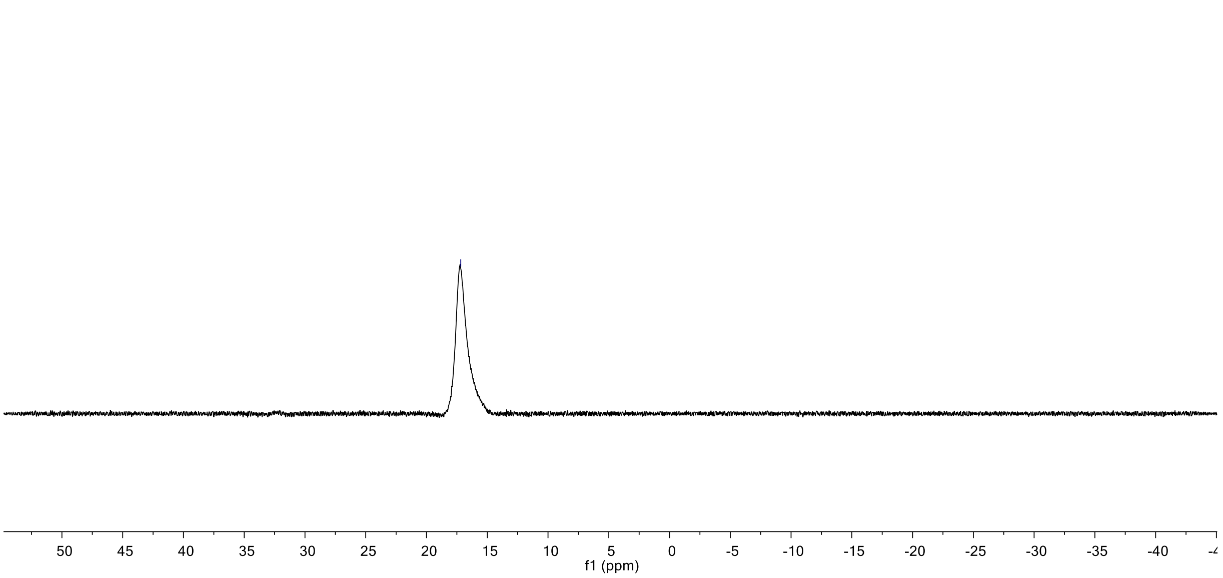

tert-butyl ((4,4,5,5-tetramethyl-1,3,2-dioxaborolan-2-yl)methyl)carbamate (1g)

^{1}H-NMR (600 MHz, $CDCl_3$)

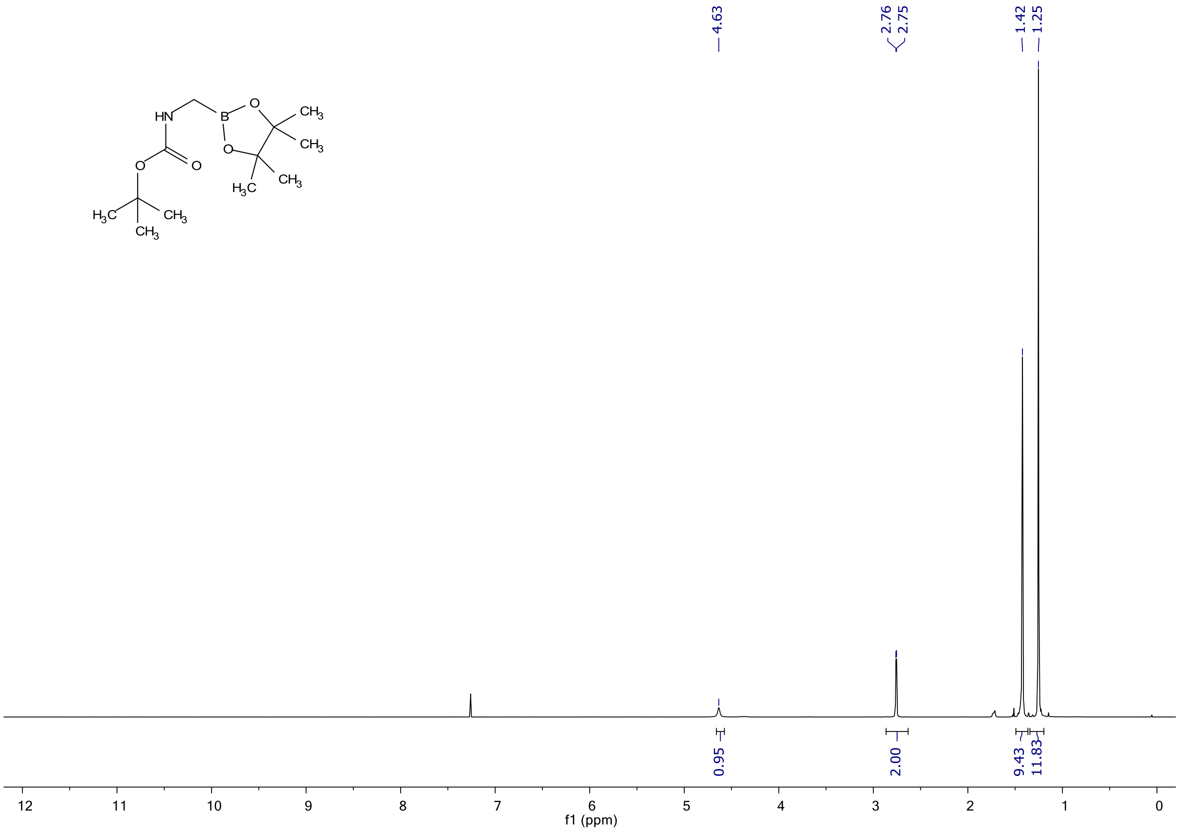

^{13}C-NMR (151 MHz, $CDCl_3$)

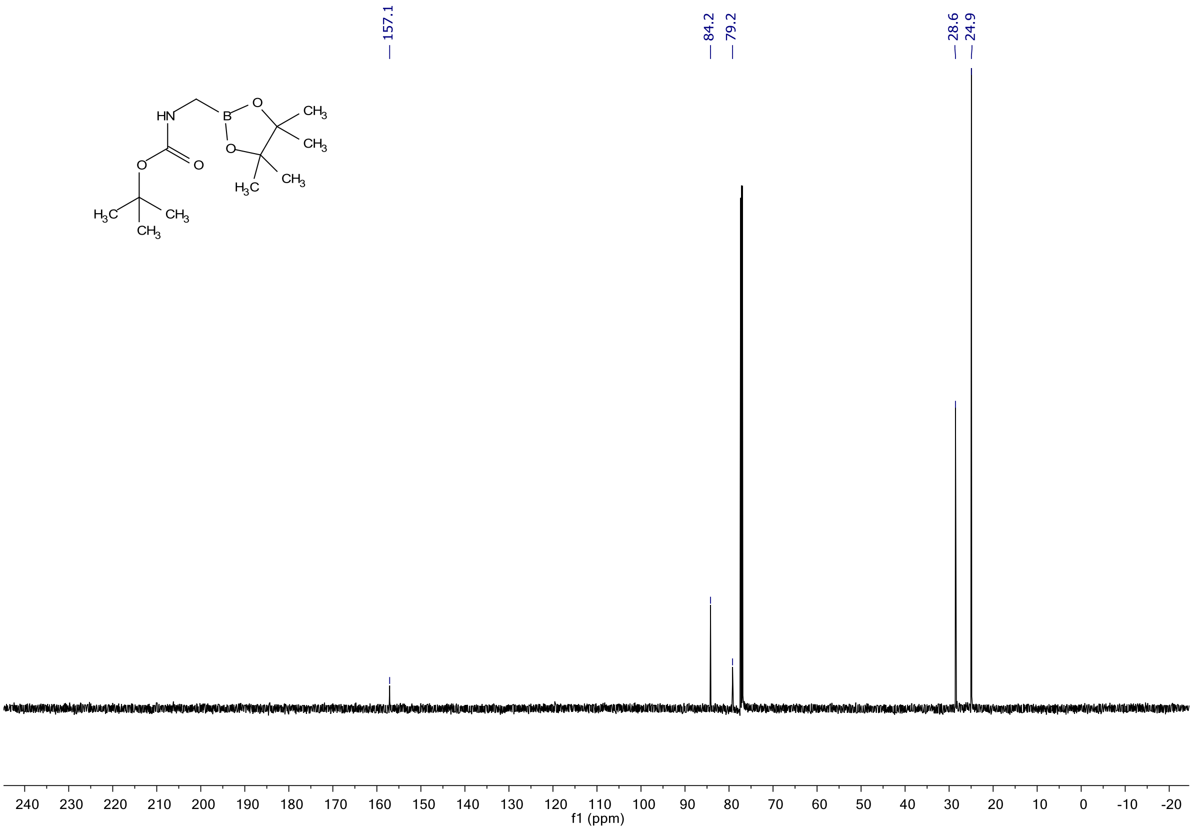

^{11}B-NMR (193 MHz, $CDCl_3$)

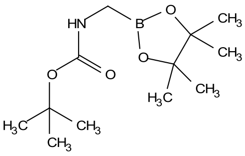

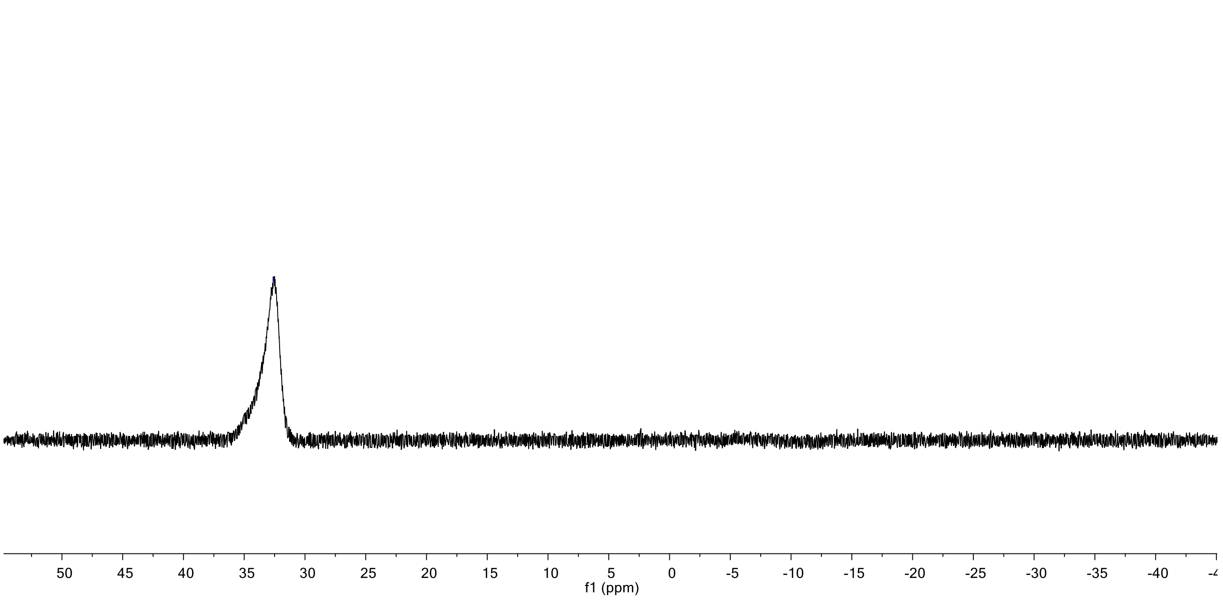

7.2 Coupling Products

7.2.1 Scope of electron-deficient alkenes

methyl 4-(4-methoxyphenyl)butanoate (3aa)

^{1}H-NMR (600 MHz, $CDCl_3$)

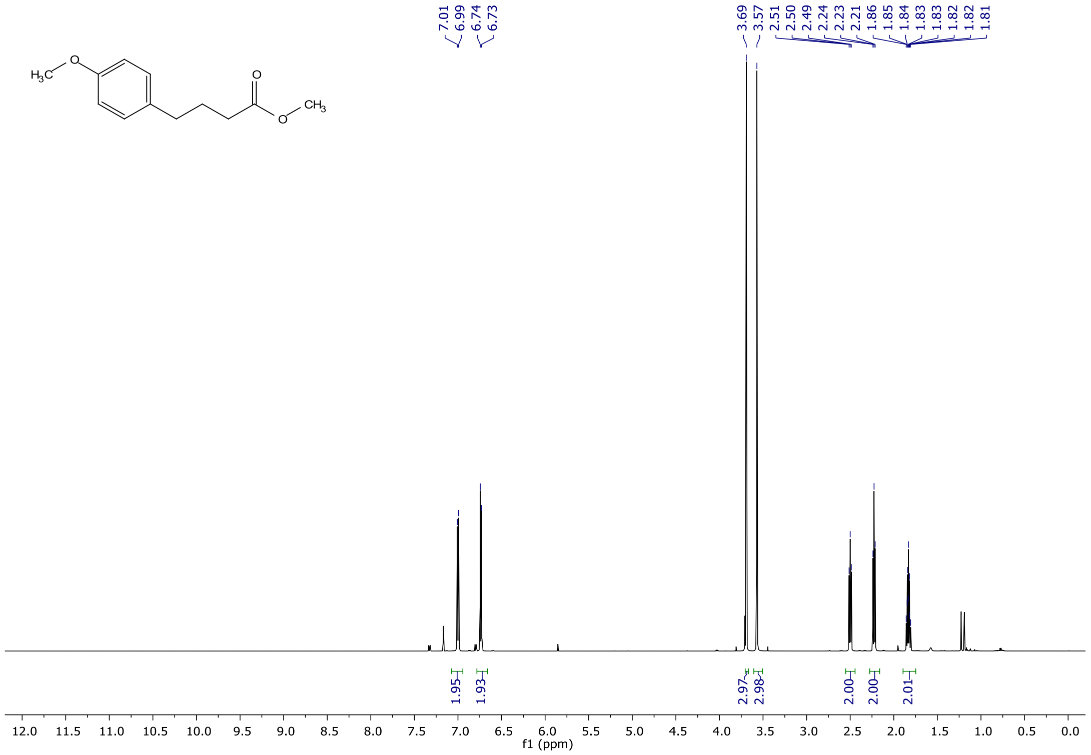

^{13}C-NMR (151 MHz, $CDCl_3$)

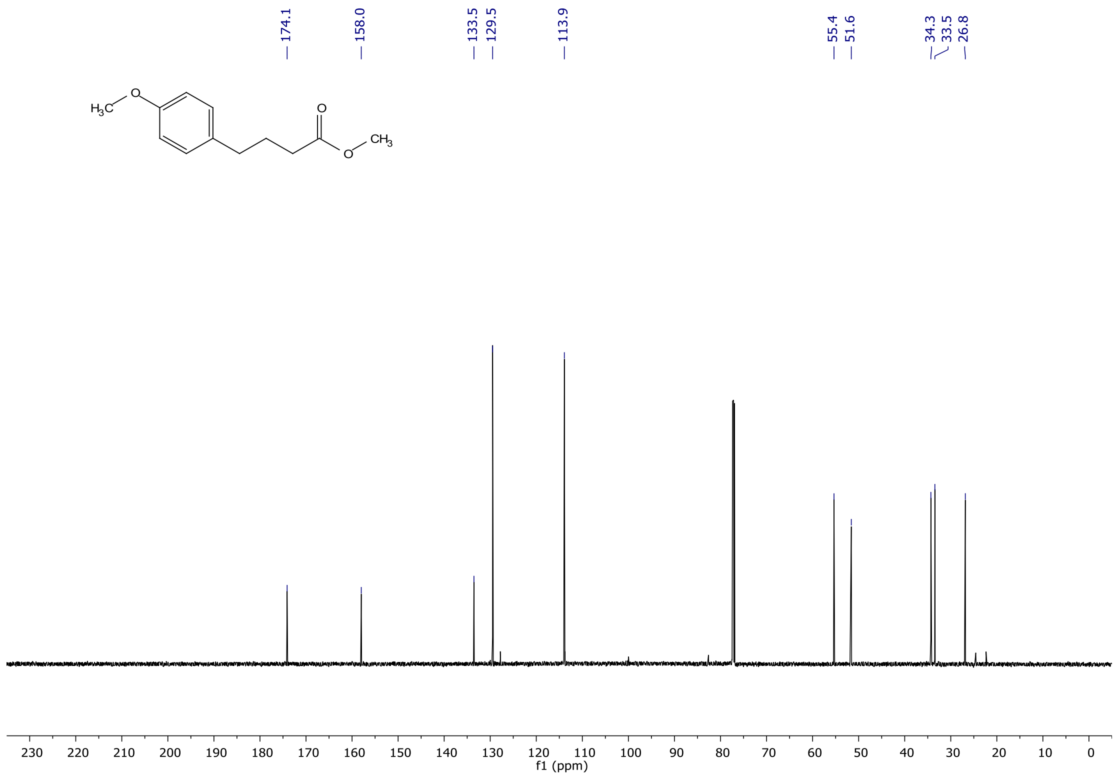

tert-butyl 4-(4-methoxyphenyl)butanoate (3ab)

^{1}H-NMR (400 MHz, $CDCl_3$)

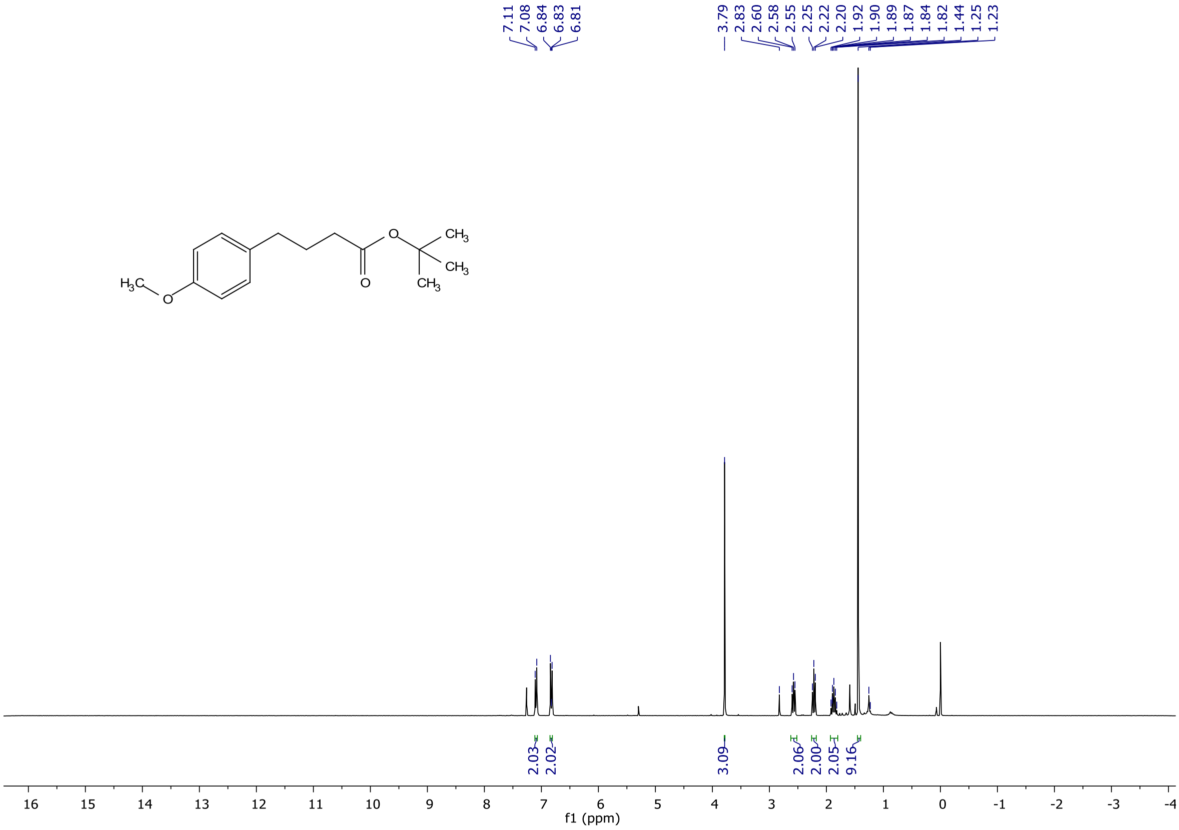

^{13}C-NMR (101 MHz, $CDCl_3$)

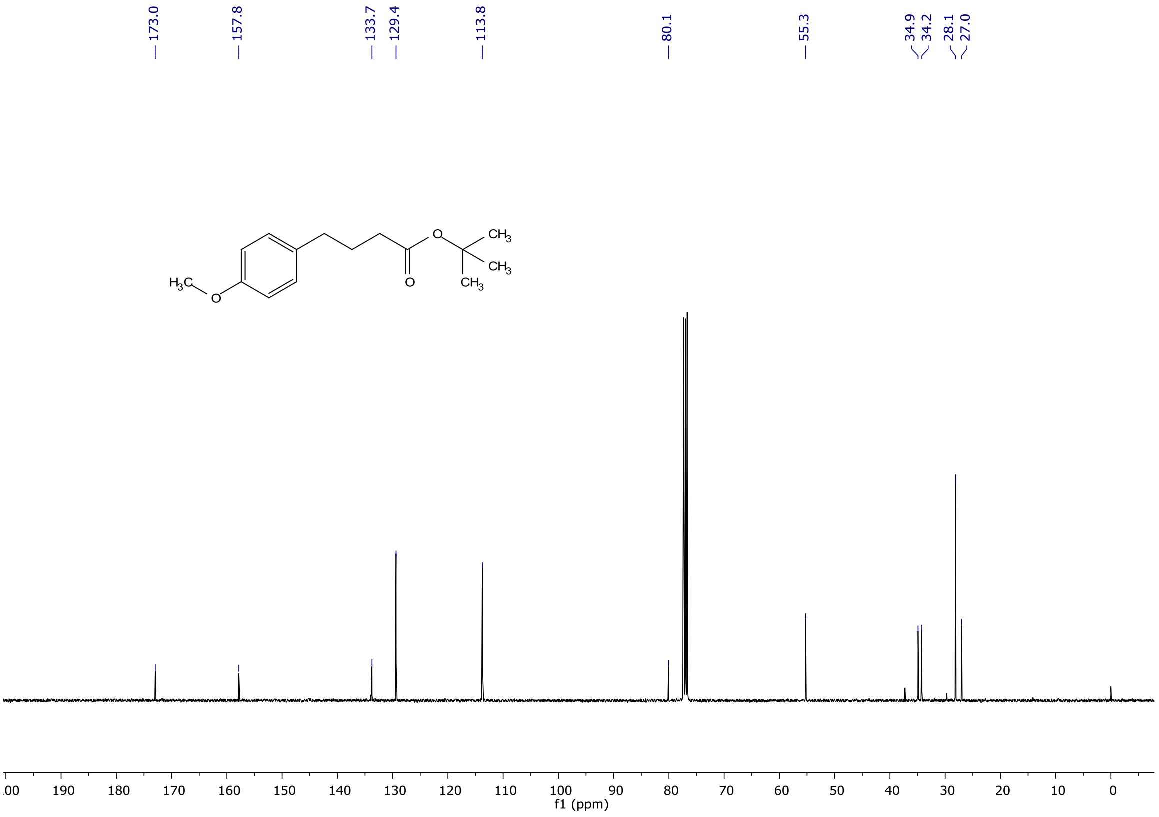

benzyl 4-(4-methoxyphenyl)butanoate (3ac)

^{1}H-NMR (400 MHz, $CDCl_3$)

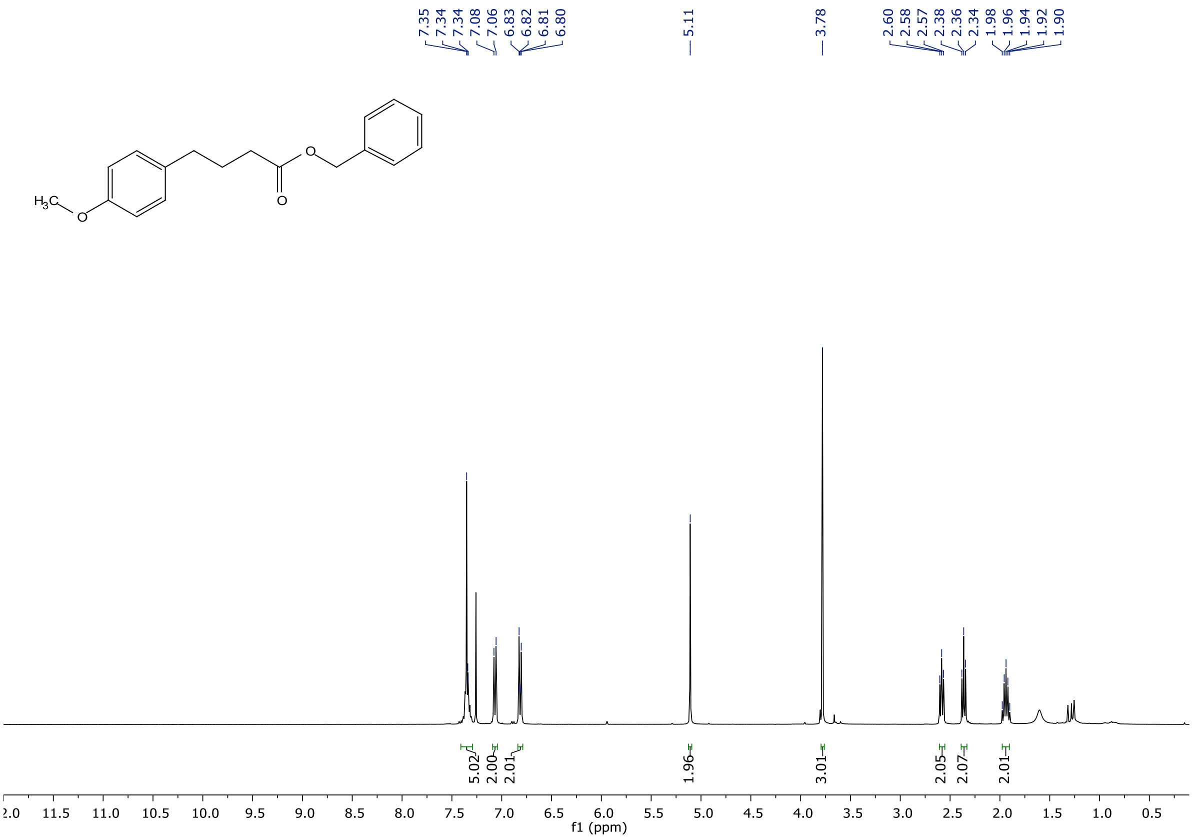

^{13}C-NMR (101 MHz, $CDCl_3$)

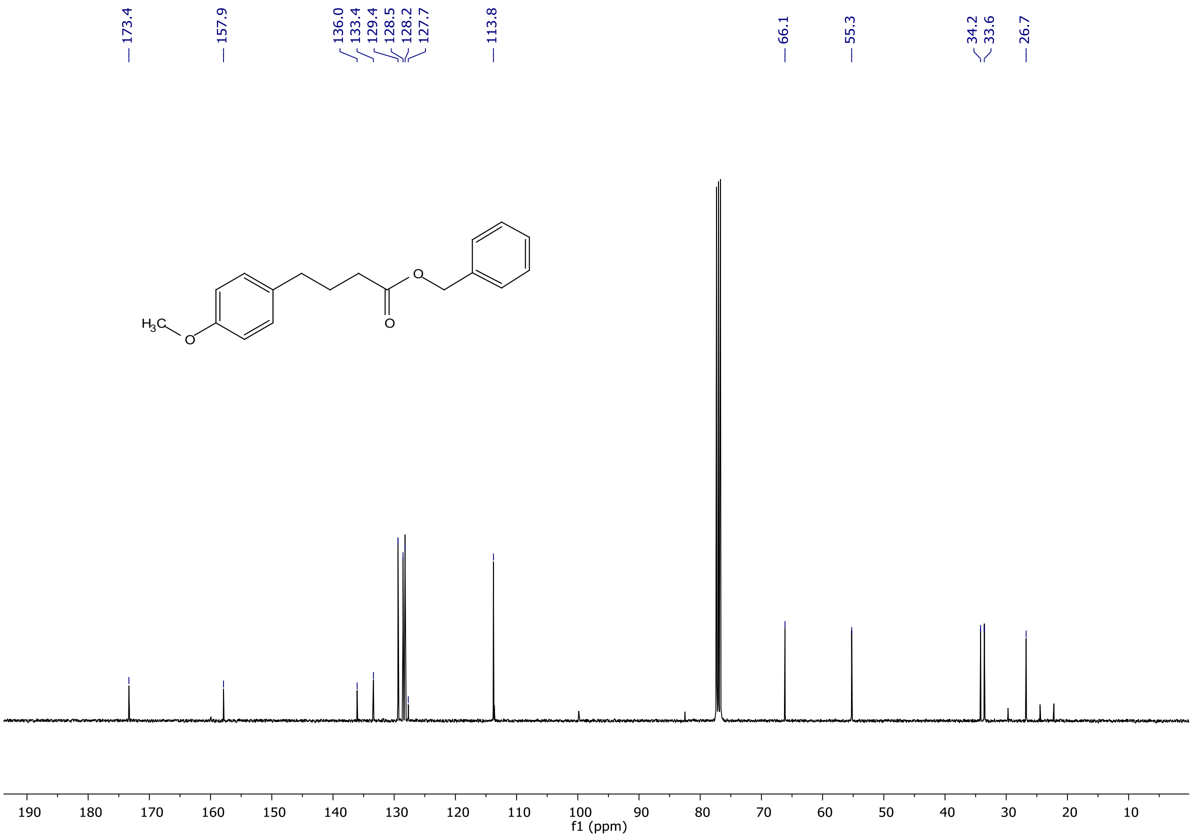

5-(4-methoxyphenyl)pentan-2-one (3ad)

^{1}H-NMR (300 MHz, $CDCl_3$)

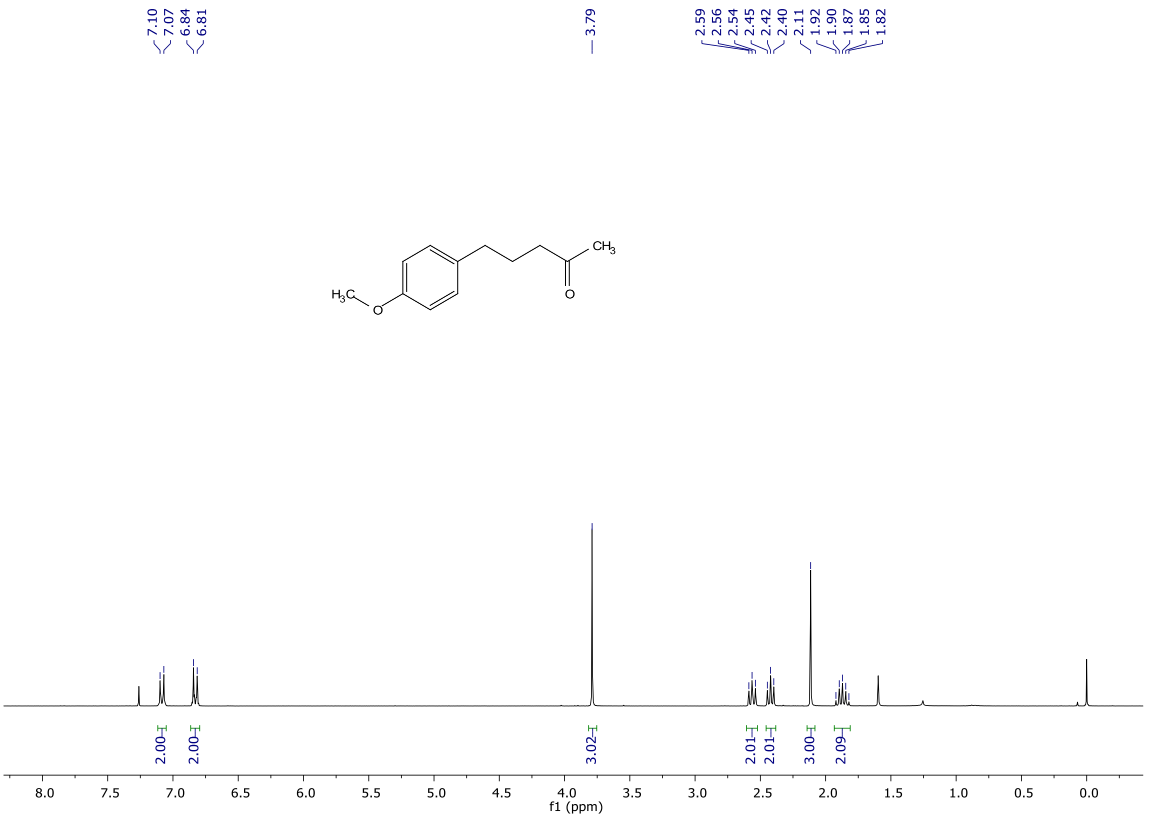

^{13}C-NMR (151 MHz, $CDCl_3$)

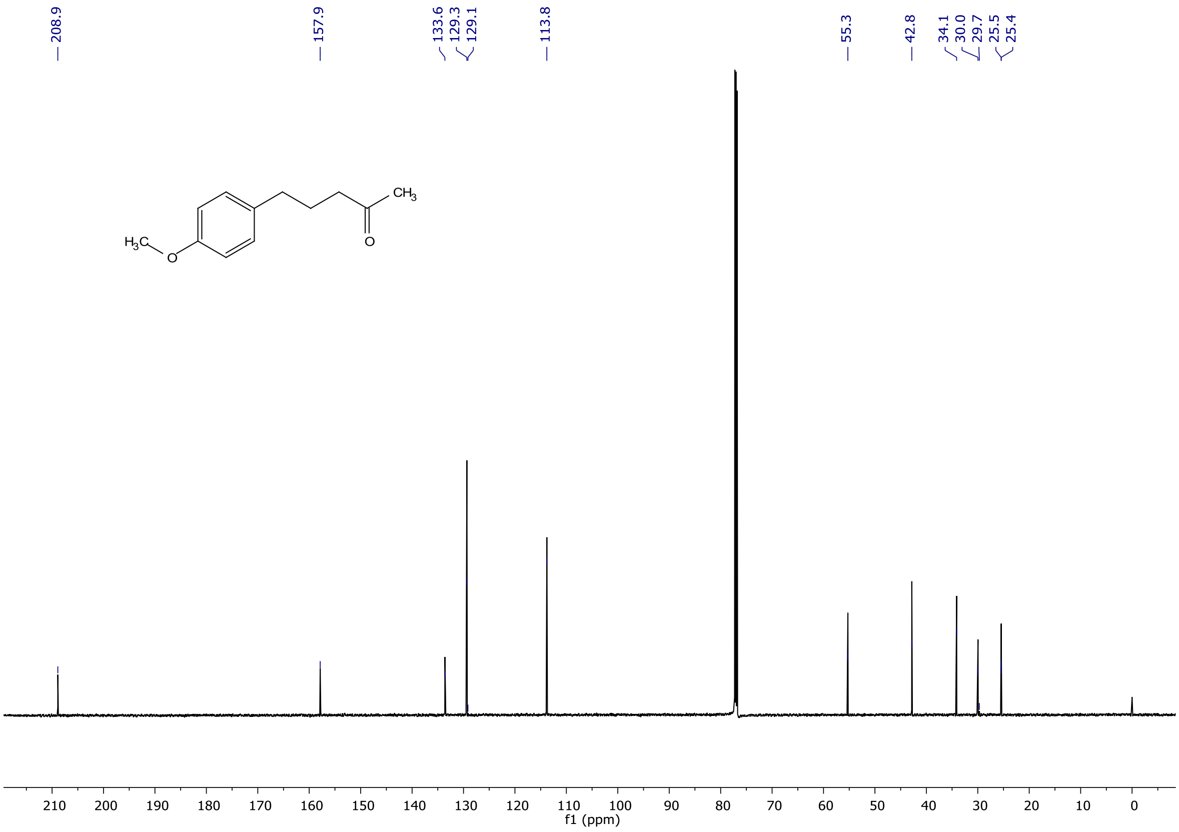

4-(4-methoxyphenyl)butanal (3ae)

^{1}H-NMR (400 MHz, $CDCl_3$)

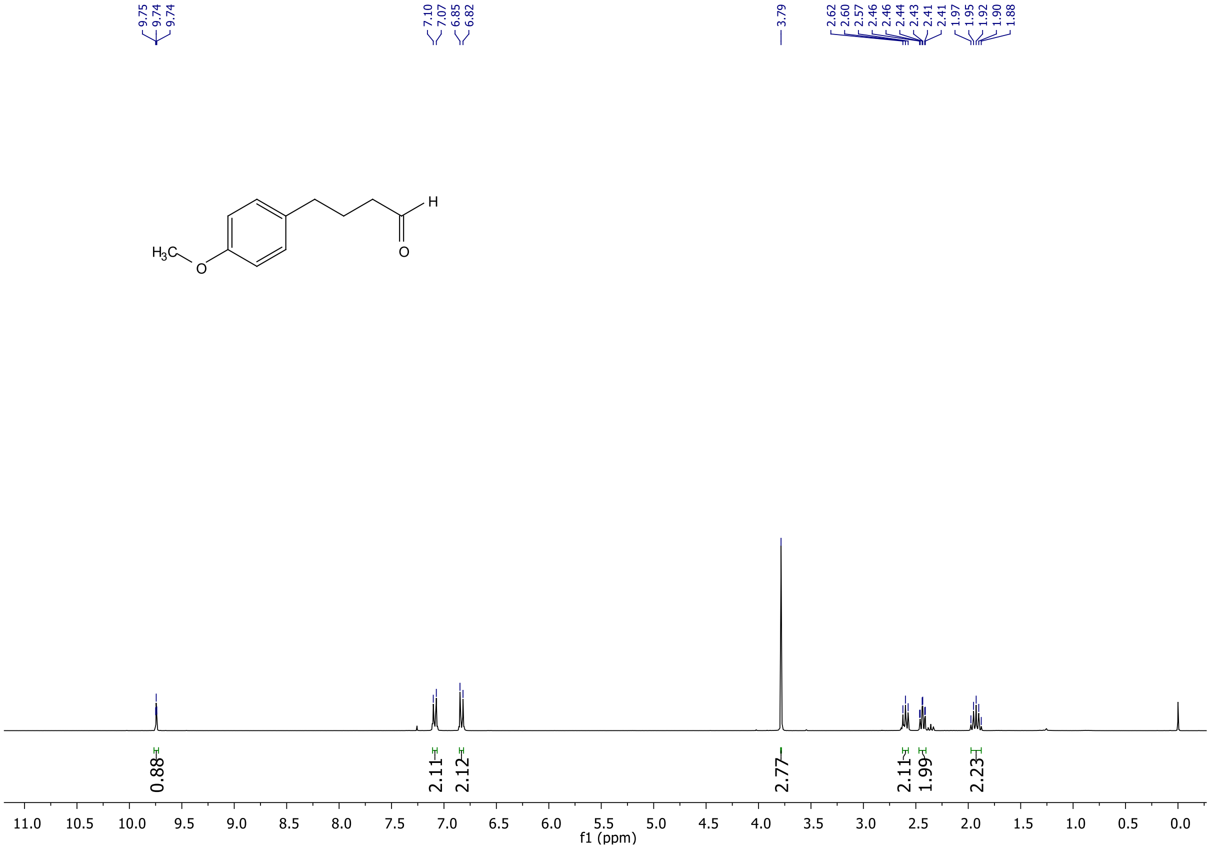

^{13}C-NMR (101 MHz, $CDCl_3$)

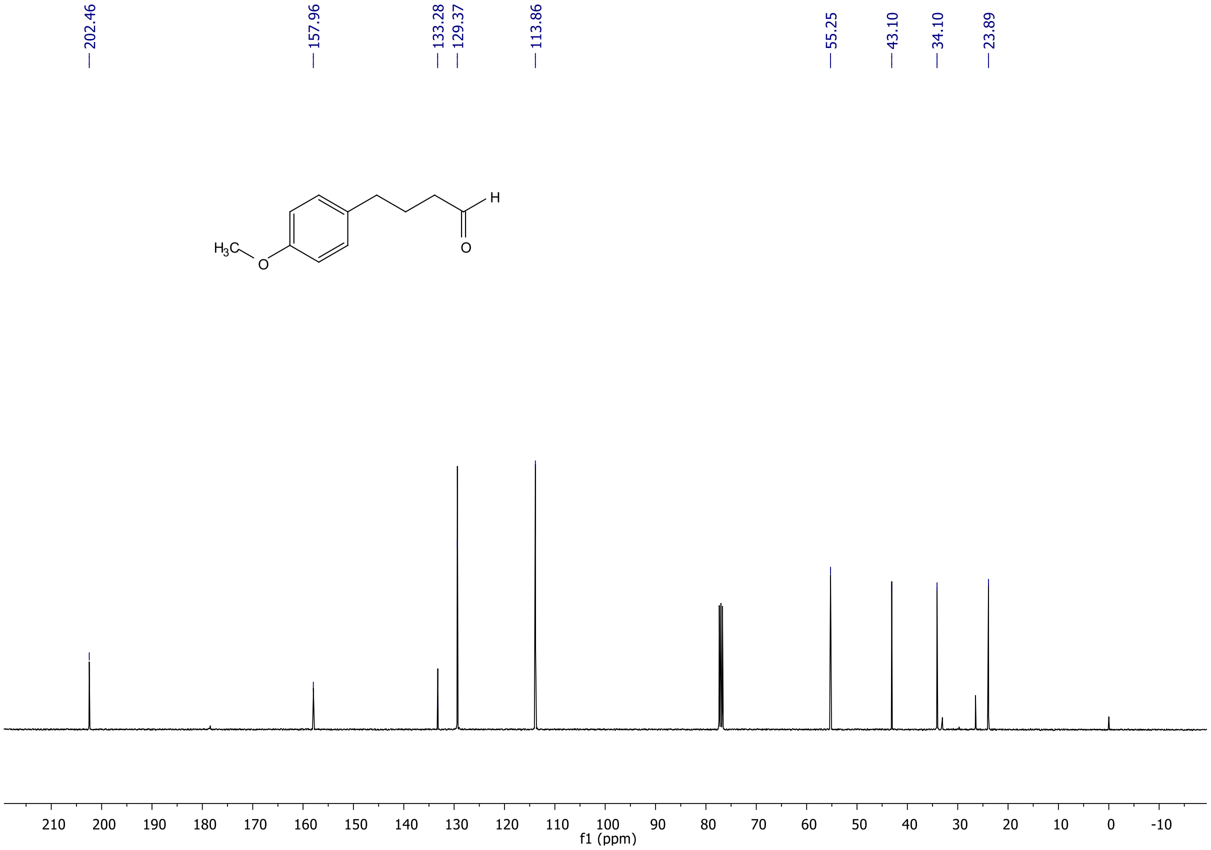

4-(4-methoxyphenyl)butanenitrile (3af)

^{1}H-NMR (400 MHz, $CDCl_3$)

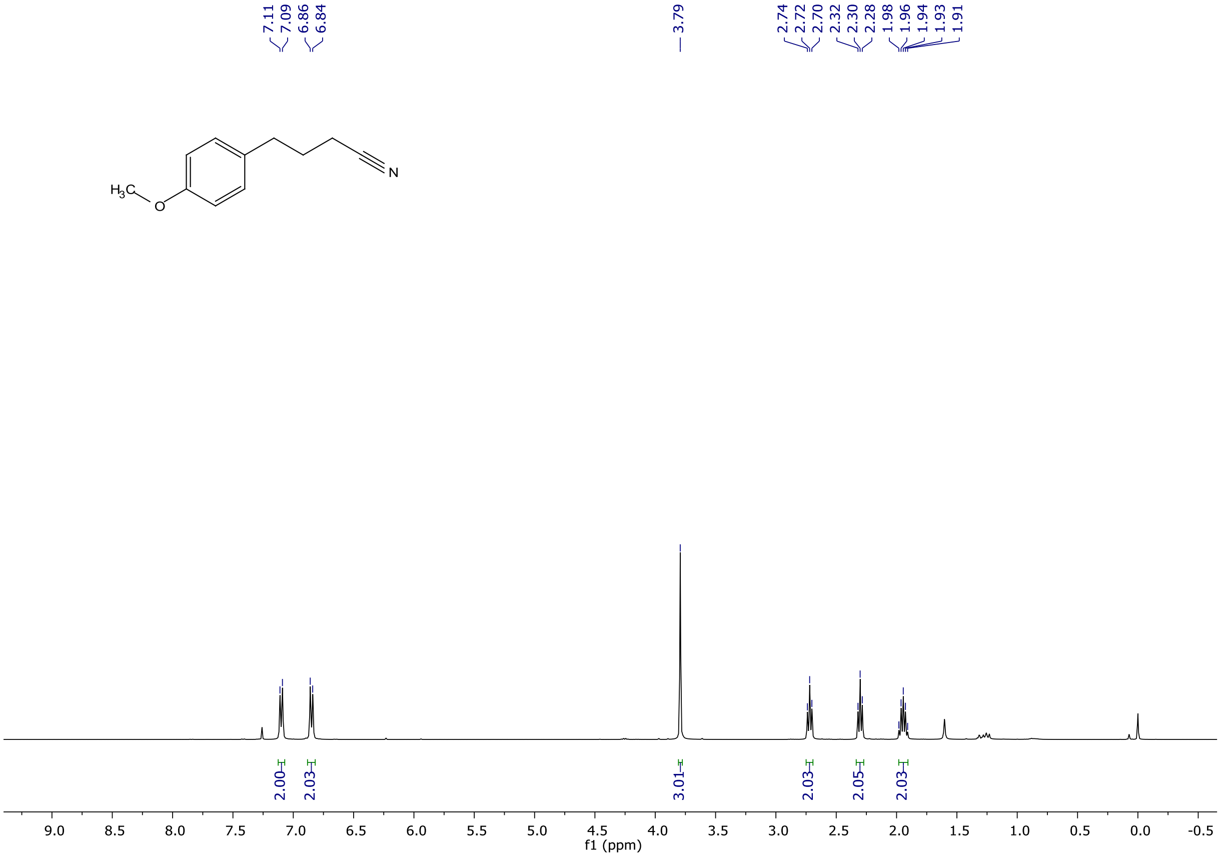

^{13}C-NMR (101 MHz, $CDCl_3$)

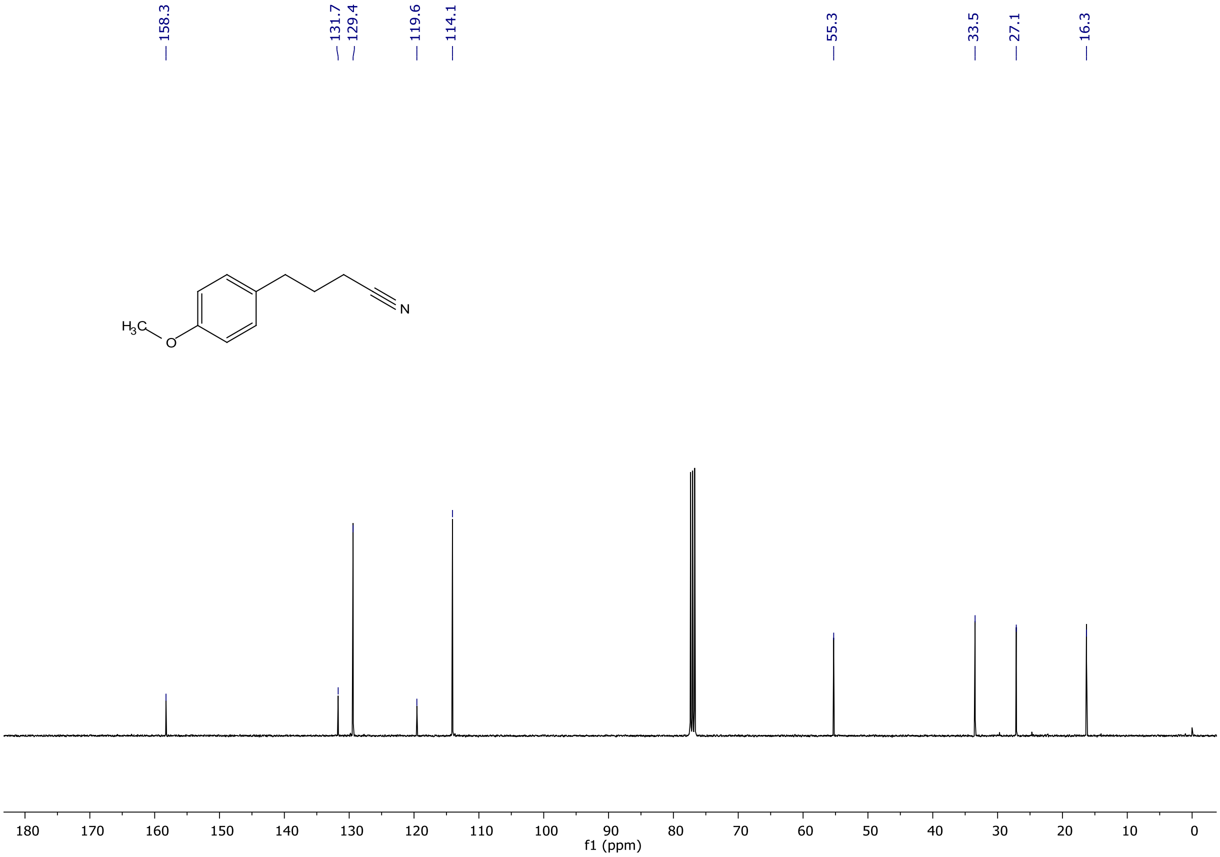

1-methoxy-4-(3-(phenylsulfonyl)propyl)benzene (3ag)

^{1}H-NMR (400 MHz, $CDCl_3$)

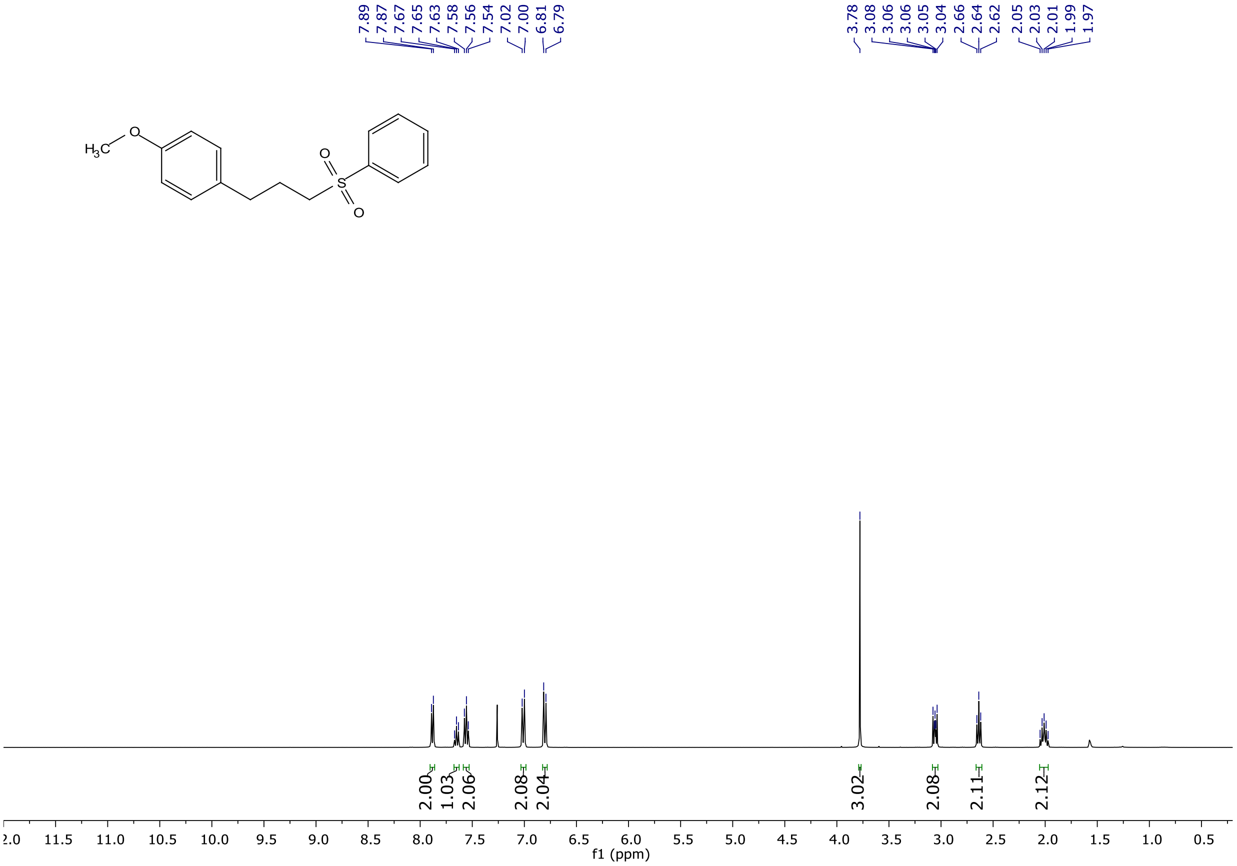

^{13}C-NMR (101 MHz, $CDCl_3$)

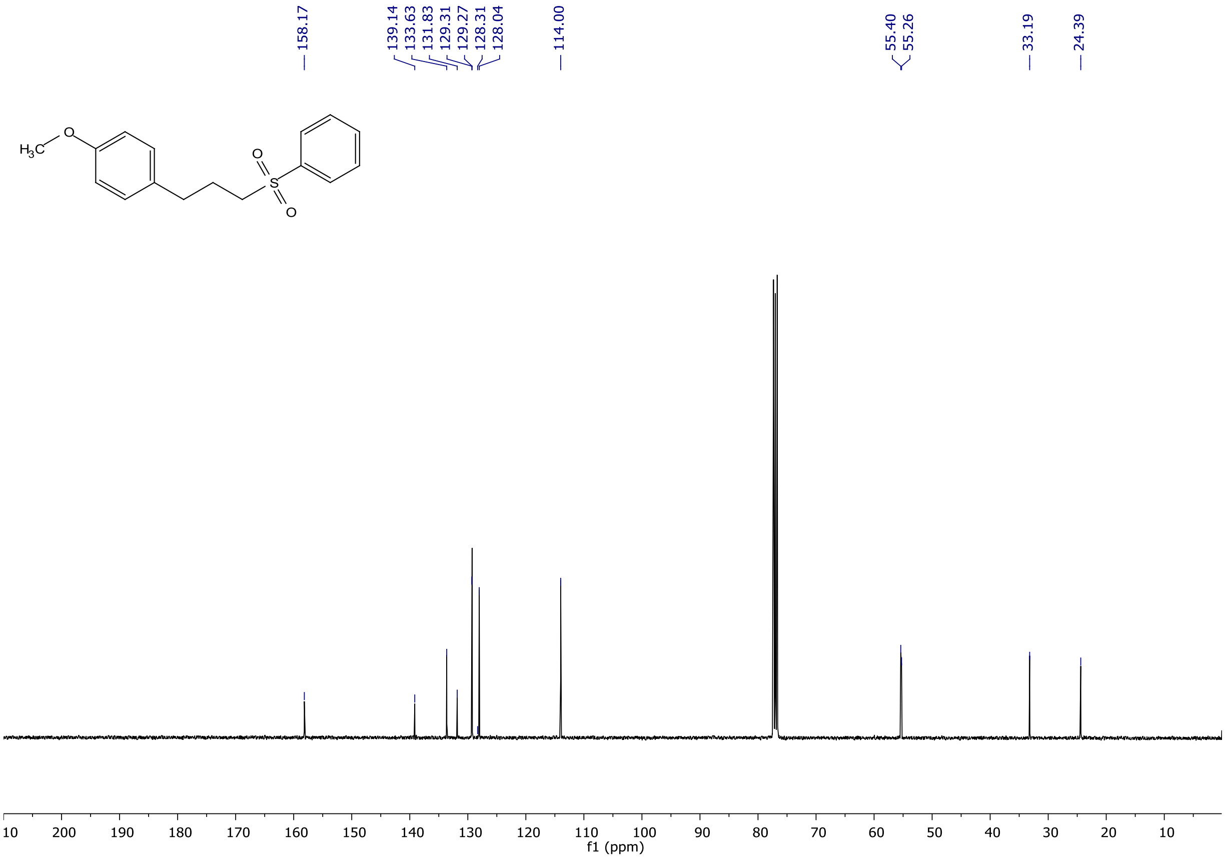

methyl 4-(4-methoxyphenyl)-2-methylbutanoate (3ah)

^{1}H-NMR (400 MHz, $CDCl_3$)

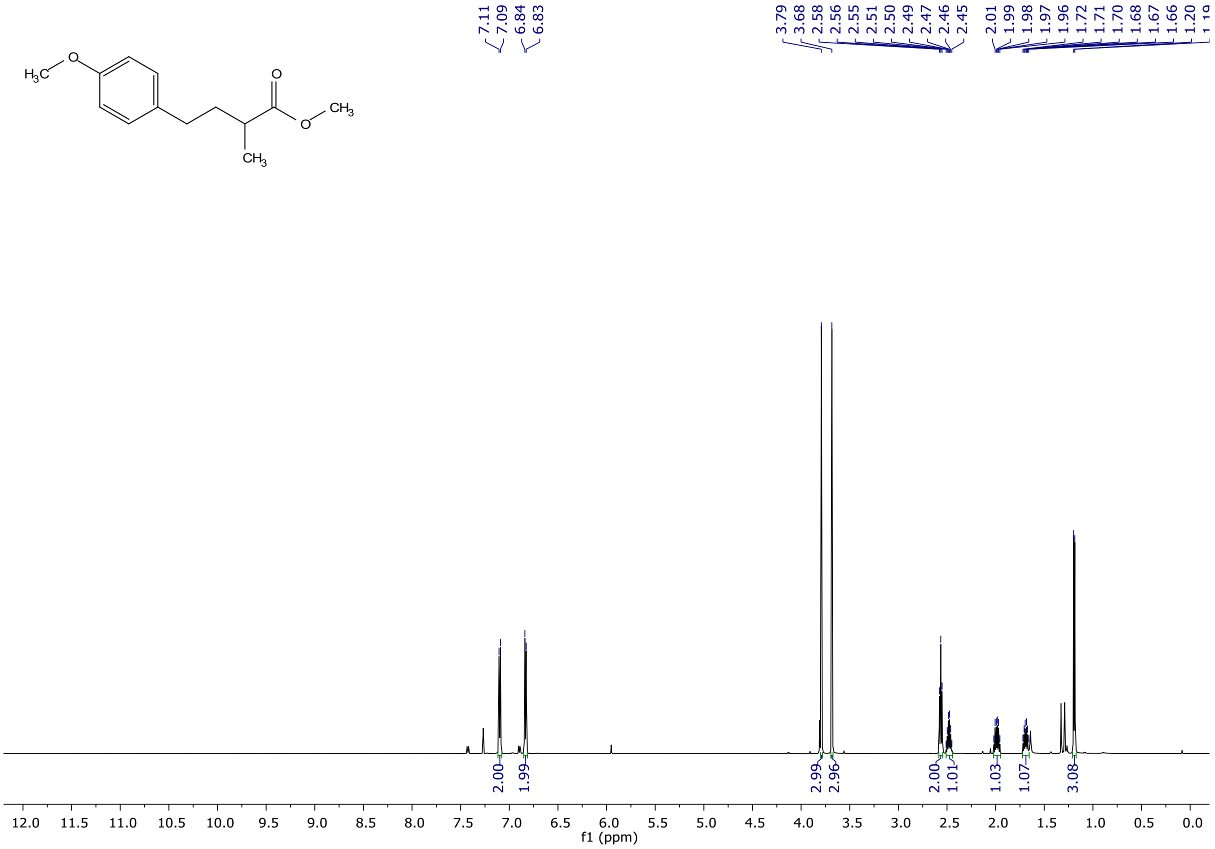

^{13}C-NMR (101 MHz, $CDCl_3$)

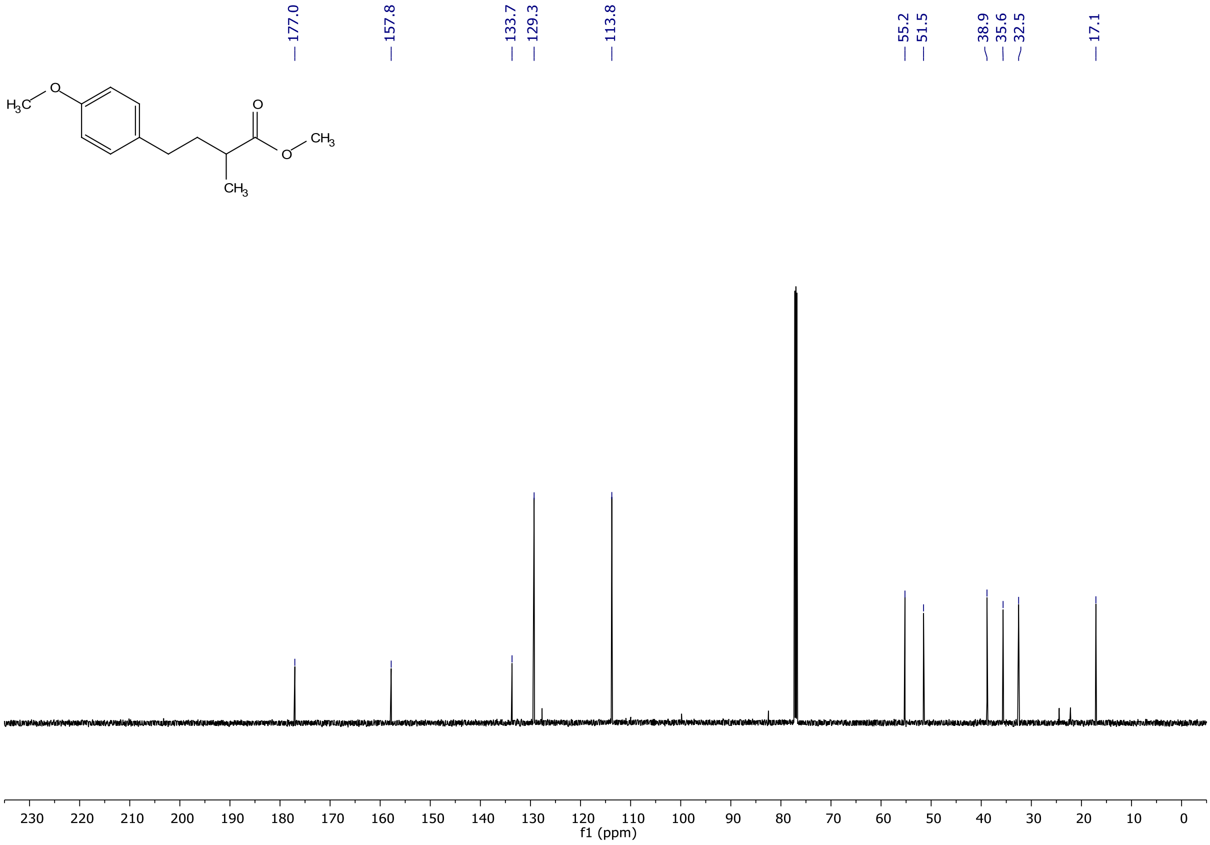

3-(4-methoxyphenethyl) dihydrofuran-2(3*H*)-one (3ai)

^{1}H-NMR (600 MHz, $CDCl_3$)

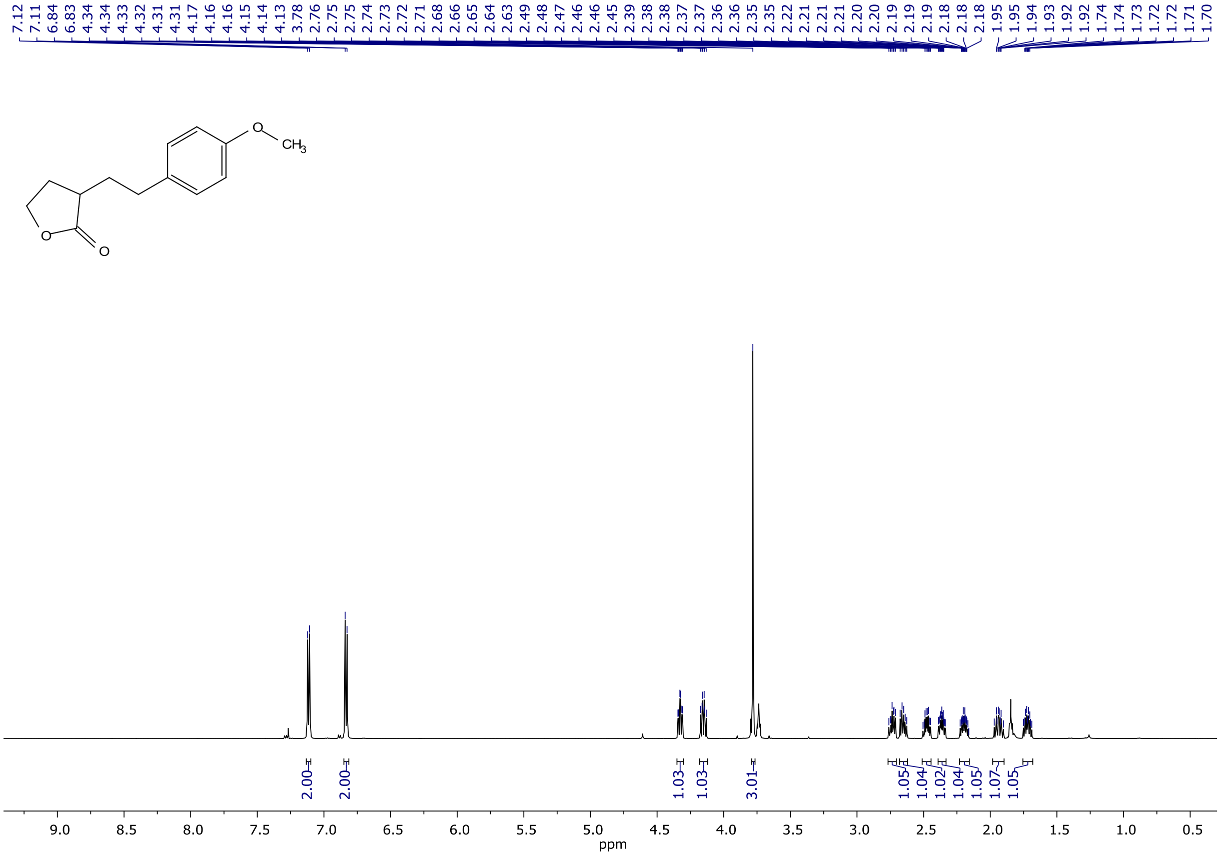

^{13}C-NMR (151 MHz, $CDCl_3$)

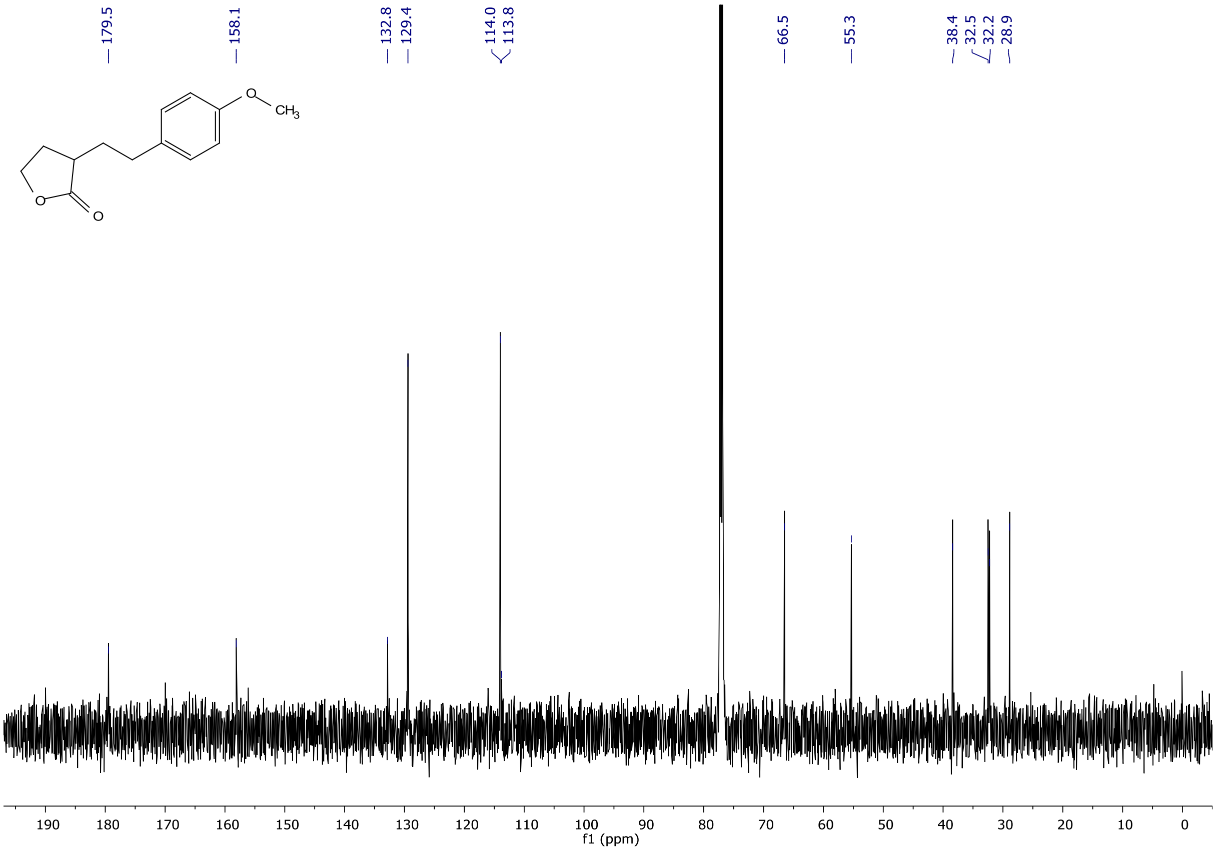

2-(4-methoxybenzyl)cyclopentanone (3aj)

^{1}H-NMR (400 MHz, $CDCl_3$)

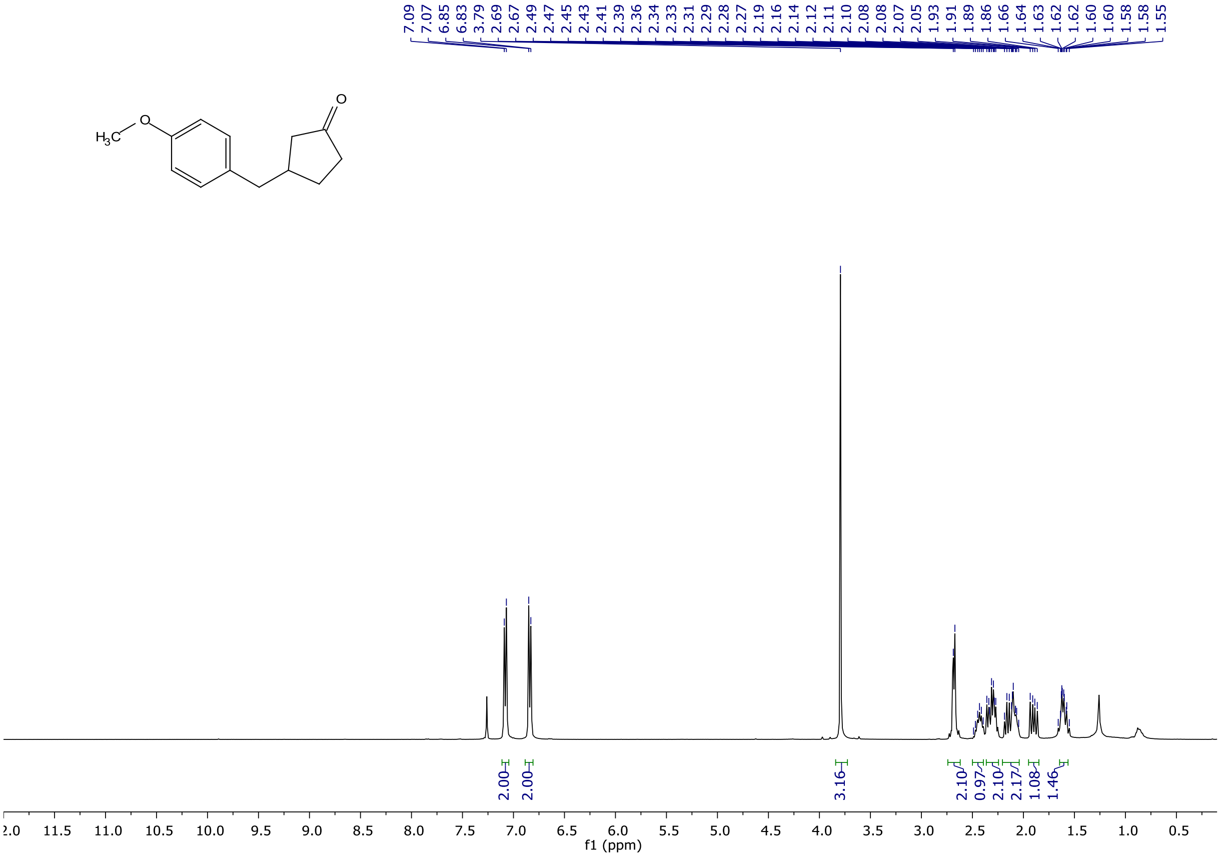

^{13}C-NMR (101 MHz, $CDCl_3$)

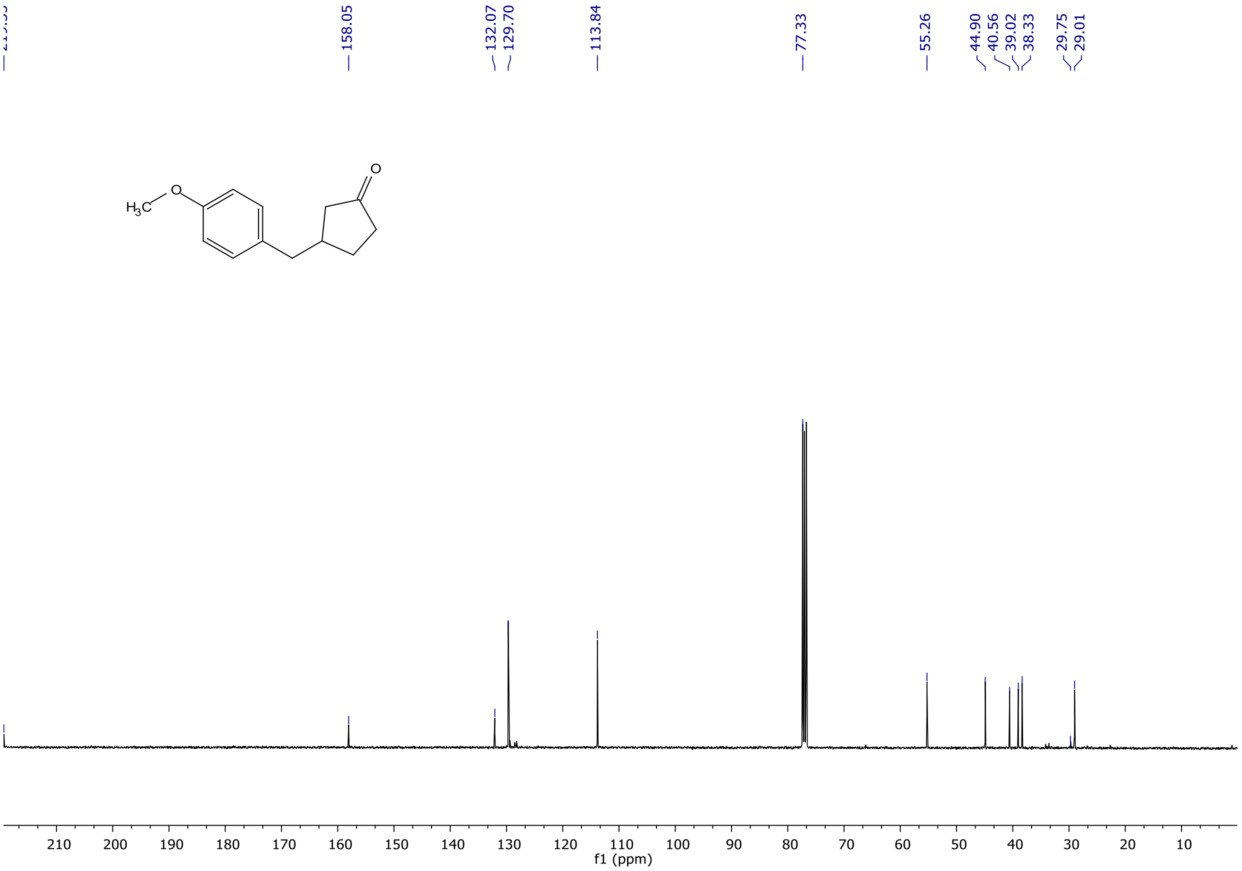

3-(4-methoxybenzyl)cyclohexanone (3ak)

^{1}H-NMR (400 MHz, $CDCl_3$)

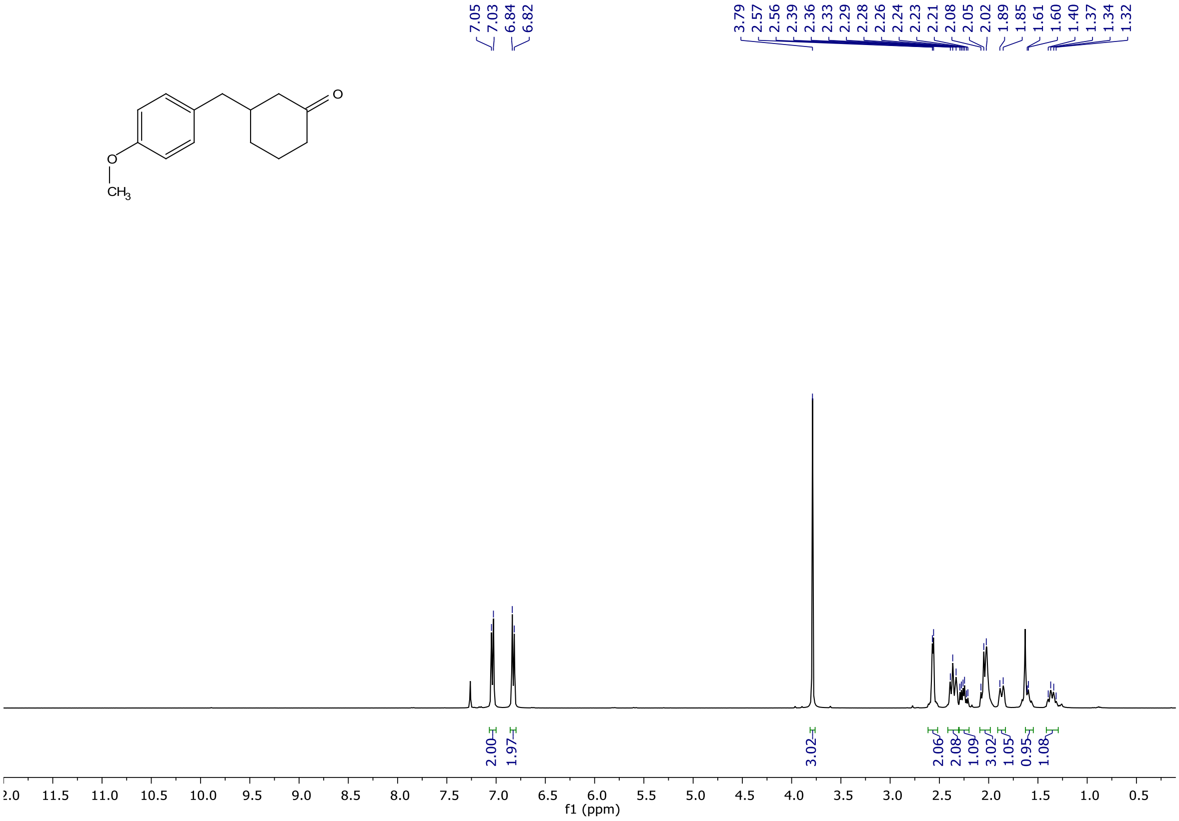

^{13}C-NMR (101 MHz, $CDCl_3$)

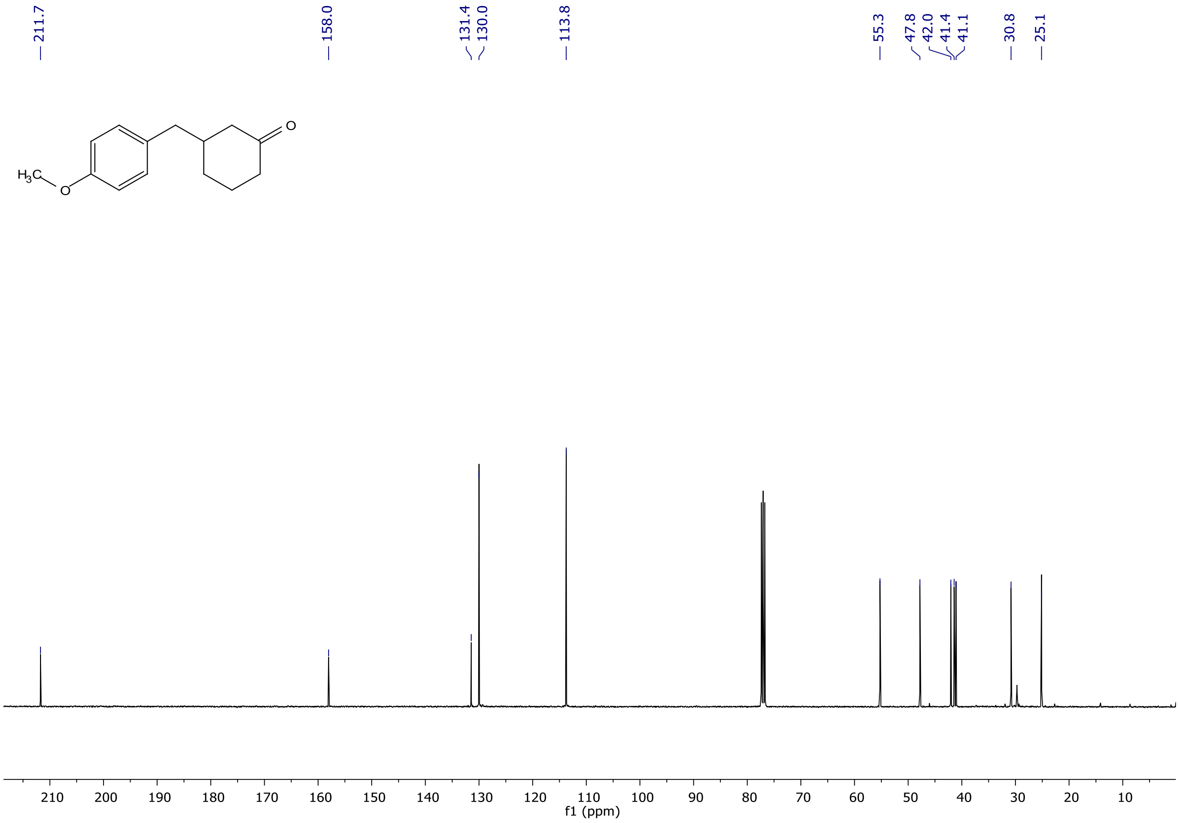

Diethyl 2-(4-methoxybenzyl)succinate (3al)

^{1}H-NMR (400 MHz, $CDCl_3$)

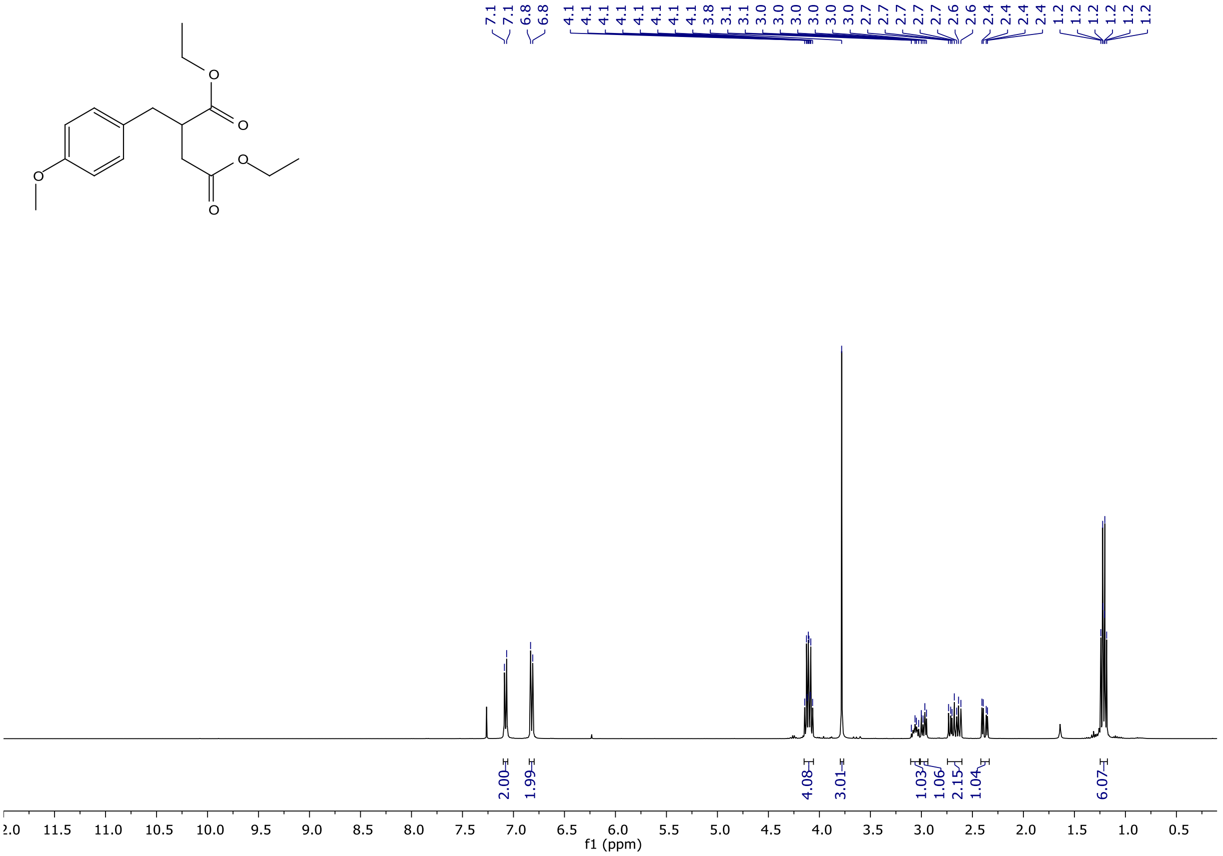

^{13}C-NMR (101 MHz, $CDCl_3$)

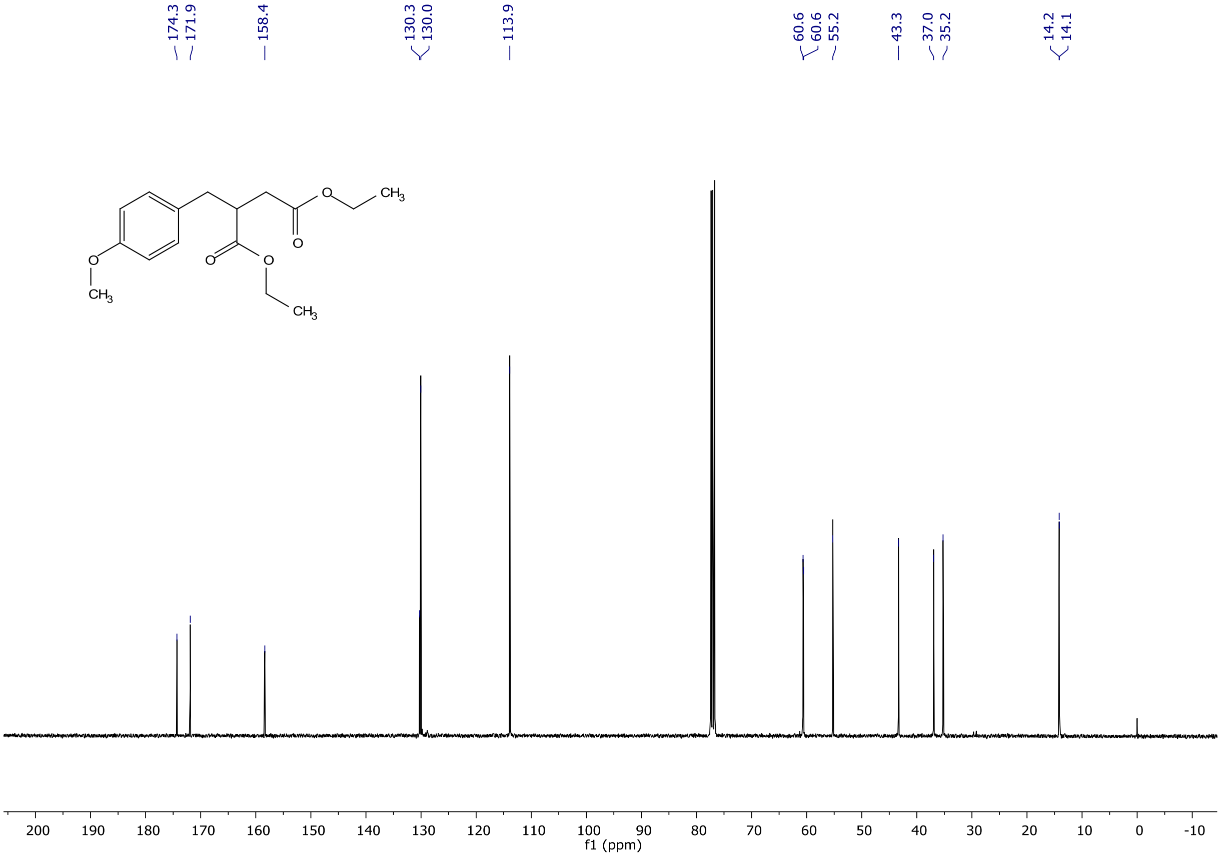

4-(3-(4-methoxyphenyl)propyl)pyridine (3am)

^{1}H-NMR (600 MHz, $CDCl_3$)

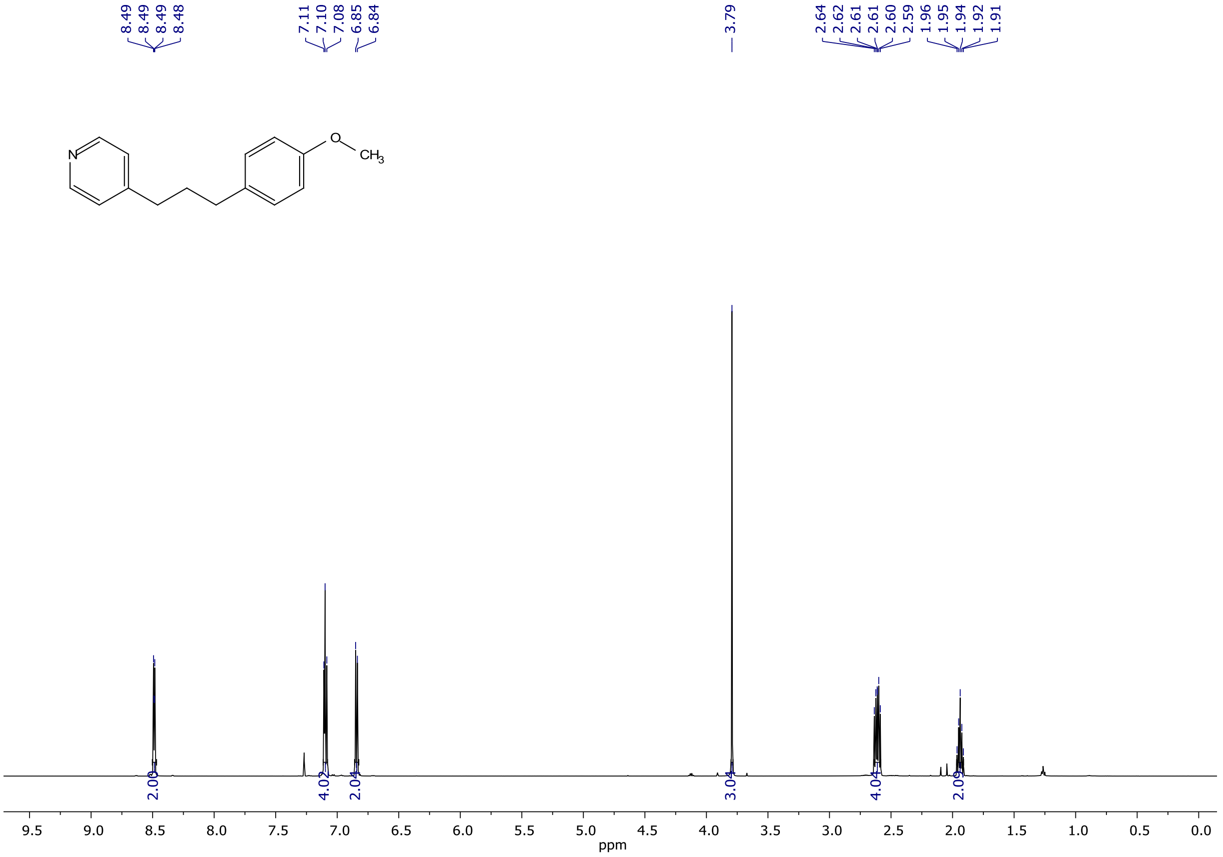

^{13}C-NMR (151 MHz, $CDCl_3$)

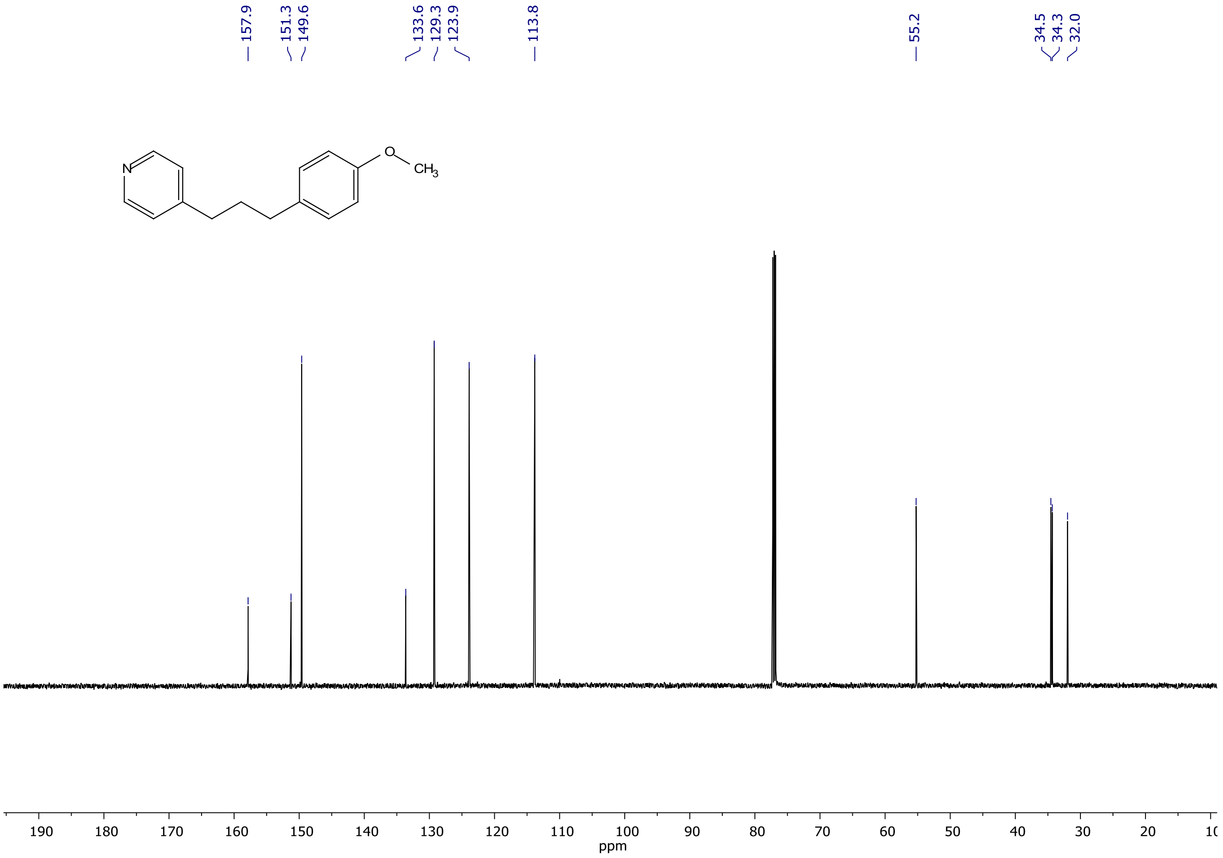

2-(3-(4-methoxyphenyl)propyl)pyridine (3an)

^{1}H-NMR (400 MHz, $CDCl_3$)

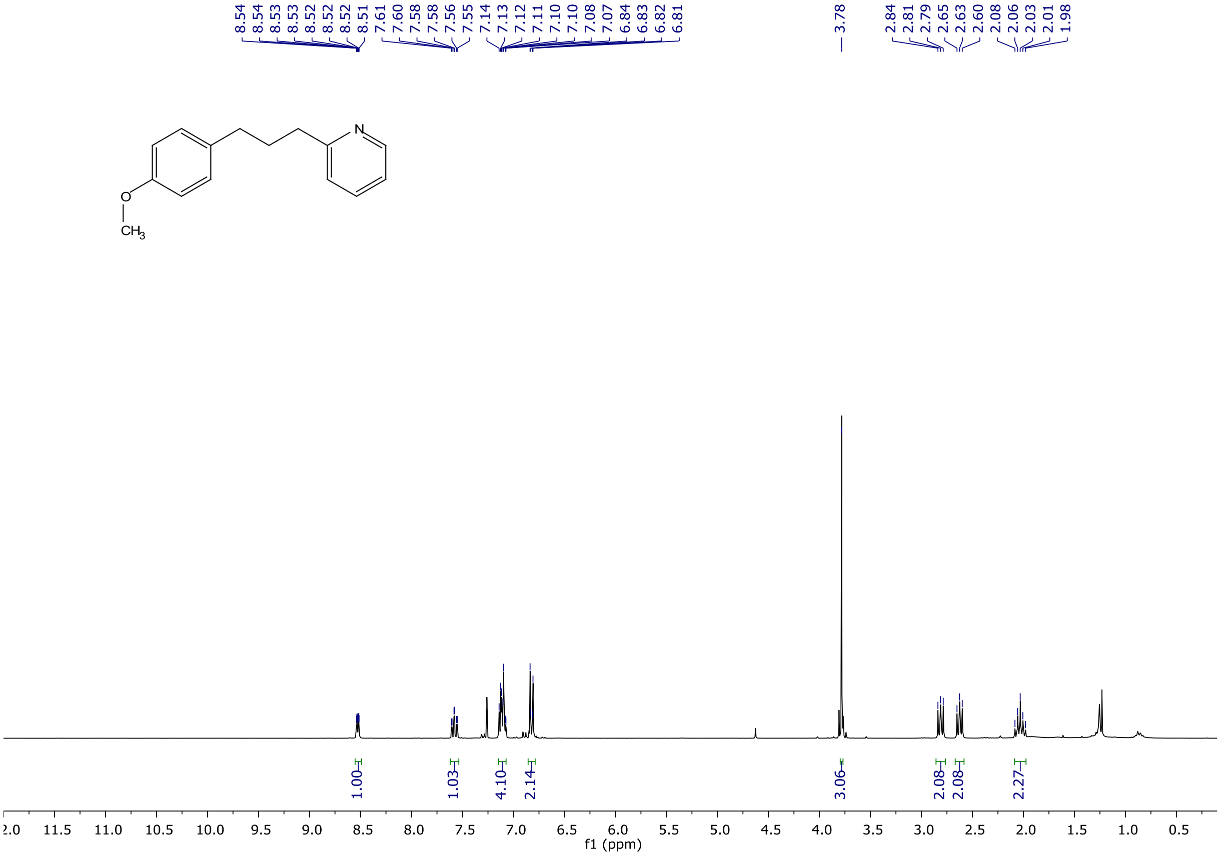

^{13}C-NMR (101 MHz, $CDCl_3$)

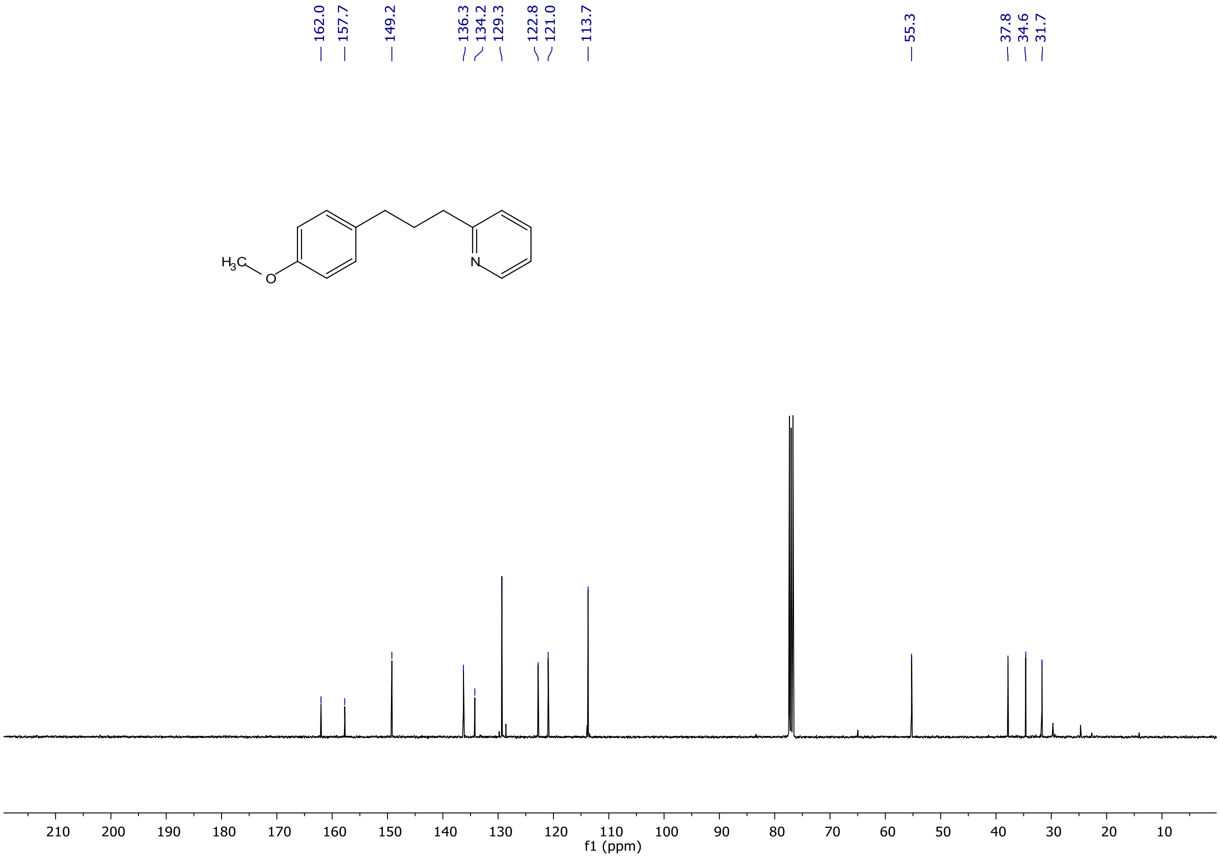

2-(3-(4-methoxyphenyl)-1-phenylpropyl)pyridine (3ao)

^{1}H-NMR (400 MHz, $CDCl_3$)

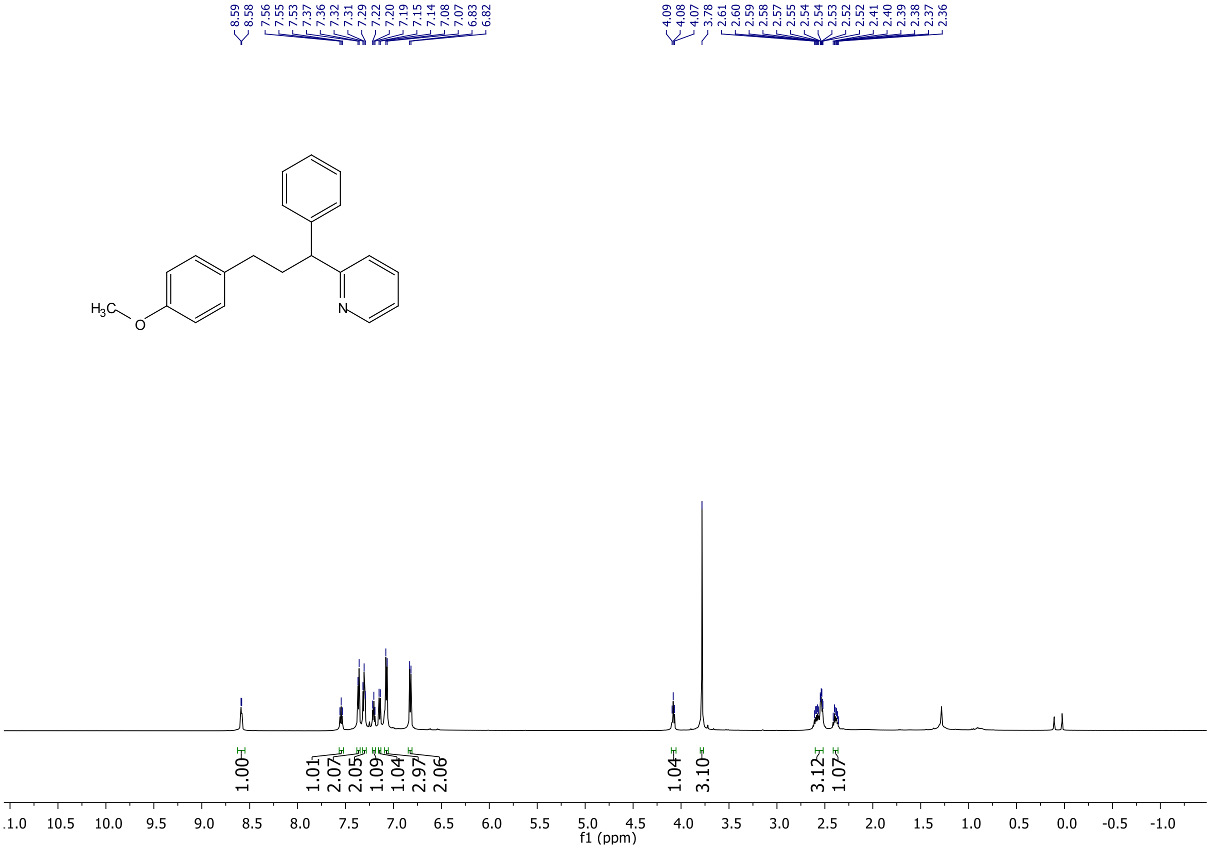

^{13}C-NMR (101 MHz, $CDCl_3$)

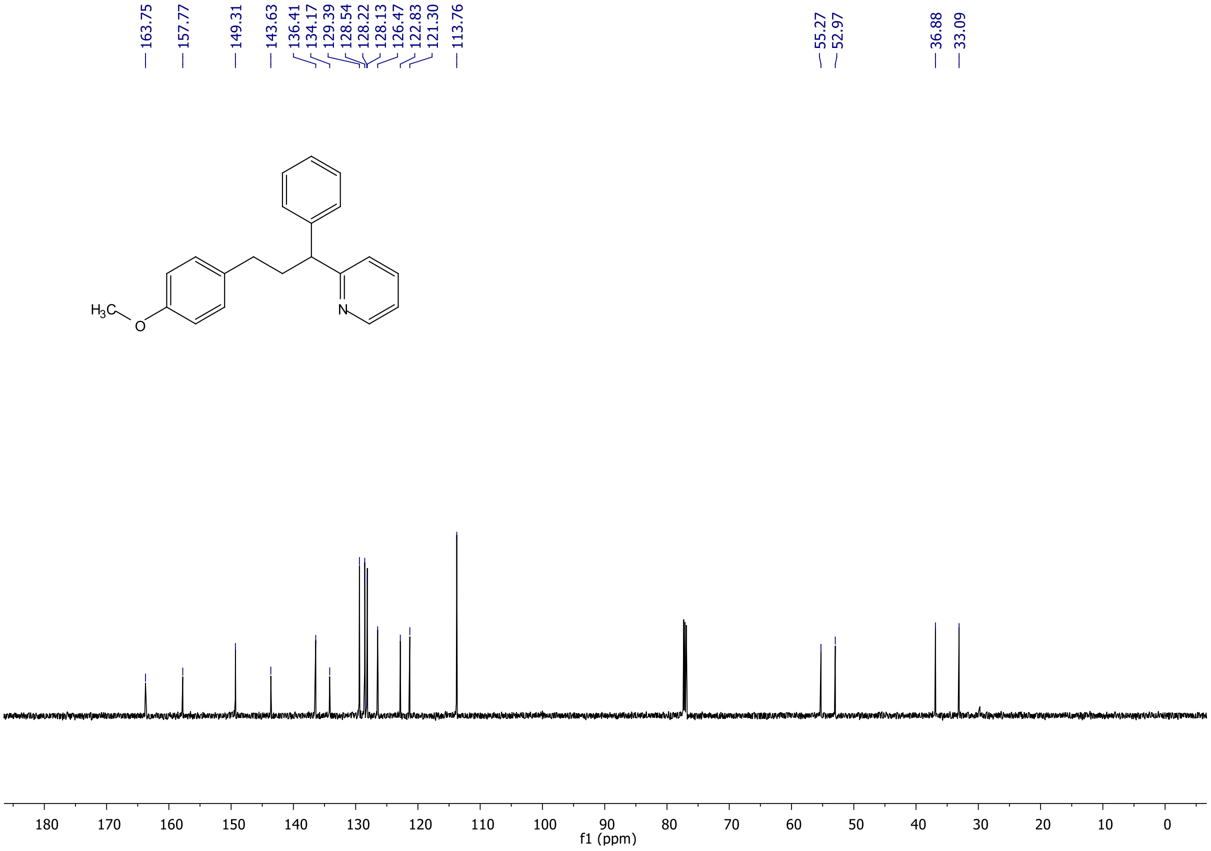

1,2,3,4,5-pentafluoro-6-(3-(4-methoxyphenyl)propyl)benzene (3ap)

^{1}H-NMR (400 MHz, $CDCl_3$)

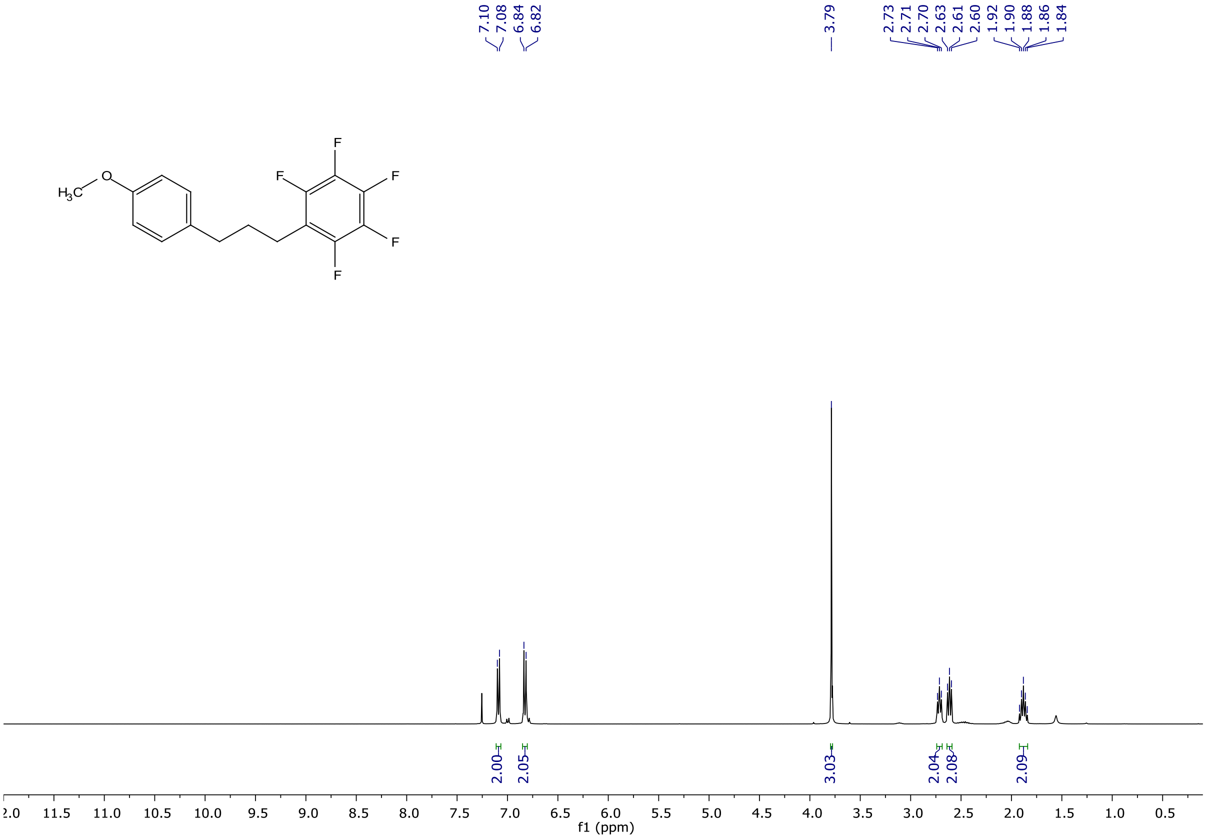

^{13}C-NMR (101 MHz, $CDCl_3$)

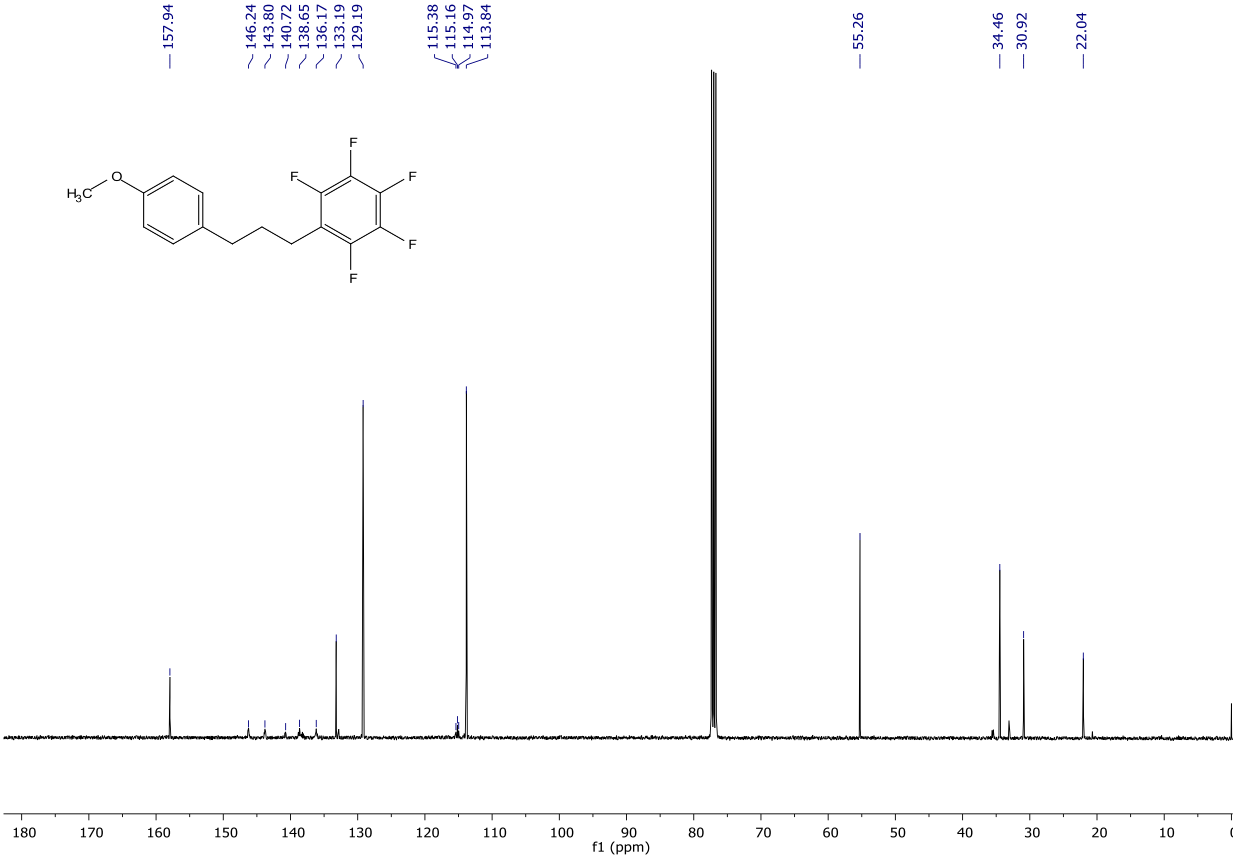

3-(4-methoxybenzyl) chroman-4-one (3aq)

^{1}H-NMR (400 MHz, $CDCl_3$)

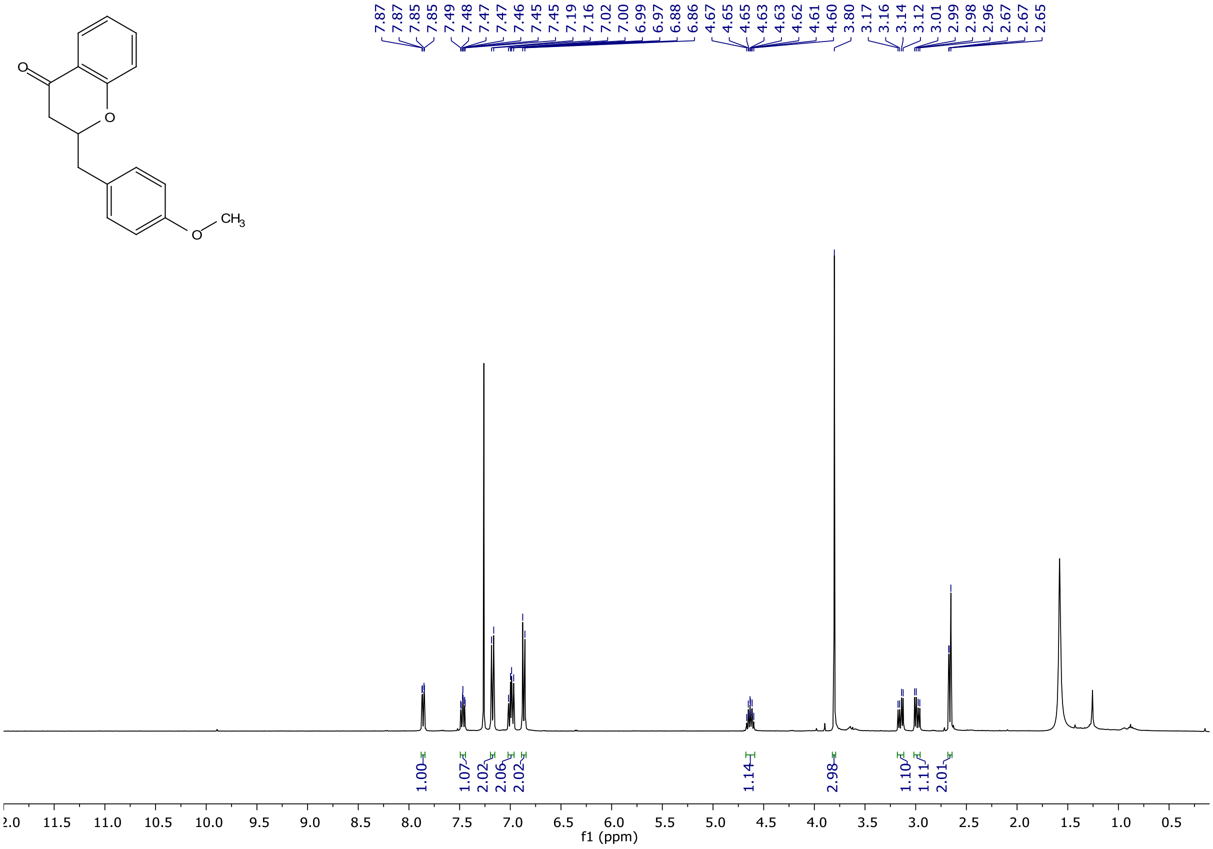

^{13}C-NMR (101 MHz, $CDCl_3$)

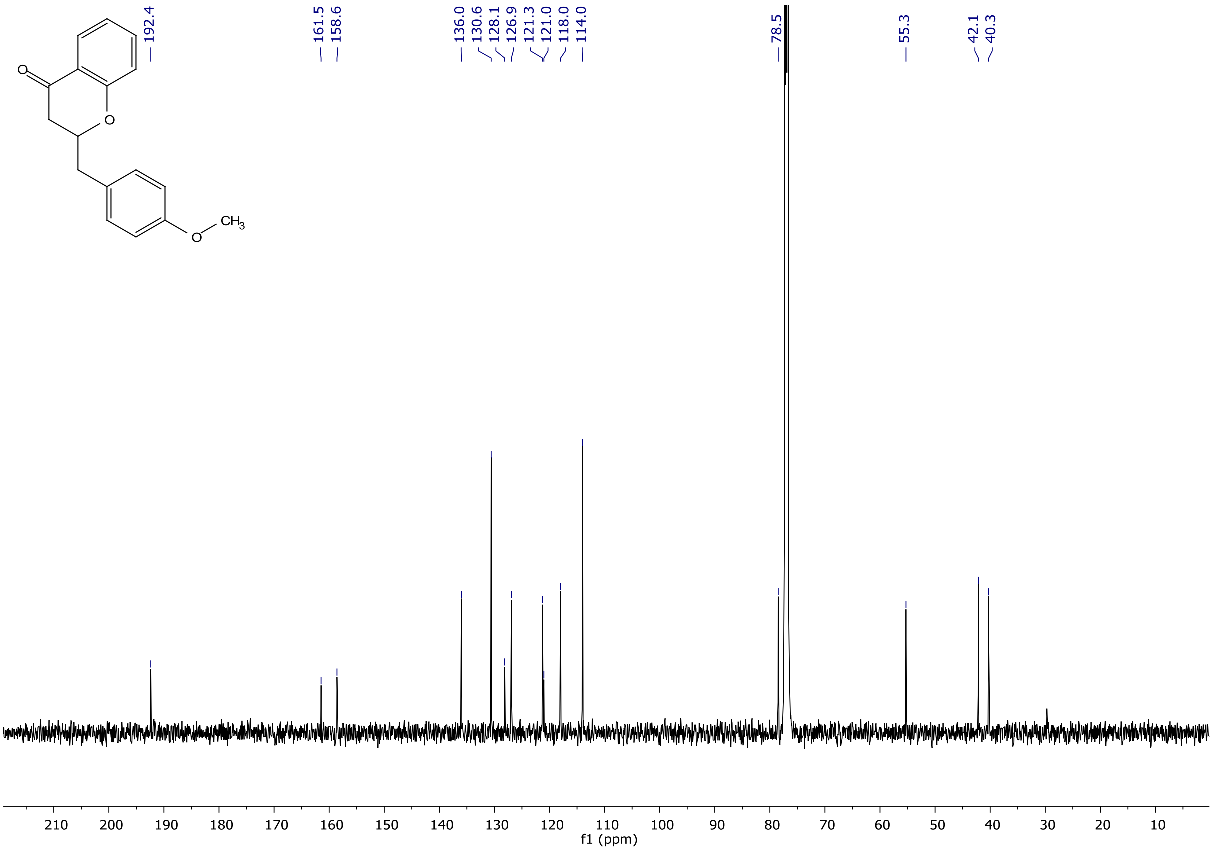

3-(4-methoxybenzyl)-2-phenylchroman-4-one (3ar)

^{1}H-NMR (400 MHz, $CDCl_3$)

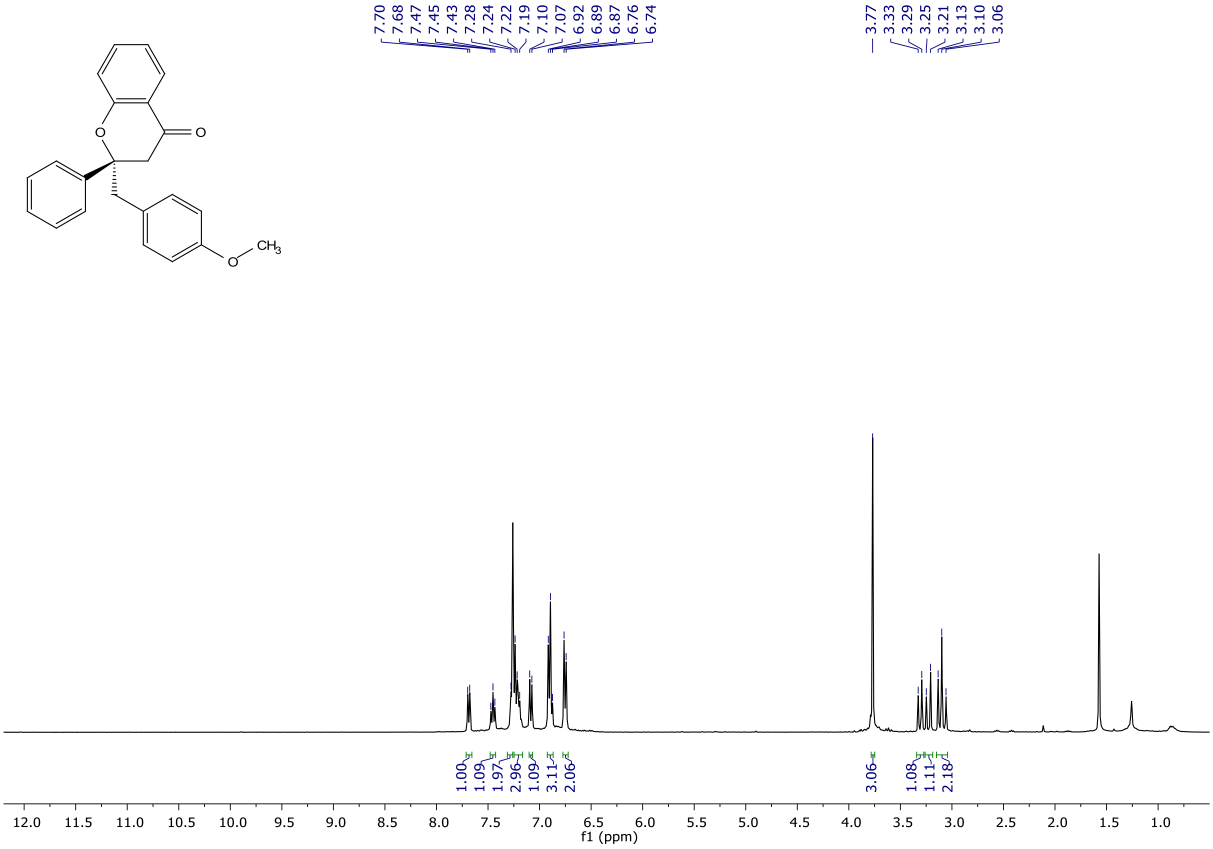

^{13}C-NMR (101 MHz, $CDCl_3$)

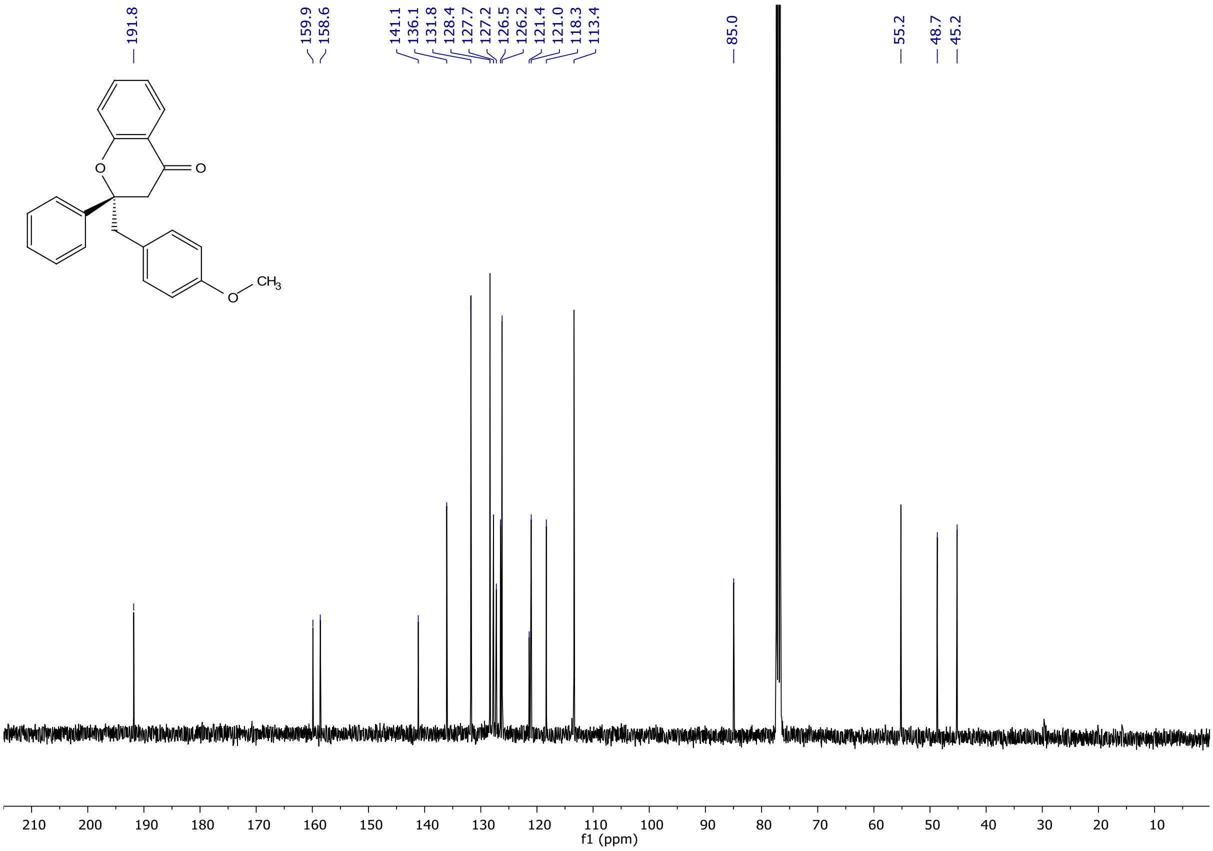

7.2.2 Scope of alkyl boronic esters

5-(4-methoxyphenyl)pentan-2-one (3ad)

^{1}H-NMR (600 MHz, $CDCl_3$)

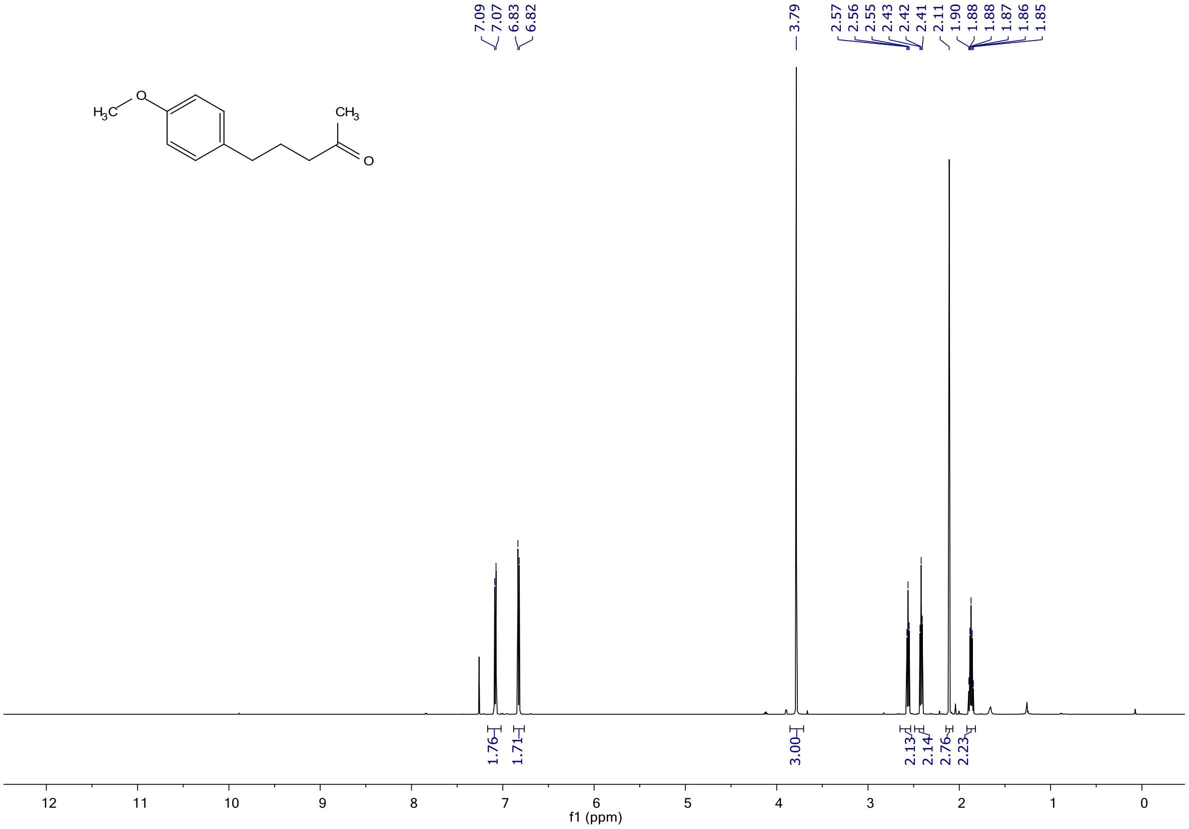

^{13}C-NMR (151 MHz, $CDCl_3$)

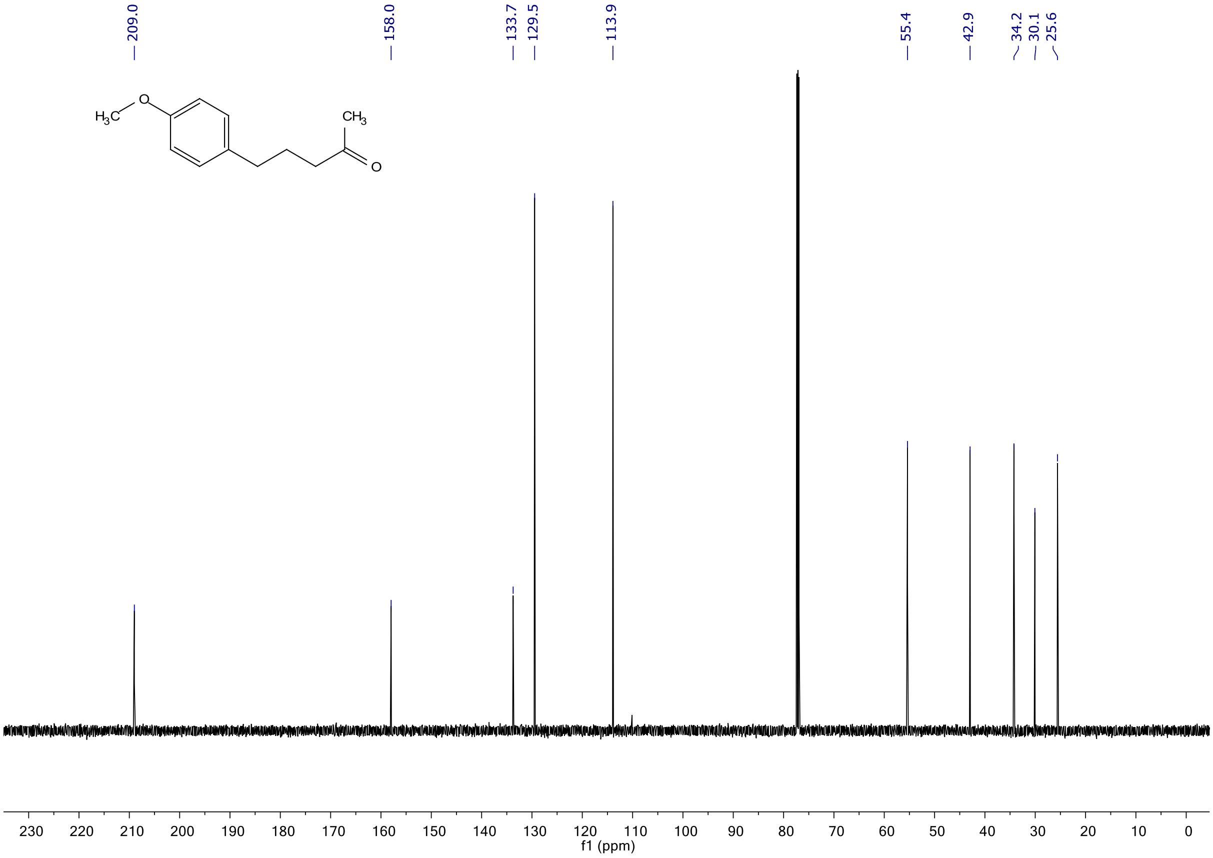

5-phenylpentan-2-one (3bd)

^{1}H-NMR (600 MHz, $CDCl_3$)

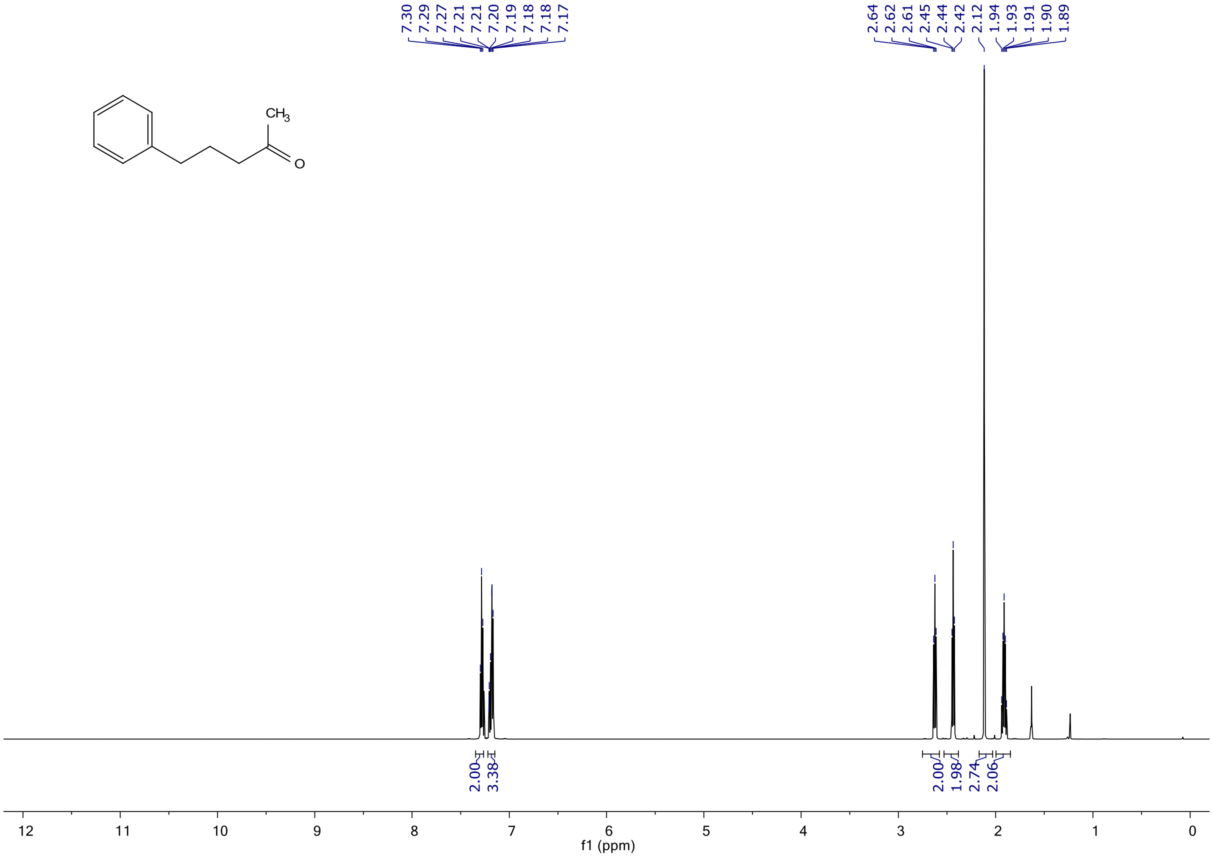

^{13}C-NMR (151 MHz, $CDCl_3$)

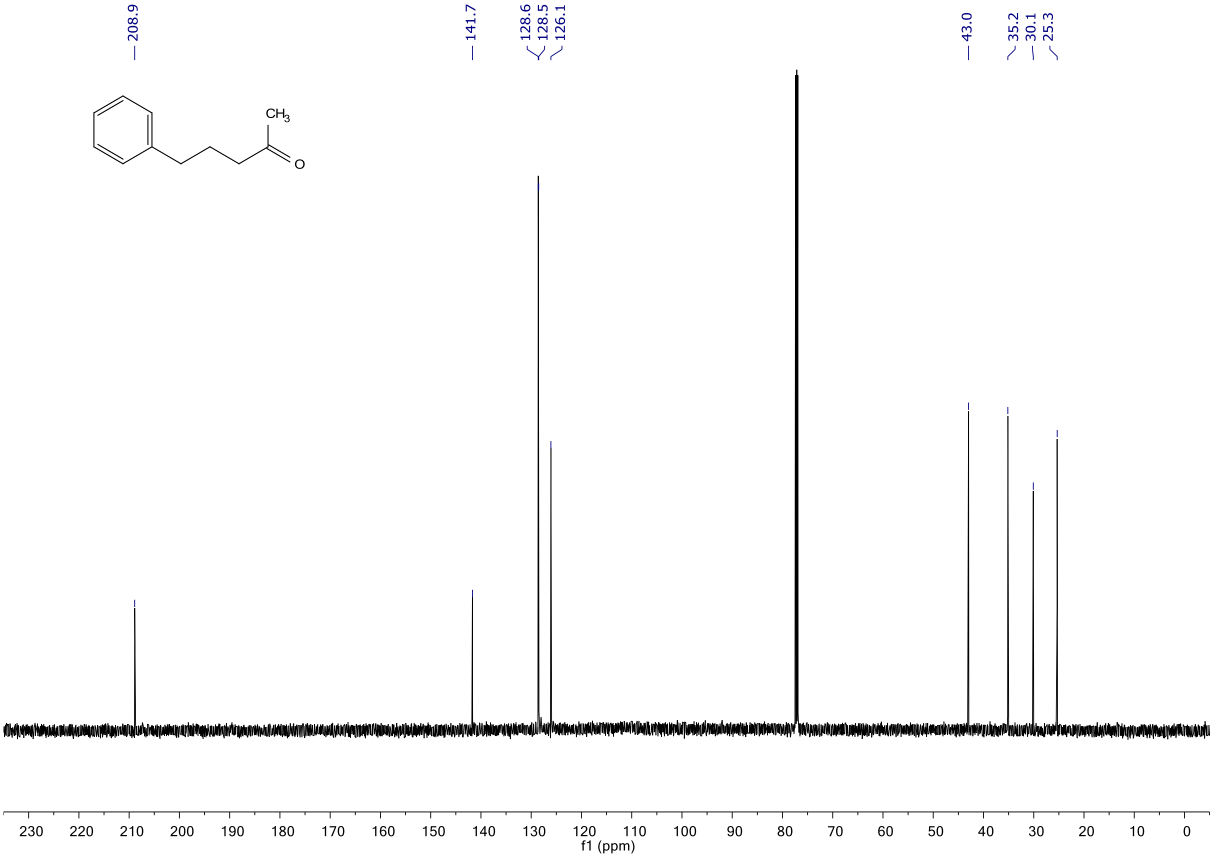

5-(*p*-tolyl)pentan-2-one (3cd)
^{1}H-NMR (600 MHz, $CDCl_3$)

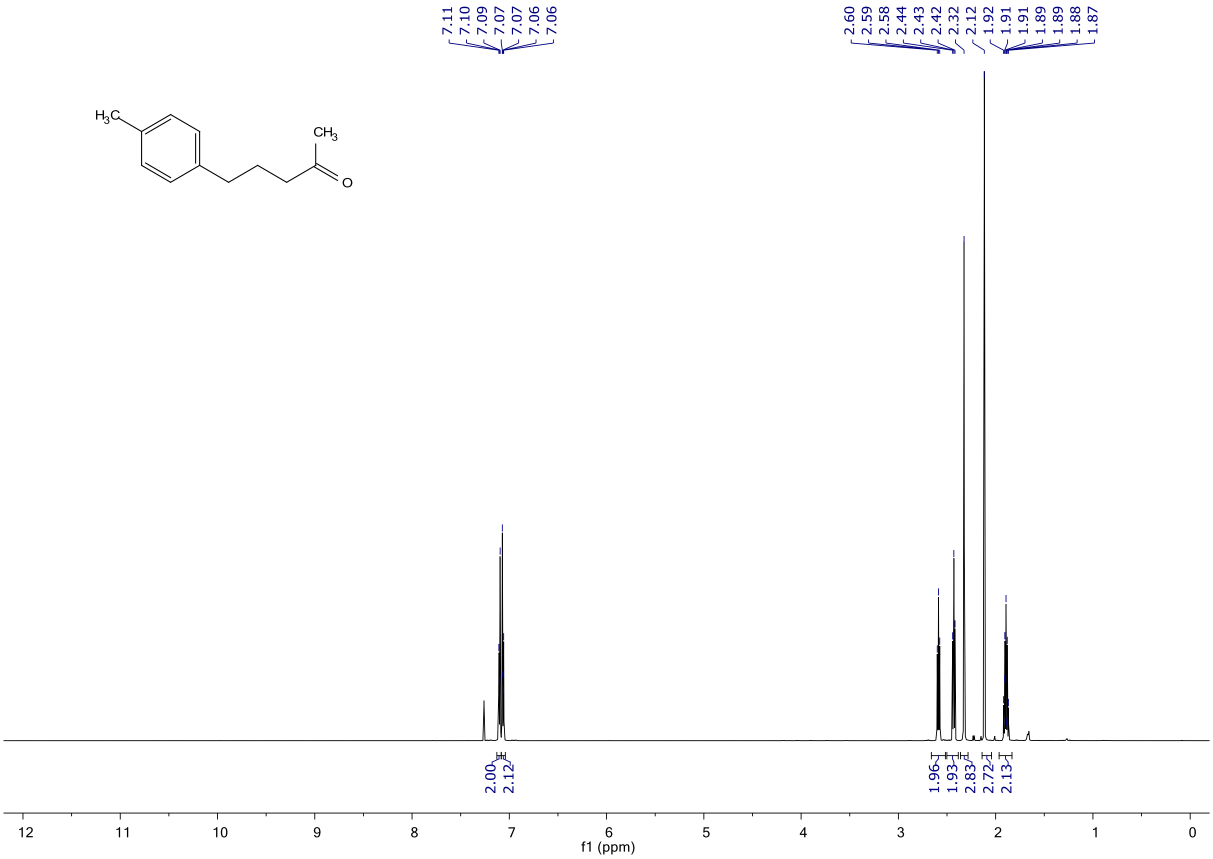

^{13}C-NMR (151 MHz, $CDCl_3$)

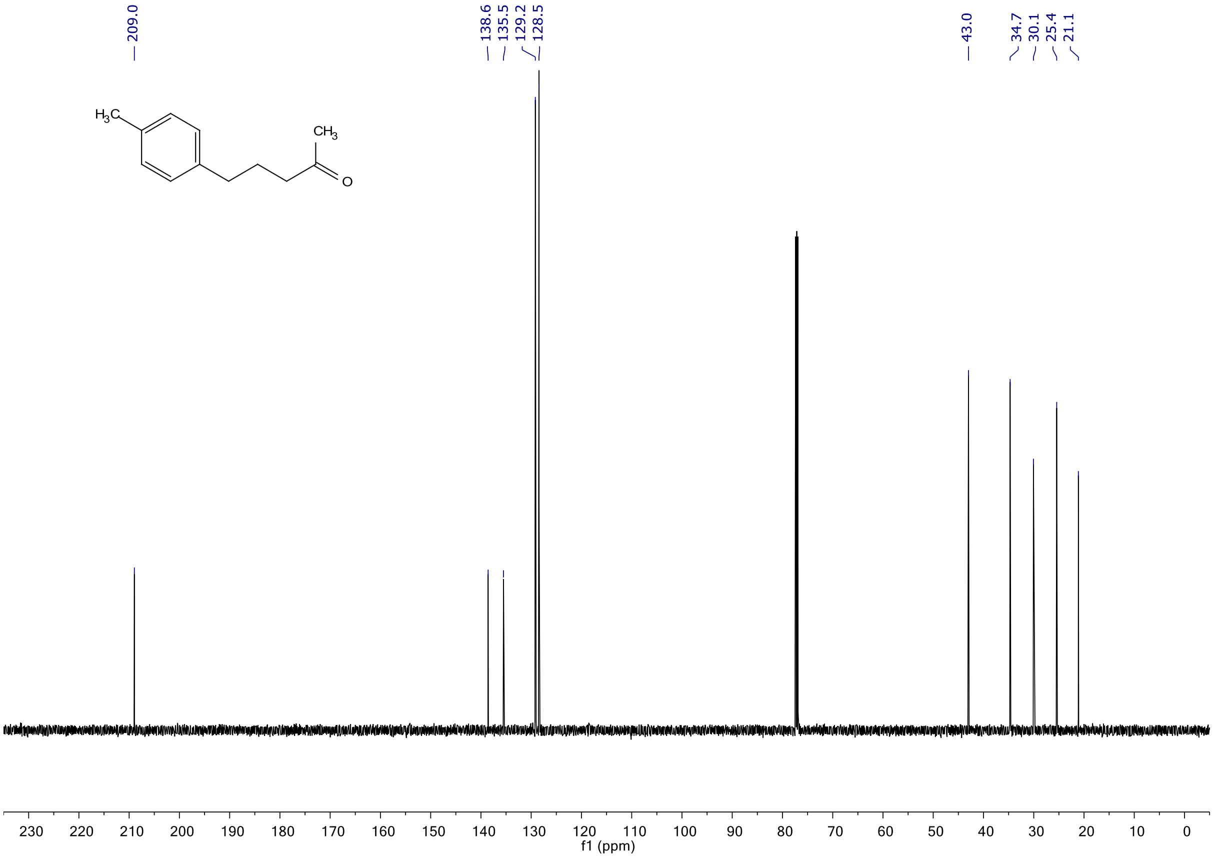

5-(4-fluorophenyl)pentan-2-one (3dd)

^{1}H-NMR (600 MHz, $CDCl_3$)

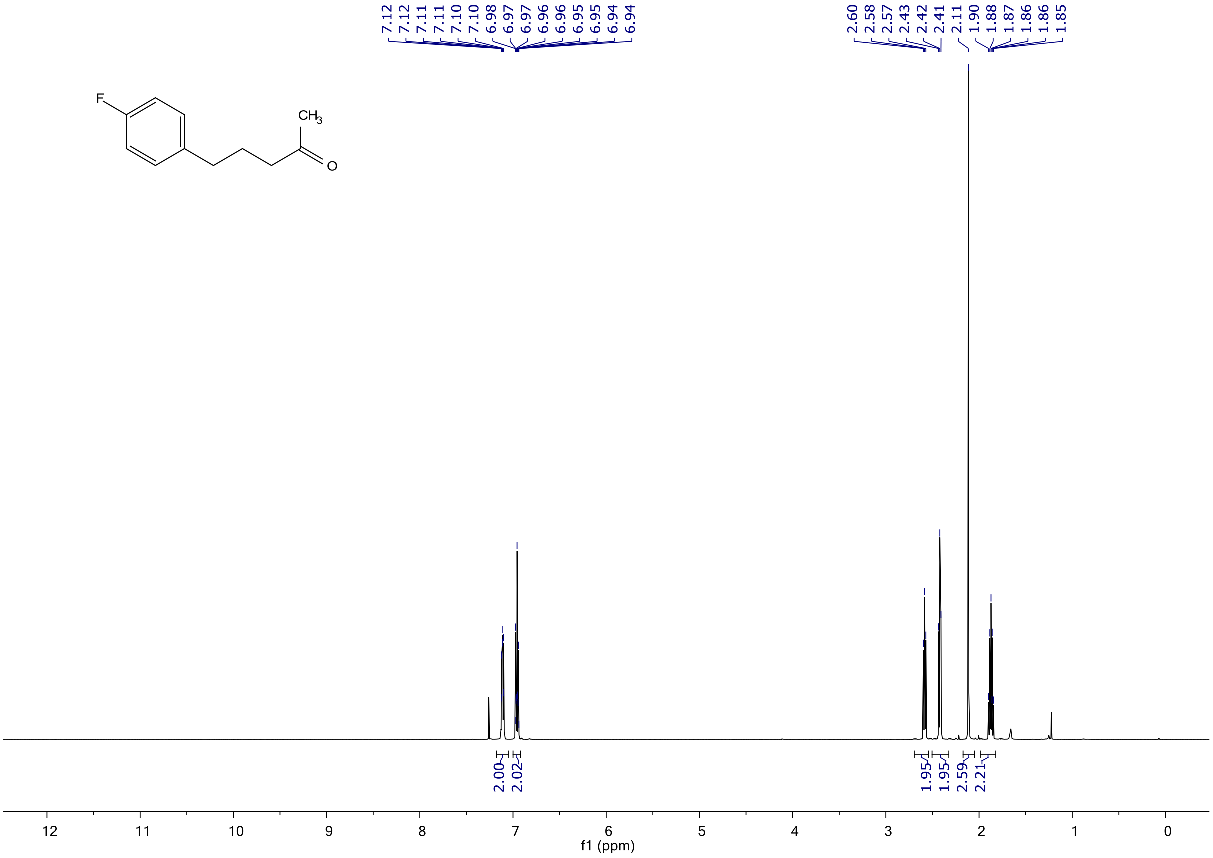

^{13}C-NMR (151 MHz, $CDCl_3$)

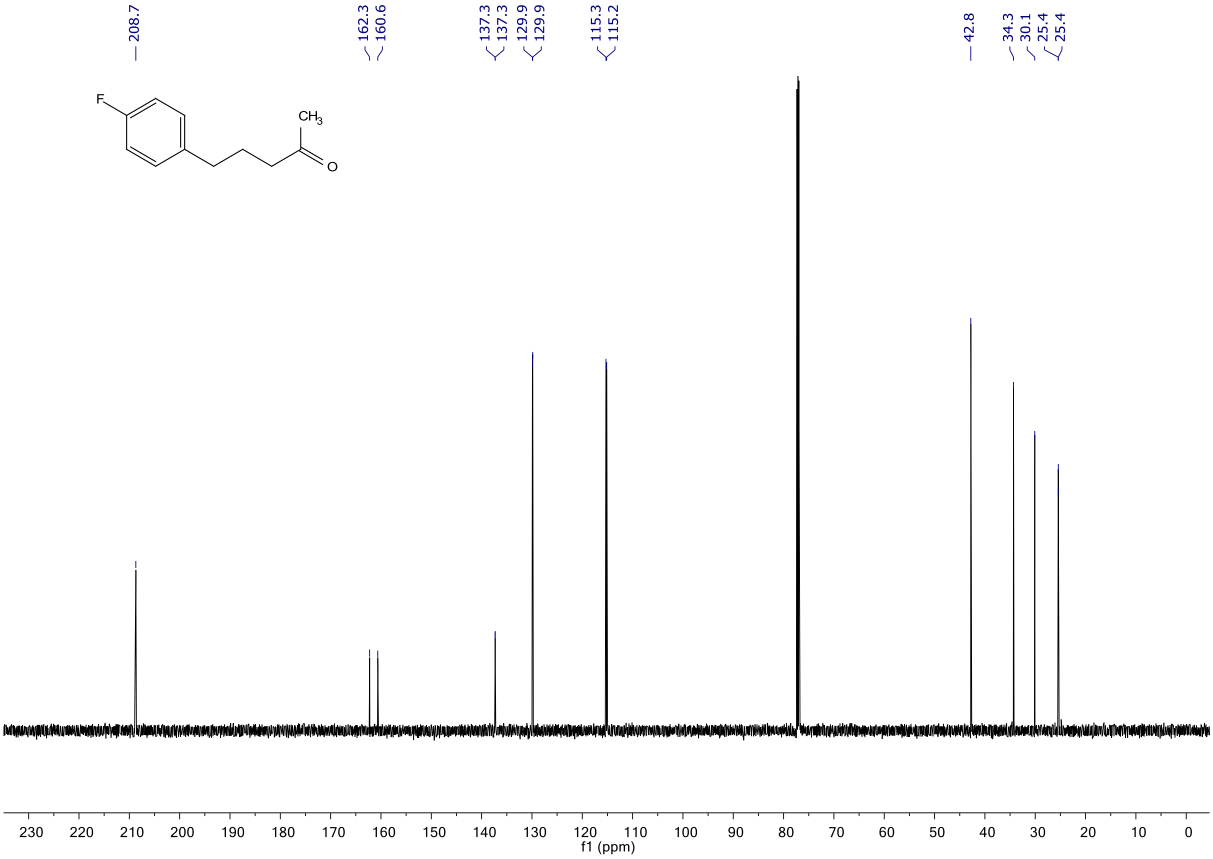

5-(3,5-dimethoxyphenyl)pentan-2-one (3ed)
^{1}H-NMR (600 MHz, $CDCl_3$)

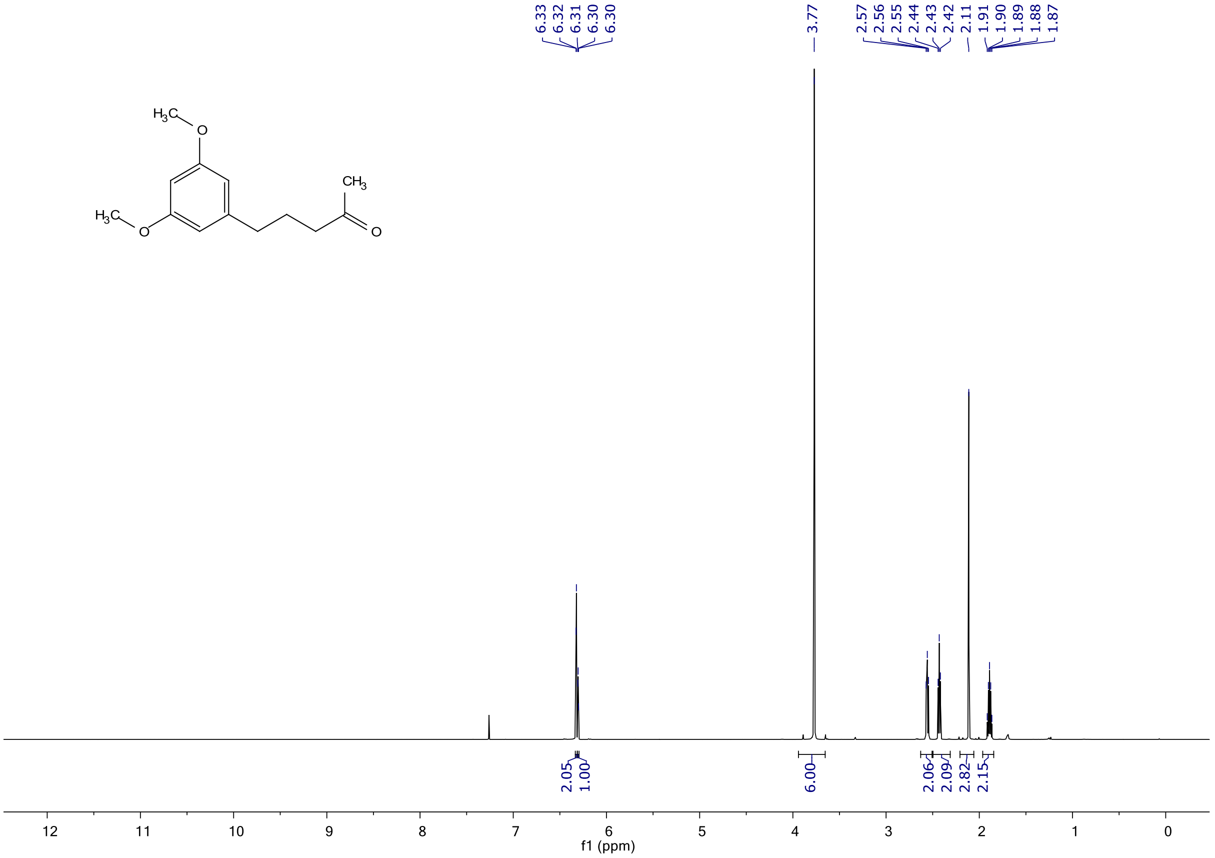

^{13}C-NMR (151 MHz, $CDCl_3$)

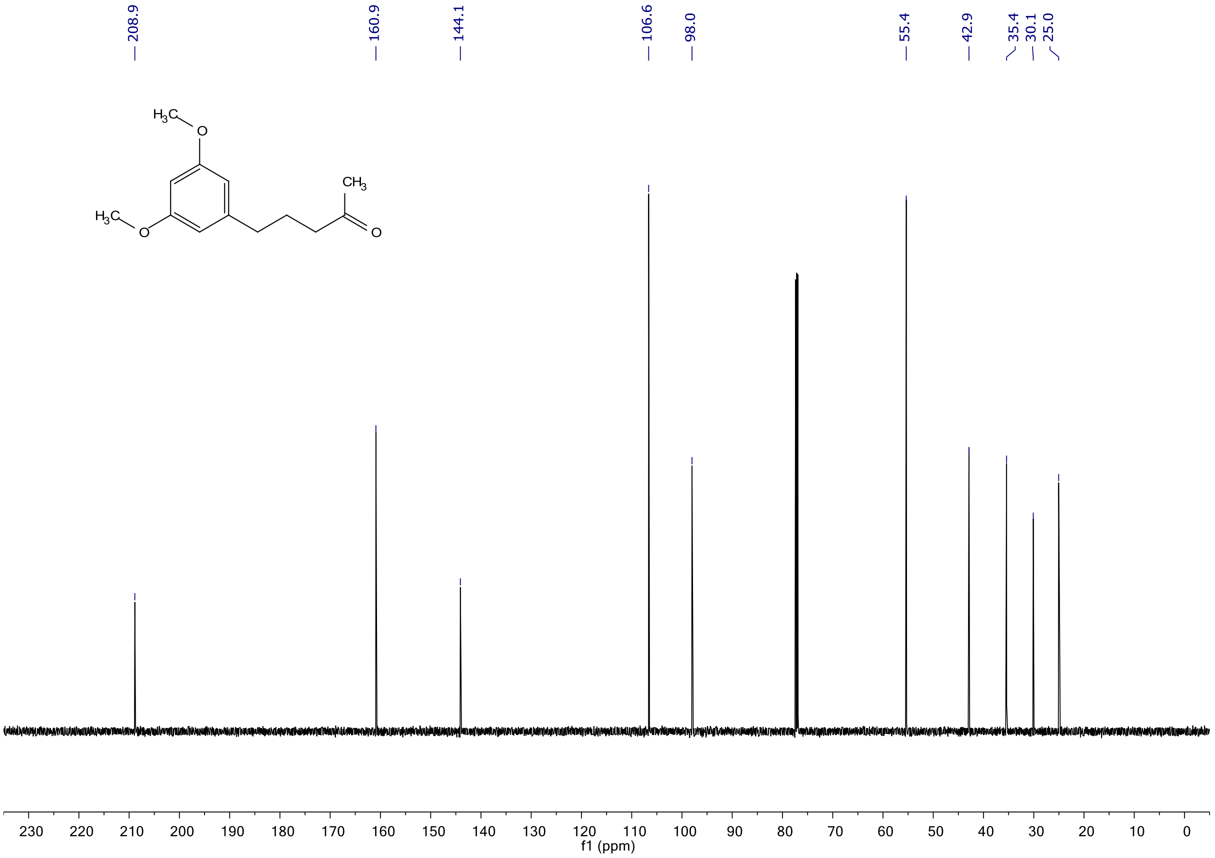

5-(phenylthio)pentan-2-one (3fd)

^{1}H-NMR (600 MHz, $CDCl_3$)

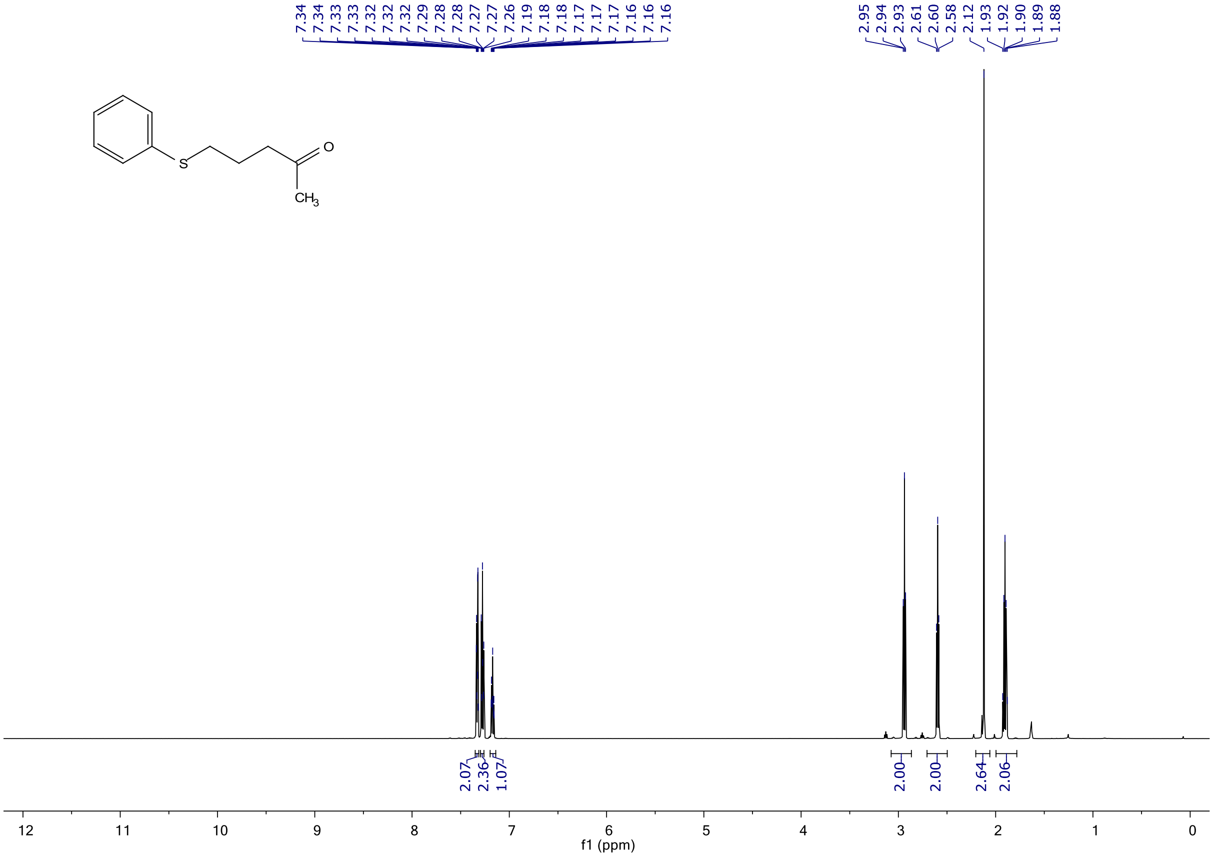

^{13}C-NMR (151 MHz, $CDCl_3$)

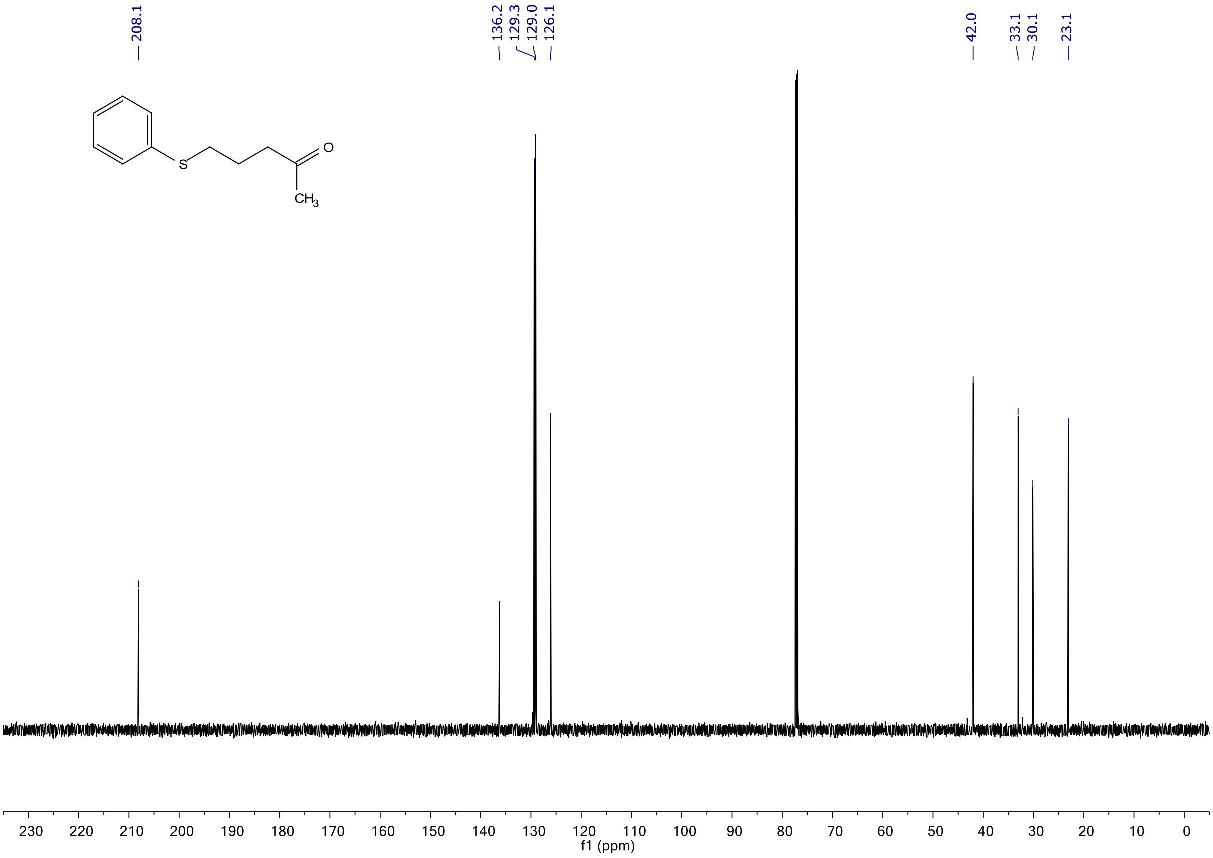

***tert*-butyl (4-oxopentyl)carbamate (3gd)**

^{1}H-NMR (600 MHz, $CDCl_3$)

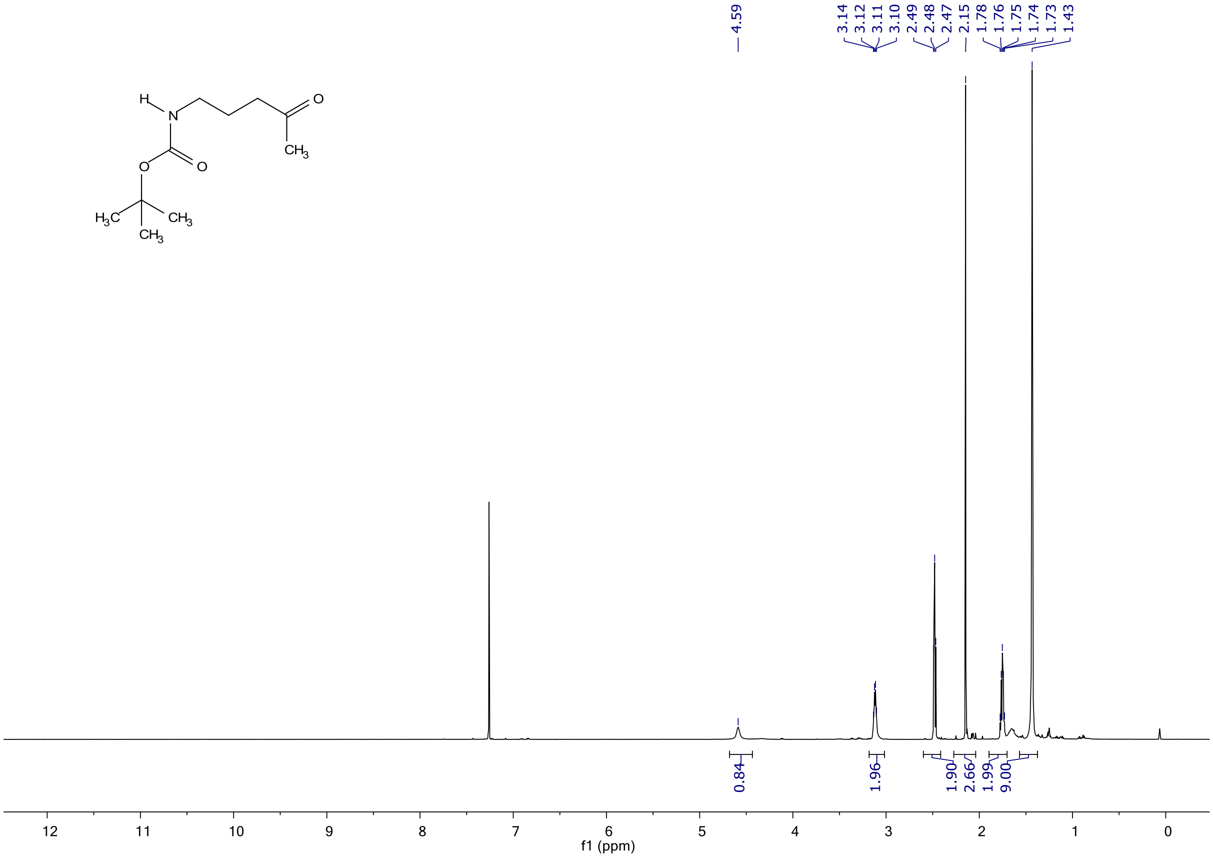

^{13}C-NMR (151 MHz, $CDCl_3$)

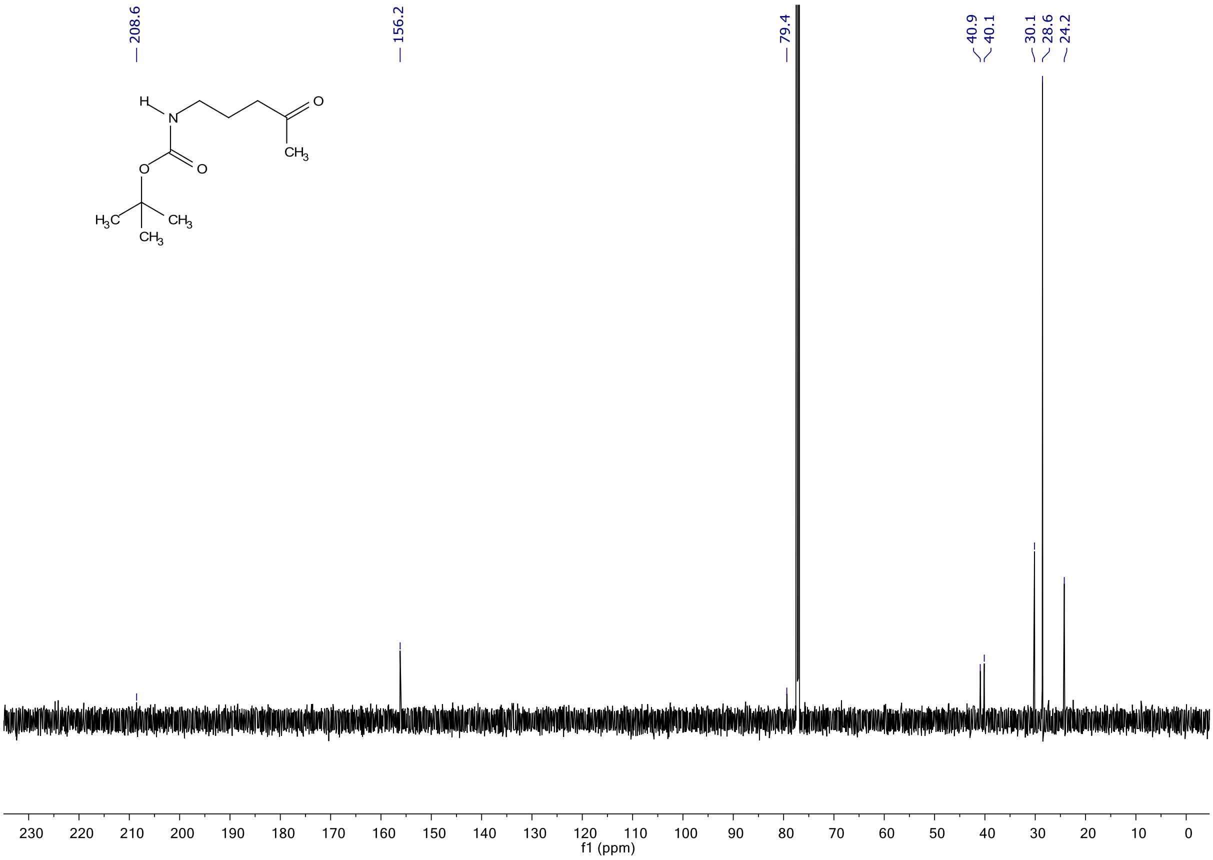

***tert*-butyl methyl(4-oxopentyl)carbamate (3hd)**
^{1}H-NMR (600 MHz, $CDCl_3$)

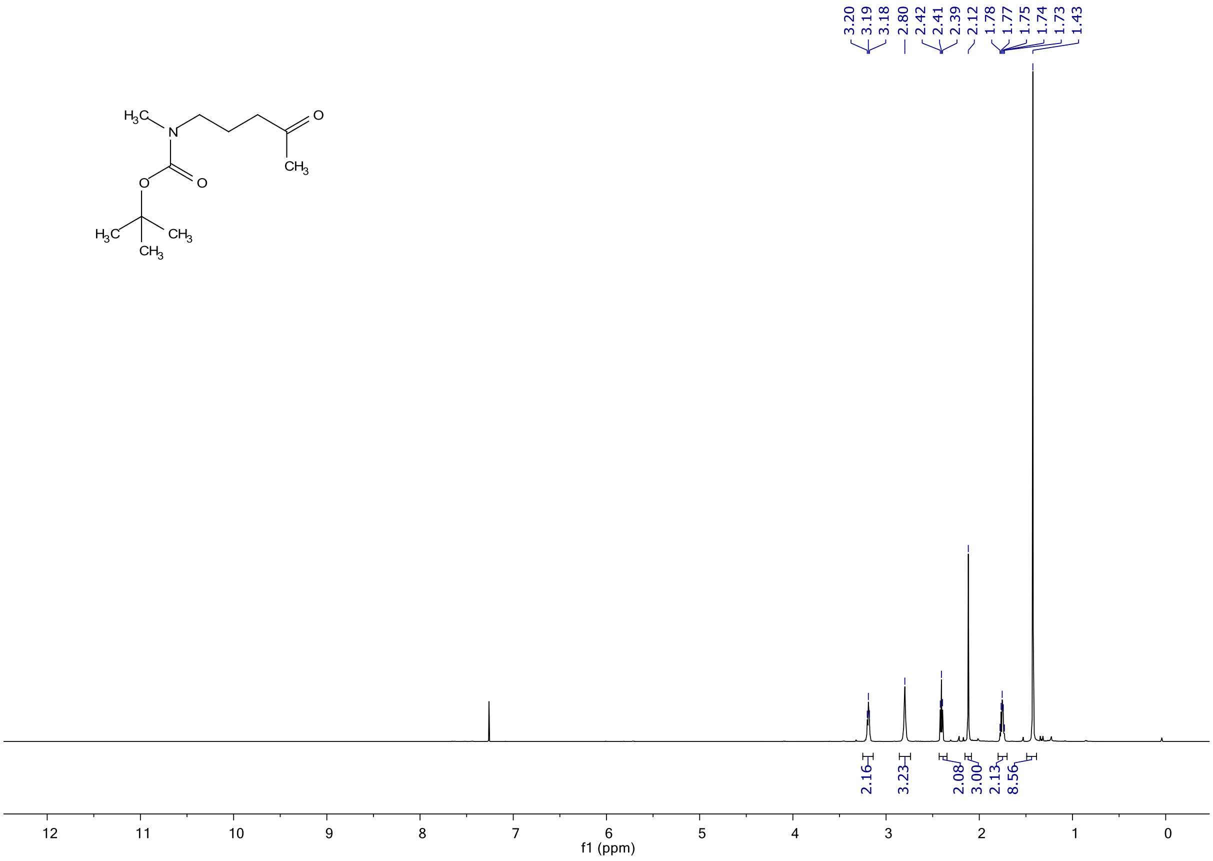

^{13}C-NMR (151 MHz, $CDCl_3$)

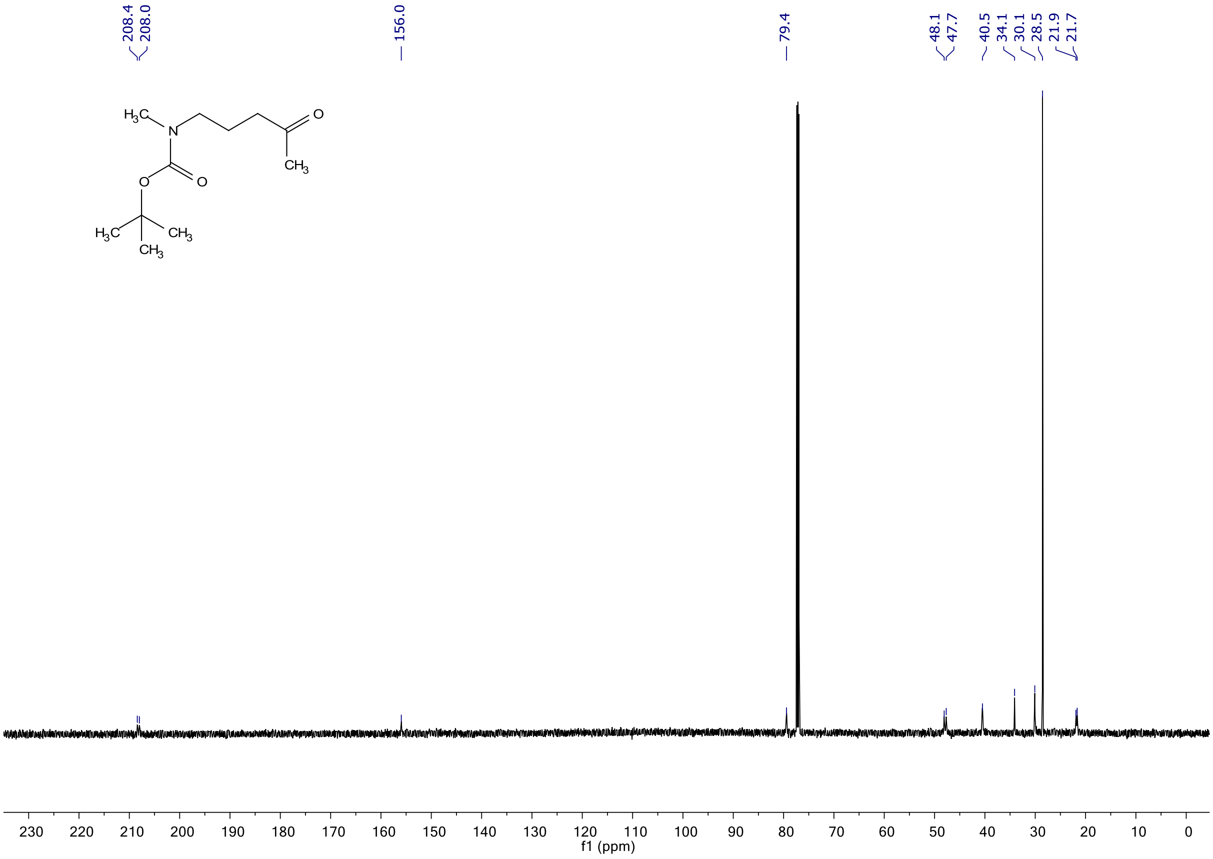

5-(*p*-tolyl)hexan-2-one (3id)

^{1}H-NMR (600 MHz, $CDCl_3$)

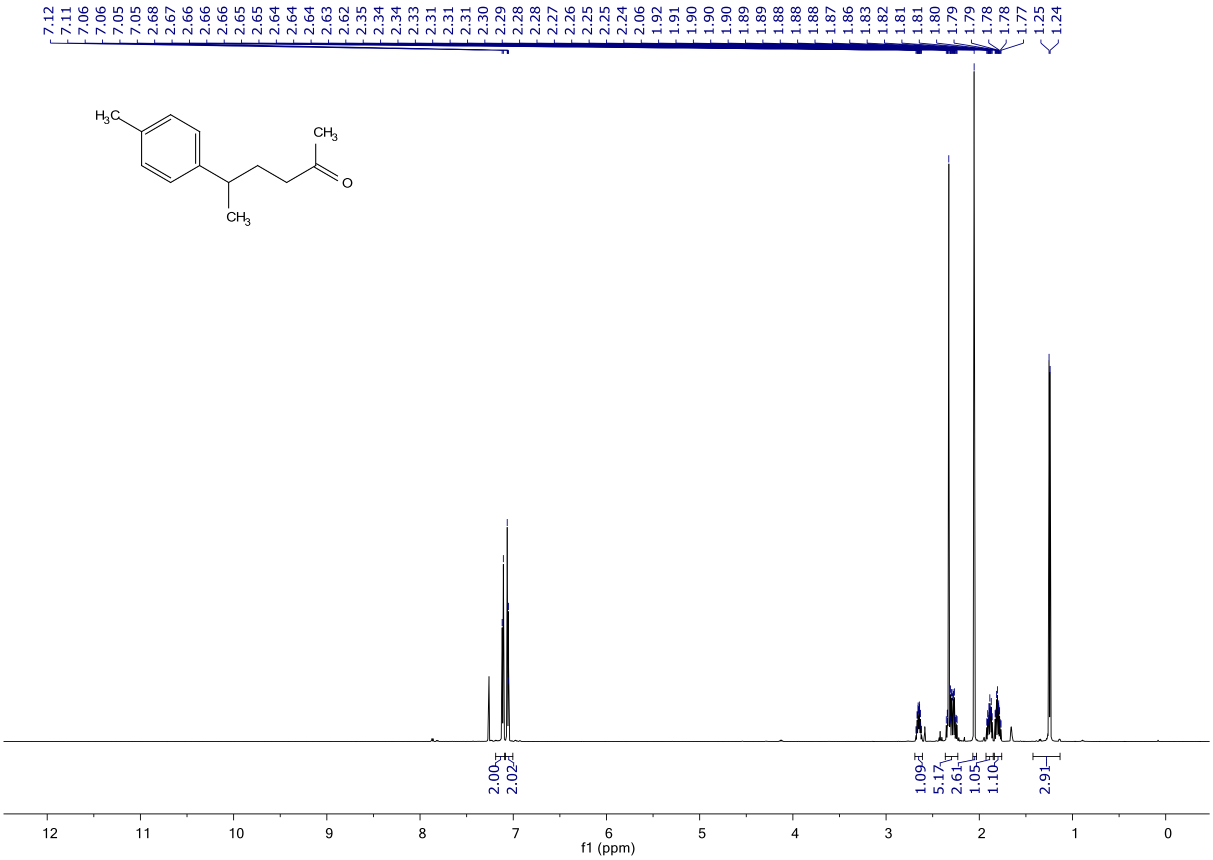

^{13}C-NMR (151 MHz, $CDCl_3$)

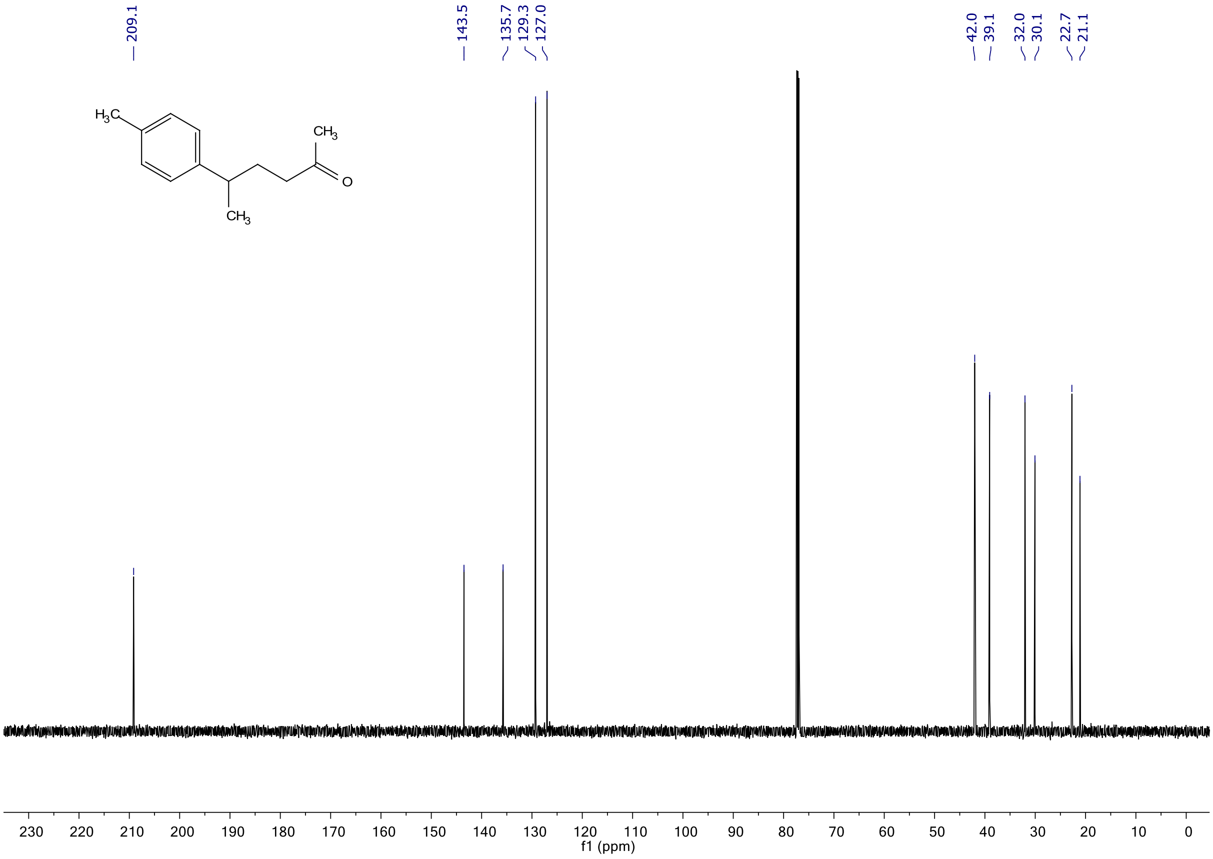

5-phenylhexan-2-one (3jd)

^{1}H-NMR (600 MHz, $CDCl_3$)

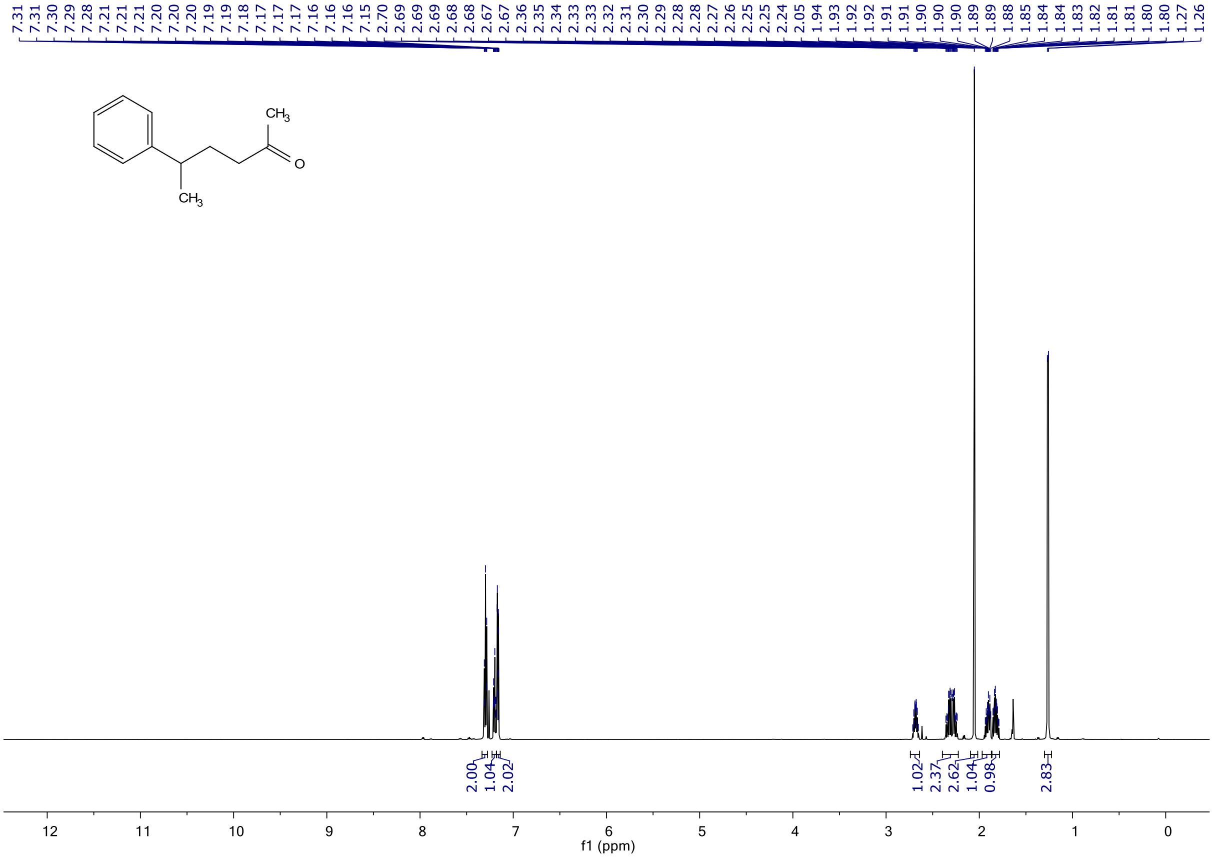

^{13}C-NMR (151 MHz, $CDCl_3$)

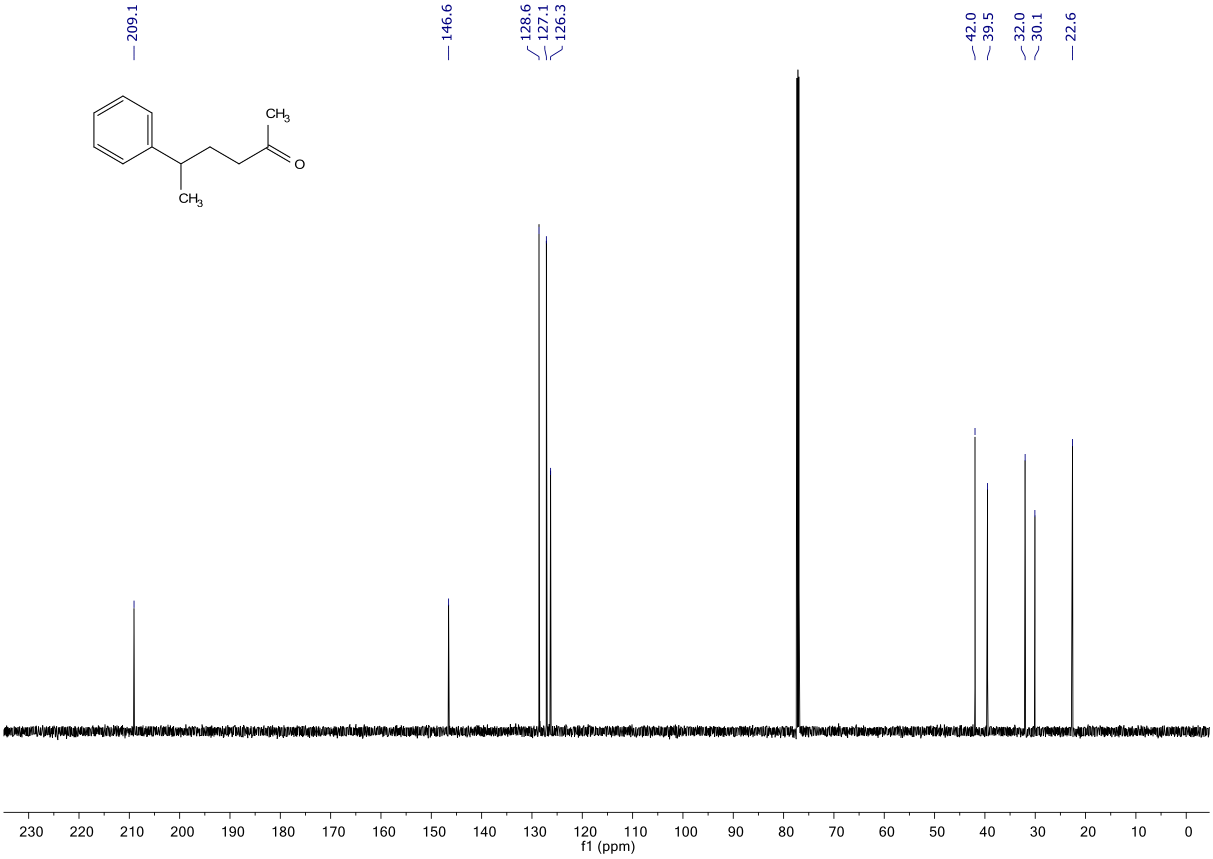

5,6-diphenylhexan-2-one (3kd)

^{1}H-NMR (600 MHz, $CDCl_3$)

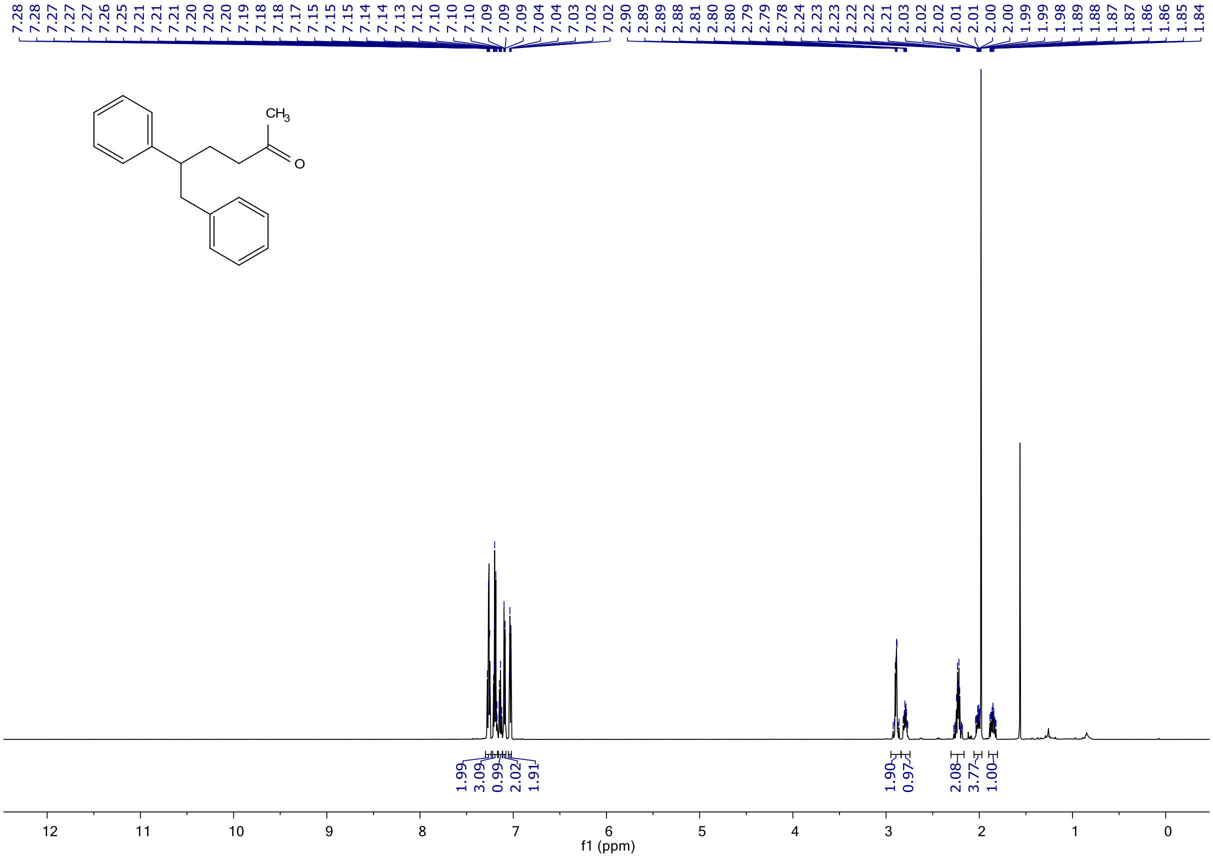

^{13}C-NMR (151 MHz, $CDCl_3$)

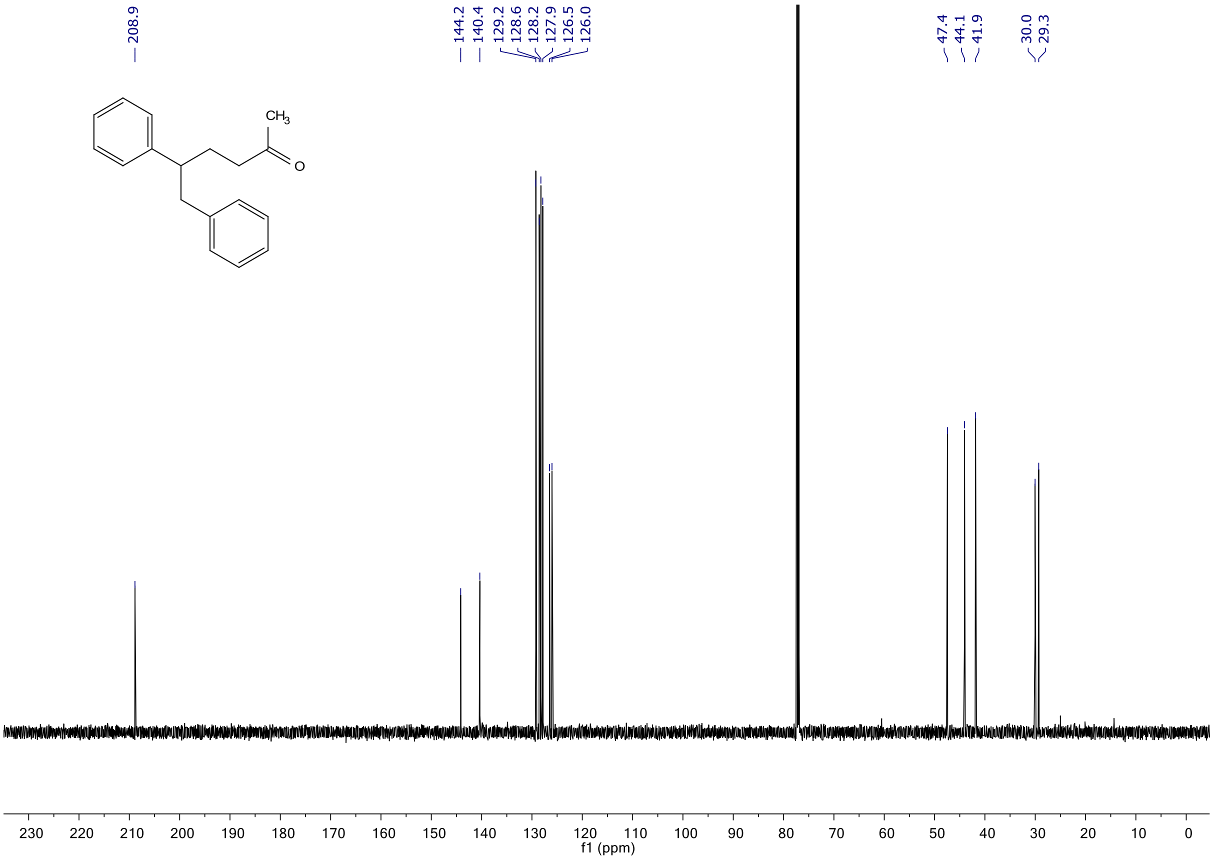

4-(1-(4-chlorophenyl)cyclobutyl)butan-2-one (3nd)
^{1}H-NMR (600 MHz, $CDCl_3$)

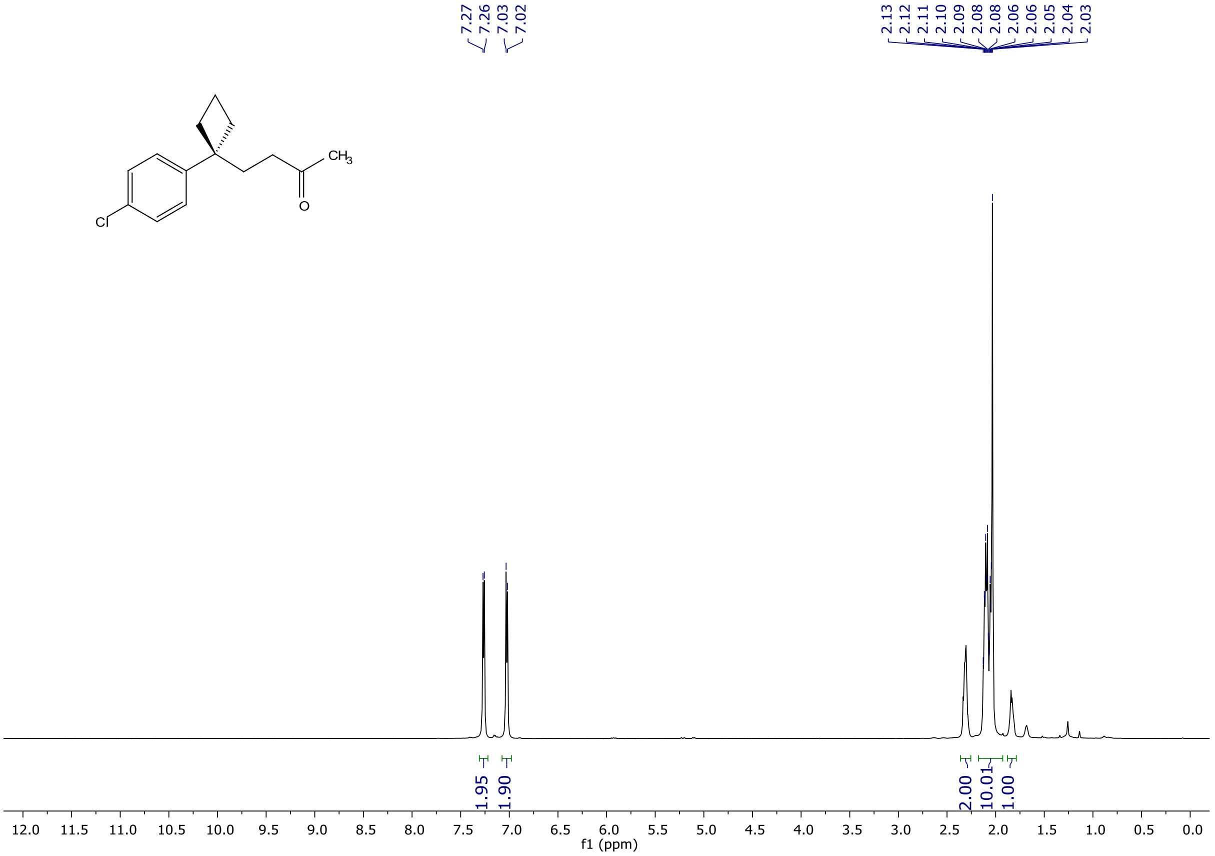

^{13}C-NMR (151 MHz, $CDCl_3$)

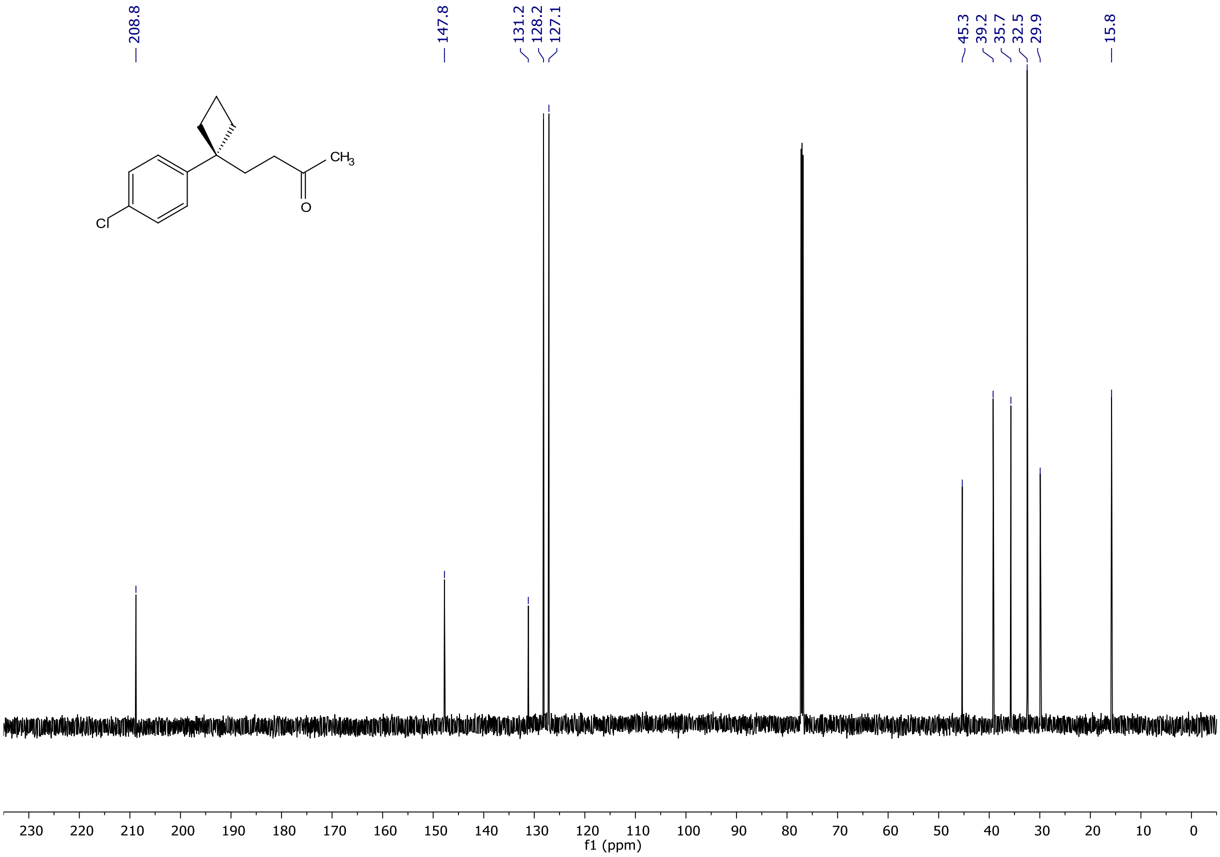

4-(4-(4-chlorophenyl)tetrahydro-2H-pyran-4-yl)butan-2-one (3od)

^{1}H-NMR (600 MHz, $CDCl_3$)

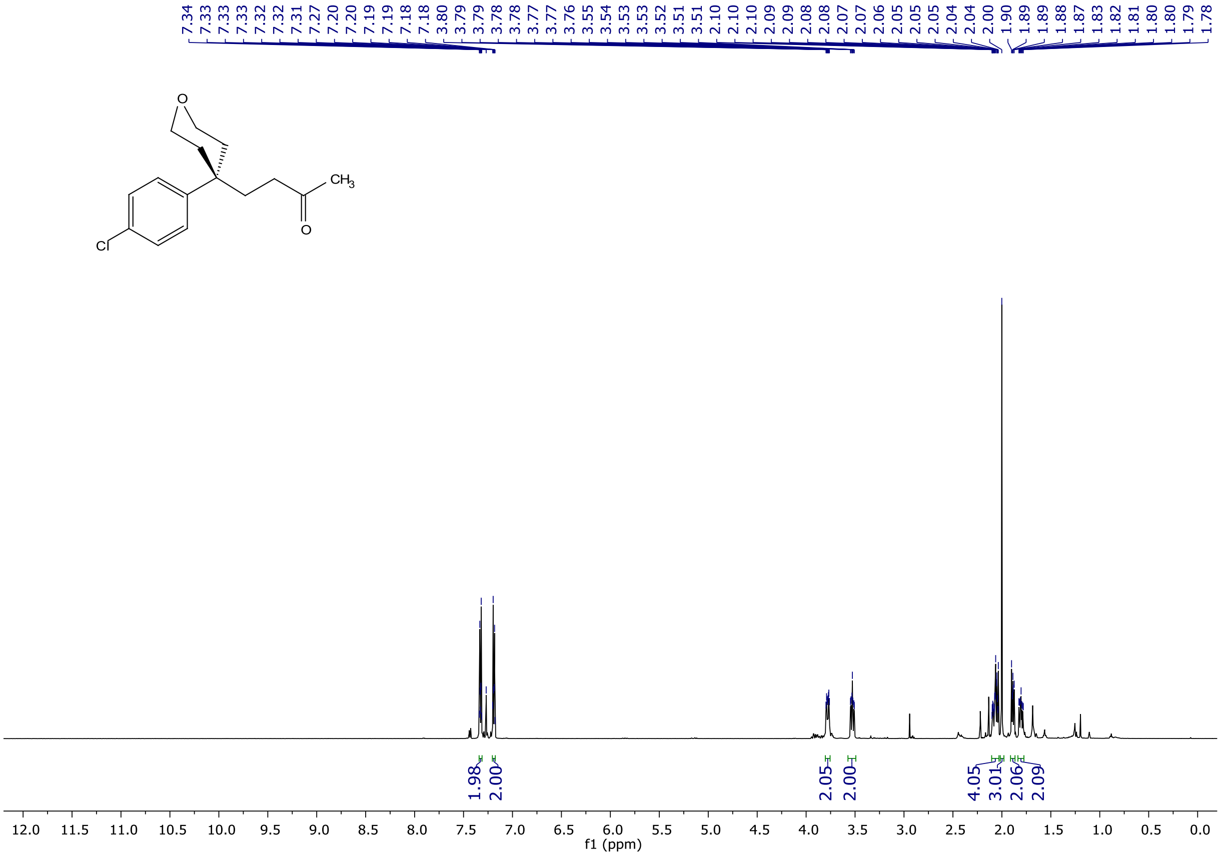

^{13}C-NMR (151 MHz, $CDCl_3$)

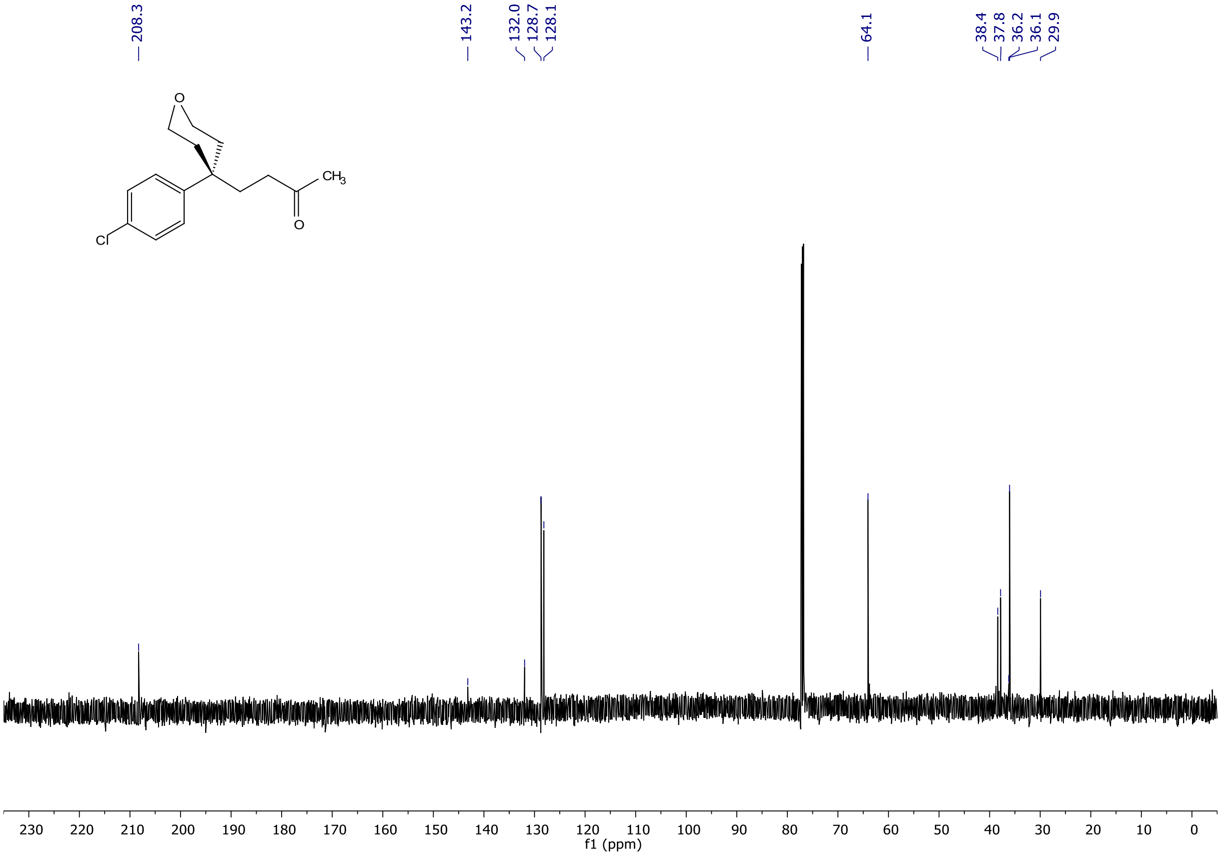

5,5-dimethylhexan-2-one (3pd)
^{1}H-NMR (600 MHz, $CDCl_3$)

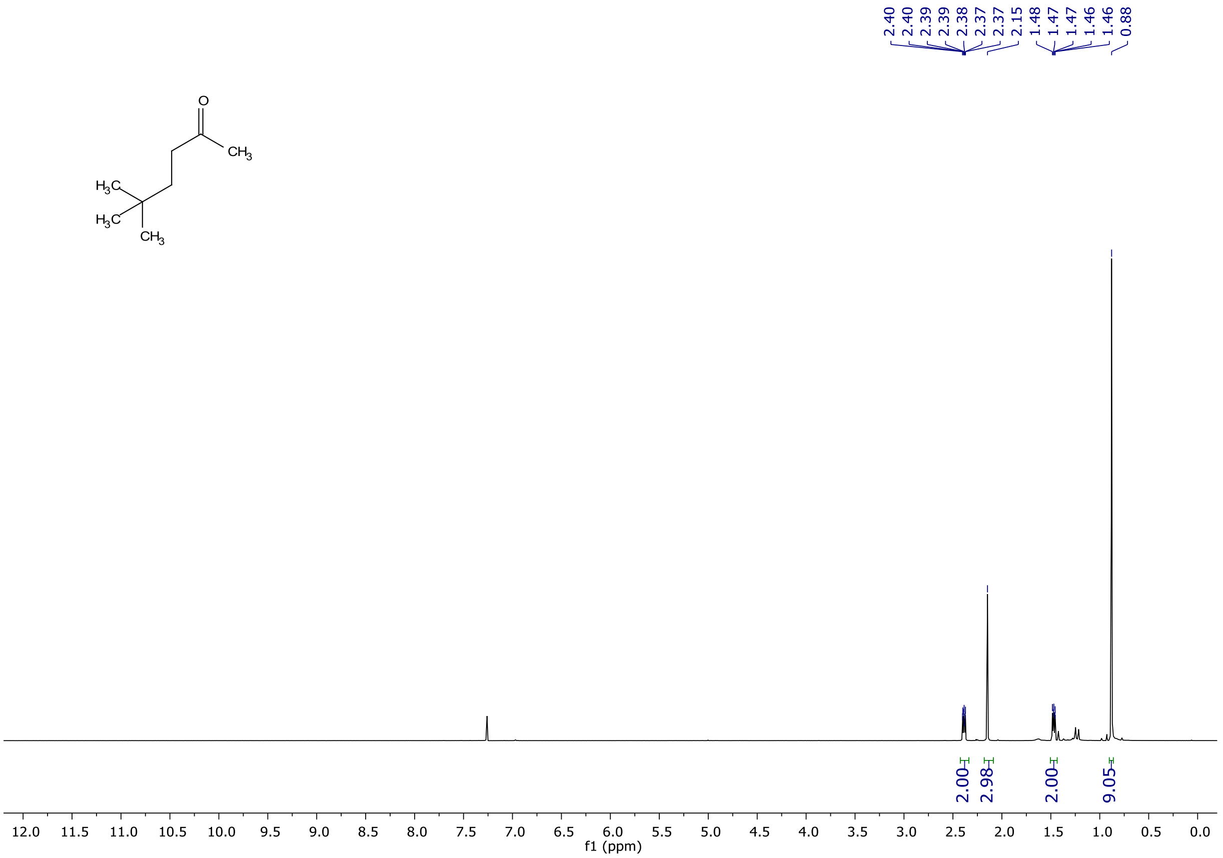

^{13}C-NMR (151 MHz, $CDCl_3$)

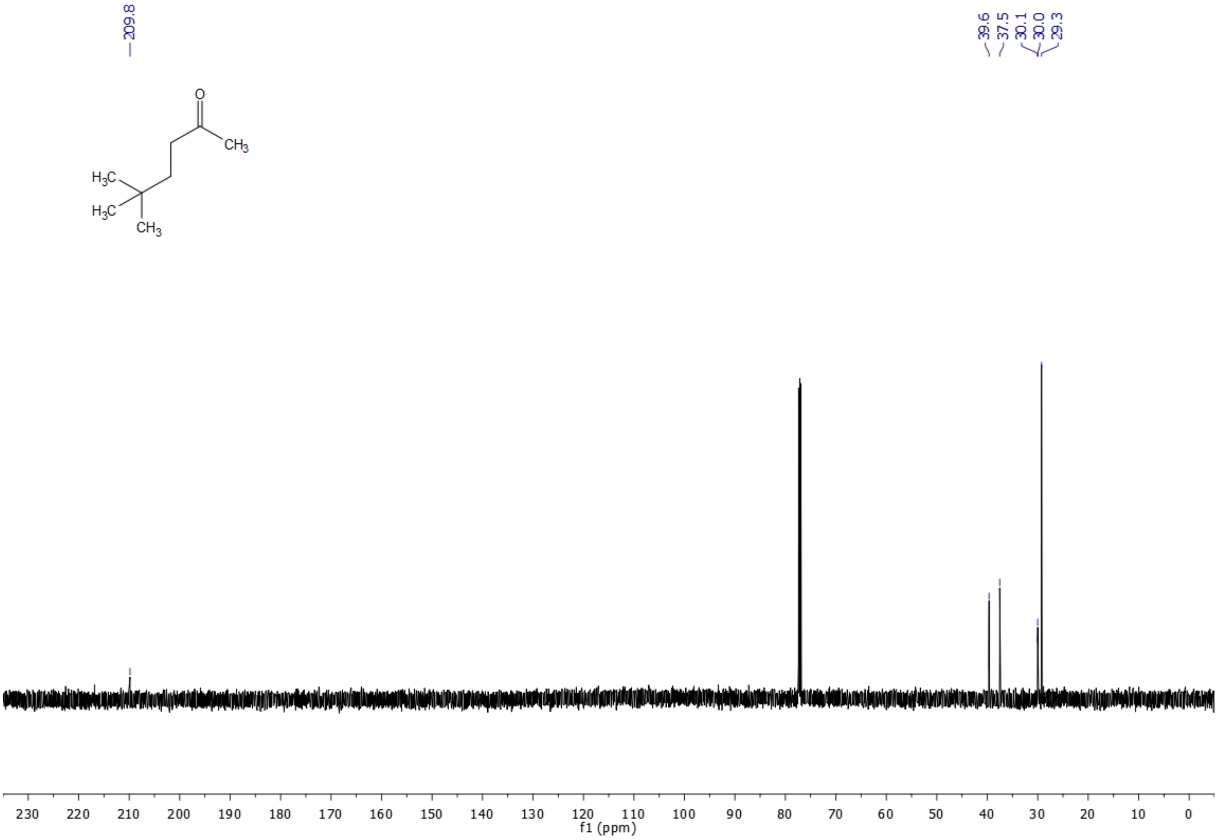

7.2.3 Scope of aryl and alkyl boronic acids

4-(4-methoxyphenyl)butan-2-one (5ad)

^{1}H-NMR (600 MHz, $CDCl_3$)

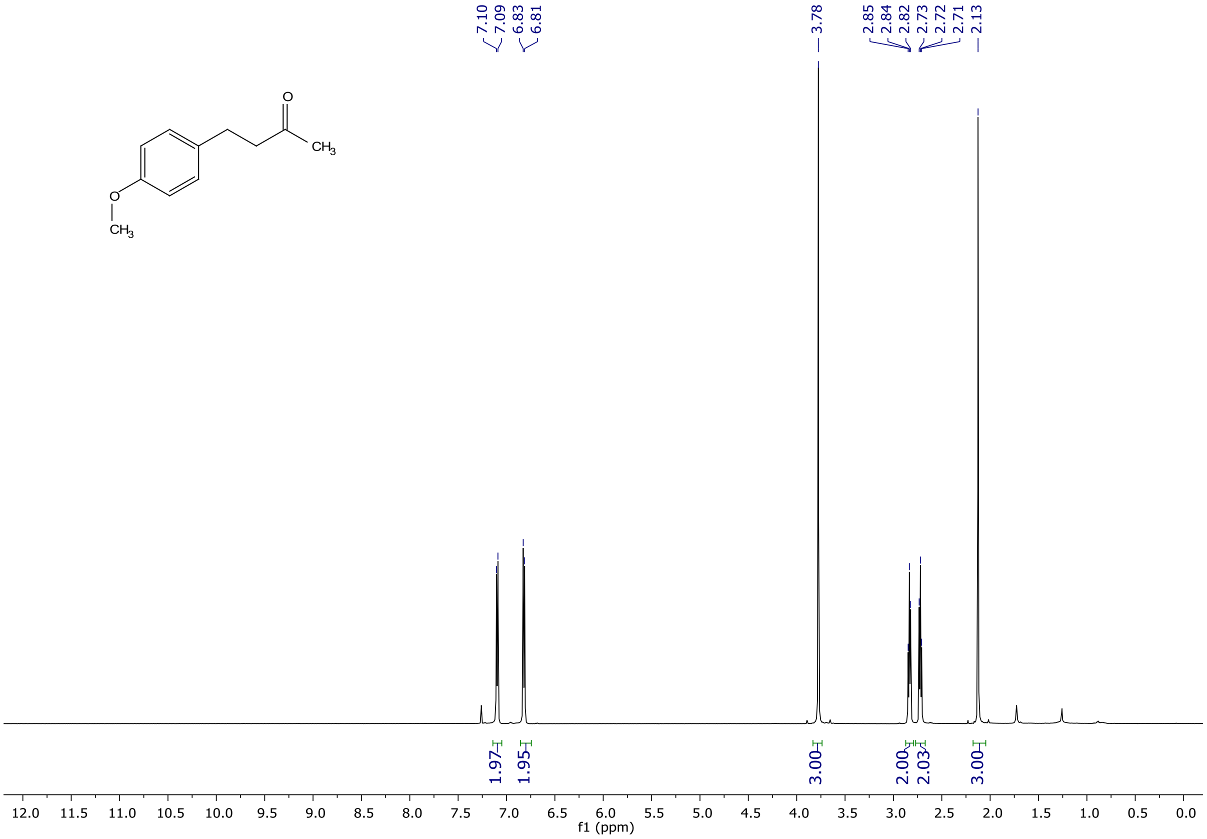

^{13}C-NMR (151 MHz, $CDCl_3$)

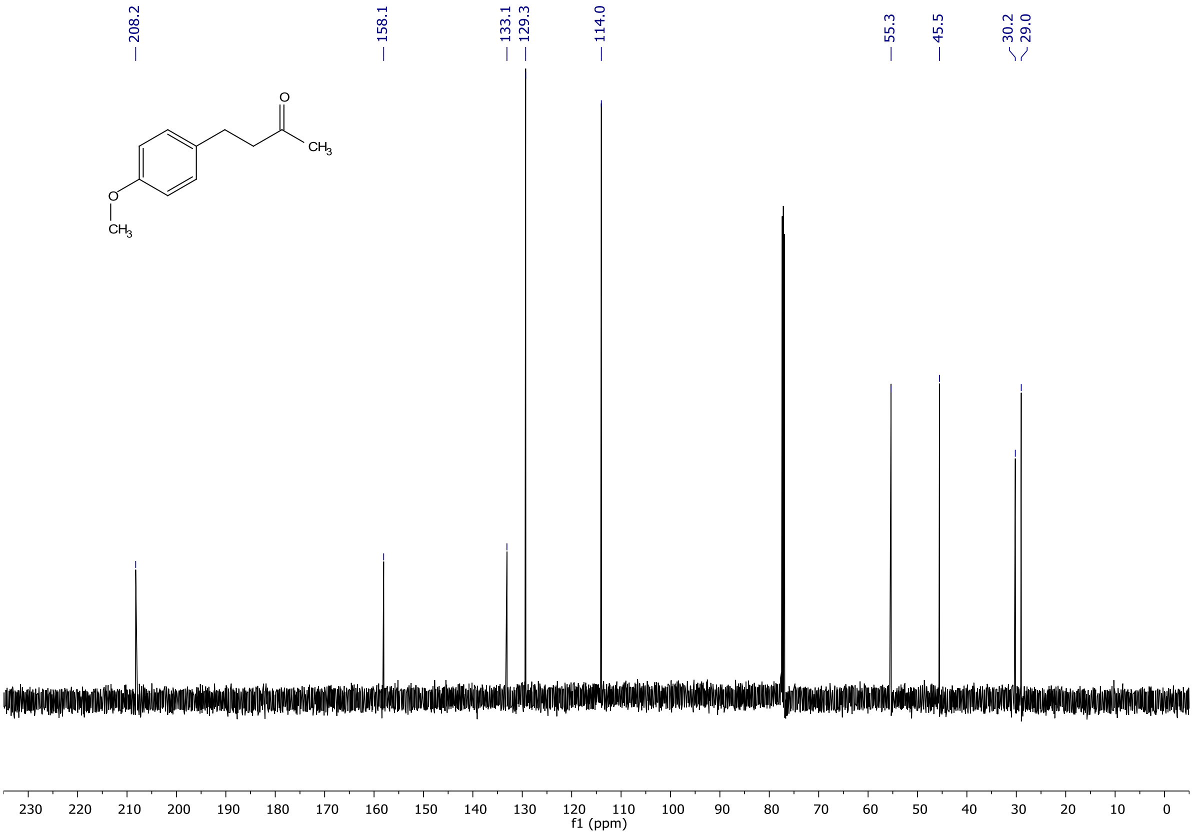

4-(4-(methylthio)phenyl)butan-2-one (5bd)

^{1}H-NMR (600 MHz, $CDCl_3$)

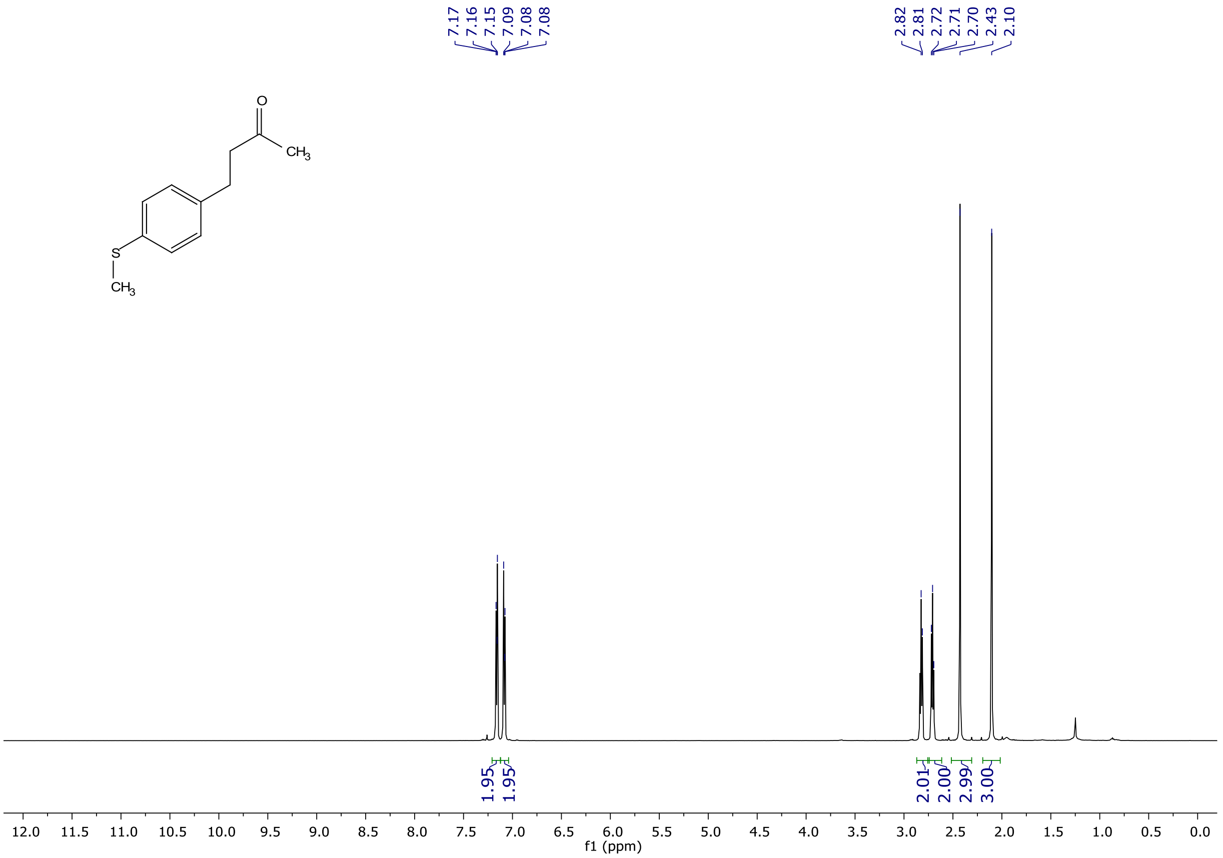

^{13}C-NMR (151 MHz, $CDCl_3$)

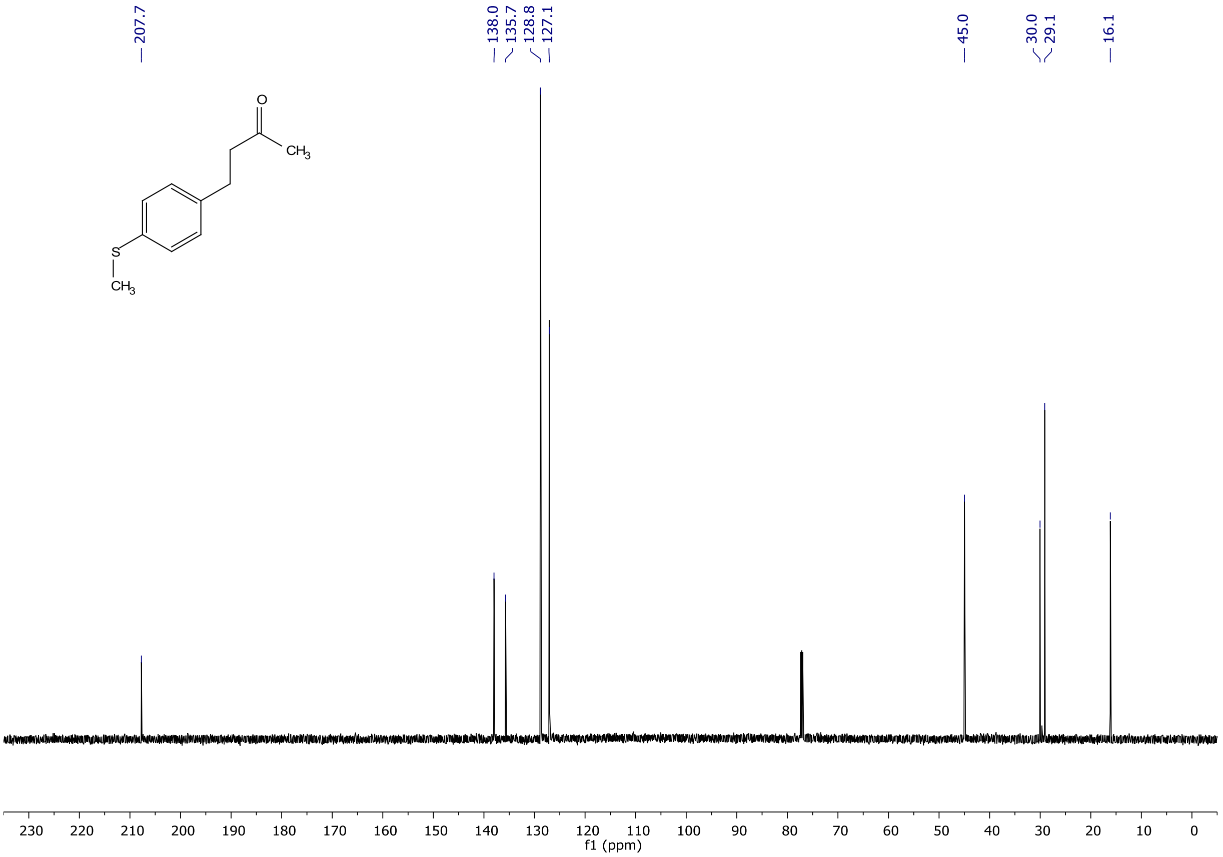

***tert*-butyl (4-(3-oxobutyl)phenyl)carbamate (5cd)**

^{1}H-NMR (600 MHz, CDCl$_3$)

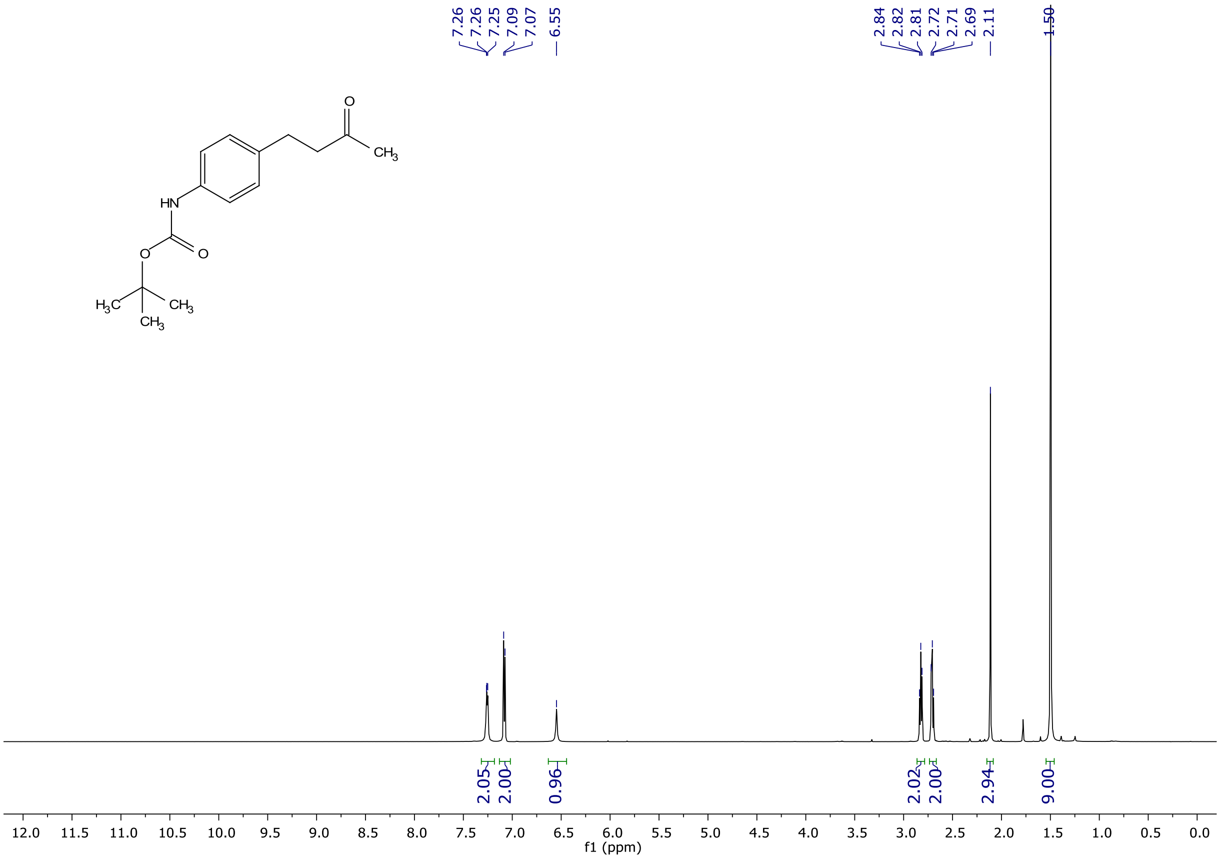

^{13}C-NMR (151 MHz, CDCl$_3$)

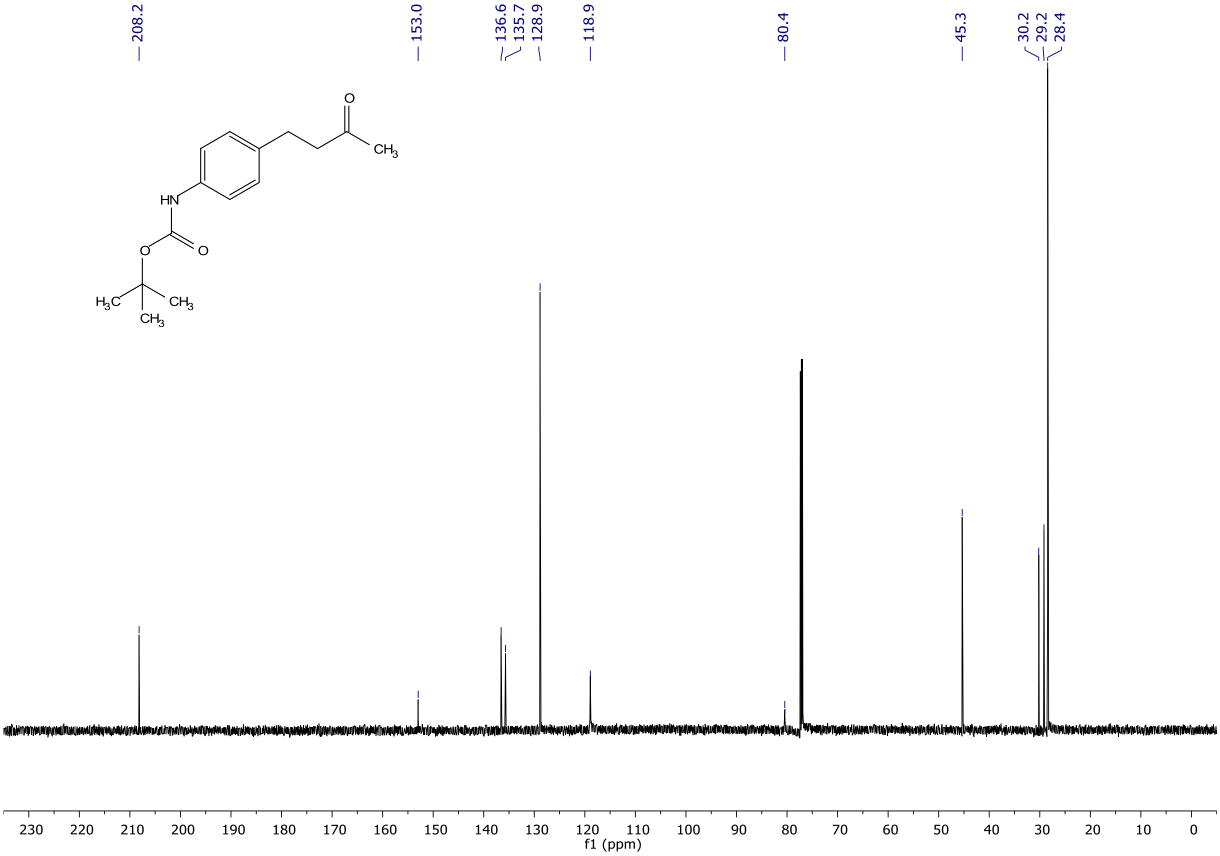

N-(4-(3-oxobutyl)phenyl)acetamide (5dd)

^{1}H-NMR (600 MHz, $CDCl_3$)

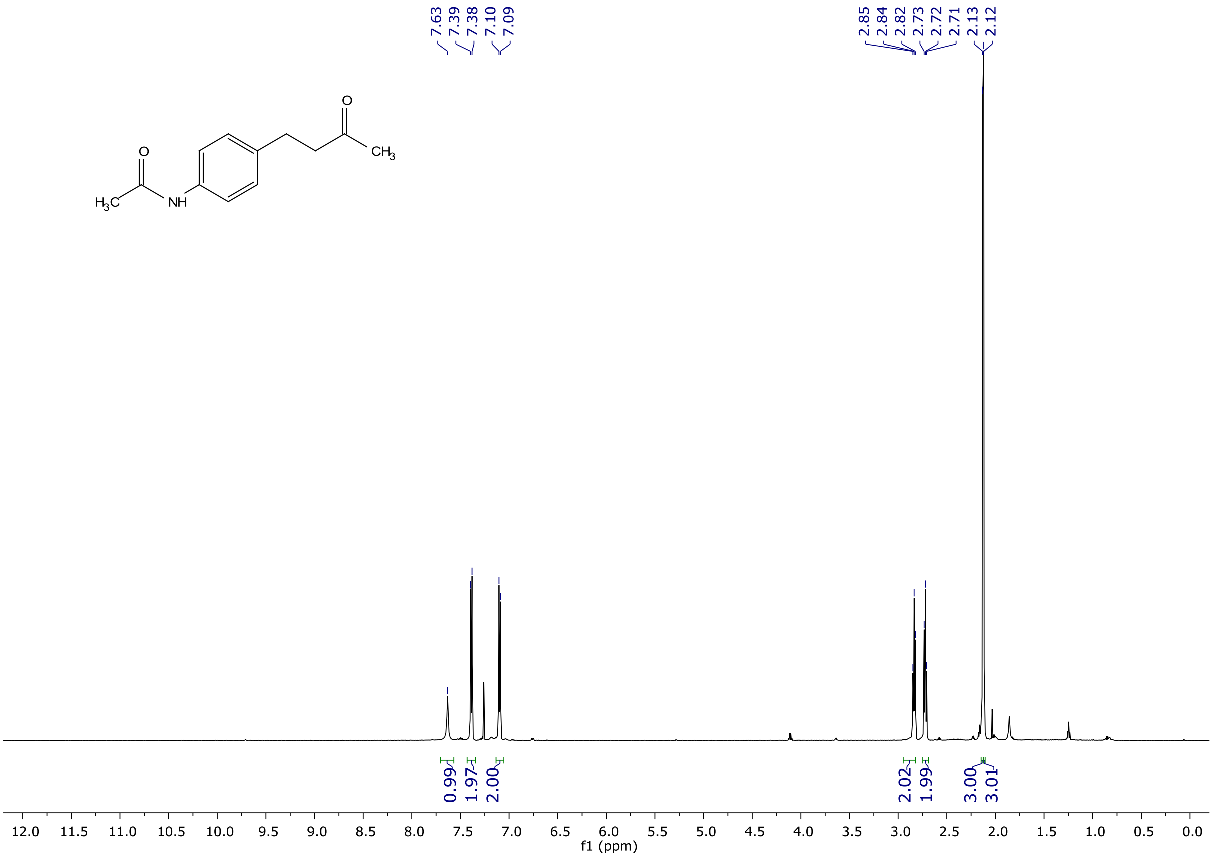

^{13}C-NMR (151 MHz, $CDCl_3$)

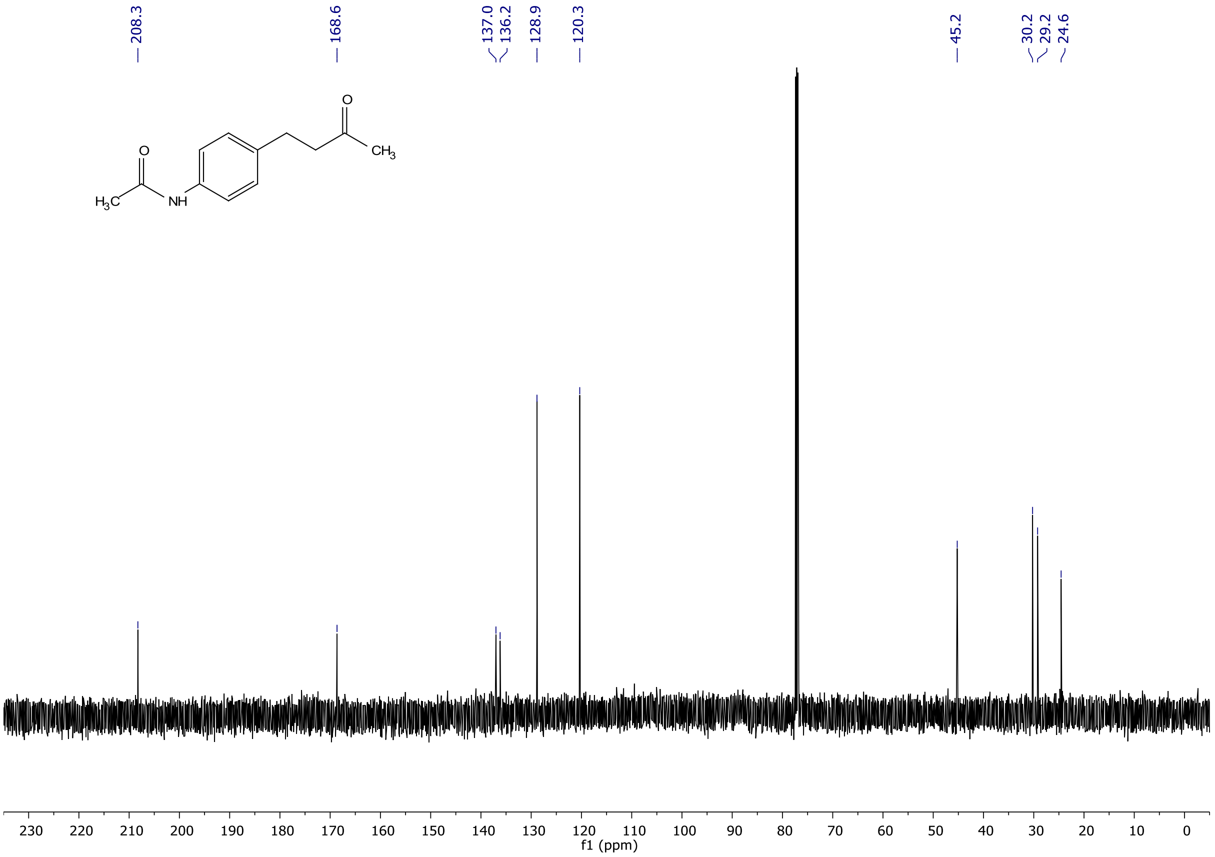

N-(3-(3-oxobutyl)phenyl)acetamide (5ed)

^{1}H-NMR (600 MHz, $CDCl_3$)

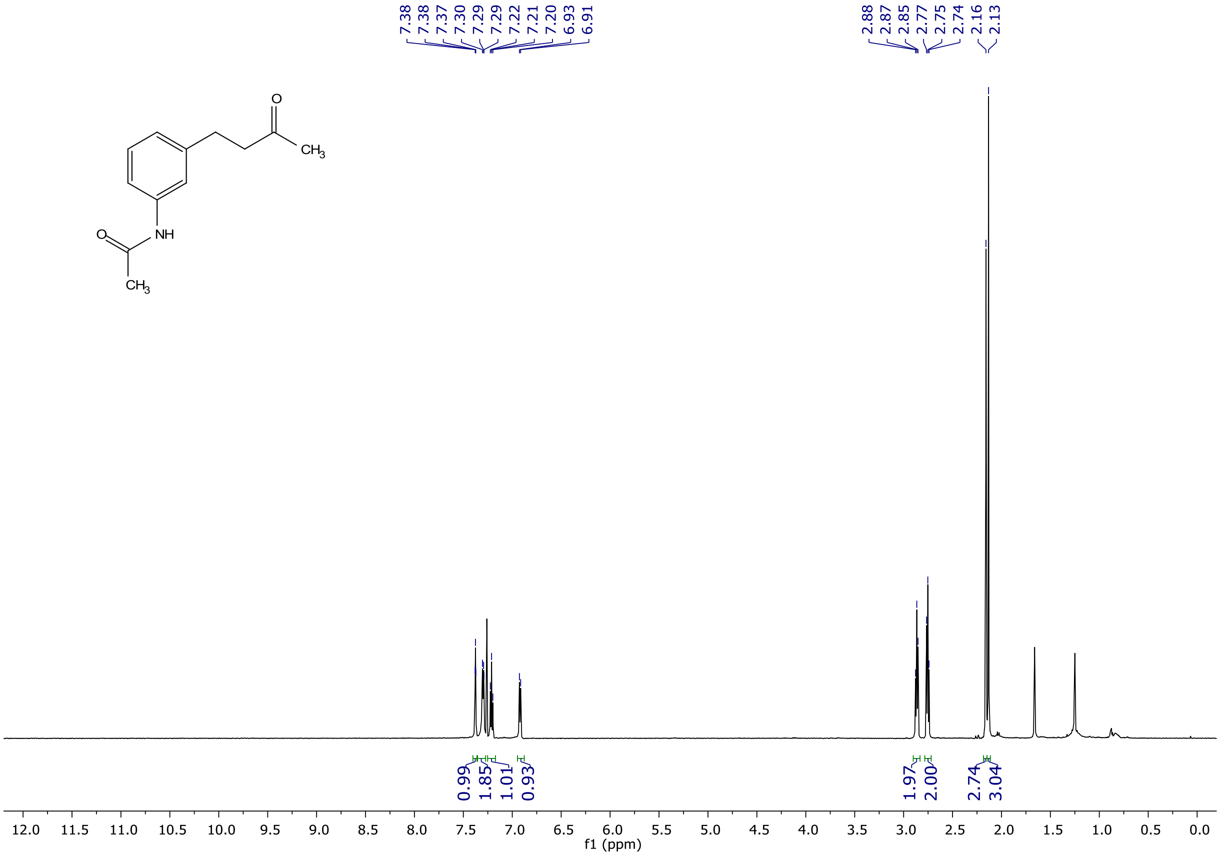

^{13}C-NMR (151 MHz, $CDCl_3$)

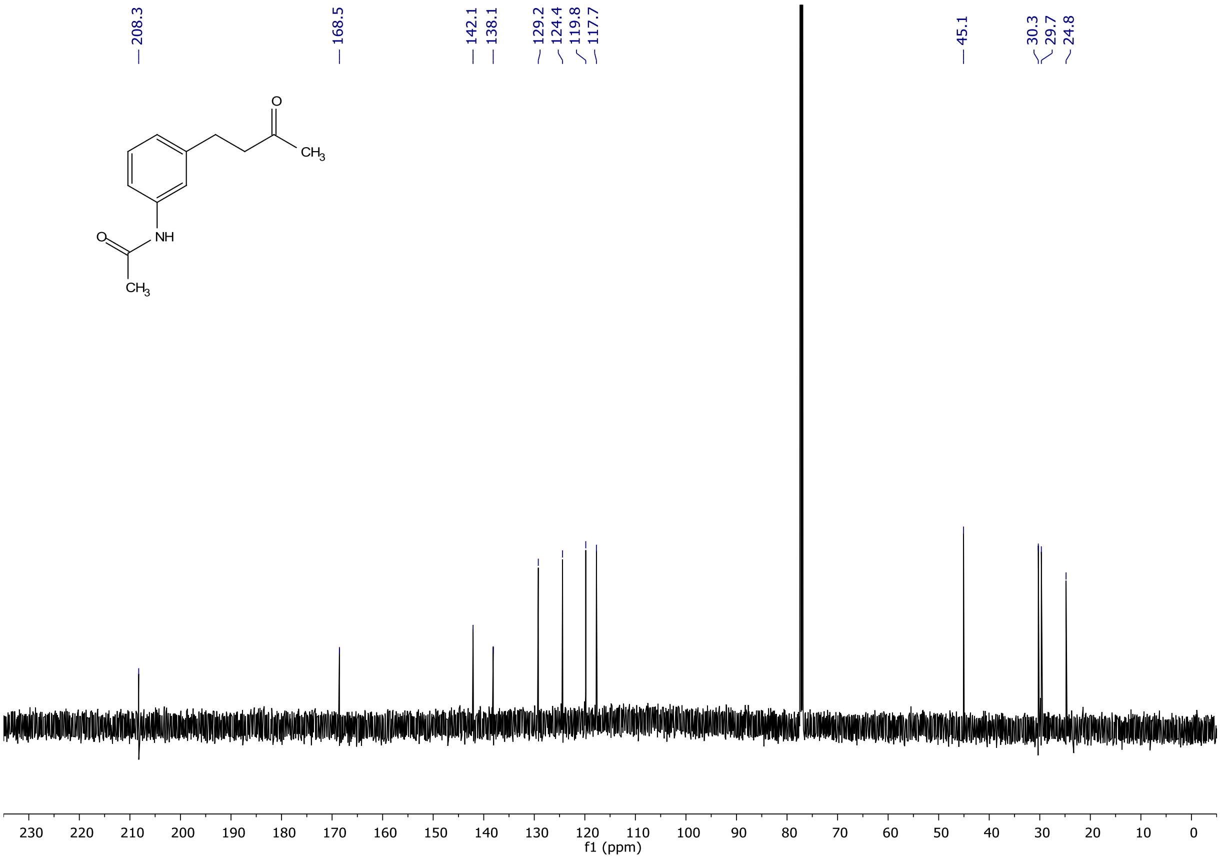

4-(2-methoxyphenyl)butan-2-one (5fd)

^{1}H-NMR (600 MHz, $CDCl_3$)

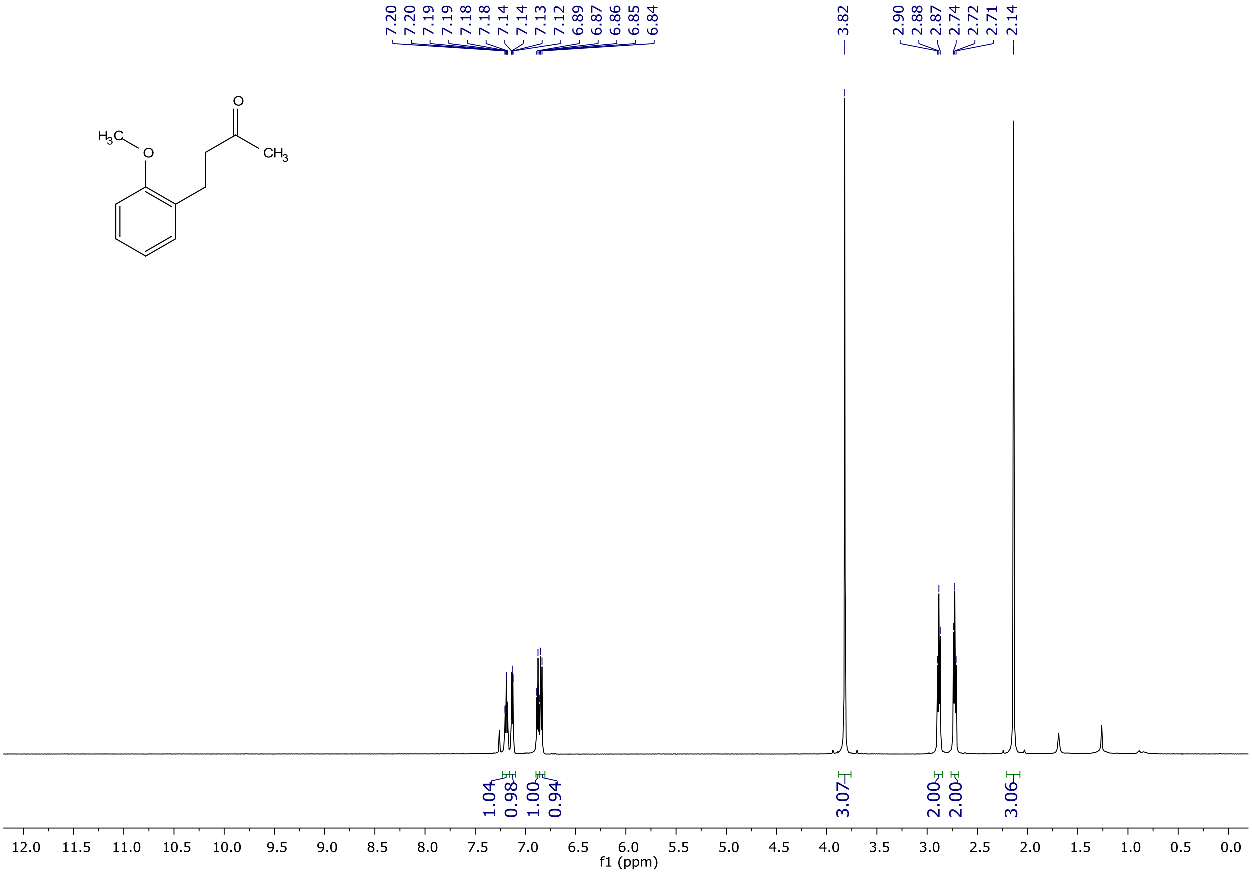

^{13}C-NMR (151 MHz, $CDCl_3$)

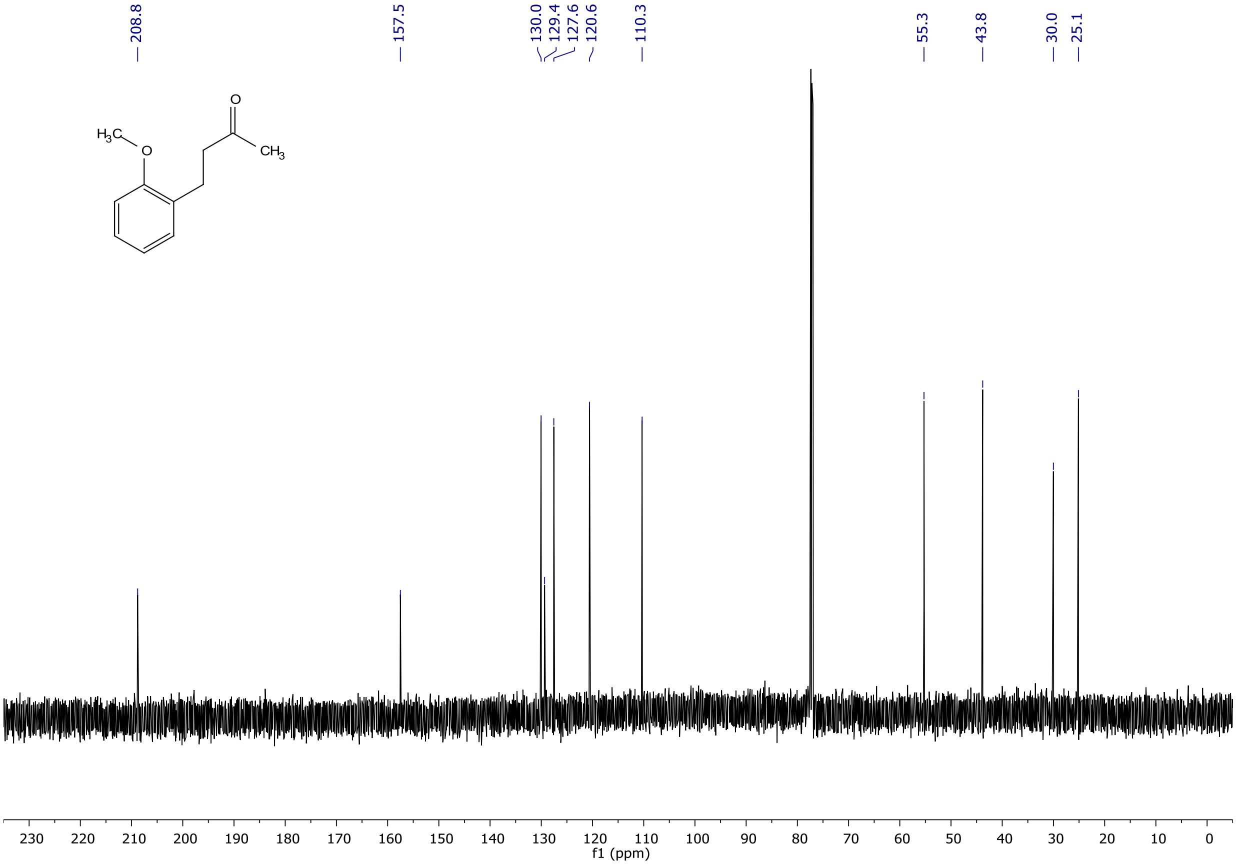

4-(2-(methylthio)phenyl)butan-2-one (5gd)

^{1}H-NMR (600 MHz, $CDCl_3$)

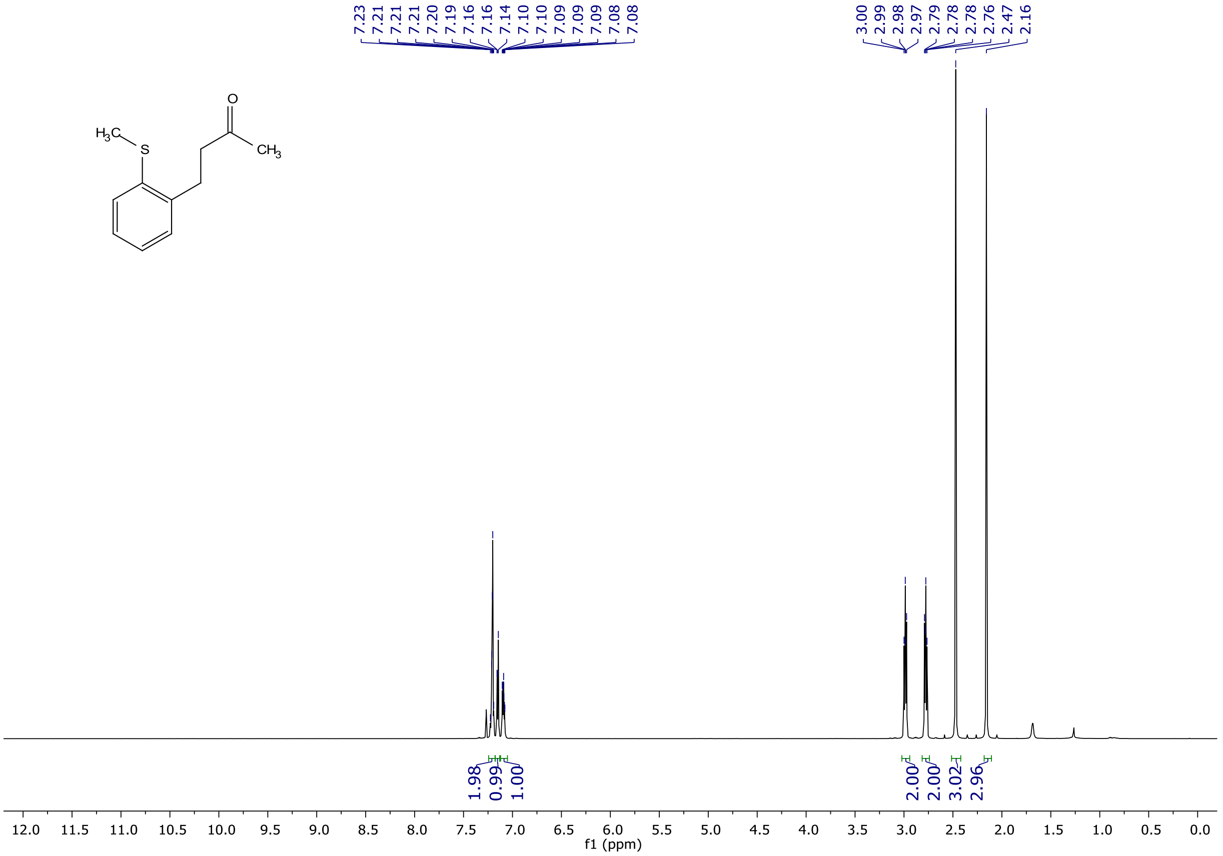

^{13}C-NMR (151 MHz, $CDCl_3$)

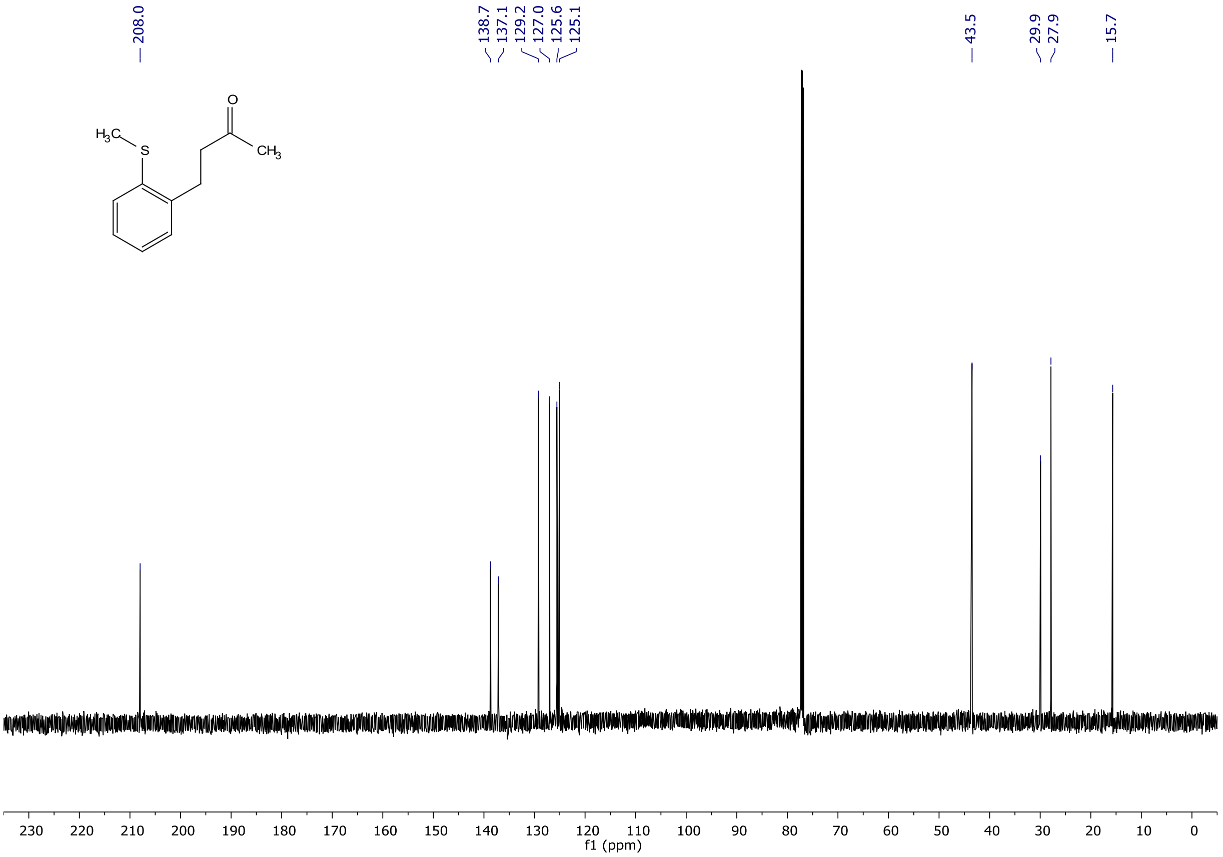

4-(benzo[d][1,3]dioxol-5-yl)butan-2-one (5hd)

^{1}H-NMR (600 MHz, $CDCl_3$)

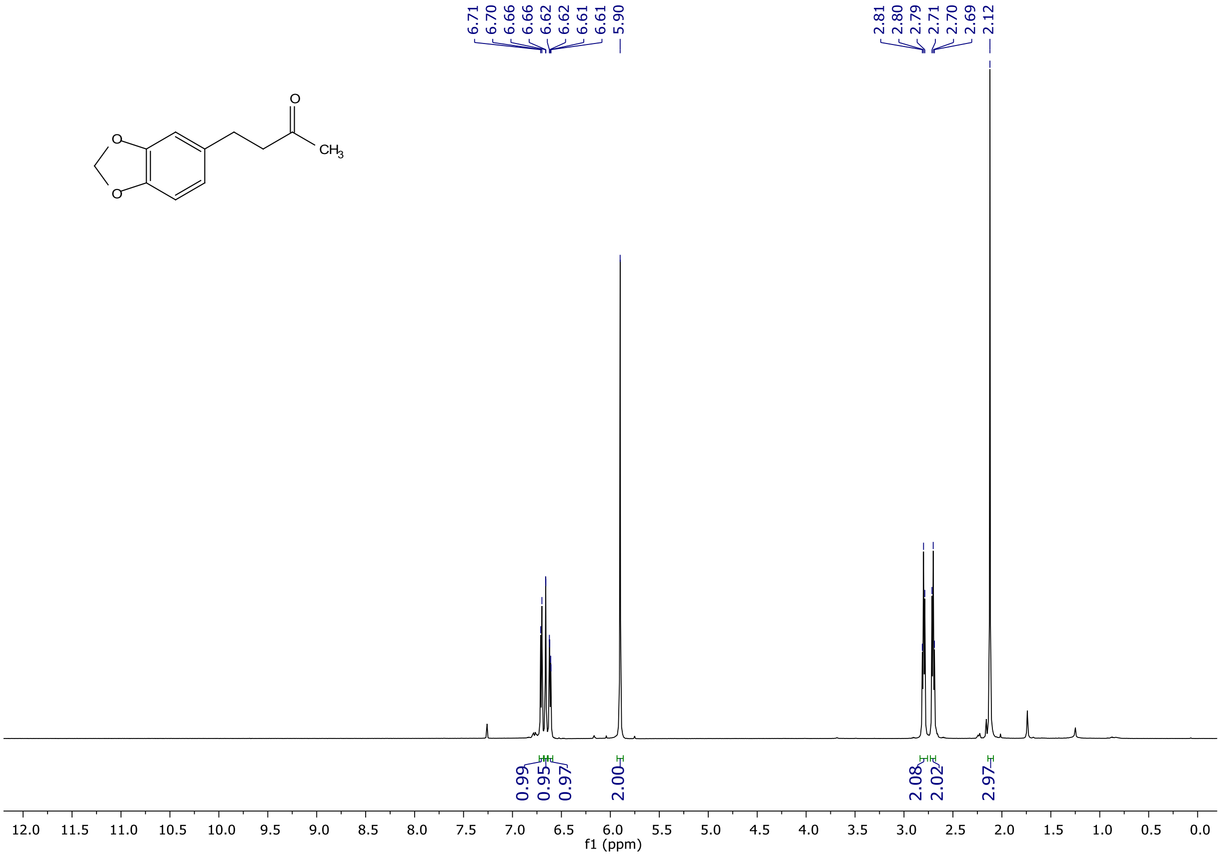

^{13}C-NMR (151 MHz, $CDCl_3$)

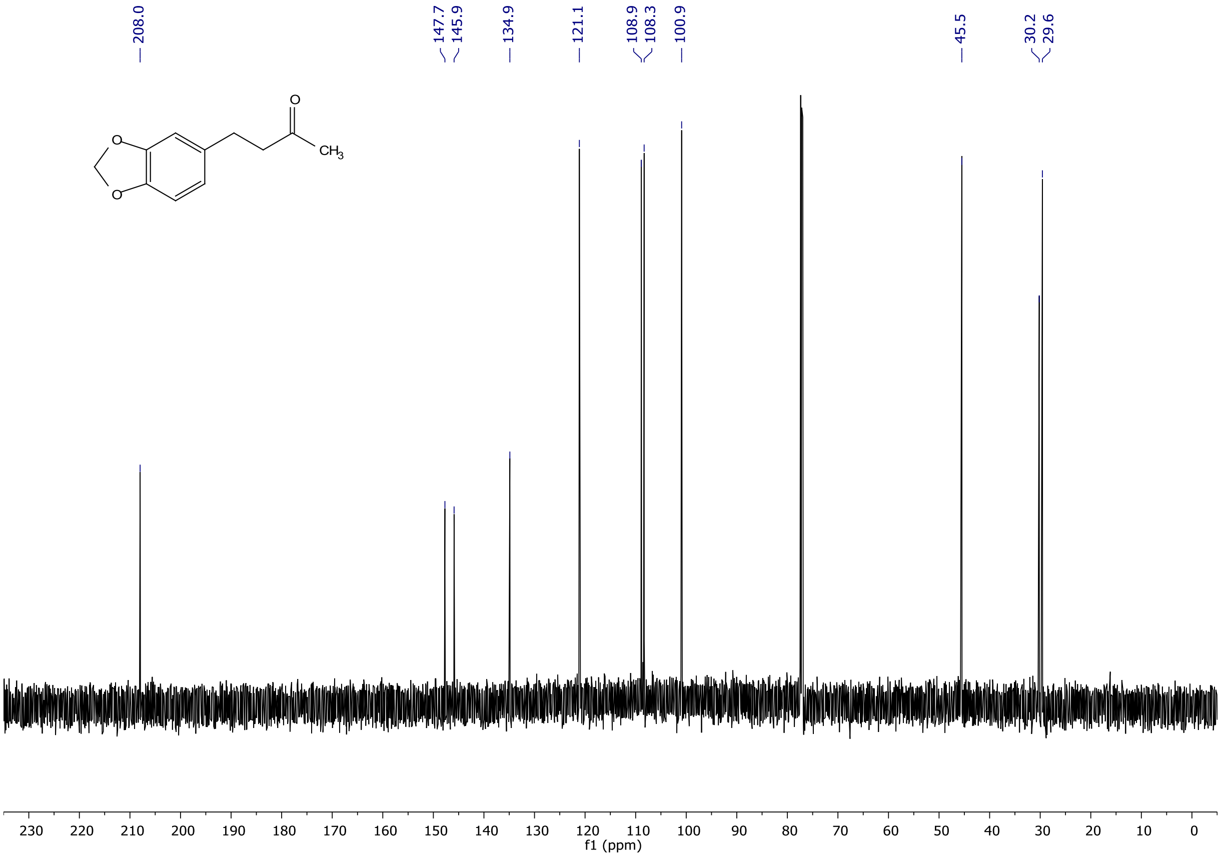

4-(2,3-dihydrobenzo[b][1,4]dioxin-6-yl)butan-2-one (5id)

^{1}H-NMR (600 MHz, $CDCl_3$)

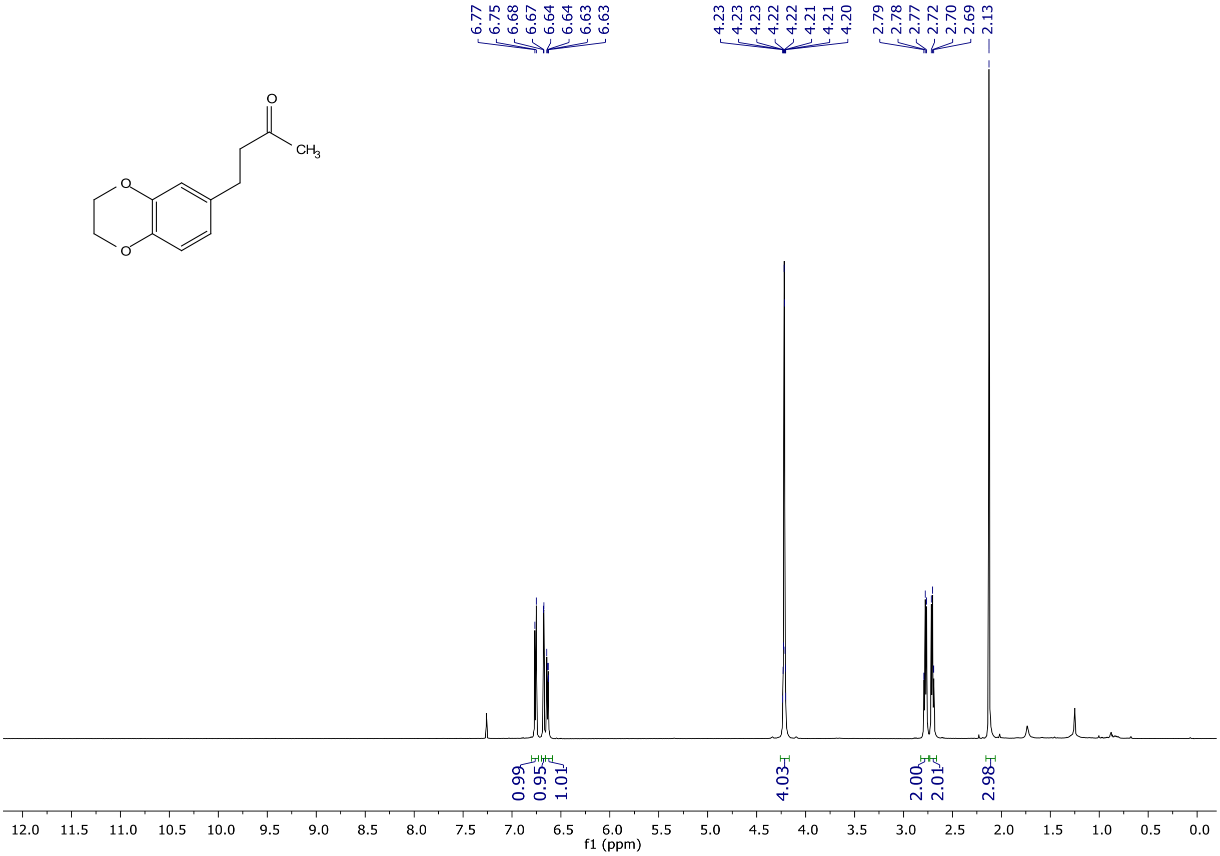

^{13}C-NMR (151 MHz, $CDCl_3$)

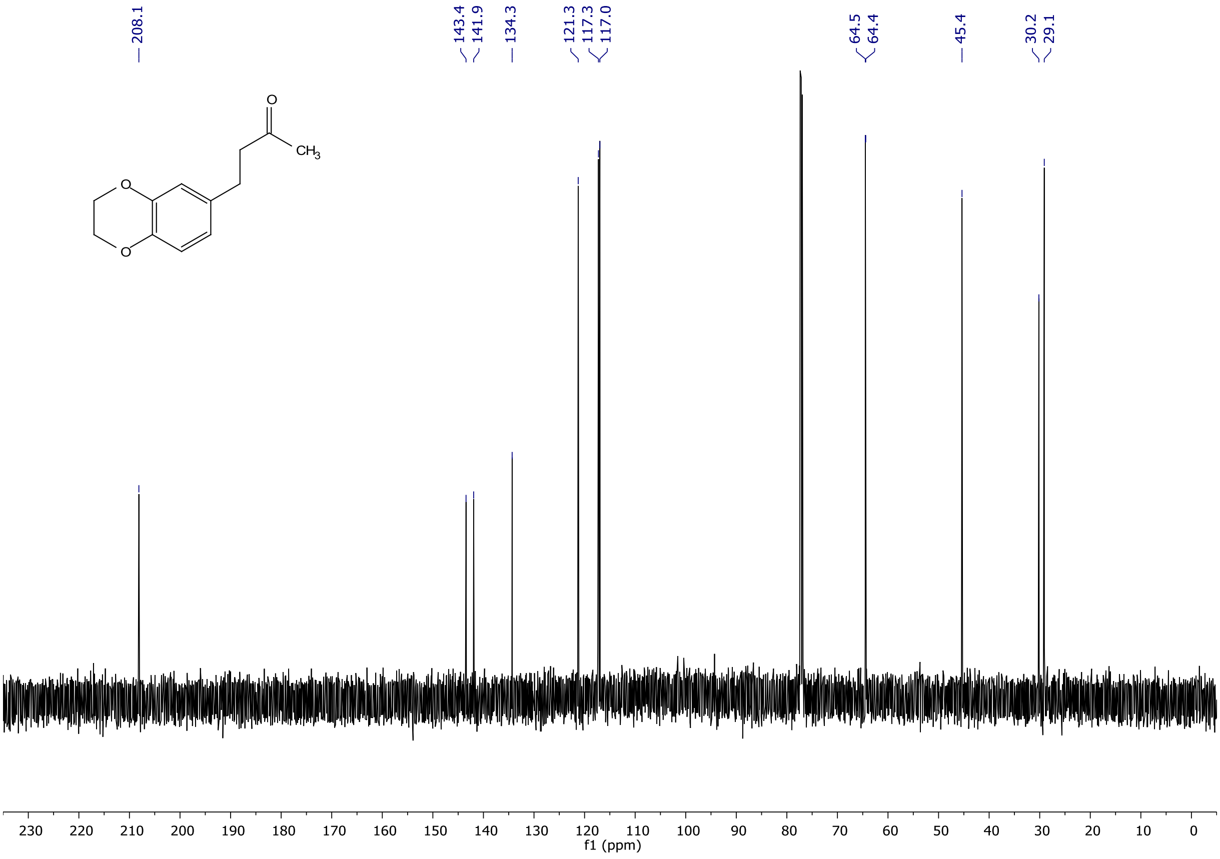

4-(1H-indol-5-yl)butan-2-one (5jd)

^{1}H-NMR (600 MHz, $CDCl_3$)

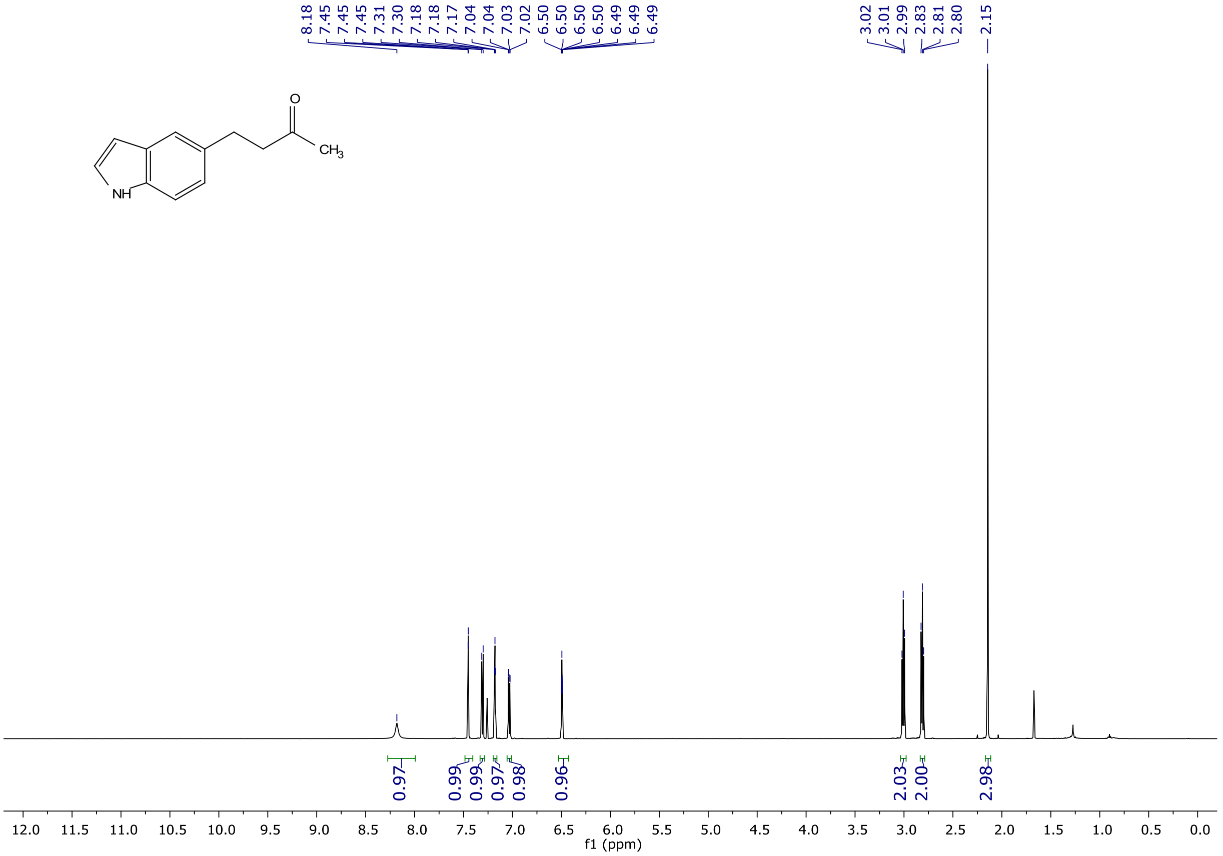

^{13}C-NMR (151 MHz, $CDCl_3$)

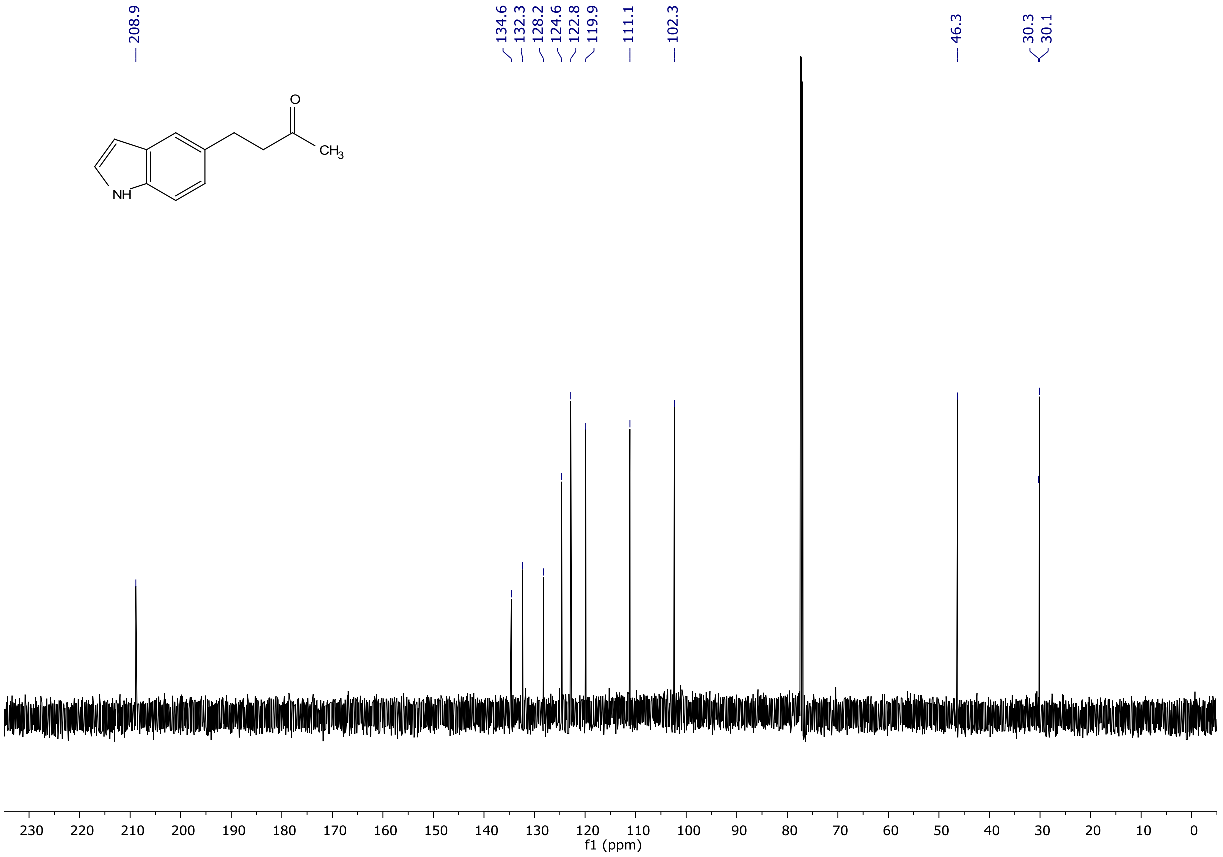

4-(1H-indol-6-yl)butan-2-one (5kd)

^{1}H-NMR (600 MHz, $CDCl_3$)

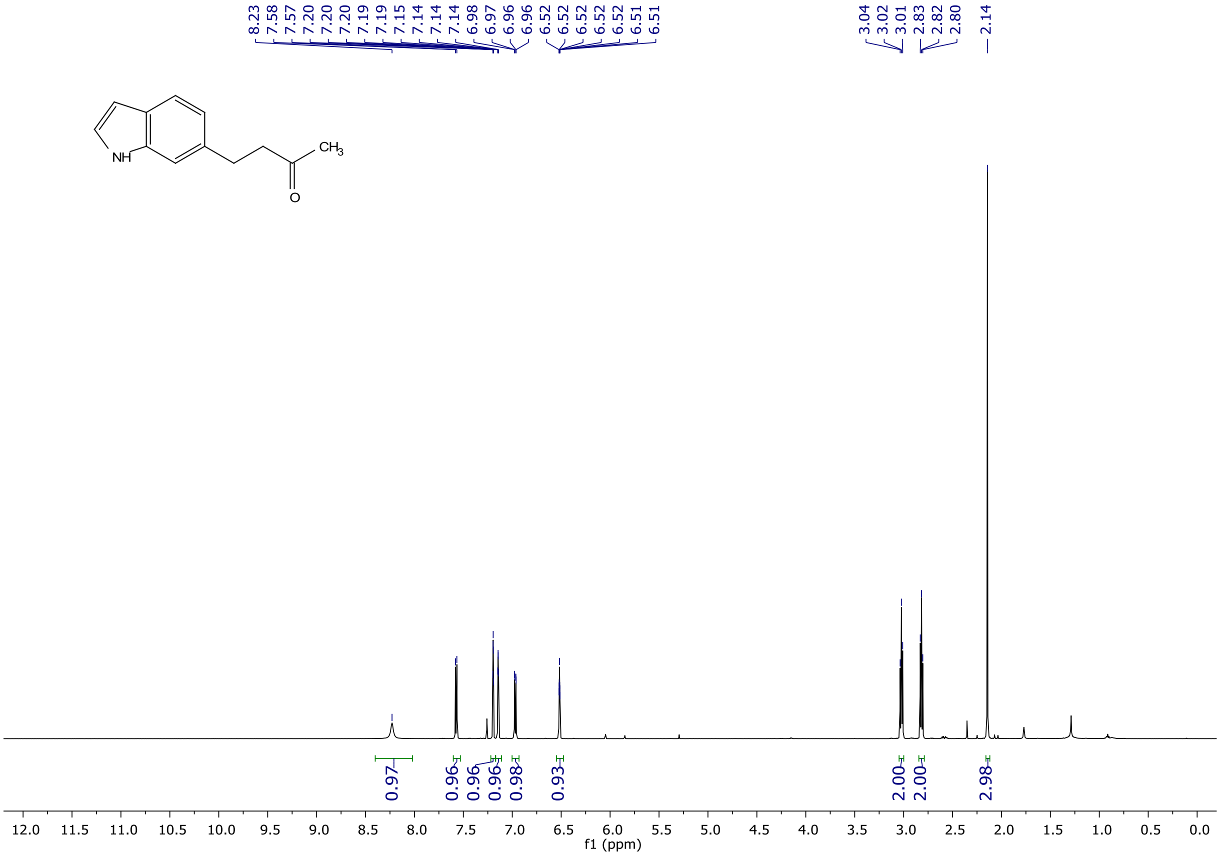

^{13}C-NMR (151 MHz, $CDCl_3$)

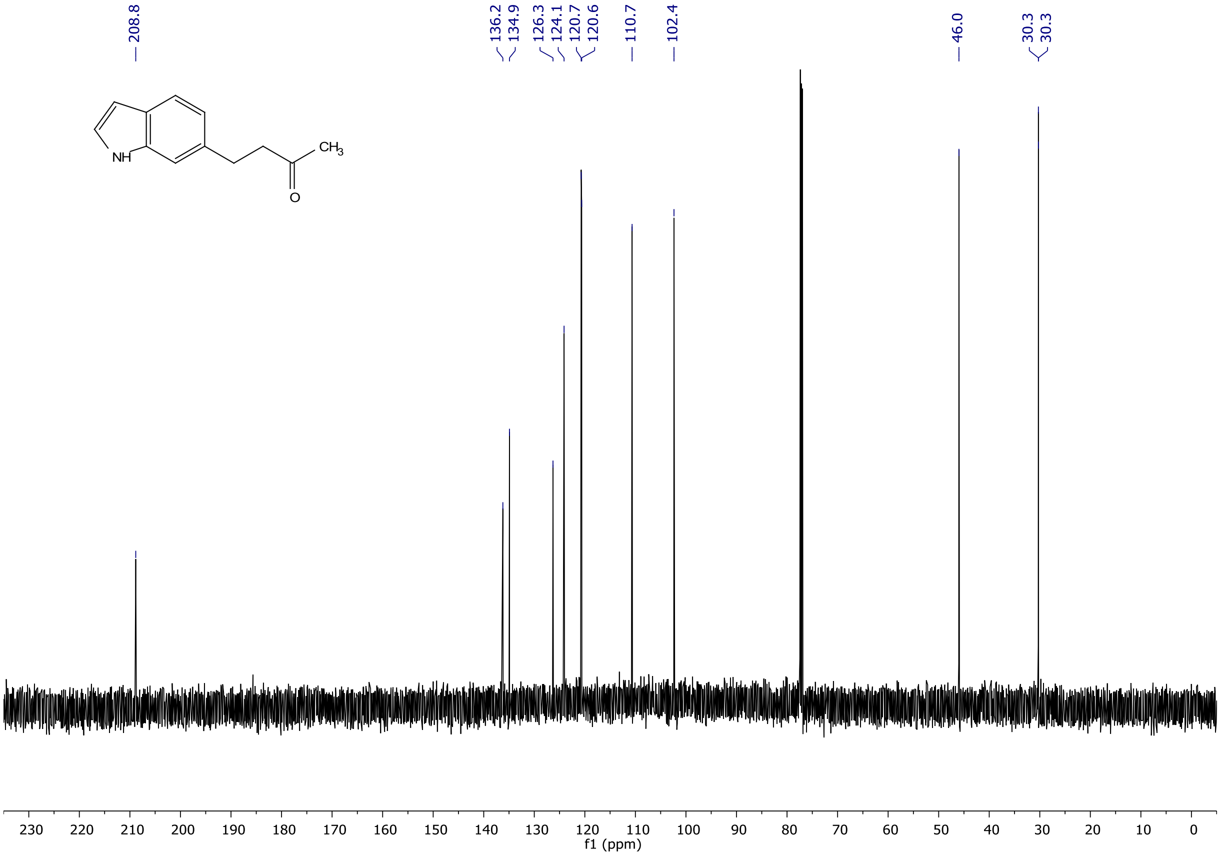

4-(dibenzo[b,d]thiophen-4-yl)butan-2-one (5ld)

^{1}H-NMR (600 MHz, $CDCl_3$)

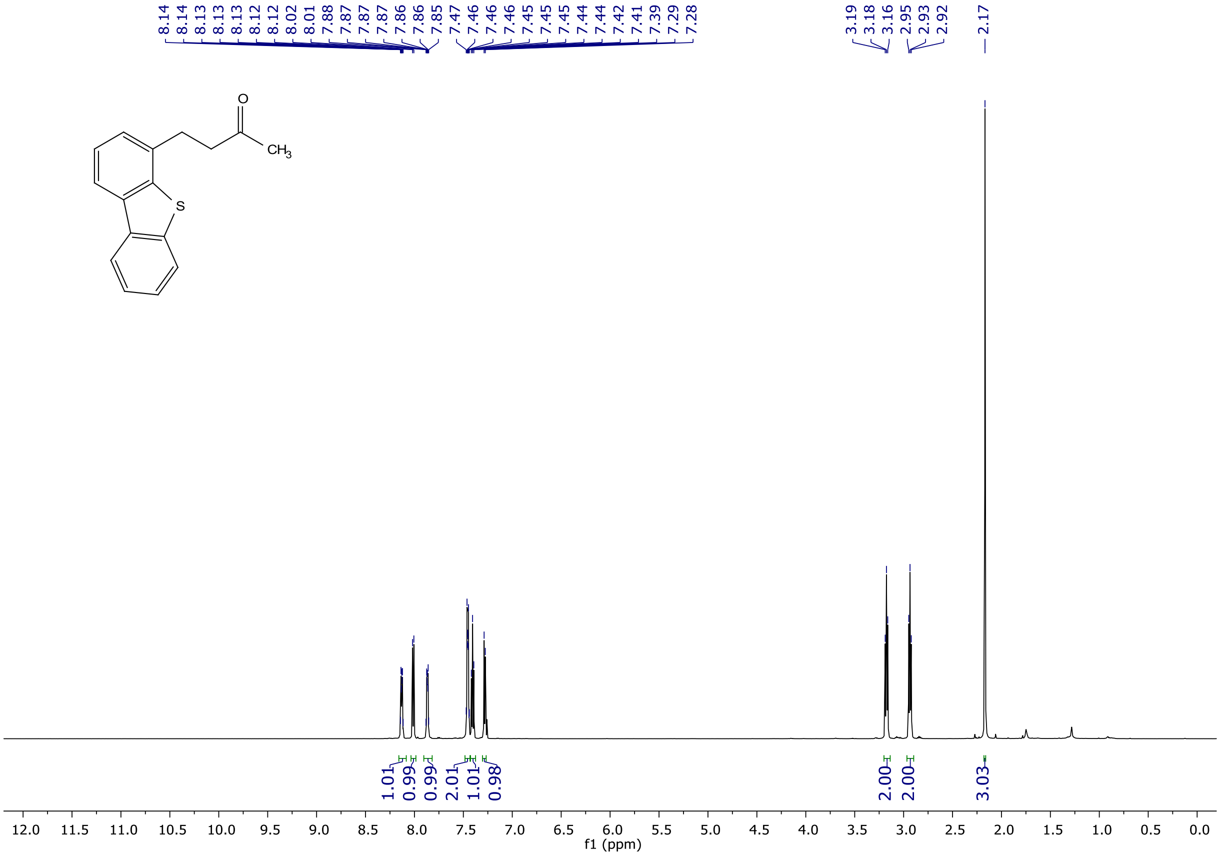

^{13}C-NMR (151 MHz, $CDCl_3$)

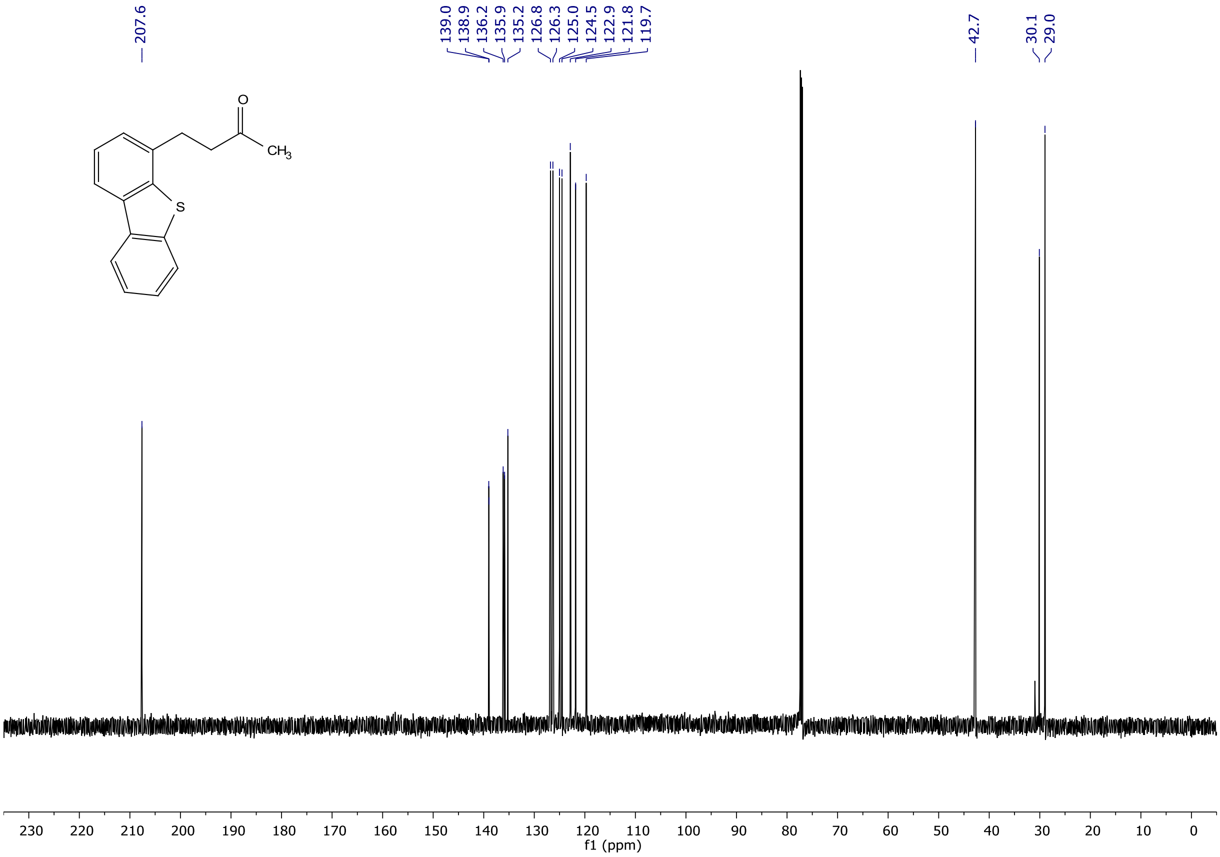

6-phenylhexan-2-one (5md)

^{1}H-NMR (600 MHz, $CDCl_3$)

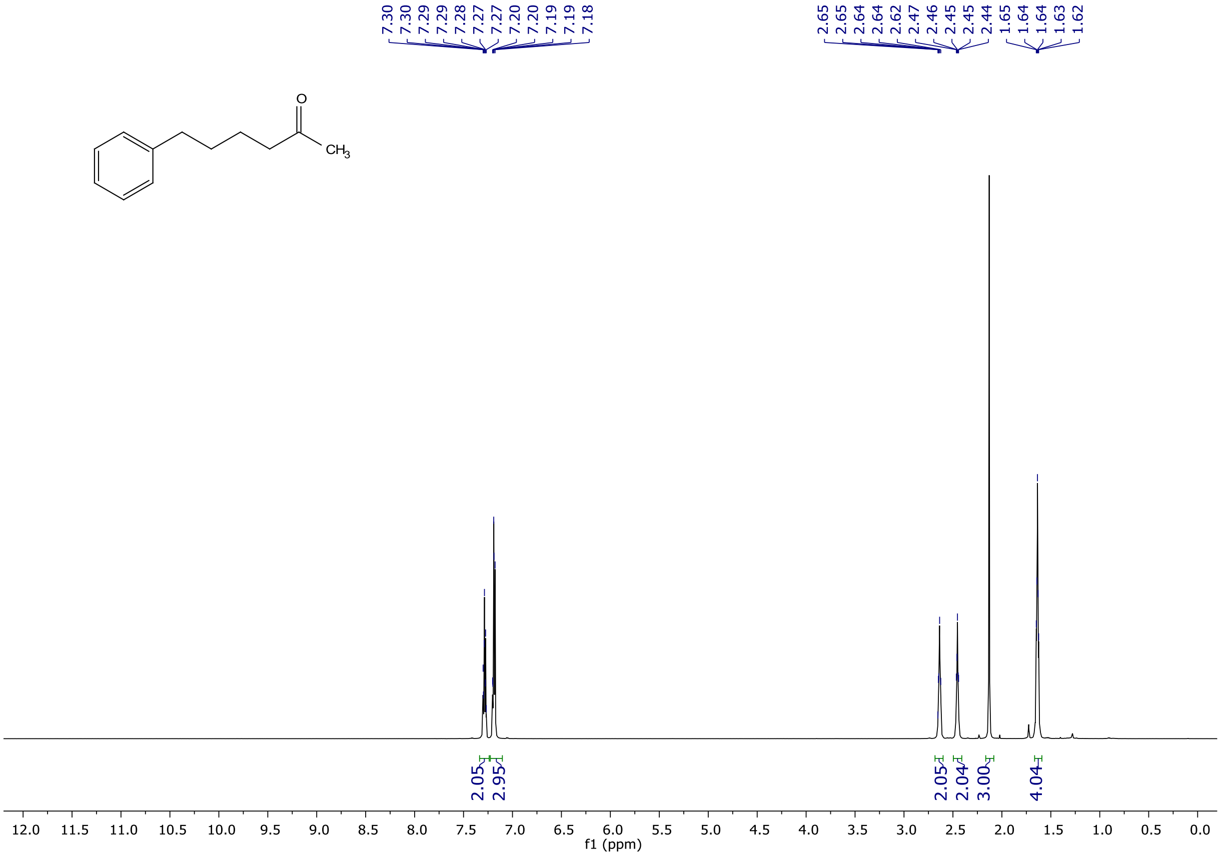

^{13}C-NMR (151 MHz, $CDCl_3$)

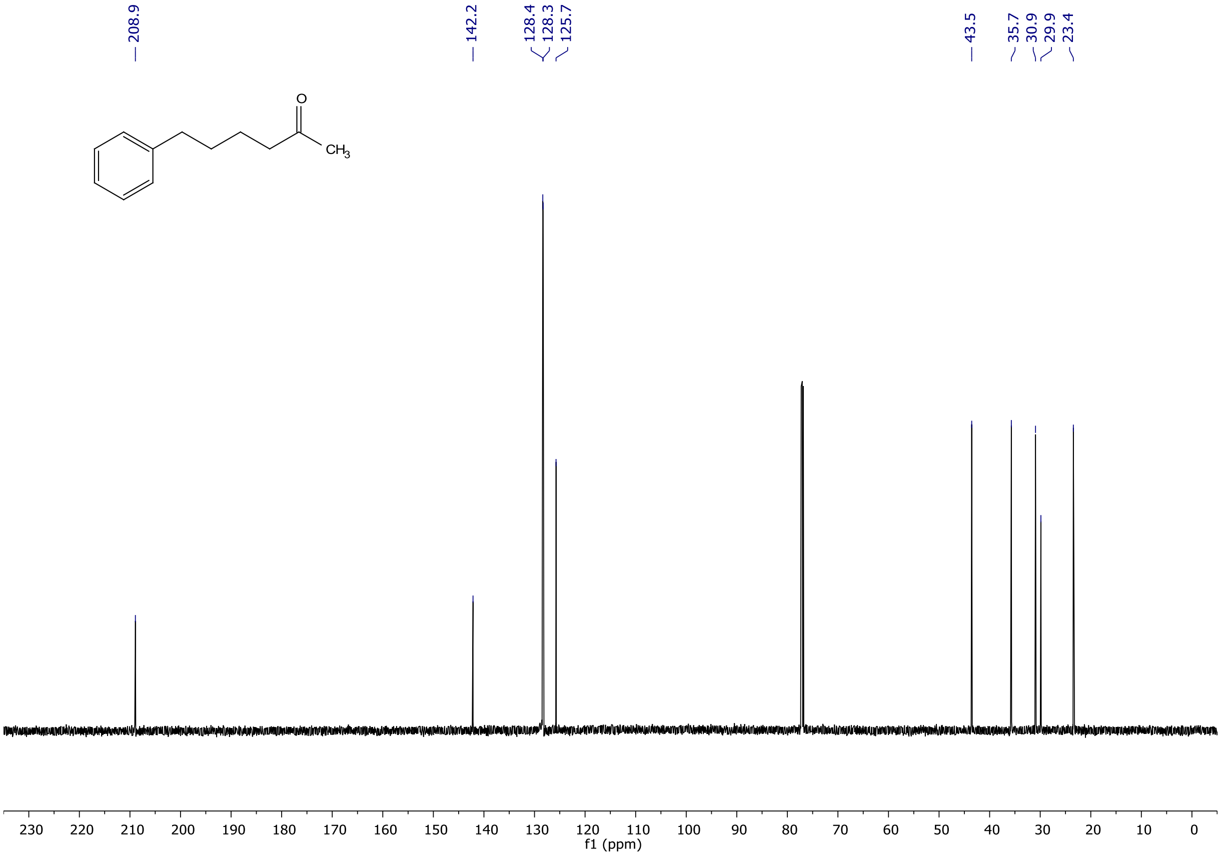

6-methylheptan-2-one (5nd)
^{1}H-NMR (600 MHz, CDCl3)

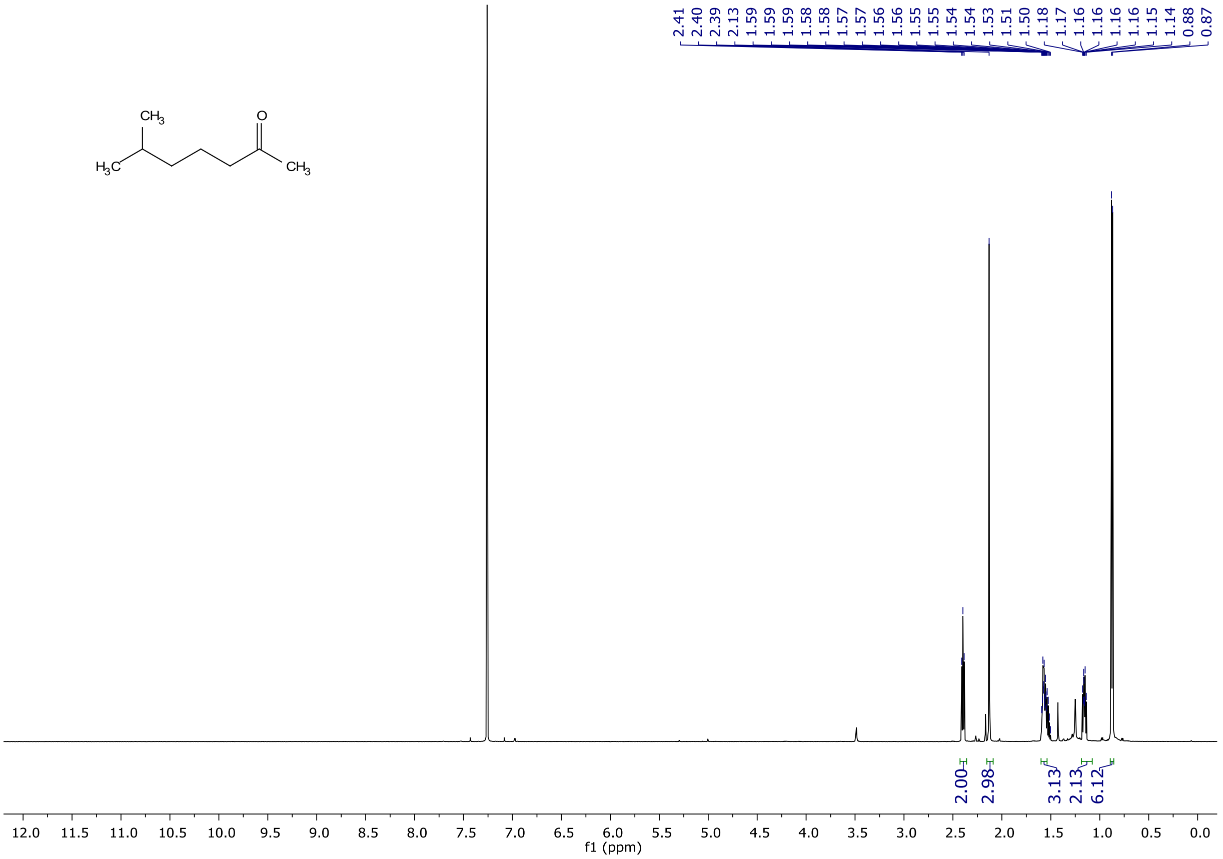

^{13}C-NMR (151 MHz, CDCl3)

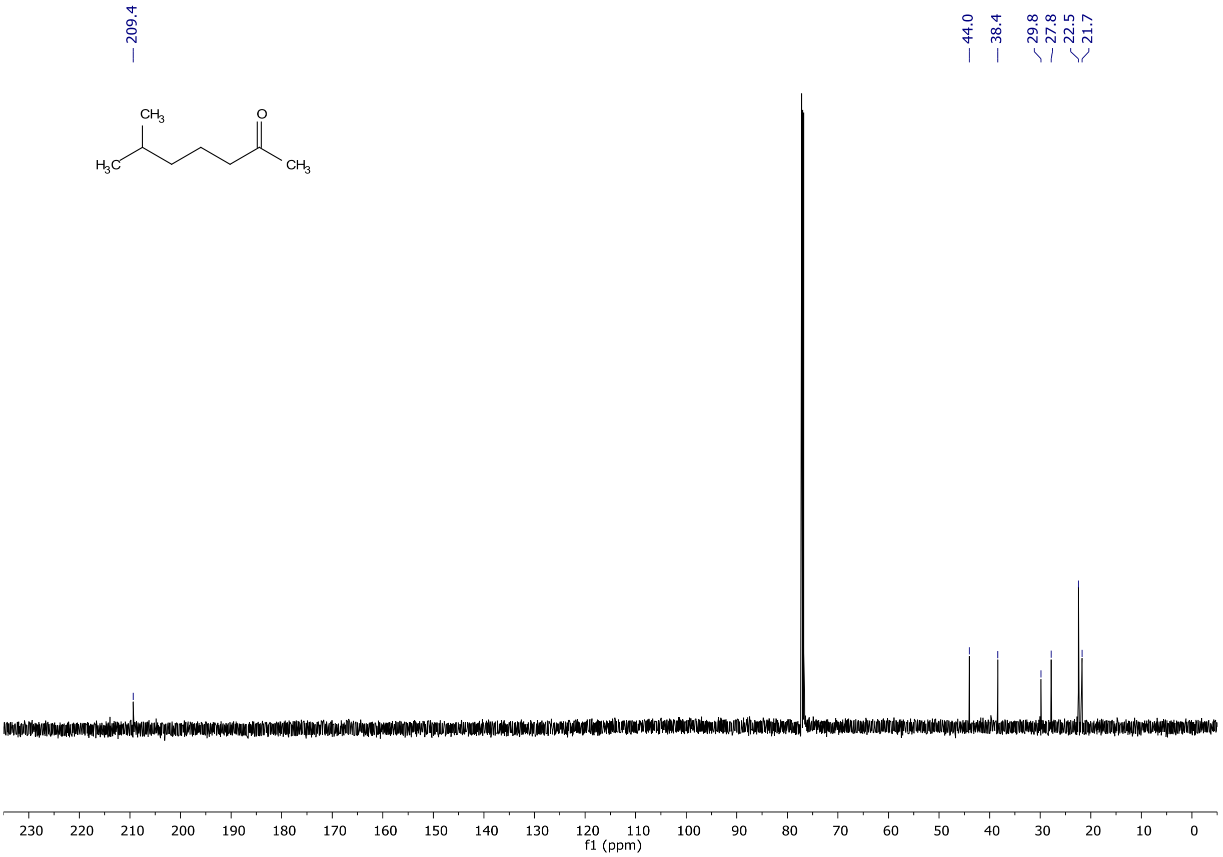

10-bromodecan-2-one (5od)

^{1}H-NMR (600 MHz, $CDCl_3$)

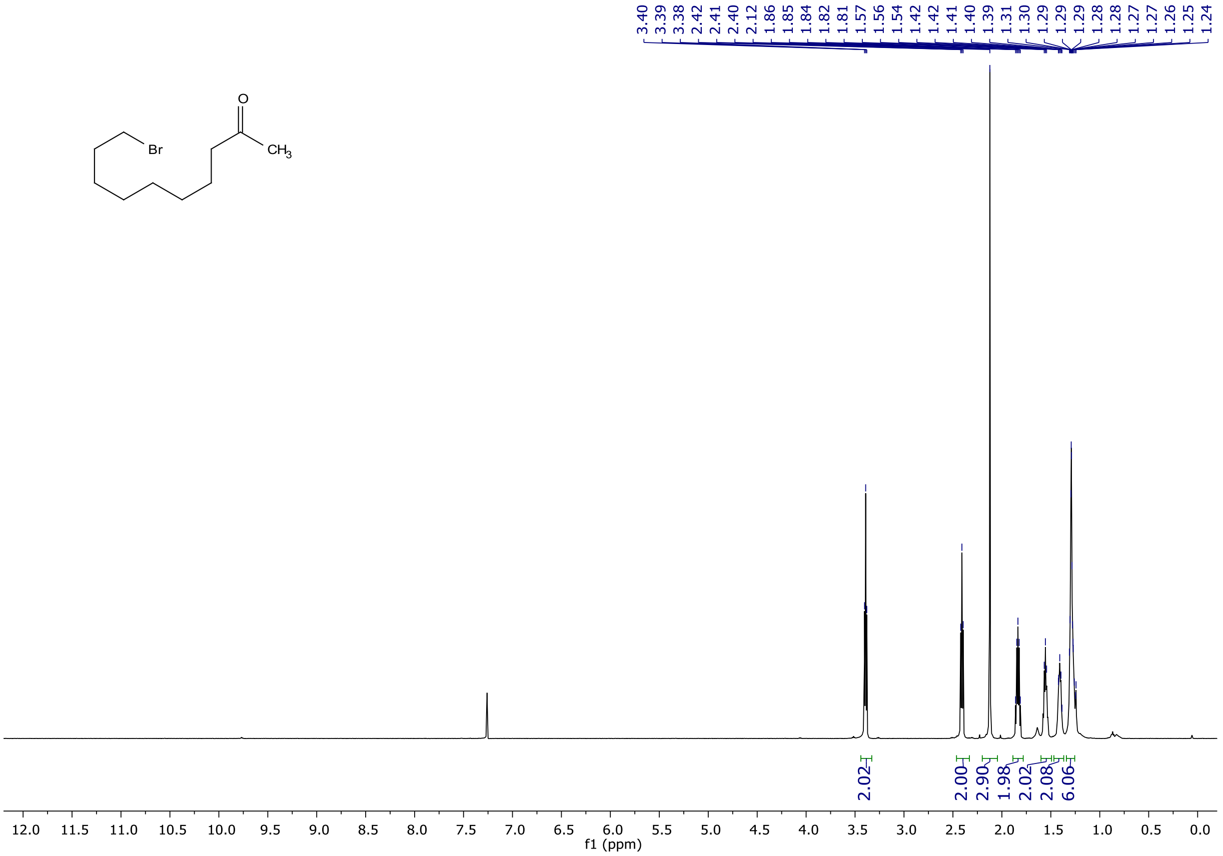

^{13}C-NMR (151 MHz, $CDCl_3$)

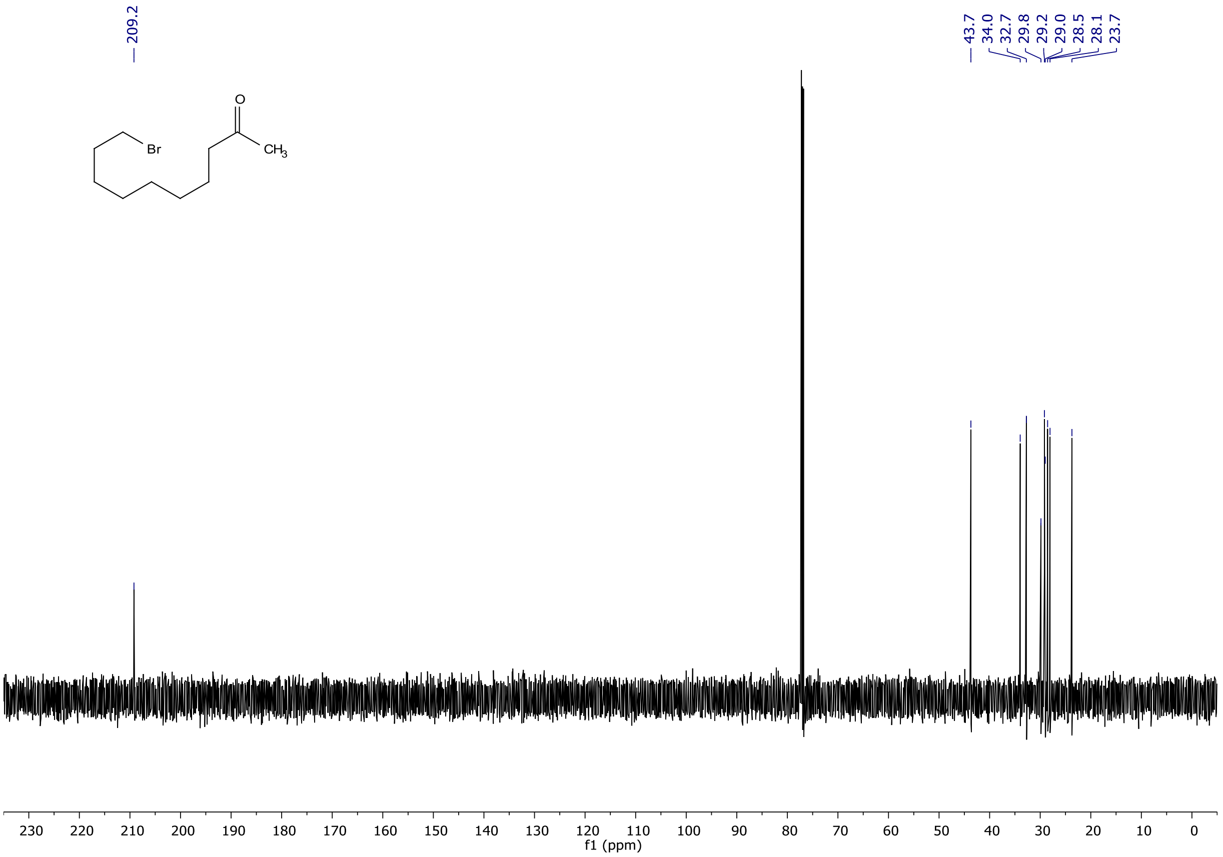

4-cyclobutylbutan-2-one (5pd)

^{1}H-NMR (600 MHz, $CDCl_3$)

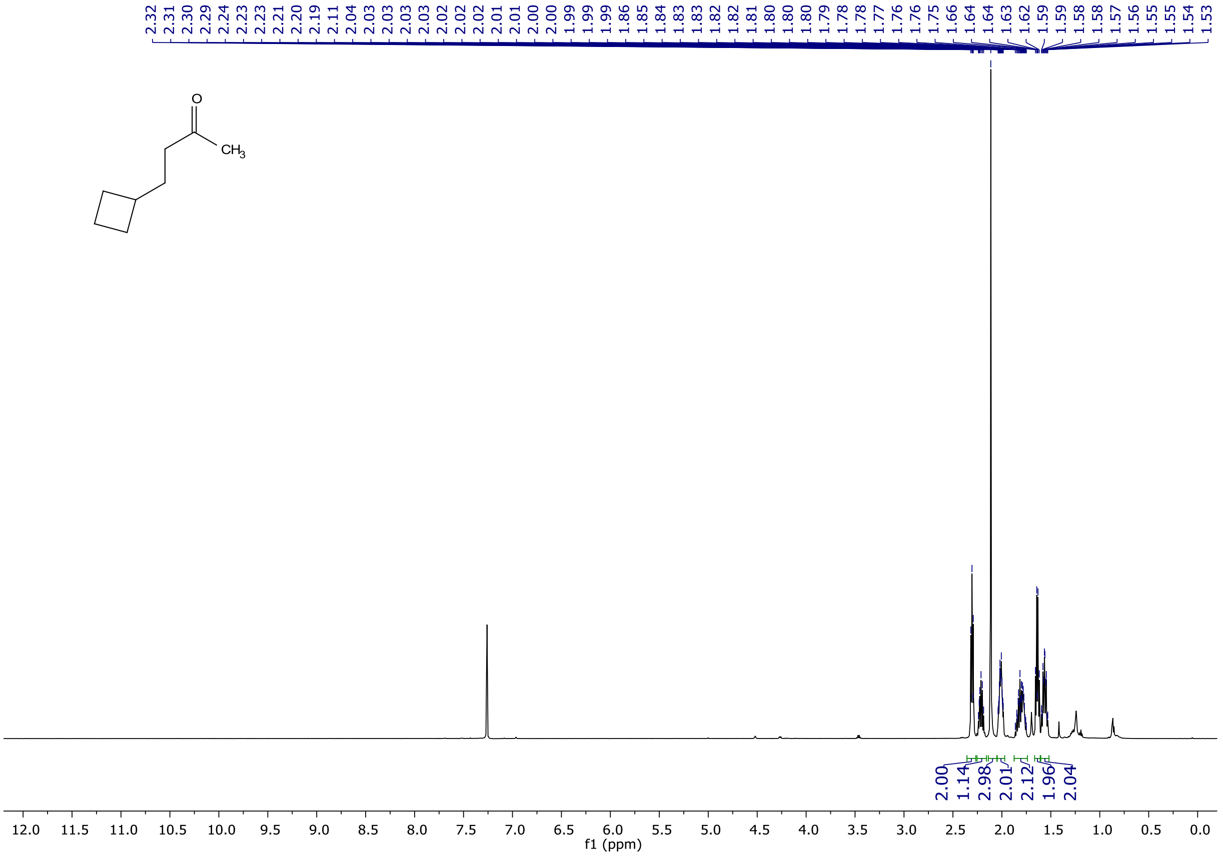

^{13}C-NMR (151 MHz, $CDCl_3$)

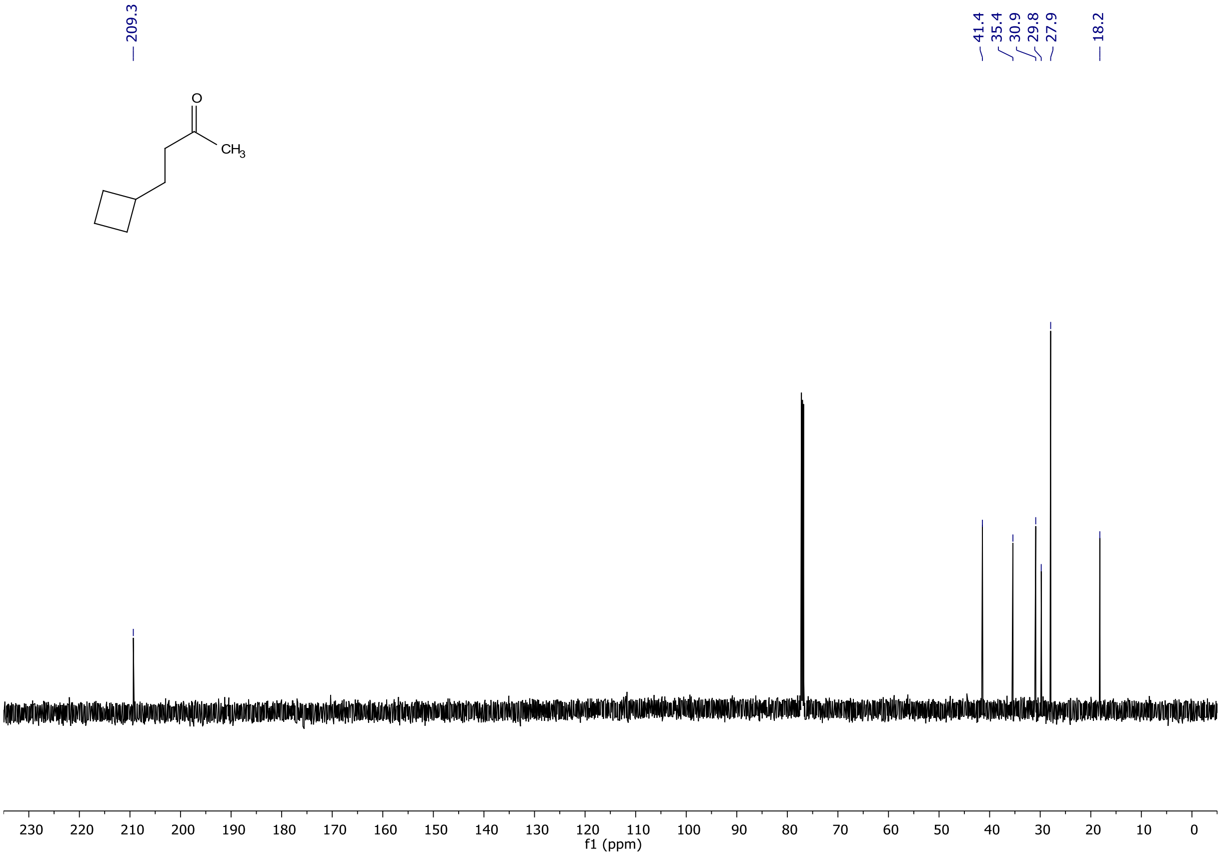

4-cyclohexylbutan-2-one (5qd)

^{1}H-NMR (600 MHz, $CDCl_3$)

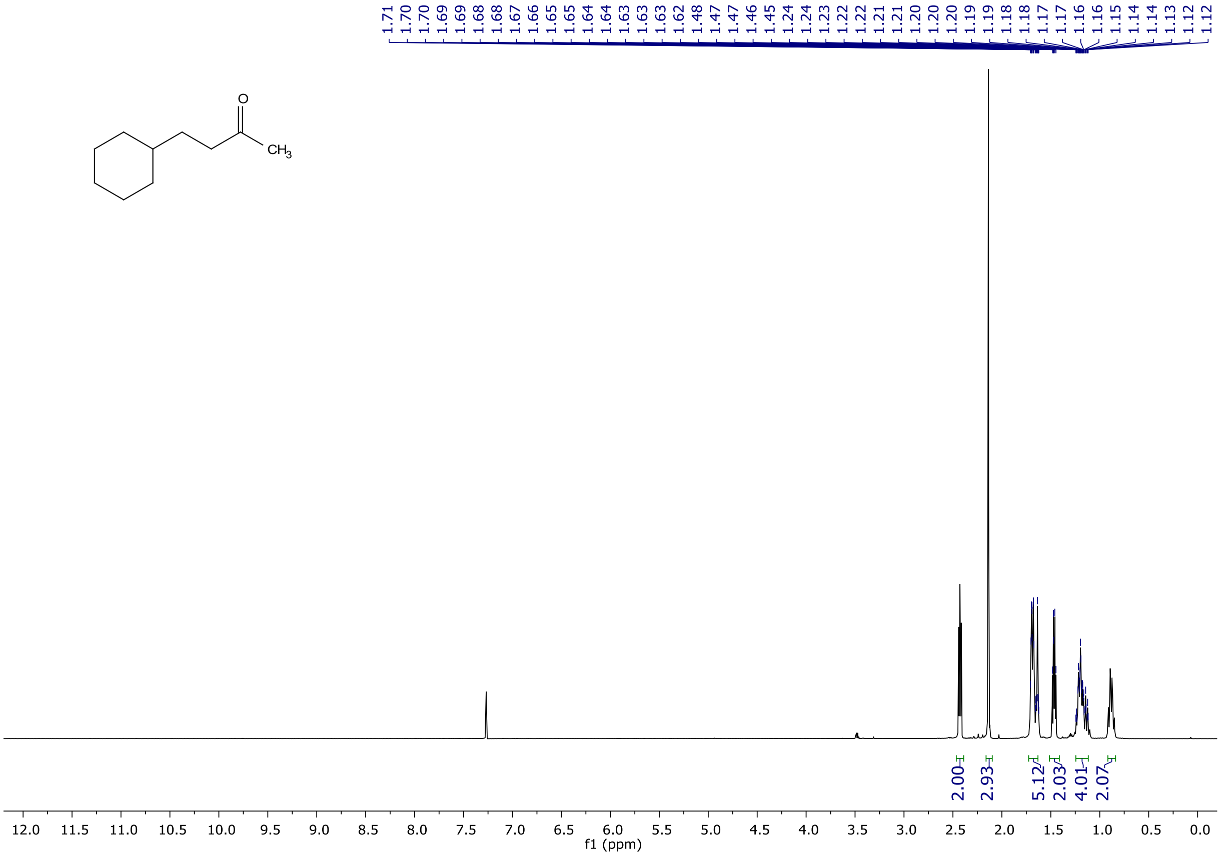

^{13}C-NMR (151 MHz, $CDCl_3$)

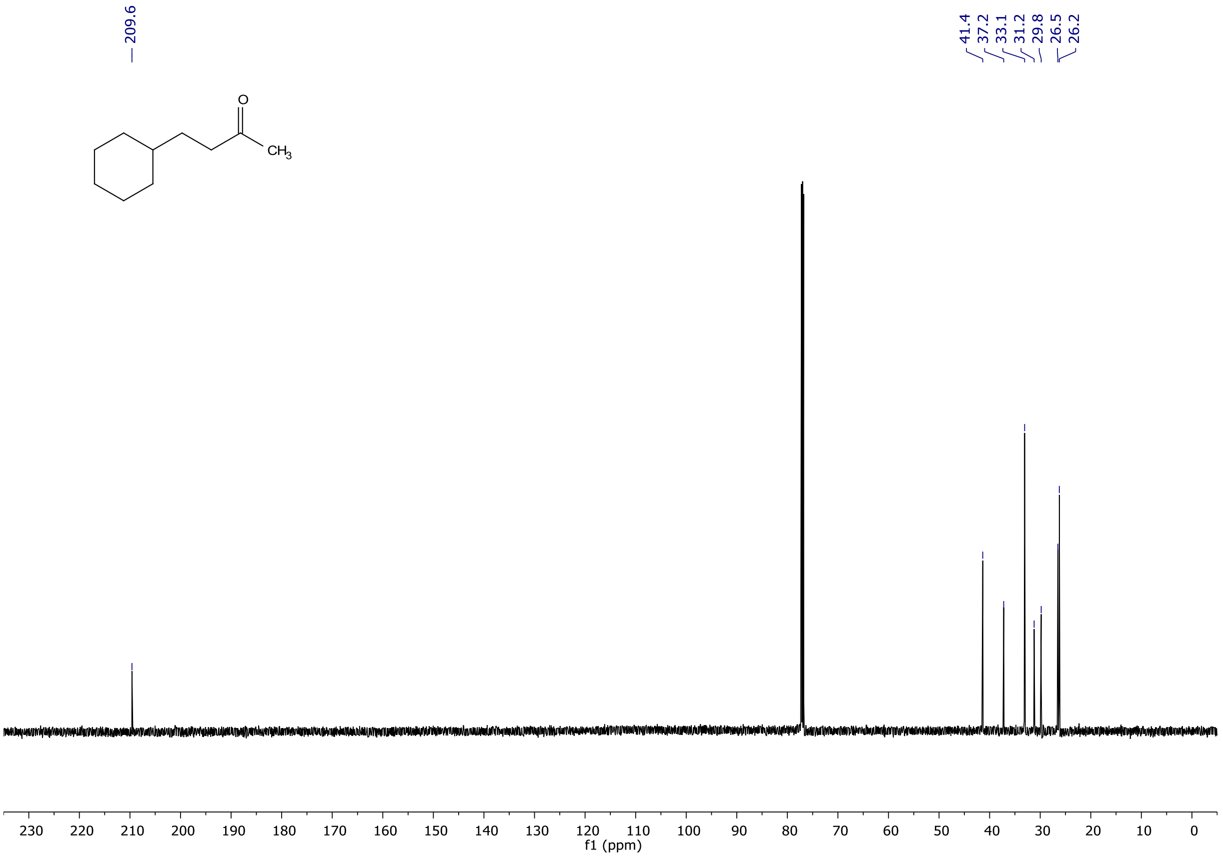

***tert*-butyl 2-(3-oxobutyl)pyrrolidine-1-carboxylate (5rd)**

^{1}H-NMR (600 MHz, $CDCl_3$)

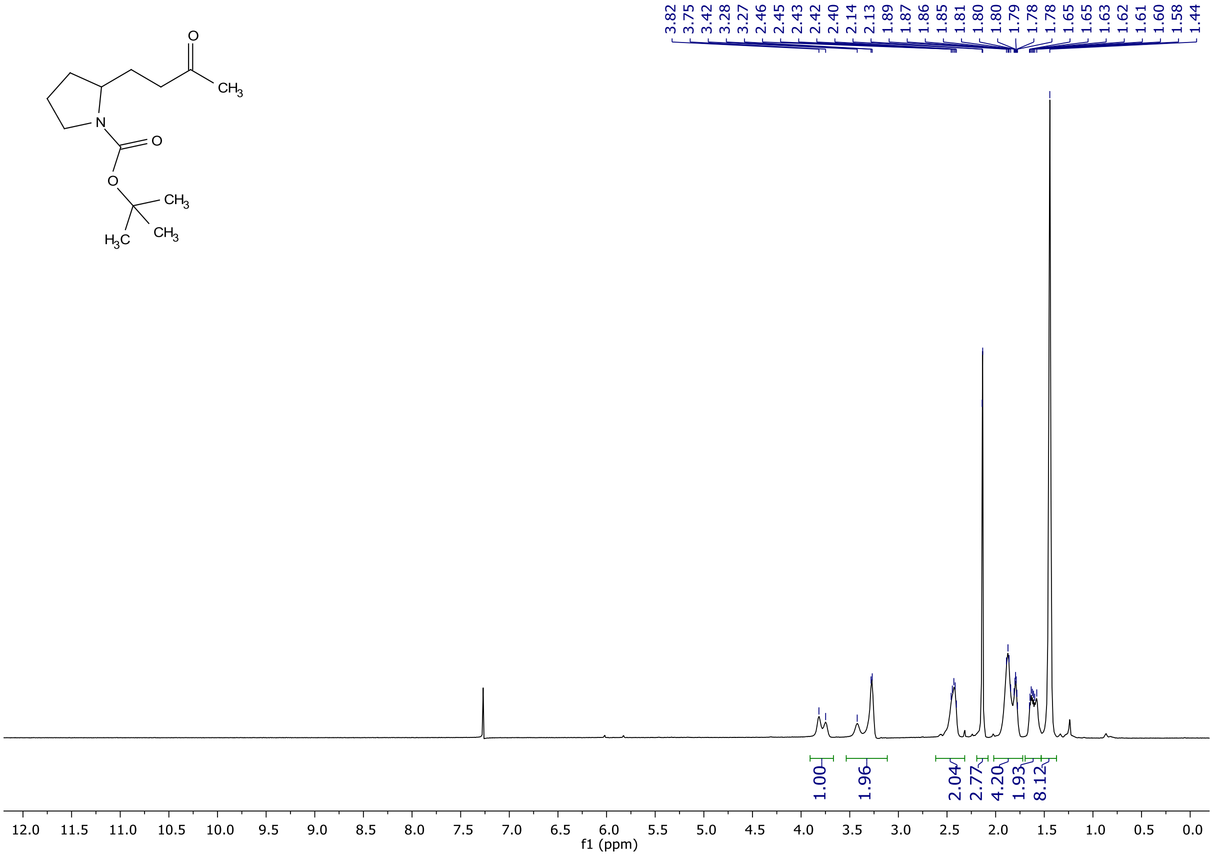

^{13}C-NMR (151 MHz, $CDCl_3$)

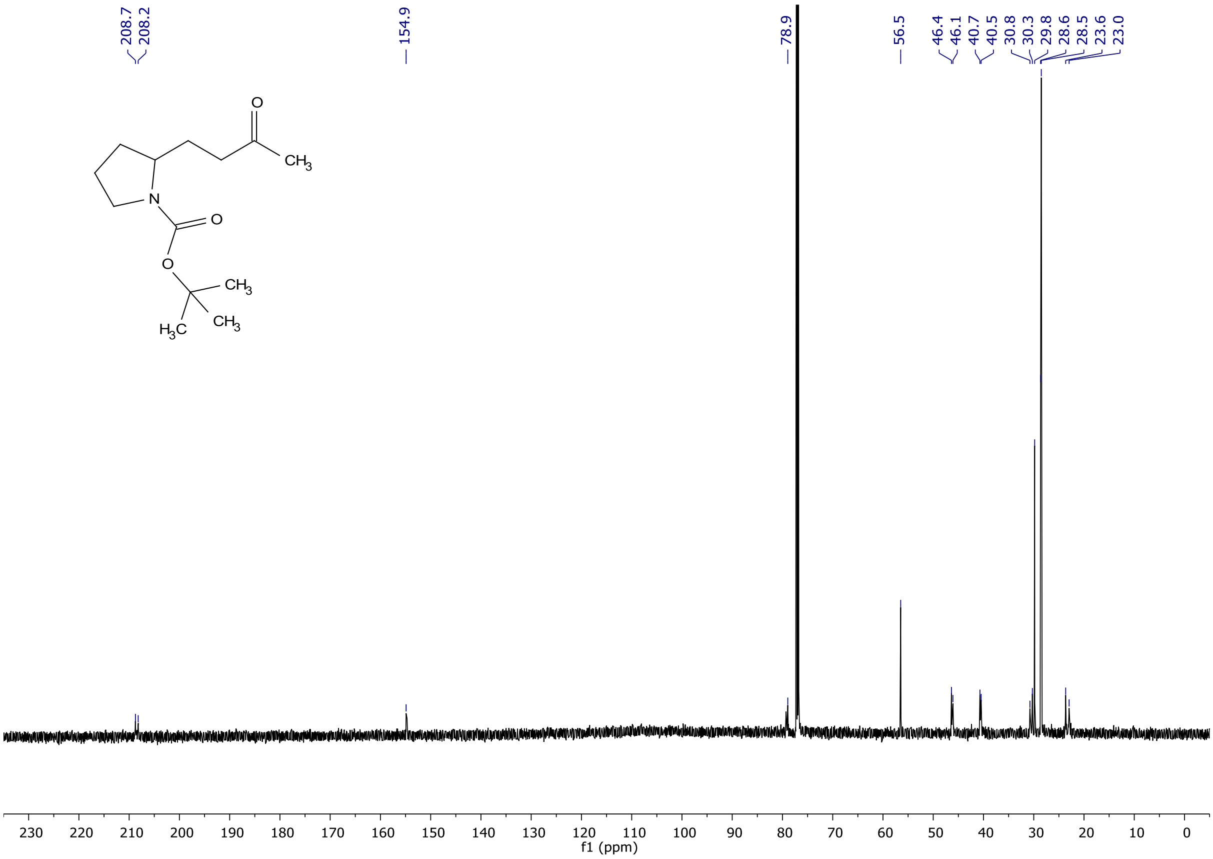

2,5-dichloro-N-(2-((2-methyl-7-oxooctan-4-yl)amino)-2-oxoethyl)benzamide (5sd)

^{1}H-NMR (600 MHz, $CDCl_3$)

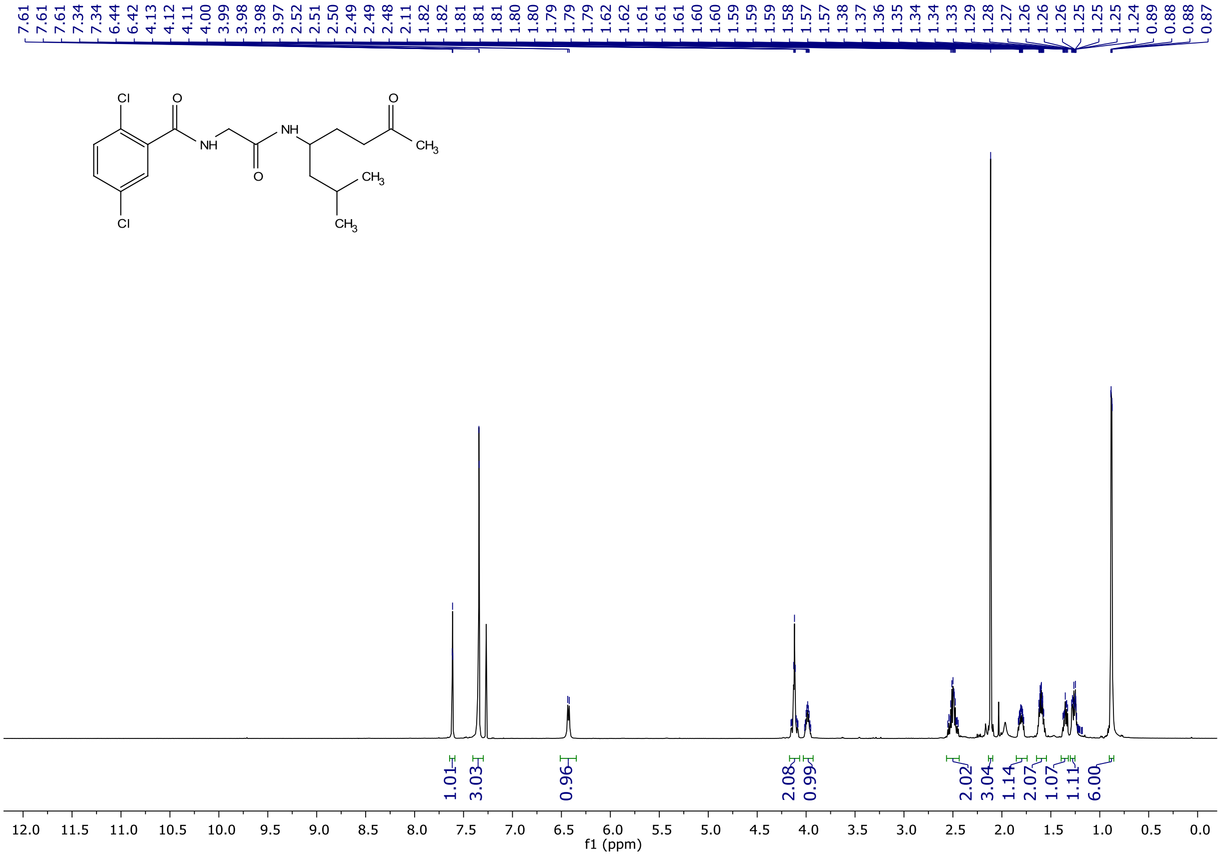

^{13}C-NMR (151 MHz, $CDCl_3$)

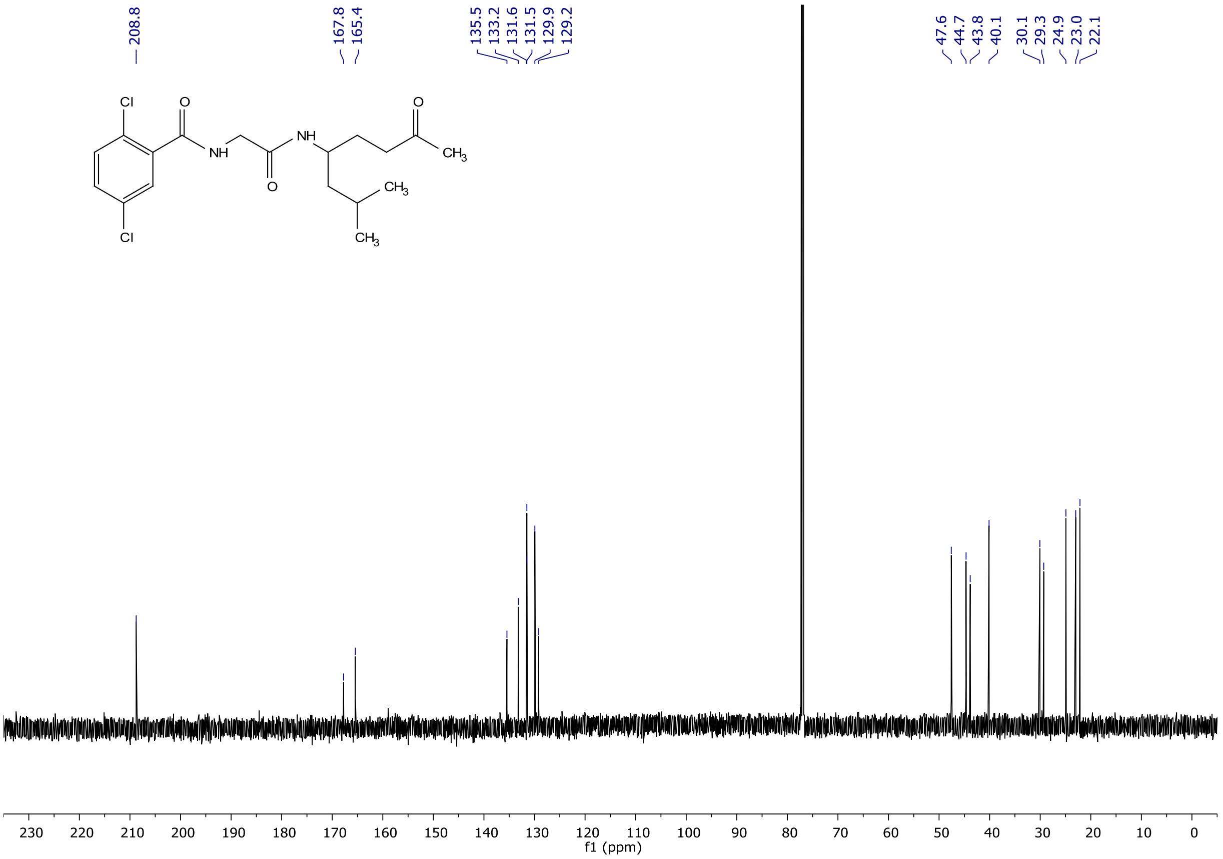

Supplement: Supplementary file 1 — Supplementary [file ANIE-56-15136-s001.pdf]
